# Luminescent Lanthanides in Biorelated Applications: From Molecules to Nanoparticles and Diagnostic Probes to Therapeutics

**DOI:** 10.1021/acs.chemrev.4c00615

**Published:** 2025-02-17

**Authors:** Carlson Alexander, Zhilin Guo, Peter B. Glover, Stephen Faulkner, Zoe Pikramenou

**Affiliations:** † Chemistry Research Laboratory, Department of Chemistry, University of Oxford, 12 Mansfield Road, Oxford OX1 3TA, United Kingdom; ⊥ Department of Chemistry, Hong Kong Baptist University, Kowloon Tong, Hong Kong SAR, China; ‡ Department of Materials Science and Engineering, Southern University of Science and Technology, Shenzhen 518055, China; § Defence Science and Technology Laboratory (DSTL), Porton Down, Salisbury SP4 0JQ, United Kingdom; ∥ School of Chemistry, University of Birmingham, Birmingham B15 2TT, United Kingdom

## Abstract

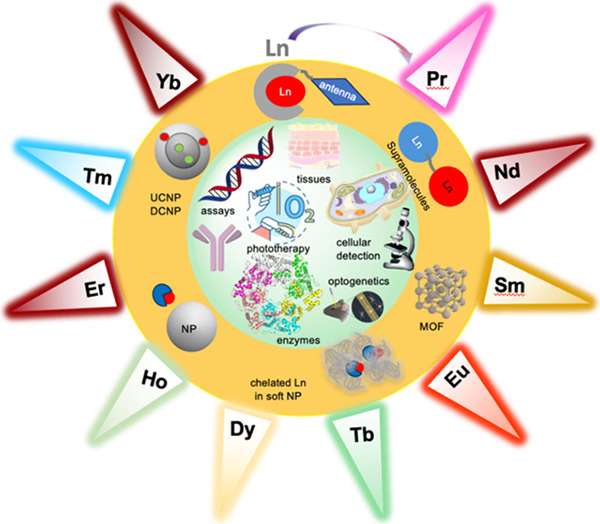

Lanthanides are particularly effective in their clinical applications in magnetic resonance imaging and diagnostic assays. They have open-shell 4*f* electrons that give rise to characteristic narrow, line-like emission which is unique from other fluorescent probes in biological systems. Lanthanide luminescence signal offers selection of detection pathways based on the choice of the ion from the visible to the near-infrared with long luminescence lifetimes that lend themselves to time-resolved measurements for optical multiplexing detection schemes and novel bioimaging applications. The delivery of lanthanide agents in cells allows localized bioresponsive activity for novel therapies. Detection in the near-infrared region of the spectrum coupled with technological advances in microscopies opens new avenues for deep-tissue imaging and surgical interventions. This review focuses on the different ways in which lanthanide luminescence can be exploited in nucleic acid and enzyme detection, anion recognition, cellular imaging, tissue imaging, and photoinduced therapeutic applications. We have focused on the hierarchy of designs that include luminescent lanthanides as probes in biology considering coordination complexes, multimetallic lanthanide systems to metal–organic frameworks and nanoparticles highlighting the different strategies in downshifting, and upconversion revealing some of the opportunities and challenges that offer potential for further development in the field.

## 1 Introduction

Lanthanide ions (Ln) have been widely applied in biological imaging and diagnostic assays as a direct consequence of the unique properties of open-shell 4*f* electrons. Lanthanide luminescence signals from visible to near-infrared (NIR) provide optical signatures that enable detection in spectral regions and time-domains inaccessible from other probes. The nature of *f–f* luminescence and choice of 4*f*-emitting lanthanides widen applications in a) disease diagnosis with multiplex detection advancing from *in vitro* to cellular assays, b) biological imaging with detection on the NIRII window (1000–1700 nm) allowing deep tissue imaging, and c) therapy with triggering bioresponsive function in cellular pathways. In this review, we explore how systems containing luminescent lanthanide ions can be used to probe biological environments through demonstrating a response to chemical change in their surroundings. We have considered lanthanide luminescence in systems ranging from molecular to supramolecular–nanomolecular constructs including metal–organic frameworks to nanoparticles. The latter are presented with designs ranging from soft matter to gold, silver, and silica nanoparticles which either incorporate chelated lanthanide complexes and down-converting particles (DCNP) or doping lanthanides which bring the unique properties of upconverting nanoparticles (UCNP). In this review, we have structured the sections based on their biological activity from lanthanide systems used in diagnostic assays as probes for cellular and tissue imaging, leading to bioresponsive functions in detection and therapy. Recent reviews have covered specific systems such as luminescent lanthanide complexes in sensing,^1,2^ theranostic properties,^3^ bioimaging,^4−6^ of coordination polymers,^7^ metal–organic frameworks,^8−10^ and nanocrystals/upconverting nanoparticles.^11−16^ Lanthanides in biochemical systems have not been covered in our review but have been reported elsewhere.^17−19^ We also have not covered the rapidly emerging field of lanthanide hydrogels^20^ or lanthanide in nanothermometer applications.^21−23^

**Figure 1:**
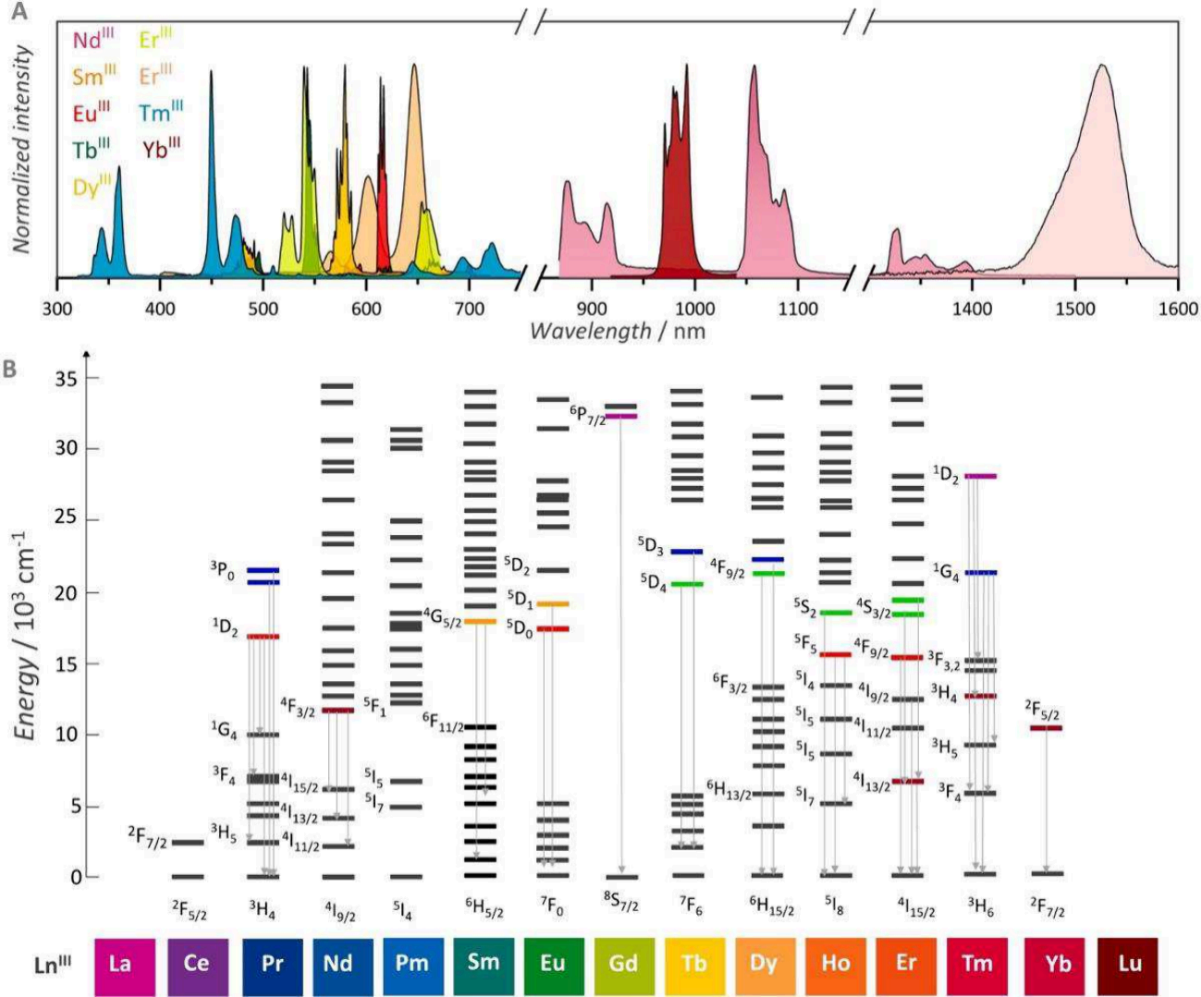
A) Fingerprint emission spectrum of lanthanides in visible and NIR regions; B) Partial Dieke diagram highlighting the energy levels arising from the 4*f*^n^ configurations of Ln(III) and their primary electronic transitions. Reproduced with permission from ref (24). Copyright 2025 Elsevier.

Lanthanide(III) luminescence covers a broad spectrum from ultraviolet (Gd) to visible (orange Sm, red Eu, green Tb, yellow Dy, blue Tm) and near-infrared (Pr, Nd, Ho, Er, Yb) (Figure 1).^24^ It originates from *f–f* transitions which are Laporte forbidden but they become allowed based on noncentrosymmetric interactions from the local environment. The different character of the transitions magnetic dipole vs electric dipole influences the intensity of the transitions with the latter being parity forbidden but become allowed based on the noncentrosymmetric environment. Most of the absorption and emission lines of the lanthanides are induced electric dipole transitions. The intensity of these transitions is particularly sensitive to the nature of the metal ion environment, and they can be either completely absent or very intense depending upon the ligand field and these transitions are called “hypersensitive”. The absorption coefficients are very low (1–10 L mol^–1^ cm^–1^), the lifetimes of the excited states originating from high multiplicity states are relatively long (microsecond to millisecond), and the emissive rates are slow which result in long-lived and narrow line-like emission bands.

All designs exploit the unique photophysical properties of the *f*-elements with the formally forbidden nature of such transitions giving rise to long luminescence lifetimes of the order of microseconds for most lanthanide ions and milliseconds for europium(III) and terbium(III) complexes. These properties can be exploited to achieve unique degrees of sensitivity and control, and in this review, we explore how their photophysics can be harnessed to good effect. The long lifetimes permit time-resolved separation of lanthanide-centered signals from scattered light and biological fluorescence, which occur on much faster time scales.^4,25−28^ Such time-gated approaches can give rise to very low detection limits. Furthermore, the narrow emission lines permit multiplexing of lanthanide probes, meaning that several probes can be studied simultaneously.^5,29−31^

**Figure 2:**
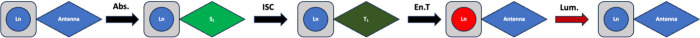
Triplet mediated energy transfer in a lanthanide–antenna system. Abs. = absorbance, ISC = intersystem crossing, En.T = Energy Transfer, Lum. = Luminescence.

In the vast majority of designs of molecular complexes, energy transfer from a sensitizing chromophore occurs via the triplet state of the chromophore, commonly known as the “antenna effect”^32−37^ (Figure 2) and in some cases through charge transfer states of *d*-metal complexes.^38^ Absorption of a photon of appropriate energy gives rise to the formation of the chromophore singlet state, which in turn forms the triplet state by intersystem crossing. Energy transfer to the lanthanide then occurs if it is energetically favorable, with surplus energy being dissipated through phonon assistance via the local vibrational manifold. For efficient and irreversible energy transfer, there should be spectral overlap between the triplet state and the lanthanide acceptor state, with a sufficient energy gap between the triplet and lanthanide emissive state to preclude Boltzmann repopulation of the triplet state. Energy transfer can occur through space (Förster energy transfer) or through bonds (Dexter exchange): the latter is generally favored where there is a through-bond pathway for exchange or superexchange.^39^ While triplet mechanisms dominate, ytterbium complexes can offer a special case in which energy transfer is mediated by a double electron transfer pathway if such a pathway is thermodynamically allowed.

**Figure 3:**
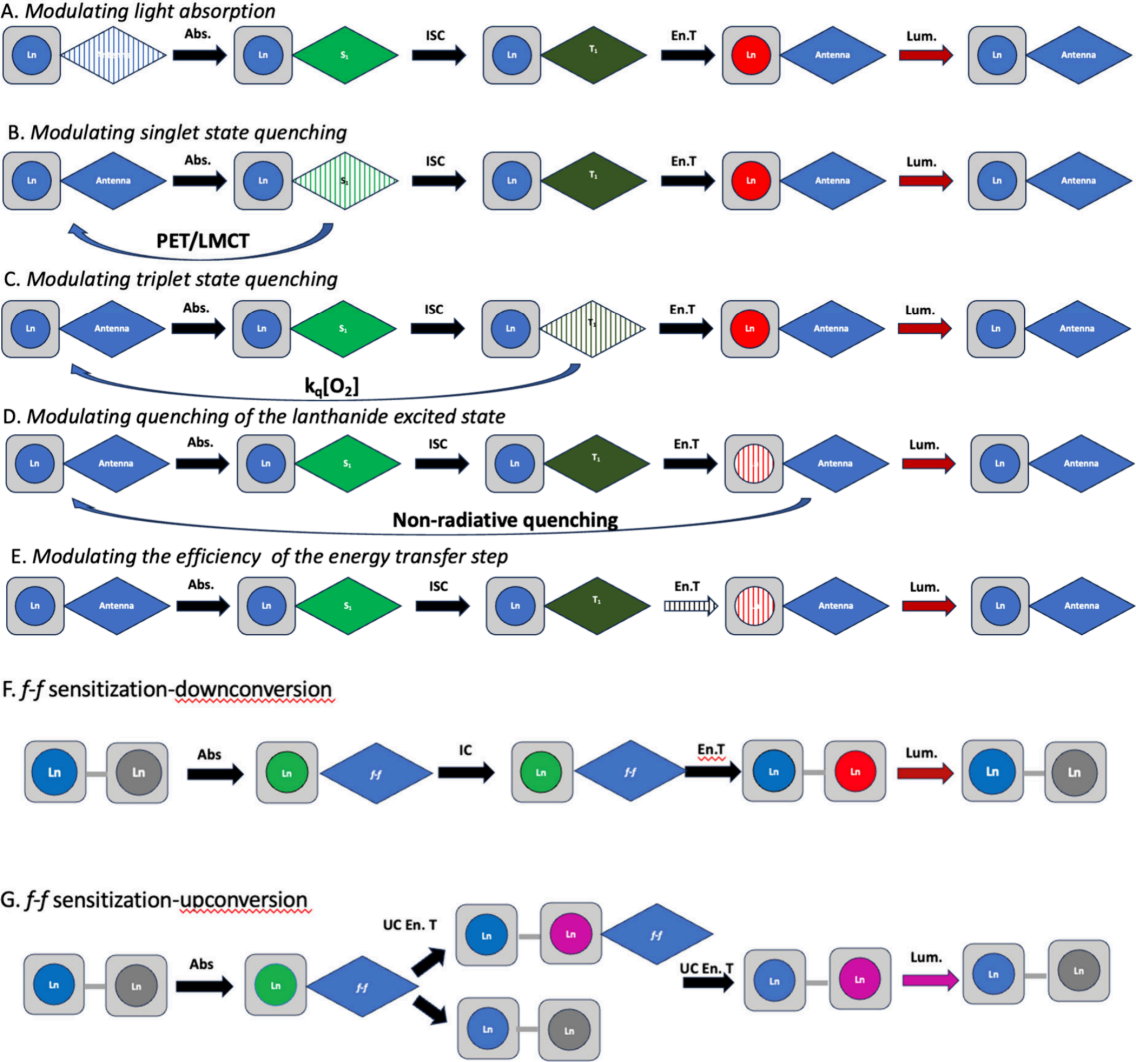
Possible perturbations to energy transfer and emission in lanthanide containing systems.

The multistep pathways associated with sensitized emission open a wide range of possibilities for developing responsive probes since perturbation of any step will change the overall outcome. Figure 3 illustrates a number of possibilities:

A. Chemical change to the chromophore will modulate the absorption of a particular wavelength of light. This change is a gate-keeper for the rest of the pathway, and increasing absorption will increase overall emission intensity, while decreasing absorption attenuates the signal.^40^ Furthermore, changes to the profile of the absorption spectrum open up the prospect of ratiometric response, allowing absolute quantification of an event by comparing the signal intensity following excitation at two different wavelengths.

B. Once an excited singlet state has formed, it can be quenched collisionally before intersystem crossing can occur. This most frequently occurs via photoelectron (PET) transfer quenching and has been used to observe change in chloride ion concentration.^40^

C. Similarly, the triplet state can be quenched by collisions with other triplets. In the context of nature, this most commonly involves quenching by molecular oxygen, which is unusual in having a triplet ground state. Triplet quenching can be important in two types of complexes, those in which the triplet state energy is sufficiently close to the emissive state of the lanthanide to permit rapidly reversible energy transfer,^41^ and those in which energy transfer is slow^42,43^ (e.g., as a consequence of long distance through-space energy transfer).

D. Once the lanthanide excited state has formed, changes to the local environment can perturb the probability of luminescence. At its most straightforward, this can involve displacement of solvent by coordination of a molecular guest-reducing nonradiative quenching by solvent oscillators, and increasing the luminescence intensity and lifetime.^33^ Alternatively, nonradiative quenching pathways can be added or removed; this is particularly common in europium(III) complexes where LMCT states are often lower in energy than the lanthanide emissive state.^40^ Europium constitutes a special case in other ways since the emissive ^5^D_0_ → ^7^F_2_ transition is hypersensitive to the lanthanide coordination environment, meaning that changes in ligand structure (or coordination of a guest) will change the intensity of this transition relative to others.^44,45^

E. Energy transfer from the triplet state to the lanthanide can be modulated in a variety of ways. Removing a through bond pathway for energy transfer ensures that less efficient Förster pathways must be followed,^46^ while changing the chromophore lanthanide separation can influence the efficiency of through space pathways.^47^ In the case of ytterbium(III), the existence of a mechanistic dichotomy between the triplet mediated and electron transfer mediated processes can be exploited by controlling the feasibility of the latter path.^48^

In multimetallic lanthanide systems, pathways for lanthanide luminescence signal sensitization can also involve *f*–*f* energy transfer pathways. These range from multimetallic molecular systems to nanosized lanthanide constructs or nanoparticle host matrices with doped luminescent lanthanides (Figure 3). The latter systems are dominated by upconverting nanoparticles (UCNPs) where many mechanisms have been postulated for the lanthanide sensitization.^49,50^ The main distinct pathways involved in designs of multimetallic lanthanide systems are summarized below:

F. Direct excitation of the lanthanide excited state either in the UV–visible or in some cases in NIR region leads to internal conversion in the manifold of *f*-excited states and subsequent energy transfer to the lanthanide with lower excited state levels which then luminesces. It is worth noting that although excited state absorption is weak, the internal conversion is fast and efficient with the energy transfer mechanism based on the Förster pathway based on the weak orbital overlap of the *f*-based donor–acceptor pair. In solid state nanoarchitectures, multimetallic systems overcome the issue of low molar absorptivity of the *f*-excited states with high local concentrations of the metals but also the proximity of the metal centers in the host matrix plays significant role in the efficiency of the energy transfer process. This approach is widely observed in downshifting conversion nanoparticle systems.

G. Near infrared (NIR) excitation of lanthanides is widely used in up-conversion systems. The most common approach and mechanism involves sensitizer–activator lanthanide pairs where NIR excitation absorbed by the lanthanide sensitizer is transferred to the activator lanthanide which then emits in the visible. This process is described as an energy transfer upconversion where the absorbed photon of the sensitizer in a metastable state is transferred to its ground-state and to the excited-state the activator lanthanide leading to its excitation on an upper emitting state. In this type of energy transfer, the concentration of the ions in a host matrix is important for the distance of the two sensitizer–activator pairs. The plausible mechanistic pathways for upconversion have been reviewed elsewhere,^49^ and we will not elaborate on the processes here.

The designs of the UCNP are influenced by the nature of the matrix and the position of the dopants in order to avoid deactivation pathways from other ions or phonon deactivation from the lattice. Fluoride host matrices have been the most popular with many designs based on core–shell structures manipulating the position of sensitizer–activator Ln(III) pairs such as Yb/Tm, Yb/Er, and Yb/Ho. Yb provides a unique advantage for excitation at the NIR, 980 nm, which makes it an attractive sensitizer for either upconversion or downshifting energy schemes for tissue penetration. It is important to consider both mechanisms influencing quantum efficiency in nanoparticle systems, especially those containing the Yb/Er pair. Upconversion via an intermediate lanthanide state relies on the intermediate state existing for long enough to permit absorption of a second photon. This state can also be quenched by emission, nonradiative decay, or downconversion to a lower lying acceptor state. In the case of Yb/Er pair, excitation of the Yb center at 980 nm can result in both upconversion to give Er-centered emission of visible light, or downconversion to give rise to erbium centered emission at 1530 nm. The challenges in the nanoparticle design involve not only the reduction on the phonon-assisted nonradiative pathways but also the intermetallic interactions accompanied by the external excitation source where intensity and pulse delays may influence the formation of the intermediate state and hence the subsequent pathways.

Circularly polarized luminescence (CPL) is gaining attention in recent times as it is important in biological applications to obtain information for the local chiral structural changes associated with the local environment surrounding the emitting species. From a chiral nonracemic luminescent molecule, CPL spectroscopy measures the difference in luminescence intensity of left circularly polarized light versus the right circularly polarized light. Owing to the difficulty in measuring the absolute emission intensities, the degree of CPL is reported (quantified) as luminescence dissymmetric ratio (or factor), *g*_*lum*_.^51^ This represents the ratio of the difference in emission intensity to the average total luminescence intensity. Larger *g*_*lum*_ values occur when the transition considered is electric dipole forbidden but magnetic dipole allowed. Among the emissive lanthanides, the ^5^D_0_ → ^7^F_1_ transition in Eu(III) exhibits high *g*_*lum*_ as it is a magnetically allowed transition. However, the *g*_*lum*_ values in lanthanide complexes are generally found to be low in water and dependent on the local environment at the lanthanide center. Accordingly, the most dramatic effects are observed in rigid systems where there is a significant change in ligand donor set or helicity at the lanthanide center.

For bioimaging and assay applications, there are important factors to consider such as the strength of the lanthanide signal and the solubility of the complex. Lanthanide luminescence is quenched by high energy vibrations of coordinating and second sphere ligands, such as O–H, N–H, and C–H, restricting molecular designs to high lanthanide coordination numbers to avoid water coordination and nanoparticle designs to introduce protective shell coatings. For molecular complexes, it is challenging to compare the values of quantum yields (which range from 1–70%), some performed in an integrating sphere or by relative comparisons with standards. Many reported values are performed in nonaqueous solvents or conditions which do not replicate the biological environment (temperature, solvent, pH). Additionally, the quantum yield measurements need to consider wavelength excitations consistent with microscope conditions to provide an overall evaluation of comparison of the probes’ performance. Brightness defined by the product of the extinction coefficient and the quantum yield at a given wavelength represents the amount of light effectively available for microscopy imaging, and it is a more comprehensive presentation of light emission potential of the complexes. On nanoparticles, the sizes and lanthanide designs vary, and rationalization is mainly achieved for similar systems (e.g., in upconverting nanoparticles, the comparisons are restricted by the nanoparticle design and the size).

Aqueous solubility of the lanthanide complexes is challenging to achieve, and the charge of the metal complex plays an important role. Additionally, some molecular designs for bioimaging involve lipophilic targeting groups to enhance penetration by cell membranes; hence, hydrophilicity tends to be compromised. A balance of hydrophilicity is important to achieve the best results. It is important to note that DMSO is commonly used to dissolve compounds for bioimaging in order to improve solvation; however, care shall be taken of its potential toxicity effects due to its oxidation by reactive oxygen species.^52^

**Figure 4:**
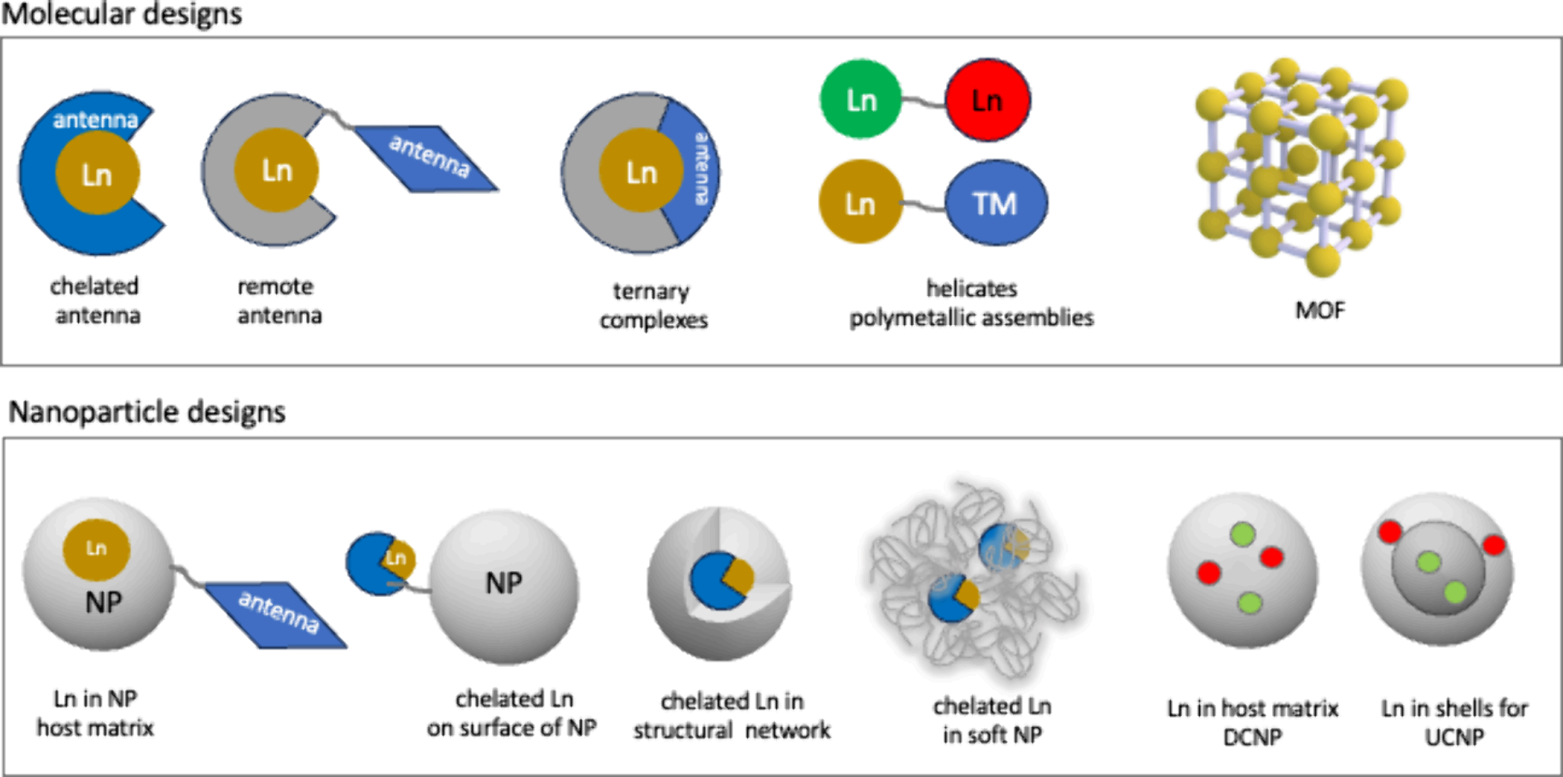
From molecular to nanoparticle designs of lanthanide sensitization schemes.

Lanthanide probes have been incorporated in molecular, supramolecular, and nanoparticle designs with aims not only to increase luminescence signal efficiency but also probe biological recognition and detection or stimulate localized events. Designs range from antenna organic sensitizer ligands to pendant groups, helicates, multimetallic lanthanides, and dendrimers to metal–organic frameworks and nanoparticles with either incorporated lanthanide complexes or host matrices doped with ions (Figure 4).

In this review, we describe a range of systems, from molecular complexes to polymetallic assemblies and nanoparticles, that exploit these pathways in a variety of ways, focusing on biologically relevant interactions. In Section 2, we provide an overview of the lanthanide systems used in detection and assays structured around detection of nucleic acids, enzyme monitoring, and biomarker recognition, mainly focused on molecular complexes for *in vitro* assays. Section 3 reviews molecular to nanosized probes for cellular imaging, moving to Section 4 where the focus is on tissue imaging. Finally, Section 5 describes lanthanide systems in bioresponsive assays and tissues where cellular diagnostics, light-activated biological activity, and therapeutic applications are reported as *in vitro* and *in vivo* examples.

## 2 Lanthanide Probes in Diagnosis and Detection

### 2.1 Lanthanide Complexes in Bioassays

As discussed, the long-lived luminescence from lanthanide ions can be separated from short-lived processes such as scatter and autofluorescence using time gating techniques. For almost half a century, scientists have exploited this property to develop lanthanide-based ultratrace assays that can be used in screening. These build on the principles of immunoassay that have been established over many years, initially using radioisotopes and subsequently organic fluorophores, but for the purposes of this review, we will focus on introducing the basic principles of the use of lanthanide ions in such applications.

**Figure 5:**
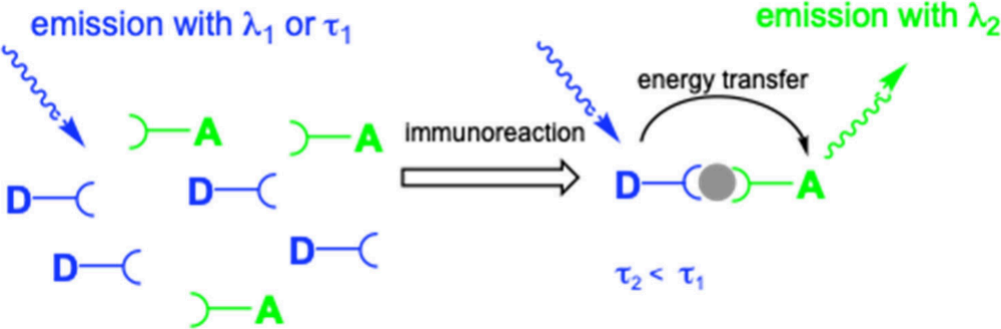
Schematic illustration of a typical FRET “sandwich” assay.

Luminescence-based assays rely on a series of recognition events and generally exploit the concept of Förster (Fluorescence) resonance energy transfer (FRET) through space between two chromophoric units that are assembled as a consequence of interaction between an antibody and an antigen (also known as sandwich assay) with one chromophore as an energy donor while the other acts as an energy acceptor (Figure 5). The rate and the efficiency and energy transfer are determined by the separation distance between the two chromophores and the spectral overlap between the emission spectrum of the donor and the absorption spectrum of the acceptor. In general, lanthanide ions are most frequently used as energy donors in such systems due to their long lifetimes which are easily monitored and the narrow spectral lines associated with lanthanide absorption that tend to make them relatively ineffective as energy acceptors.

Early work by Leif et al.^53^ focused on the use of europium complexes with 1,10-phenanthroline and β-diketonates as tags for antibodies. This revealed the potential of using long-lived luminescence, but the kinetic instability of the complexes proved a serious limitation, particularly in the presence of competitor ligands. From this early work, several requirements became apparent: kinetically stable systems, functional groups appended to such systems facilitate binding, and longer wavelength absorption are desirable to avoid excessive autofluorescence and permit the use of glass optics.

**Figure 6:**
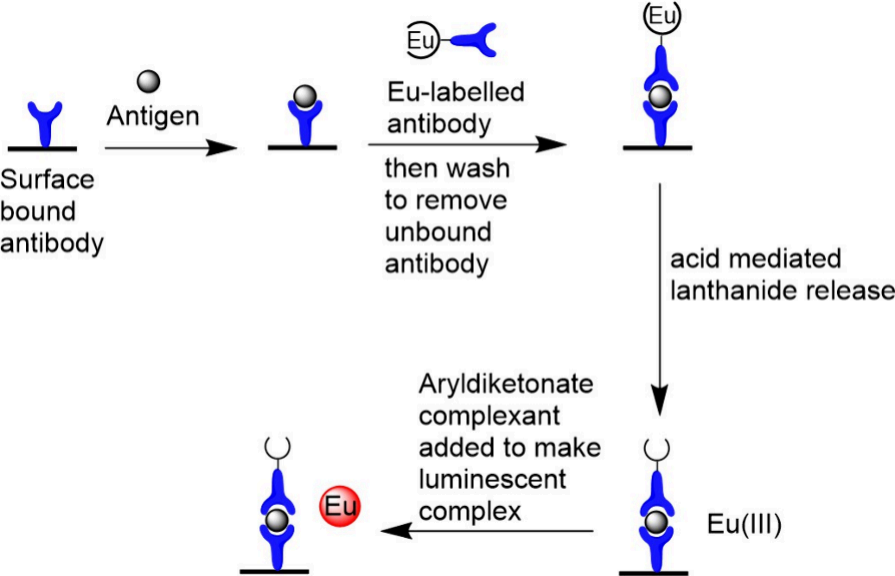
Schematic illustration of the heterogeneous DELFIA assay.

The first assay to be commercialized was the DELFIA assay.^29,54,55^ DELFIA (dissociation enhanced lanthanide fluorescence immunoassay) is a heterogeneous assay which uses a lanthanide complex based on aminocarboxylate ligands such as EDTA, EGTA, or DTPA, linked to the antibody by reaction of appended isothiocyanate groups with nucleophilic residues (particularly amines) on the protein surface. These polydentate ligands lend increased stability to the lanthanide complexes used in the assay and bind strongly to lanthanide ions around physiological pH. The assay (Figure 6) uses well plates coated with an unlabeled antibody to which an antigen can bind. The lanthanide-conjugated antibody is then added, and the mixture incubated. When the target antigen is present, it acts to link the lanthanide-conjugated antibody to the surface of the plate via the unlabeled antibody. Washing the plate removes any free (uncomplexed) antibody so that the remainder is used to quantify the amount of europium present and hence the concentration of the target antigen. This is achieved by lowering the pH to less than 4, leading to protonation of the ligand, releasing the lanthanide. The amount of lanthanide (and hence the antigen) is determined by treatment of the free lanthanide with an aryl diketonate, which sensitizes the lanthanide luminescence; the luminescence intensity is directly proportional to concentration.

The ideas underpinning the DELFIA^Ⓡ^ assay have spawned a wide variety of other approaches, including the CYBERFLUOR assay,^56^ which incorporates the lanthanide binding domain into an antibody conjugate, removing the need for “developing” the assay with a separate ligand.

The field of lanthanide bioassay has been widely reviewed,^57−60^ so this review focuses on a range of illustrative examples of bioresponsive systems.

### 2.2 Luminescent Lanthanides in Their Interaction with Nucleic Acids

Luminescent probes for nucleic acid recognition are attractive for *in vitro* diagnostics development but also have potential for *in cellulo* imaging, monitoring localization and activity, with potential therapeutic activity. We have summarized the role of luminescent lanthanides in their interaction with nucleic acids primarily based on the lanthanide coordination environment in order to give an overview of strategies and approaches for detection. In our review, we considered systems where lanthanides coordinate directly to nucleic acids, lanthanides as coordination complexes and finally lanthanide nanoparticle constructs (Table 1).

**Table 1:**
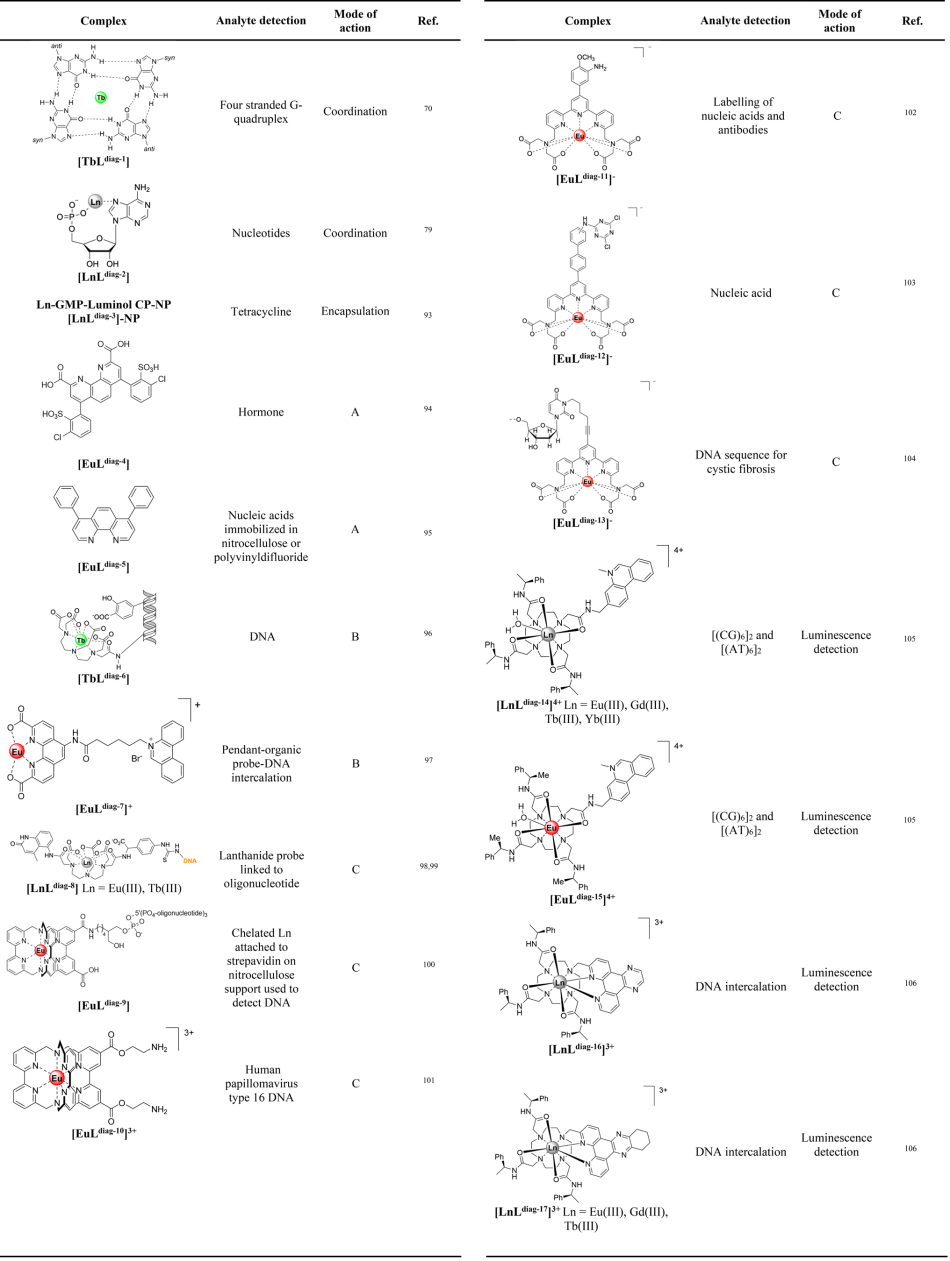

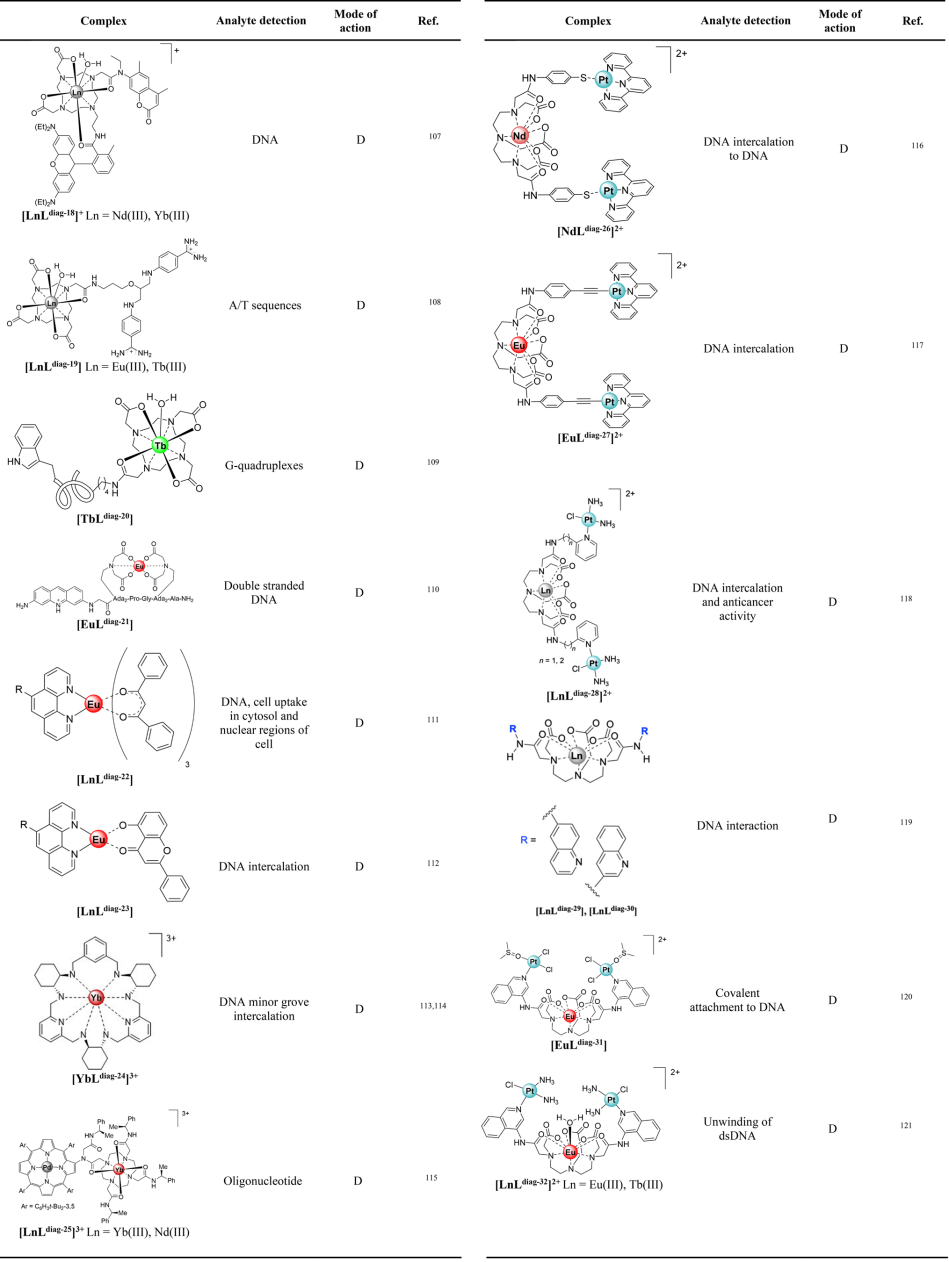

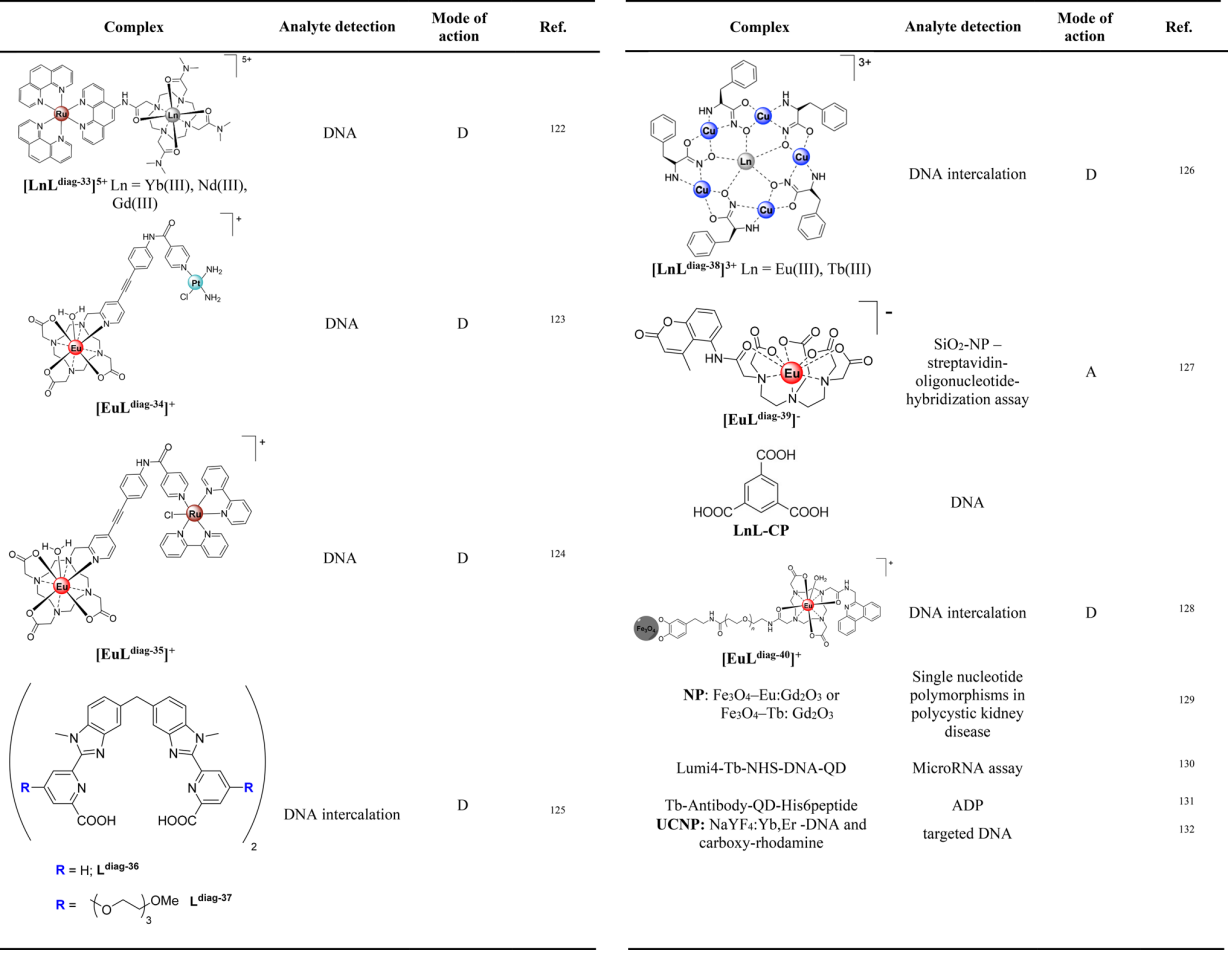
Lanthanide-Nucleic Acid Systems for Detection and Assays

#### 2.2.1 Lanthanide Sensitization upon Coordination to Nucleic Acids

Sensitization of luminescence by nucleobases was identified early on as a sensitive method for *in vitro* detection of nucleic acids. Most studies are based on detection of the green Tb(III) luminescence signal enhancement in the presence of single strand oligonucleotide sequences.^61−69^ An early report of enhancement of Tb(III) luminescence was shown for the binding of Tb(III) to a four-stranded G-quadruplex **[TbL**^**diag-1**^**]** by Chatterji et al.^70^ The selective enhancement of Tb(III) by single stranded DNA vs duplex DNA has been used in sensing schemes for aptamers^71^ or for detection based on structural conversion of DNA.^72^

Detection of Eu(III) luminescence lifetime permits the time-resolved detection of nucleic acids and determination of the number of coordinated water molecules.^73^ In these studies, it was identified that single strand nucleotides show sensitization of the lanthanide emission but not duplex DNA.^67^ This feature has been used for quantification of single strand content of DNA.^74^ It was found that induced conformational changes of poly(dG-dC) are influenced by Tb(III) binding and that one nucleotide enhances the Tb(III) signal more, based on the distance of other ones not being efficient for energy transfer.^75^

**Figure 7:**
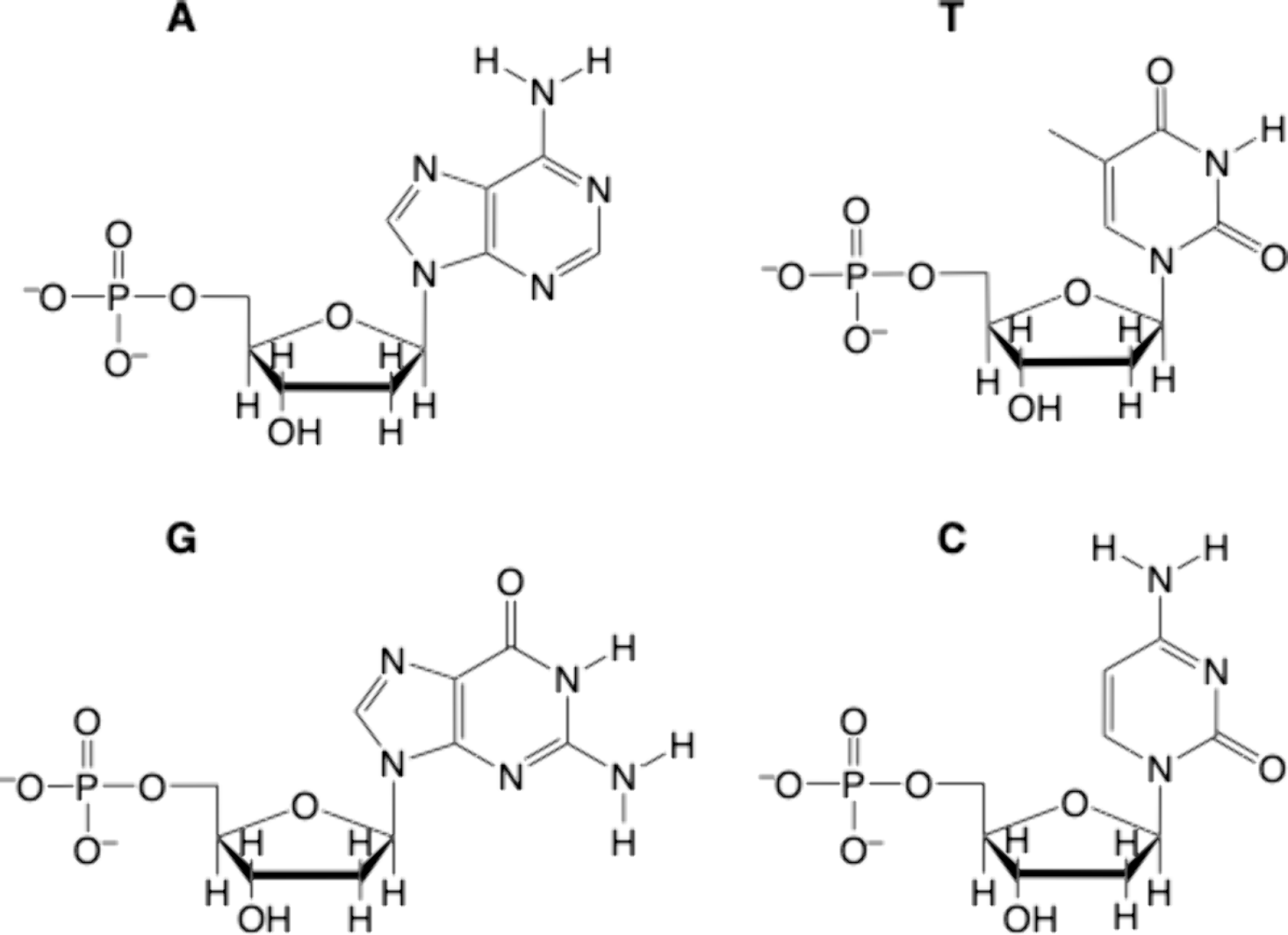
DNA base pairs.

An examination of the sensitization of Tb(III) emission by nucleobases showed that cytosine C only leads to an enhancement of the Tb(III) signal with guanine G on a small enhancement.^76^ The difference in enhancement between the bases is attributed to the binding of the lanthanide to electron rich donors in C (O2 and N3) and G (O6 and N7), but the difference in enhancement is attributed to the difference in quantum yield formation and binding stability or kinetics. However, examination of the sensitization efficiency of the deoxymonophosphate of the nucleobases, dCMP, dGMP, dAMP, and dTMP revealed that dGMP leads to the highest enhancement. This is in agreement with previous studies^66,67^ and studies that also show that GMP > GDP > GTP.^73^ This supports the claims that the phosphate group aids in the binding of the lanthanide to be closer to the sensitizer. In the case of dCMP, it is postulated that the base pair is far from the nucleobase for efficient energy transfer to take place.^76^ Ancillary ligands such as 1,10-phenanthroline^77^ or tetracycline have been suggested to the nucleotide–lanthanide complexation in order to enhance the sensitivity of detection signal of the DNA in the presence of yeast RNA.^78^ This differentiation of sensitization has been used in detection of mismatches of single DNA strands. While duplex strands do not enhance Tb(III) emission, mismatches can be detected, with the largest enhancement observed for the GG mismatch, followed by CA, GA, and CC mismatches.^76^ DNA base pairs are shown in Figure 7.

The binding of nucleotides to lanthanides **[LnL**^**diag-2**^**]**(79) and how it influences conformational transitions of DNA^69^ has been further analyzed with optical spectroscopic techniques^80^ but also NMR, infrared spectroscopy,^81^ and isothermal microcalorimetry.^82^

The characteristic sensitization properties have been used in detection schemes of cations such as Ag(I) and Hg(II) and endogenous thiol groups. The Tb(III) complex of guanine/thymine-rich DNA (5′-[G_3_T]_5_-3′) was used for the luminescent detection of biothiols by the selective turning the Tb(III) signal off/on based on the presence of Hg(II) which quenches the Tb(III) signal in the absence of any thiols. The capture of Hg(II) by endogenous thiols, due to its high affinity, leads to reduced quenching of the Tb(III) luminescence which is bound to a guanine/thymine-rich DNA (5′-[G_3_T]_5_-3′). The approach was also used in detection of sequence-specific DNA as a label-free probe^71^ and demonstrates the wide applicability of time-resolved assays based on Tb(III) luminescence. In another approach, the binding of Tb(III) to G-quadruplex was employed as a probe to the detection of cysteine.^83^ In the presence of Ag(I), the G-quadruplex complex with Tb(III) is disrupted due to affinity of Ag(I) to guanine and the Tb(III) is enhanced, which is attributed to more efficient energy transfer to Tb(III). Upon addition of cysteine, the G4 quadruplex rearranges to bind to Tb(III) due to the high affinity of Ag(I) to cysteine and the Tb(III) luminescence is quenched. The assay reaches a limit of detection of 20 nM with good reproducibility. The sensitization of Tb(III) emission by DNA oligonucleotides has been introduced as a tool for detection of metal ions via time-gated luminescence and luminescence lifetime sensing based on the different synergies of metal binding properties with DNA.^84^ The strategy allows pattern sensing for discriminating metal species, including alkali-metal ions, alkaline-earth-metal ions, transition/post-transition metal ions, and lanthanide ions. The same group also demonstrated characterization of Eu(III) with ssDNA as label free probes for discriminating metal ions based on time-resolved luminescence assays.^85^

It has been shown that the interactions of adenosine and guanine monophosphate nucleotides lead to higher oligomers and luminescent supramolecular networks.^86^ The luminescent lanthanide networks have emerged as attractive materials^87^ for imaging^86,88,89^ but also in the detection of nucleic acids by adsorption of complementary sequences with sensitivity to 0.9 nM target DNA.^90^ The formation of luminescent Eu(III) and Tb(III) microsized fibers as new functional materials has been shown based on coordination of lanthanides with thymidine a ratio of 3:1 of thymidine to lanthanide.^91^ Hydrogels based on nucleosides have been reported for detection of pH, temperature, and metal ions.^92^ Nanoparticles based on lanthanide coordination polymers of guanosine 5′-monophosphate **[LnL**^**diag-3**^**]-NP** have been prepared with luminol for the detection of tetracycline antibiotic, demonstrating the applicability of detection in paper for point of care devices with smart phone detection.^93^

#### 2.2.2 DNA Assays Based on Chelated Lanthanides

**Figure 8:**
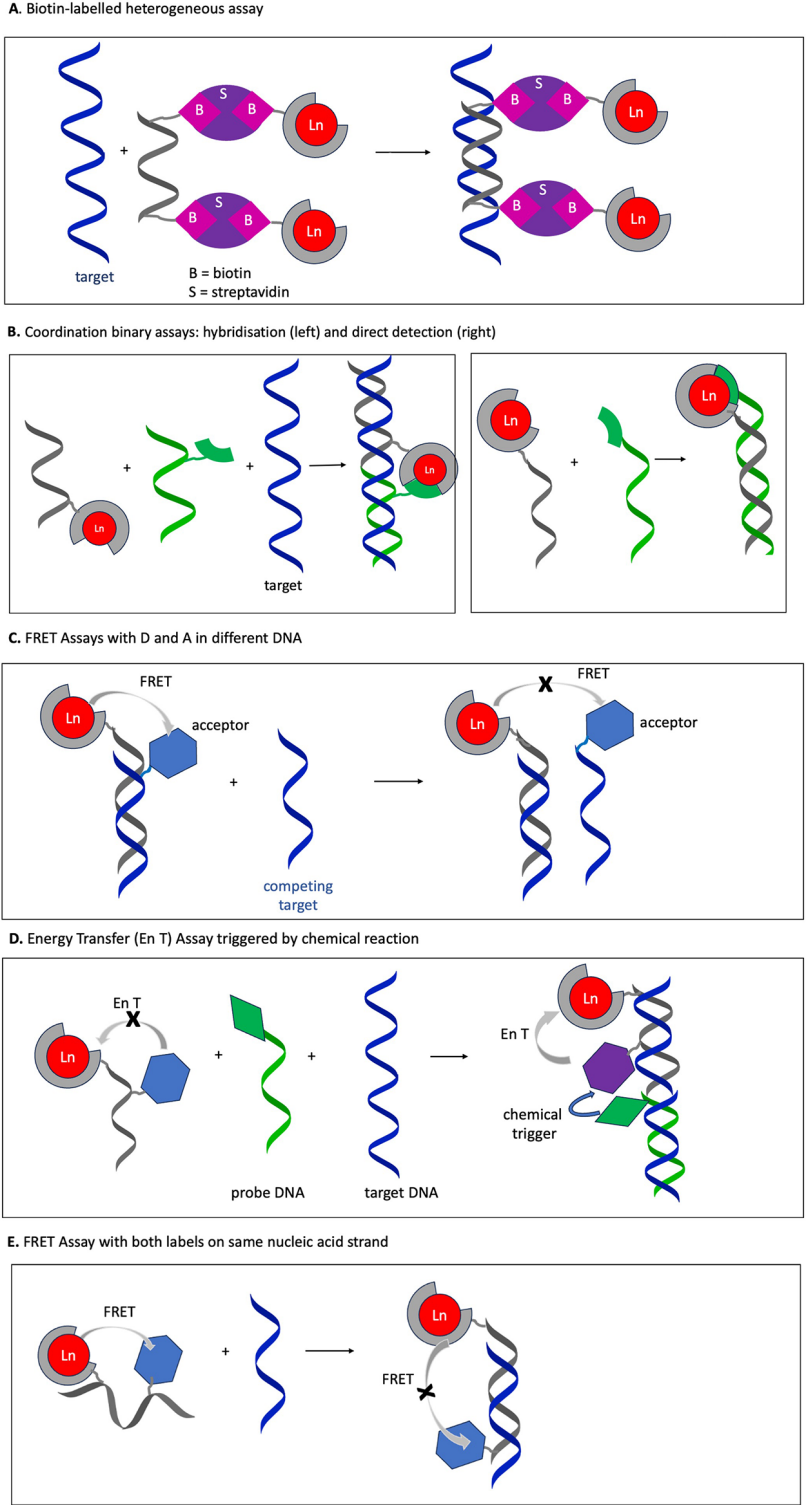
Illustration of strategies in nucleic acid assays using Ln-complexes as probes.

Chelated lanthanides have been extensively used as probes in nucleic acid assays based on different strategies, summarized in Figure 8. These assays are based on appended lanthanide complexes for recognition of nucleic acid strands using heterogeneous platforms (Figure 8A) or homogeneous assays (Figure 8B–E). The latter have the advantage of fewer washing steps and direct detection. Some of the proposed assays have used the lanthanide coordination chemistry (Figure 8B) to bring antenna moieties in close proximity with the chelated lanthanide to achieve nucleic acid recognition. The rest of the approaches involve energy transfer, which is mainly based on resonance mechanism (FRET) to the distance of the donor–acceptor pair (Figure 8D,E).

The development of lanthanide chelates as labels was successfully introduced to DNA detection by labeling biotin in hybridization-type assays (Figure 8A).^133,134^ Diamandis et al.^133^ introduced time-resolved luminescence detection using an established assay based on streptavidin–biotin.^135^ The group had initially introduced 4,7-bis(chlorosulfophenyl)-1,10-phenanthroline-2,9-dicarboxylic acid as a label for biotin to form a luminescent complex with **[EuL**^**diag-4**^**]** and subsequently detect hormones by the biotin–streptavidin recognition in solid phase immunoassays.^94^ The Eu-biotin label was introduced successfully in DNA hybridization assays^133^ to detect target nucleic acids in the region of 10 pg by their immobilization in nitrocellulose membrane.^134,136^ The interaction of nucleic acids with the europium complex of bathophenanthroline **[EuL**^**diag-5**^**]** has been used for the detection of nucleic acids immobilized to membranes such as nitrocellulose or polyvinyldifluoride.^95^ A Eu-DTPA has been used recently in a biotin–streptavidin assay with DELFIA technology to quantify DNA adducts with anthracycline or anthraquinone.^137^

An approach for homogeneous assays relied on the coordination properties of a lanthanide complex attached to a nucleic acid. The assays had different approaches based on hybridization and target detection, and they are also mentioned as binary assays (Table 2).^138^ Two DNA strands complementary to the target DNA were chosen so that the energy donor (sensitizer) and the acceptor the Ln were in close proximity for coordination and hence sensitization of the Ln emission (Figure 8B). This principle relies to a lanthanide chelate with weak or no emission signal and empty coordination sites for the sensitizer to coordinate and come to close proximity for the energy transfer. An early report of this approach employed a Tb(III) DTPA-derivative covalently attached to a DNA strand and a salicylate sensitizer attached to another DNA strand **[TbL**^**diag-6**^**]**.^96^ Nanogram limits of detection of the target DNA were reached, although it was envisaged that amplification of the labeled DNA is required for higher sensitivity of detection.

**Table 2:**
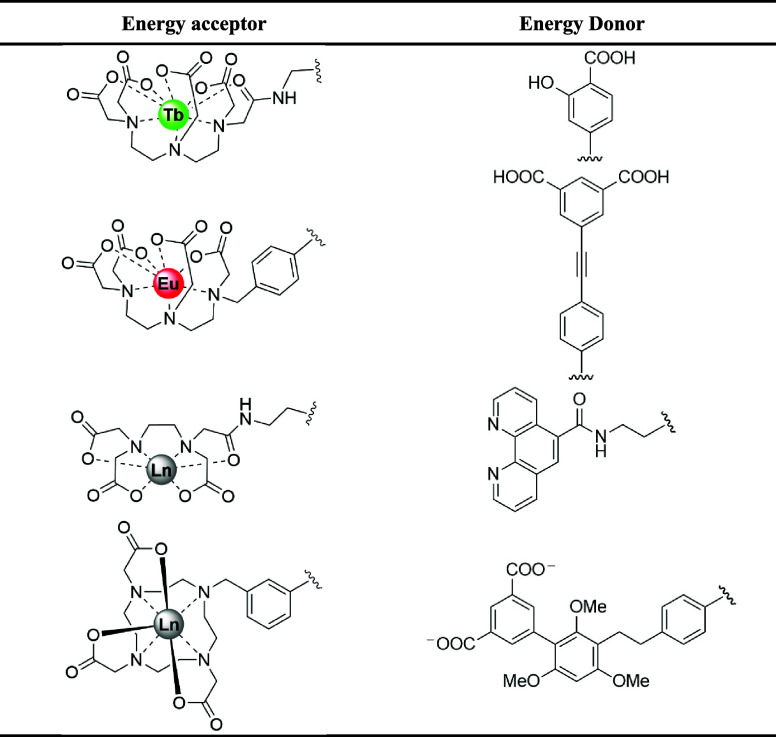
Selected Combinations Used in Coordination Assays^139^

This coordination strategy assay (Figure 8B) has been used with other ligand/sensitizer combinations in hybridization assays, employing aminopolycarboxylate chelating groups (based on EDTA/DTPA) or DOTA macrocyclic complexes in combination with sensitizers terpyridine or dppz or phen ligands (Table 3) as sensitizers attached to oligonucleotides.^138−140^ Single nucleotide polymorphism in a (thiopurine *S*-methyltransferase) gene have also been successfully detected.^141,142^ To enhance the assays to lower limits of detection, a polymerase chain reaction assay was developed with Eu(III) and Tb(III) DOTA complexes.^143^ The assay was developed for homogeneous *Chlamydia trachomatis* bacteria in urine with high accuracies.

**Table 3:**
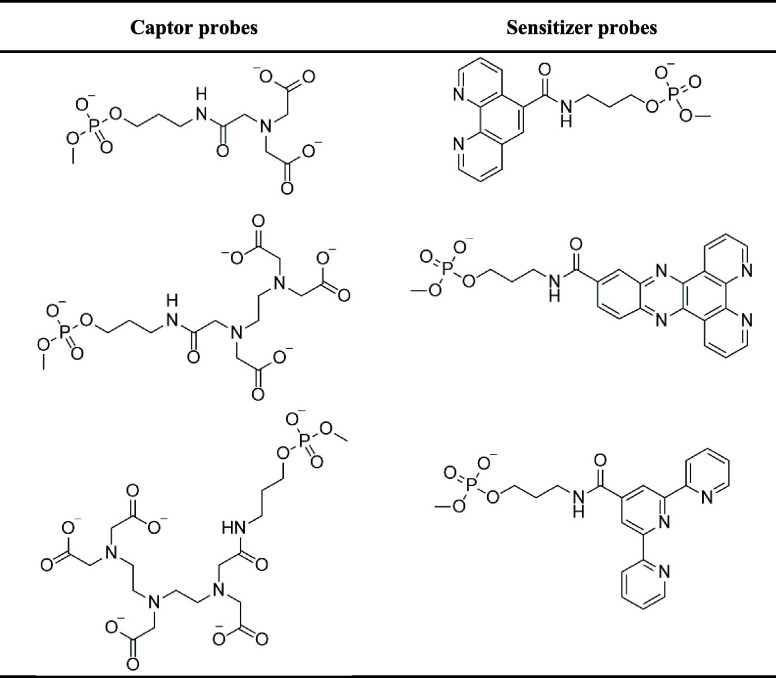
Captor and Sensitizer Chelator Used in Assays^138^

Sammes et al. introduced a new approach to assays with an intercalator for DNA recognition which led to luminescence assays. Early studies were inspired by the sensitization of the Eu(III) luminescence signal by 1,10-phenanthroline-2,9-dicarboxylic acid.^144^ This was modified with a pendant group cationic phenanthridinium group **[EuL**^**diag-7**^**]**^+^, which is known intercalator for DNA. The 1:1 binding of the sensitizer to europium was regulated by a cooperative effect with EDTA or a dicarboxylic acid derivative of diaza-15crown5.^97^ This approach was used in a homogeneous DNA assay, with a probe-DNA linked to an EDTA, which captured Eu(III) and the sensitizer–intercalator unit. The binding only occurs when the probe DNA forms a double helix with the target DNA, hence providing a sensitization assay.^145^

Selvin et al. developed an asymmetric bisamide derivative of diethylene triamino pentaacetic acid with a carbostyril group as a sensitizer and a link to an oligonucleotide **[LnL**^**diag-8**^**]**.^98,99^ The carbostyril group acts as a sensitizer for Eu(III) and Tb(III) emission. In a further development of a hybridization assay, the Eu(III) and Tb(III) chelates act as donors to Cy5 or fluorescein acceptors for Eu(III) and Tb(III), respectively (Figure 8C).^146^ The FRET process involving lanthanide as a donor showed distinct advantages over conventional organic dyes in detection, mainly due to the long lifetime of the donor which is easier and more accurate to monitor, and it is not affected by orientation factors. Additionally, the large Stokes shift with the acceptor eliminated any interference in detection of the acceptor. The distance at which 50% of FRET occurs was reported to be 65 Å. The Tb(III) complexes of carbostyril–DTPA were shown to efficiently detect small amounts of quadruplex DNA based on energy transfer to a 22mer human telomeric DNA.^147^

In a new approach, the energy transfer between a donor and a lanthanide in the same nucleotide strand is triggered by a chemical reaction due to the proximity of a DNA probe molecule carrying a reactive/reducing probe.^148^ The latter comes in close proximity with the nucleic acid carrying the energy donor and the lanthanide complex only in the presence of the target DNA molecule (Figure 8D). The chemical reaction changes the donor to the lanthanide to a sensitizer which can turn on the lanthanide signal.^149^

Mathis et al. introduced the thermodynamically and kinetically stable Eu-cryptate attached either to streptavidin or biotin in hybridization assays on nitrocellulose support which delivered attomolar detection limits for target DNA based on europium red luminescence signal **[EuL**^**diag-9**^**]**.^100,134^ The Eu(III) cryptate label was also incorporated in a time-resolved assay for detection human papillomavirus type 16 DNA with modified hybrids following polymerase chain reaction **[EuL**^**diag-10**^**]**^3+^.^101^ The assay enabled solid phase detection of the desired nuclei acid sequence in clinical smears by time-resolved spectroscopy. An evaluation of the lanthanide cryptate label against the ^32^P radiolabeling assay showed similar analytical sensitivity with the homogeneous assays presenting better efficiency and speed.^100^ Assays based on FRET between the Eu cryptate and an acceptor were evaluated by using directly labeled nucleotides and labels introduced via biotin–streptavidin in the approach for diagnosis of single nucleotide polymorphism.^150^ The Eu-cryptate was used to label RNA transcripts which were also modified with biotin for recognition with streptavidin labeled with allophycocyanin as acceptor as a FRET pair.^151^ Several hybridization assays have been developed, demonstrating the potential of the lanthanide cryptates in diagnostic assays (Figure 8C and E).^152^

**Figure 9:**
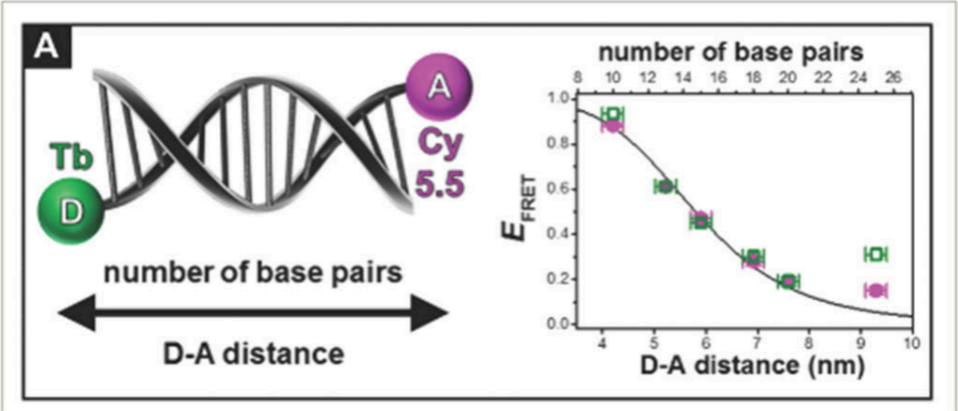
Donor–acceptor distance mediated by DNA strands. Reproduced with permission from ref (154). Copyright 2017 John Wiley and Sons.

A multidetection approach involved a Tb(III) cryptate in different types of FRET hybridization assays in combination with a fluorescent acceptor attached on the same probe single stranded nucleic acid (Figure 8E). The assay provided monitoring both Tb(III) donor and fluorescent acceptor lifetimes for multiple detection schemes of hybridized targets.^153^ The tuning of distance between a Tb(III) cryptate donor and an acceptor in two DNA strands (Figure 9) was shown by Hildebrant et al. using time gated luminescence.^154^ The study demonstrated FRET with different acceptor dyes in detection of different DNA assays.

A highly luminescent lanthanide complex based on terpyridine was developed for labeling of both nucleic acids and antibodies **[EuL**^**diag-11**^**]**^**–**^.^102^ The terpyridine is an excellent sensitizer and a pocket for Eu(III) with the NCH_2_COO^–^ giving good stabilization. The isothiocyanate derivative was successfully attached to a 20-mer oligonucleotide allowing two lanthanide complexes per oligonucleotide, leading to sensitive detection of the complementary DNA strand (1.5 × 10^–16^ mol) by time-resolved luminescence. Matsumoto et al. developed and evaluated a Eu-terpyridine label **[EuL**^**diag-12**^**]**^**–**^ for nucleic acid detection.^103^ The label was used to detect single nucleotide mutation with peptide nucleic acids by time-resolved luminescence^155^ and in solid phase assays using a DNA ligase to bring the nucleotides together for recognition.^156^

Hemilla et al. evaluated a terpyridine-aminocarboxylate label **[EuL**^**diag-13**^**]**^**–**^ for energy transfer to Alexa Fluor dyes as acceptors in a homogeneous assay for targeting a DNA sequence for cystic fibrosis.^104^ The targeting was based on a hybridization assay with the donor and acceptor labels attached to DNA sequences. A detection limit of 0.8 pM was reported which shows high sensitivity of the assay. While the acceptor absorption did not overlap with the Eu emission for conventional FRET, it was the emission of the acceptor that was monitored for its enhancement and longer lifetime which was attributed to a nonenergy transfer from higher Eu(III) states such as ^5^D_2_ and ^5^D_1_. A derivative of the same label was attached to four different acyclic nucleoside triphosphates to form an assay with four complexes for identification of two mutations in cystic fibrosis. The detection worked for synthetic DNA targets using Eu(III), Tb(III), Sm(III), Dy(III) but relying on detection of the lanthanide DELFIA technology.^157^ It was identified that blood samples however would need PCR amplification for successful analysis. The principle of the homogeneous label assay with the terpyridine label was applied by the Hemilla group in a dual DNA assay for detection of celiac disease-related target oligonucleotides demonstrating the potential in detection of single-nucleotide polymorphisms.^103,158^

#### 2.2.3 Monometallic DNA Recognition

In the medicinal bioinorganic chemistry area, metal complex interactions with DNA are dominated by transition metal architectures for their anticancer activity. However, there have been examples of lanthanide complexes for DNA assays and potential anticancer activity^159^ Lanthanides coordinated to mainly bidentate ligands with potential intercalative properties such as 1,10-phenanthroline and derivatives or quinoline, hydroxy-quinolines, and flavonoids have shown DNA interactions and cellular cytotoxicity.^159−167^ In most cases, the interactions with DNA are considered intercalative, although other modes or binding are also present. A bis-phenanthroline four-coordinate ligand was reported to lead to lanthanide complexes which bind DNA partly via intercalative binding.^168^

Cationic lanthanide complexes based on DOTA framework **[LnL**^**diag-14**^**]**^**4+**^ and **[EuL^diag-15^]^4+^** were designed with a pendant *N*-methyl phenanthridinium, and their interaction with [(CG)_6_]_2_ and [(AT)_6_]_2_ was studied.^105^ The luminescence of the pendant group is quenched as it is usually observed for organic intercalators attributed to a charge transfer interaction with the bases of the nucleic acid. However, the luminescence lifetime of the europium center remained unchanged, although the luminescence intensity was quenched upon nucleic acid interaction. Circular dichroism studies revealed weaker interaction of one of the enantiomers with [(AT)_6_]_2_ possibly due to the different mode of binding via the minor groove. Further studies with calf thymus DNA, poly(dGdC), and supercoiled plasmid DNA supported their intercalative interaction together with an interaction with the nucleobase phosphate group to the hydrated Eu center.^169^

Tetraazatriphenylenes have also been used as a pendant intercalator for DNA recognition.^106^ The DOTA-based ligand provides a ligand framework for isolation of enantiomerically pure positively charged lanthanide complexes, studied for their interaction with polynucleotides **[LnL**^**diag-16**^**]**^**3+**^ and **[LnL**^**diag-17**^**]**^**3+**^. Interestingly, interaction with poly(dGdC) led to more quenching of europium emission than the interaction of poly(dAdT). Spectroscopic studies suggested that the interaction with poly(dAdT), for both l- and d-isomers is predominantly intercalative binding. A lanthanide DOTA complex was prepared with two sensitizers coumarin and rhodamine linked to the metal complex **[LnL**^**diag-18**^**]**^**+**^. Energy transfer from coumarin to rhodamine is cascaded to the near-infrared emitting lanthanide.^107^ Upon DNA addition, the first energy transfer step is disrupted, enabling a method for DNA sensing in near-infrared. A modification of a DOTA complex with bis-aminobenzimidine units **[LnL**^**diag-19**^**]** showed A/T-selective binding driven by the bis-aminobenzimidine recognition units and sensitization for specific double stranded A/T sequences.^108^

The Vazquez group also demonstrated the functionalization of a 20-mer peptide with a Tb-DOTA complex and a tryptophan antenna **[TbL**^**diag-20**^**]**.^109^ Upon binding of the peptide to its target RNA hairpin, an increase of the emission takes place due to the conformational change, which allows proximity of the tryptophan to Tb(III) for the sensitization to take place. The group demonstrated the sensitization in biological media, which shows the potential of detection of other structured RNA targets. A new class of DOTA-based macrocycles functionalized with guanine residues has been proposed as a template to synthesize G-quartet, and the corresponding Tb(III) complexes have been shown to interact with G-quadruplexes as promising agents for cellular probes.^72^

Delangle et al. reported the design of a lanthanide binding peptide for the detection of double stranded DNA by Eu(III) luminescence **[EuL**^**diag-21**^**]**.^110^ The peptide bears a proflavine moiety which acts as sensitizer for Eu(III) emission and a DNA intercalating group. Addition of DNA leads to quenching of the Eu-centered luminescence. Binding studies of lanthanide tris-diketonates, with phenanthroline as ancillary ligand to DNA, were recently reported, **[LnL**^**diag-22**^**]**, accompanied by cellular uptake imaging studies revealing mainly cytosol and some nuclear uptake.^111^ These complexes are neutral, and there is not significant evidence of intercalation to ctDNA. Another family of complexes based on deprotonated 5-hydroxyflavone and 1,10-phenanthroline **[LnL**^**diag-23**^**]** shows cytotoxicity in cancer cells, although the nature of the lanthanide ion does not seem to have an effect on cytotoxicity.^112^ Studies show significant DNA interaction, and docking studies suggest DNA intercalation.

A pair of chiral complexes of a nonaaza macrocyclic amine with Yb(III) **[YbL**^**diag-24**^**]^3+^**(113,170) have been shown to enantioselectively bind to B-form DNA.^114^ The P-enantiomer stabilizes both poly(dG-dC)_2_ and poly(dA-dT)_2_, while the M-enantiomer stabilizes poly(dA-dT)_2_ but destabilizes poly(dG-dC)_2_. A possible mechanism postulated from experiments is binding to the minor groove of DNA.

#### 2.2.4 Polymetallic Lanthanide Complex Recognition

DNA topology recognition is mainly dominated by transition metal complexes with defined structure control rather than lanthanide architectures which can be limited by kinetic and thermodynamic instability depending on the choice of ligands. Luminescent polymetallic lanthanide structures most commonly are constructed by combinations of lanthanides and transition metals for their interactions to nucleic acids.

A Pd-porphyrin was covalently attached to a chiral, cationic lanthanide DOTA derivative **[YbL**^**diag-25**^**]**^**3+**^. The Pd-porphyrin acts as a sensitizer of Nd(III) and Yb(III) near-infrared luminescence in the absence of oxygen and in the presence of oligonucleotide.^115^ Interestingly, the binding to nucleic acid inhibits the quenching of the porphyrin’s triplet state by oxygen.

**Figure 10:**
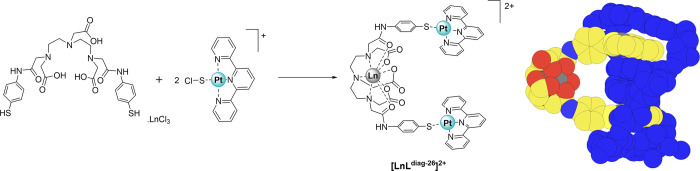
Self-assembly of **Ln-Pt_2_** metallohairpins. Adapted and reproduced with permission from ref (116) . Copyright 2003 American Chemical Society.

An approach to rigid hairpin-shaped bis-intercalators was introduced with heterometallic lanthanide complexes based on the **Ln-Pt_2_** motif established with [Ln-L**^diag-26^]**^**2+**^(Figure 10).^116^ The versatile motif can be self-assembled based on the selective coordination preference of the hard lanthanide ion vs the soft platinum center. Bis-intercalators are known to increase the detection limit for DNA.^171^

The lanthanide binding site is based on a bisamide derivative of diethylene triamine pentaacetic acid (DTPA) which has two appended Pt(II)-terpyridine units **[NdL**^**diag-26**^**]**^**2+**^. It forms a neutral lanthanide remote center with positive charges on the platinum intercalating unit. Upon interaction with calf-thymus DNA, considerable stiffening of the DNA is observed by linear dichroism studies, supporting the bis-intercalating mode of the trinuclear complex. The luminescence of the near-infrared emission Nd(III) encapsulated in the aminocarboxylate ligand is not affected by binding to DNA. In this system, there is an LLCT transition between the ligands of the Pt(II) center which interferes with any sensitization process. A modification of the coordinating ligand to the Pt-terpyridine unit to ethynyl led to a shift of the charge transfer band and the complex showed photosensitization of Eu(III) luminescence signal upon interaction with ct-DNA **[EuL**^**diag-27**^**]**^**2+**^.^117^ Modification of the Pt-unit to a *cis*-[Pt(NH_3_)_2_Cl]^+^, which has known anticancer activity, demonstrated the potential of the system as a theranostic when gadolinium was used for imaging **[LnL**^**diag-28**^**]**^**2+**^.^118^

Quinoline derivatives of the DTPA(bisamide) ligand **[LnL**^**diag-29**^**]**^+^ and **[LnL**^**diag-30**^**]** have shown promising results for sensitization of red emission.^119^ A derivative of quinoline was shown enhanced luminescence upon interacting with ctDNA and cleavage of DNA, which indicated the potential of the complex as a phototoxic agent.^172^ A combination of one of the quinoline DTPA(bisamide) ligands with the cytotoxic *cis*-PtCl_2_(DMSO) moiety **[EuL**^**diag-31**^**]** led to luminescent Eu(III) complexes, which also have cytotoxic effects due to their covalent attachment to DNA.^120^

Patra et al. reported a DTPA complex bearing two quinoline units, each of which was coordinated to cisplatin **[LnL**^**diag-32**^**]**^**2+**^.^121^ These hairpin shaped complexes were found to cross-link to DNA in the nucleolus. Electronic absorption titration shows significant hypochromism due to strong binding propensity of these complexes through a LnPt_2_–DNA adduct formation and possible stacking interaction through two planar quinoline–Pt moieties with DNA. Binding and circular dichroism studies suggest structural deformation and unwinding of ds-DNA helices.^121^

Gunnlaugsson et al. developed a family of *d–f* bimetallic Ln-Ru complexes based on a DOTA amide derivative with a ruthenium tris-phenanthroline unit **[LnL**^**diag-33**^**]**^**5+**^.^119,122^ The overall complex has a 5+ charge, and upon interaction to DNA, the Yb(III) signal is turned OFF, whereas the ruthenium signal is still ON. In the Nd(III) case, the Nd(III) signal is unaffected by the DNA interaction.

Kwong, Wong et al. reported a hydrophilic Eu(III) DO3A complex bearing a rigid π-conjugating antenna with an isonicotinamide ligand which is coordinated to cisplatin **[EuL**^**diag-34**^**]**^**+**^.^123^ The excitation energy absorbed by the antenna is channeled via intersystem charge transfer to Pt(II); thereby, weak Eu(III) emission is detected. Upon photodissociation of the cisplatin from **[EuL**^**diag-34**^**]**^**+**^, energy transfer from the antenna’s triplet excited state to the first excited state of Eu(III) occurs with significant Eu(III) emission in an off–on manner. This design provides a real-time trace able delivery vehicle for cisplatin to its *in vitro* target. From circular dichroism spectroscopy, **[EuL**^**diag-34**^**]**^**+**^ was found to bind to DNA and unwind its helix. This DNA cleavage photocatatylic activity was less significant in cisplatin, suggesting a distinct DNA damaging mechanism for **[EuL**^**diag-34**^**]**^**+**^. From dark cytotoxicity studies in HeLa and A549 cell lines, **[EuL**^**diag-34**^**]**^**+**^ exhibits lower dark cytotoxicity in comparison to cisplatin and the complex without cisplatin.^123^

A *cis*-[Ru(Cl)(bpy)_2_]^+^ was also found to photodissociate from **[EuL**^**diag-35**^**]**^**+**^, leading to enhancement of Eu(III) luminescence.^124^ A delayed shift in the electrophoresis was observed in **[EuL**^**diag-35**^**]**^**+**^, suggesting the release of the Ru(II) analogue which causes DNA damage upon photoirradiation. Cytotoxicity studies in HeLa cell line suggest significant light-induced cytotoxicty for **[EuL**^**diag-35**^**]**^**+**^ in comparison with **[EuL**^**diag-34**^**]**^**+**^.^124^

A displacement assay of the acridine orange intercalator was established with the neutral binuclear helicates **[Eu**_**2**_**L**^**diag-36**^**]** and **[Eu**_**2**_**L**^**diag-37**^**]**^+^ by Bünzli et al.^125^ The Eu(III) luminescence is quenched in the presence of free acridine orange. However, in the presence of DNA, it is restored based on the intercalator binding to DNA. The assay established the sensitivity of metallo-bis intercalators in detection of DNA.

Dinuclear amino acid complexes of Tb(III) have been reported to bind to human telomeric G-quadruplex and i-motif DNA structures with characteristic luminescence enhancement.^173^ Lanthanide 15–metallacrown-5 complexes based on Eu(III) and Tb(III) with phenylalanine hydroxamic acid and Cu(II) have been reported to interact with G-quadruplex DNA, leading to luminescence quenching of the lanthanide signal.^126^ An assay to evaluate the G-quadruplex binding was proposed.

#### 2.2.5 Lanthanides in Nanoparticles for DNA Detection and Recognition

**Figure 11:**
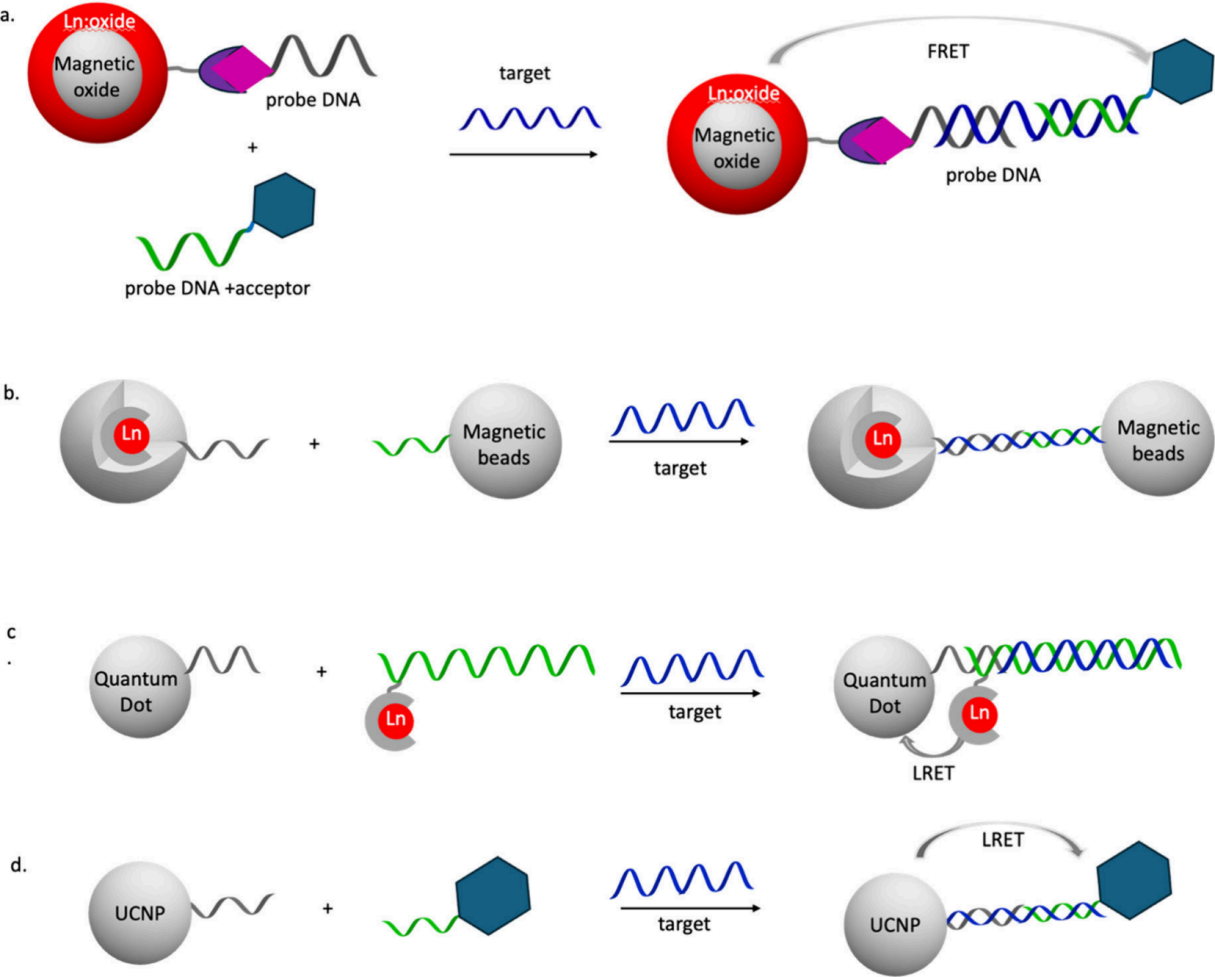
Illustration of strategies involving nanoparticle-based hybridization assays.

Nanoparticles have been attractive in DNA detection due to the versatility of approaches for attachment of nucleotides to the surface of the particle and incorporation of the luminescent center either in the core of the particle or on the surface (Figure 11). Some approached involve magnetic oxide nanoparticles for separation and isolation of the target nucleotides (Figure 11a, b); host networks like silica particles have been involved to encapsulate the chelated lanthanide in their network, allowing recognition of the nucleotides on the surface of the nanoparticles; nanoparticle designs based on quantum dots (QD) and upconverting nanoparticles (UCNP) involve luminescence resonance energy transfer mechanisms for detection based on the lanthanide luminescence signal as the donor (Figure 11c, d).

An early approach in introducing lanthanide luminescence is based on inclusion of the lanthanide complex, **[EuL**^**diag-38**^**]^3+^**, into the silica particle framework using a microemulsion technique.^127^ The oligonucleotide was attached on the surface of the particles using a streptavidin–biotin conjugation. A demonstration of using these particles for DNA detection was established using a DNA sandwich assay with magnetic beads labeled with an oligonucleotide. The response showed higher sensitivity than a commercial organic probe without nanoparticles.

A luminescent Eu-DOTA derivative bearing a benzoquinoline potential intercalator to DNA was attached to iron oxide particles via a polyethylene glycol linker **[EuL**^**diag-39**^**]**^–^, **[EuL**^**diag-40**^**]**^**+**^. The europium emission is >95% quenched upon addition of ctDNA.^128^ The luminescence effect is compared to Ru-complexes with dppz (dipyrido[3,2-a:2′,3′-c]phenazine) intercalators attached to particles which demonstrate a turn-on of their luminescence signal.

Europium luminescent coordination polymers, Ln-CP with L = 1,3,5-benzenetricarboxylic acid bridging ligands, which adopt rod structures, are shown to adsorb single stranded DNA labeled with 6-carboxyfluorescein and quench its fluorescence. Upon addition of DNA’s complementary strand, the signal of 6-carboxyfluorescein is restored, for which the europium signal remains unchanged, providing a colorimetric sensor for DNA.^174^

Magnetic nanoparticles or beads have been combined with luminescent lanthanides in different approaches for DNA assays. Magnetic beads provide the attraction of separating the hybridized DNA to eliminate any background signals. In one approach, core–shell nanoparticles Fe_3_O_4_–Eu:Gd_2_O_3_ or Fe_3_O_4_–Tb:Gd_2_O_3_ were used in a hybridization assay for detection of single nucleotide polymorphisms in polycystic kidney disease.^129^ The nanoparticles were functionalized with neutravidin so that a biotinylated DNA probe can be attached. Another DNA probe with the acceptor Alexa-fluor dyes was also used in the hybridization assay so that FRET will take place upon presence of the target. This solution approach allowed detection of two single nucleotide polymorphisms with Eu(III) and Tb(III) nanoparticles.

Colloidal semiconductor quantum dots (QD) have been involved in FRET-based homogeneous assays with lanthanides for time gated assays. QD provide potential multiplexing for bioprobes based on their color tunability and the possibility of surface attachment for biomolecular recognition. Streptavidin-labeled Eu(III) or Tb(III) probes were assessed as donors in biotin functionalized QD.^175,176^ A commercially available derivative of terbium cryptate with *N*-hydroxy succinimide, Lumi4-Tb-NHS, has been conjugated to reporter DNA and acted as donor to QD conjugated via biotin to short nuclei acid strands.^130^ An assay has been developed for detection of three different microRNAs when three different QD have been used. The importance of the sizes of QD on the amplification process of nucleic acids has been elucidated, and limit of detection down to 80 fM has been reported.^177^ A number of hybridization assays for nucleic acid detection have been reported based on this technology.^5^

Another approach of the FRET QD assays involved detection of adenosine diphosphate based on Tb-labeled antibody and a QD modified on its surface with an ADP modified His6-peptide. The Tb-labeled antibody selectively recognizes ADP versus ATP. In the presence of free ADP, the antibody does not interact with the QD; hence, the FRET is turned off. The assay allows a limit of detection of 10 nm of ADP.^131^

UCNP) have been attractive probes to take part in FRET or LRET due to the color availability and excitation wavelengths where there is no interference, opening possibilities for optical barcoding assays.^178,179^ Most of the UCNP applications involve imaging and therapeutic effect in tissues, and there are few in assays. An early example demonstrated involvement of UCNP in homogeneous sandwich assays for detection of target DNA.^132^ The UCNP is based on NaYF_4_:Yb,Er functionalized with a probe DNA which is hybridized with a complementary strand tagged with the acceptor, carboxy-rhodamine. Luminescence resonance energy transfer takes place in the presence of target DNA with reduction of the lanthanide luminescence signal. Soukka’s group showed that the assay can be used for multiplex DNA detection by single near-IR excitation.^180^ The approach has also been used with an intercalating dye as acceptor for detection of single-base mismatched target with limit of detection to 20 fmol.^181^ An enzyme exonuclease III was shown to improve detection sensitivity of this assay for human immunodeficiency viral DNA.^182^

#### 2.2.6 Lanthanides in DNAzymes

The selective sensitization of lanthanide luminescence by nucleic acids has been used in DNAzymes for the development of luminescence-based sensing platforms.^183^ Discrimination of lanthanides has been investigated based on an array of five DNAzymes.^184^ A paper-based sensor has been developed to detect heavier lanthanides using UCNP.^185^ Lanthanides are also known to cleave RNA^186−188^ and can play a vital role as cofactors in DNAzymes. The detection of many of the DNAzyme assays for RNA cleaving is based on external fluorescent probes.^189^ However, lanthanide luminescence has been used to elucidate the role of spectroscopically silent metal ions in DNAzymes and evaluate their activity.^190−192^ Terbium luminescence signal and luminescence lifetime have been used to inform about the metal binding site of a specific DNAzyme.^193^ It was shown that Tb(III) is a competitive and reversible inhibitor for the Zn(II) and Pb(II)-dependent DNAzyme activity. The sensitized Tb(III) luminescence was based on its coordination with the phosphate group and the N-7 guanine by Tb(III), which is expected to also influence the geometry of the sugar–phosphate backbone and the stacking interaction between the bases in double-stranded DNA. Sensitization of Tb(III) by a close by guanine was also used in studies to demonstrate the requirement of the presence of two metal cations for catalysis in RNA-cleavage DNAzyme, Ce13d.^87^ The binding trend of a series of lanthanides shows that the lanthanide binding is not the limiting step in nucleic acid catalysis.^194^ The change in terbium luminescence in selective DNAzymes has been used to monitor the binding of Na(I). The studies have revealed that there is a selective aptamer pocket for Na(I), which displaces Tb(III) and reduces its luminescence signal.^195^

#### 2.2.7 Anthrax Assay

A very successful assay in lanthanide sensitization approaches is based on the detection of anthrax bacterial spores. Table 4 highlights complexes used for detecting anthrax bacterial spores.

**Table 4:**
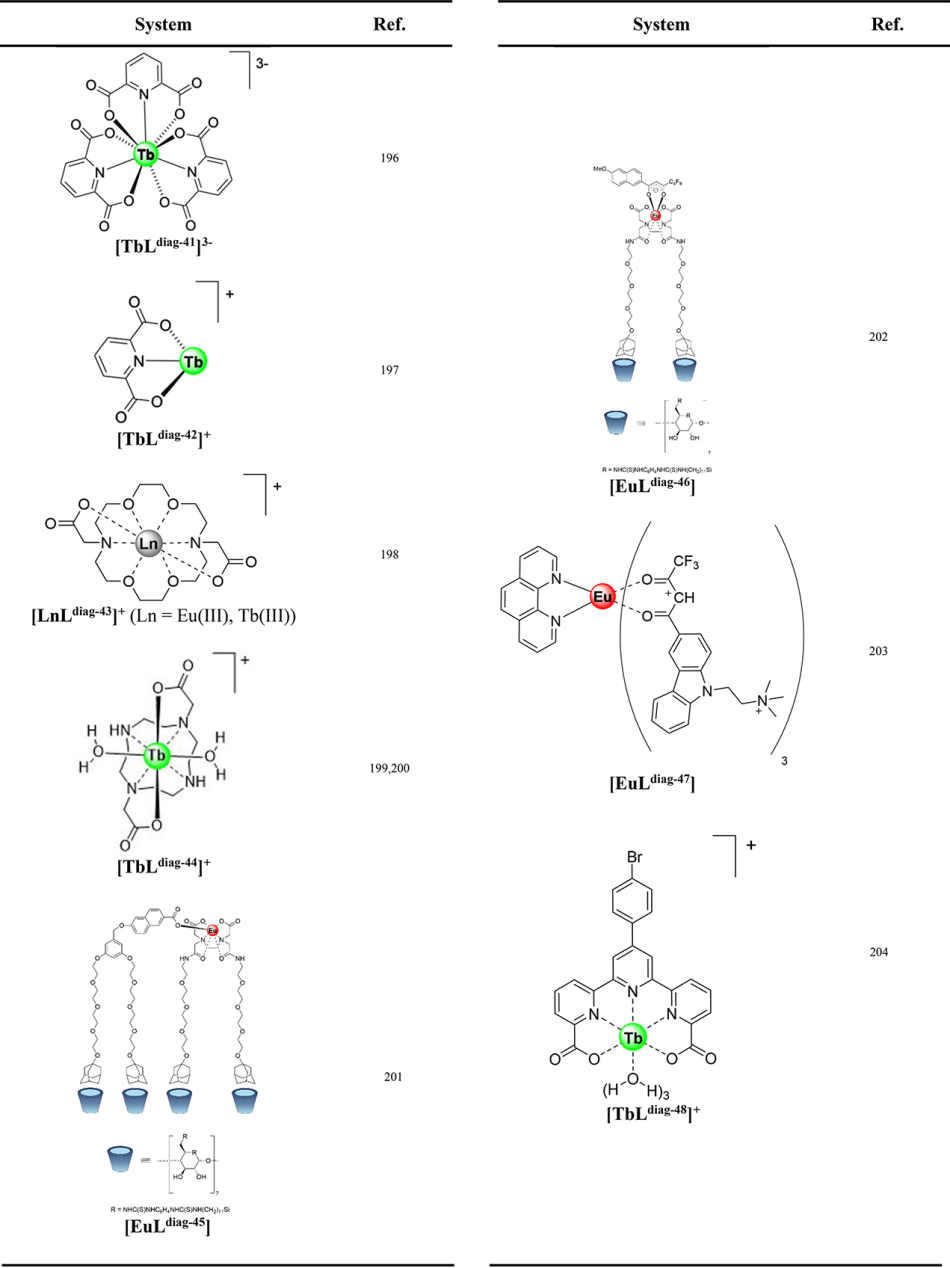
Lanthanide Complexes in Anthrax Assays

Bacterial spores are challenging to detect with common approaches used in microbe detection based on bioluminescence assays.^196^ It was established that dipicolinic acid (pyridine-2,6-dicarboxylic acid) is present in bacterial spores^205,206^ in considerable quantities, 5–15% of total mass in bacterial spores, and its sensitization of Tb(III) upon binding in a 3:1 ratio was initially introduced for Tb(III) detection.^207−209^ The assay for detection of dipicolinic acid was established with 10^4^*Bacillus subtilis* spores per mL detected by monitoring Tb(III) green luminescence **[TbL**^**diag-41**^**]**^**3–**^.^196^ A quantification method for sampling bacterial spores from air and analyzing by Tb(III) detection was established by the Ponce group at Jet Propulsion Laboratories **[LnL**^**diag-42**^**]**^**+**^.^197^ To improve the binding event and interference of phosphate ions, a previously studied approach was used to fill in the lanthanide coordination sphere using a macrocycle in order to eliminate anion binding and improve sensitivity **[LnL**^**diag-43**^**]**^**+**^.^198,210^ The binding of the dipicolinic acid was optimized for macrocyclic terbium complexes reducing interference of calcium ions and mitigating phosphate interference 1000-fold compared to free terbium alone **[LnL**^**diag-44**^**]**^**+**^.^199,200^

A supramolecular surface assembly based on a cyclodextrin printboard was introduced for detection of spores based on ratiometric sensing of Eu(III) and napththalene guest. The energy transfer between naphthalene and Eu(III) guest-chromophores on the surface is disrupted by the binding of the dipicolinic acid to Eu(III) complexed in an EDTA guest **[EuL**^**diag-45**^**]**,^201^ which restores the naphthalene blue emission. The system afforded nanomolar sensitivity of dipicolinic acid detection. An assembly of the Eu(III)-EDTA conjugate and naphthalene β-diketone in a cyclodextrin microchannel surface was used to screen dipicolinic acid and ATP **[EuL**^**diag-46**^**]**.^202^

Multimetallic lanthanide assemblies are attractive for ratiometric studies of dipicolinic acid. These include supramolecular assemblies of Eu(III) complexes **[EuL**^**diag-47**^**]** with sulfonatocalixarenes modified with Tb(III), which led to analysis of 2.2 × 10^4^ spores/mL^203^ but also coordination polymers,^211−213^ nanorings,^214,215^ nanosheets,^216^ and metal–organic frameworks.^217−221^ A Tb(III)-based arylbromo terpyridine complex **[TbL**^**diag-48**^**]^+^**was reported to interact with dipicolinic acid in *Bacillus anthracis* bacterial endospores.^204^ A 600-fold luminescence enhancement upon the addition of dipicolinic acid was observed, with a binding constant of 6.67 × 10^6^ M^–1^. The luminescence response of **[TbL**^**diag-48**^**]** was selective for dipicolinic acid in the presence of various interfering carboxylic acids, amino acids, and several other bioanalytes, and it could quantitatively detect dipicolinic acid up to ∼54 ppb (0.32 mM).^204^

Several nanoparticle approaches have been used for assembly of lanthanides for detection of dipicolinate based on lanthanide signal including fluorescent polyfluorene dots,^222^ polymers for device preparation,^223,224^ silver nanoparticles,^225^ micelles with encapsulated diketonates,^226^ and emissive silicon nanoparticles with a low limit of detection of dipicolinic acid of 5.3 nM.^227^

Colorimetric detection assays have also been introduced with upconverting nanoparticles^228,229^ and silica particles for a paper-based sensor.^230^ A portable dual emission assay method based on paper-microchip and smartphone integrated mini-device has recently been introduced.^231^

### 2.3 Enzyme Monitoring by Lanthanide Complexes

**Figure 12:**
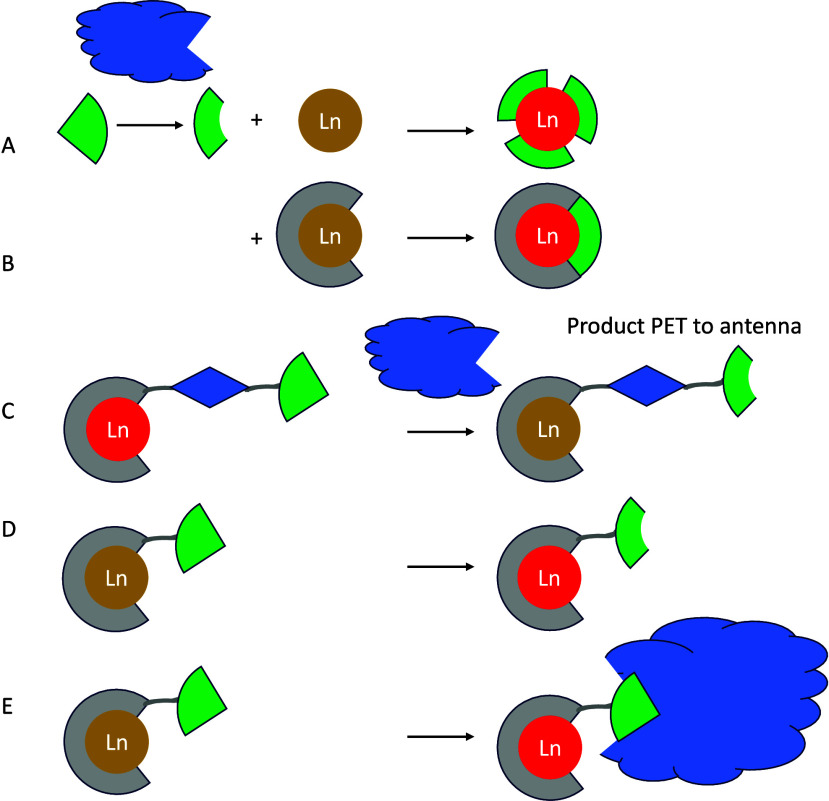
Approaches to using lanthanides for detection of enzymatic activity.

The stability of lanthanide complexes enables them to be used both in biological assays, e.g., high throughput screening, and in certain cases *in vivo*. Separation of signal is enabled by their attractive photophysical properties discussed earlier in this review, particularly their narrow band, long-lived luminescence. This section summarizes approaches to the monitoring of enzyme activity via the free enzyme interacting with components of the assay rather than conjugation of lanthanides to enzymes (Table 5). There have been a number of relevant reviews in the use of lanthanides in enzyme assays.^232−237^ For the purpose of this section, the enzyme assays are classified in terms of how the lanthanide functions within the assay, including binding of an enzyme product, lanthanide chelates with binding pockets, switching of antennas including reactive pendant arms, and reactions with enzyme products (Figure 12). For successful function in biological assays and in particular *in vivo*, there is also the need for sufficient robustness and selectivity to avoid impact on the performance of the assay, other compounds interfering with results, or adverse effects on cell viability *in vivo*.

**Table 5:**
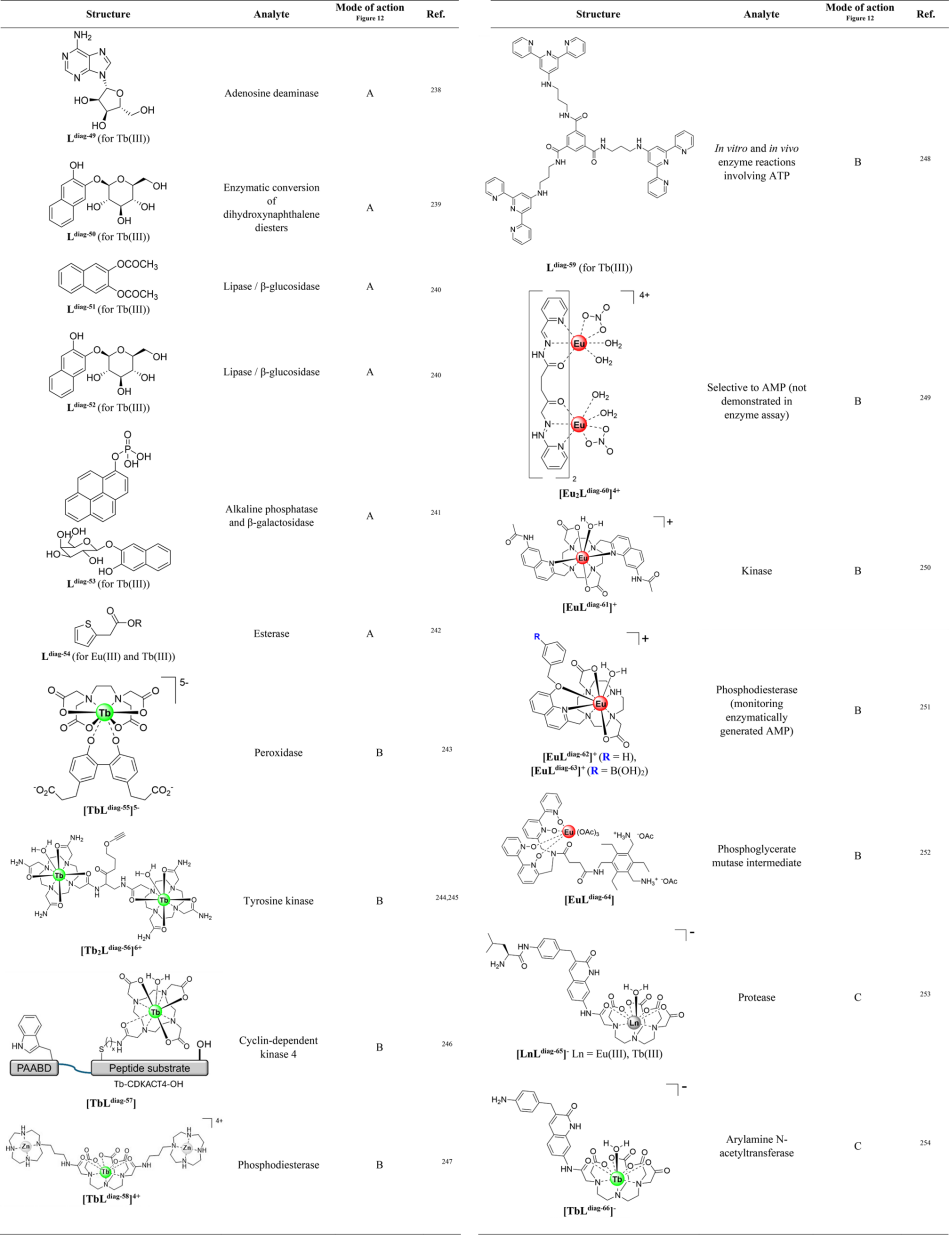

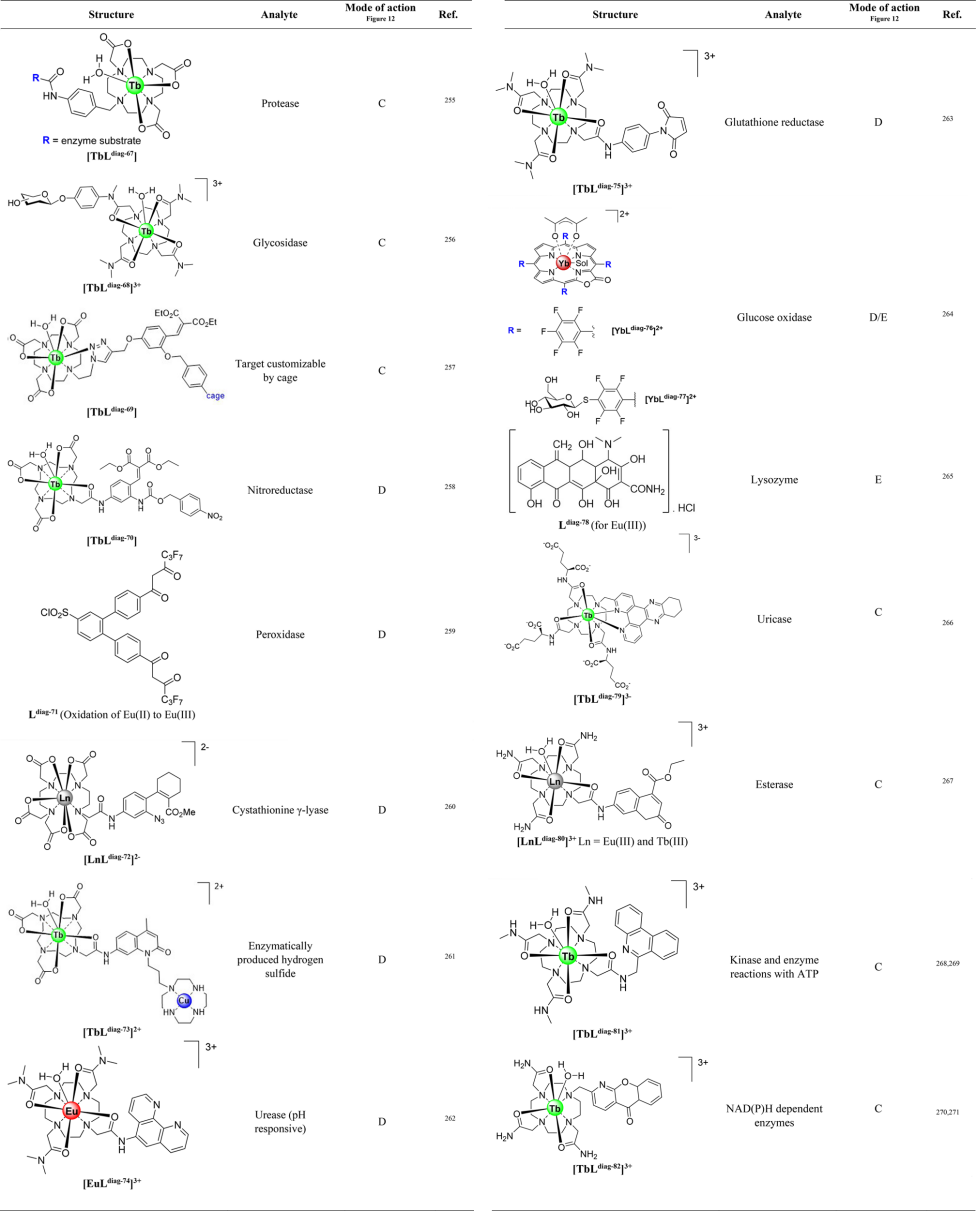
Lanthanide Complexes That Respond to Enzymes

In the simplest case, enzyme reactions convert a noncoordinating substrate, containing an antenna, into a chelating ligand. Binding of these chelates to lanthanide ions leads to a luminescent signal (Figure 12A). Examples in literature are based around substrates that either are potential antennas or are analogues of substrates with pendant chromophores. In the first example, an assay has been developed for adenosine deaminase, where the enzyme converts the noncoordinating adenosine into the bidentate compound inosine. The coordinated inosine (**L**^**diag-49**^) provides an antenna for Tb(III) sensitization, leading to a luminescent product. This system has been used in a simple buffered assay, leading to methods for screening inhibition methods for the enzyme.^238^ Similarly, lipase and related enzymes can be monitored by reaction of ester-based substrates converting these into hydroxyl groups, capable of coordination to lanthanide ions. This principle has been used to monitor enzyme activity by conversion of dihydroxynaphthalene diesters (**L**^**diag-50**^) in the presence of Tb(III)-cholate functionalized vesicles.^239^ Binding of the dihydroxy product to Tb(III) leads to an enhancement in luminescence.

Incorporation of Tb(III) ions into a nonluminescent hydrogel has also resulted in a luminescence triggering event upon enzyme cleavage of the dihydroxynaphthalene diesters and product binding atop the lanthanide ion (**L**^**diag-51**^**, L**^**diag-52**^).^240^ The same group has expanded the same hydrogel approach to monitor substrate reactions for other enzymes including alkaline phosphatase **L**^**diag-53**^, and β-galactosidase.^241^ Other examples include development of assays for monitoring esterase enzymes in cell lysates via sensitization by the thiopheneacetic acid (**L**^**diag-54**^), a product of the corresponding ester substrate.^242^ Incorporation of the lanthanide ion into a chelate ligand provides control over the coordination environment and selectivity of substrate or product binding (Figure 12B). An assay to monitor the activity of peroxidase enzymes has been developed using p-hydroxyphenylpropionic acid as a substrate with hydrogen peroxide to horseradish peroxidase, dimerizing the substrate to a biphenol ligand (**L**^**diag-55**^).^243^ The product reacts with Tb(III)-EDTA, providing an antenna for sensitization of the Tb(III). The assay luminescence is further enhanced by the addition of cesium chloride that facilitates the intersystem crossing from the fluorophore to the lanthanide.

Phosphorylation plays an important role in many biological functions, making assays that monitor enzymes such as kinase of great value. The tetraamide DOTA derivative Tb complex, **[TbDOTAM]**^**3+**^, with one bound water molecule, has been shown to bind to phosphorylated tyrosine residues, with the bound phosphotyrosine providing a chromophore to enhance the Tb(III) luminescence.^244^ Binuclear derivatives of the same ligand have been prepared, carrying a net 6^+^ charge leading to significant increase in luminescence enhancement on binding of phosphotyrosine over the mononuclear complex. Binding for the mono and binuclear complexes with phenyl phosphate (a model compound for phosphotyrosine) was greatly enhanced, with *K*_D_ based on a 1:1 complex to be 110-times lower for the binuclear complex. The increase in luminescence enhancement was therefore attributed to the enhanced guest binding. Selectivity was demonstrated over nonphosphorylated Tyr, pSer, pThr, and other phosphate-containing biomolecules. Furthermore, binuclear complexes with a linker added to the bridging alkane have been synthesized that can be conjugated to proteins using click chemistry, **[Tb**_**2**_**L**^**diag-56**^**]**^**6+**^. The resulting protein bound complexes enabled intermolecular monitoring of kinase phosphorylation of proteins.^245^

**Figure 13:**
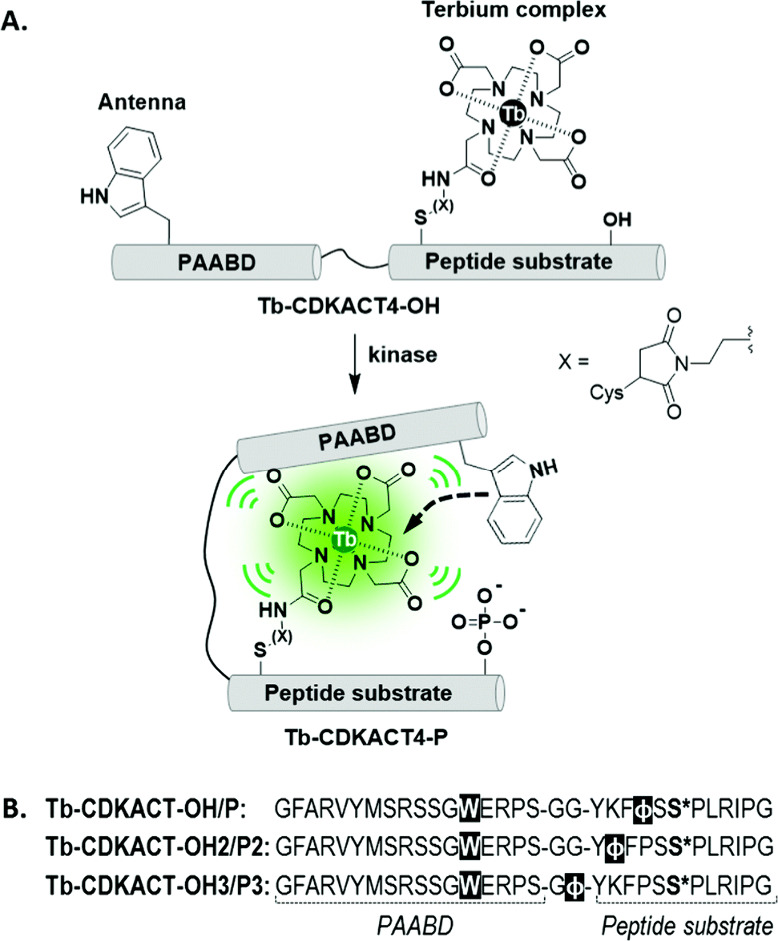
(A) Schematic representation of the biosensor. Upon phosphorylation, the phosphopeptide should bind to the recognition domain (PAABD), leading to a conformational change that should bring together the sensitizing antenna and the terbium complex, resulting in an increase in the luminescence emission. (B) The peptide sequence of the designed biosensors, where the phosphorylatable residue is bold and ϕ = Cys(DOTA-Tb(III)). Reproduced with permission from ref (246). Copyright 2017 Royal Society of Chemistry.

A lanthanide-based peptide receptor has been developed, in which a Tb(III)-DOTA complex conjugated onto the peptide **[TbL**^**diag-57**^**]** is brought into close proximity to a sensitizing tryptophan residue upon phosphorylation of the substrate by kinase, enabling intramolecular sensitization.^246^ The use of Cyclin-dependent kinase 4 (CDK4) peptides is intended to provide lanthanide probes for screening the activity of kinase enzymes mirroring the response of living cells (Figure 13).

Nucleotides are involved in a wide range of biological pathways, including acting as coenzymes with other enzyme substrates. For example, by monitoring the conversion of coenzymes, it is possible to understand kinetics for a wide range of enzyme-based processes. Incorporation of binding pockets on lanthanide complexes provides the means to control the selectivity of host–guest interactions. A trinuclear Tb(III)–Zn(II)_2_ complex has been synthesized based on a cyclen functionalized diethylenetraminepentaacetic acid DTPA-bisamide ligand **[TbL**^**diag-58**^**]**^**4+**^.^247^ The trimetallic complex is formed of a Tb(III) ion binding to the core DTPA-bisamide ligand and one Zn(II) binding to each of the cyclen moieties. Luminescence studies of the trinuculear complex confirm it is weakly luminescent, owing to the lack of chromophores to sensitize luminescence. Titration of 1 equiv of guanine monophosphate (GMP) to the complex results in a ca. 87-fold increase in luminescence, plateauing at 2 equiv, due to the bound GMP sensitizing the Tb(III) (Φ = 0.48; λ_ex_ = 285 nm, reference tryptophan). The same experiment conducted on the mononuclear Tb(III) complex yielded a 2.5-fold enhancement in luminescence by comparison. Binding of GMP to the complexes was attributed to binding to the Zn(II) pendant arms. Addition of guanine di- and tri-phosphates also resulted in an increase in luminescence plateauing at 0.5 and 0.25 equiv, respectively.

In a different approach, a terbium(III) complex based on a *C*_3_ symmetrical terpyridine ligand **L**^**diag-59**^ has been developed for the selective monitoring of ATP both *in vitro* and *in vivo*.^248^ In water, the complex luminescence is quenched by intramolecular π-stacking and coordinated water molecules and is therefore in an “off” state. Upon addition of ATP, the luminescence increase is attributed to guest binding to the Tb(III)-terpyridine-Tb(III), resulting in the probe entering an “on” state. The complexes were found to be selective to ATP over ADP and AMP, enabling the ratios of nucleotides to be determined using signal magnitude and emission decay time, by curve fitting to a Levenberg-Marquart method. This system was optimized to enable the monitoring of ATP-generating enzymes in living cells. From this, a pyruvate kinase M2 assay was optimized for high-throughput screening for novel enzyme inhibitors.^272^

The binding of AMP in preference to ADP and ATP is challenging due to preferential binding of the di- and triphosphate to lanthanide ions **[Eu**_**2**_**L**^**diag-60**^**]**^**4+**^ due to charge and denticity. Coordinatively unsaturated, binuclear Eu(III) helicates have been shown to have preferential binding to AMP over ADP, ATP, and other common anions.^249^ In this case, the preferential binding of AMP is attributed to binding of the AMP in a bridging mode between the two Eu(III) ions in the complex formed from two butanedioicacid-1,4-bis[2-(2-pyridinylmethylene)hydrazide ligands, replacing coordinated waters and therefore leading to an increase in the luminescence of the complex, replacing coordinated waters and therefore leading to an increase in the luminescence of the complex.

A real-time assay for monitoring kinase activity through ATP and ADP has been developed using a substituted cyclen chelate **[EuL**^**diag-61**^**]**^**+**^.^250^ Binding of the nucleotide to the complex results in a significant increase in luminescence, with a greater increase observed for ADP over ATP. Addition of Mg(II) with a preference to ATP binding was used to increase the selectivity of ADP over ATP through competition. In subsequent work from the same group, cyclen complexes bearing sterically crowded 8-(benzyloxy)quinoline pendant arm derivatives, **[EuL**^**diag-62**^**]**^**+**^, **[EuL**^**diag-63**^**]**^**+**^, were demonstrated to present a selective binding cavity for AMP and phosphate. Binding of AMP, phosphate, and to a lesser degree ADP to the complex results in a significant increase in the luminescence of the complex. The receptor complex was tested *in vitro* to monitor phosphodiesterase generation of AMP in real time.^251^

Chromophores incorporated either in the core chelate structure or as pendant arms, can be used to monitor enzyme activity. The signal can be modulated through several means, including binding of guests, reaction of the probe, and binding of the probe to enzymes. A luminescent sensor has been developed for monitoring 2,3-bisphosphoglycerate, a product of phosphoglycerate mutase, a compound that plays a vital role in regulating the oxygen transport by hemoglobin.^252^ The core of the host is based on a tris-functionalized triethylbenzene scaffold providing a host with two ammonium groups to bind the target, and a tetra-N-oxide bipyridine-Eu(III) chelate **[EuL**^**diag-64**^**]**. The host is designed to bind the two phosphate ions of the target. The composition of the solvent was found to be important in this case, with it being necessary to conduct the assays in organic solvents to avoid quenching of the Eu(III) luminescence. With an optimal solvent composition, addition of the target BPG resulted in a quenching of luminescence, with a 1:1 binding event between host and guest observed. The complex was screened against other glycolysis intermediates; phosphoglycerate, 2-phosphoglycerate, and phosphoenolpyruvate resulted in 2:1 host to guest binding. While not applicable to use in water, this ligand system provides an informative example of design of host–guest binding for lanthanide probes.

Chelates with reactive pendant arms provide a powerful means for selective monitoring of enzyme activity. In the following examples, the enzyme reaction alters the chromophore, for example, loss of a quenching group, reaction of the pendant arm leading to lanthanide luminescence excitation quenching, or inducing the formation of a chromophore through initiation of a cascade reaction. The use of a pendant group to modulate energy transfer from the antenna to the lanthanide can be used to turn on or off the luminescent lanthanide signal. In this case, it can either be a functional group modifying the antenna triplet state or a pendant group that deactivates that antenna (Figure 12C). Europium and terbium chelates have been rationally designed as turn-off protease probes for use in inhibitor screening. In this system, a three-part probe has been designed, with a lanthanide chelate, a luminescence switch moiety, and an antenna.^253^ The chelate is based on a DTPA-monoamide ligand with a customized pendant antenna **[LnL**^**diag-65**^**]**^**–**^. Feasibility was demonstrated for leucine aminopeptidase, an enzyme associated with, for example, tumor cell invasion and metastasis. Leucine was incorporated in the on/off switch moiety for the chelate, yielding an active chromophore for the sensitization of lanthanide luminescence. Upon enzyme cleavage of the leucine amide switch, an amine is formed raising the HOMO level, quenching the quinolone antenna and switching off the Ln(III) luminescence. A similar approach with DTPA-based chelates has been employed by the same researchers with customized chelates to screen dipeptidyl peptidase 4 inhibitors,^273^ measuring arylamine N-acetyltransferase activity in recombinant human enzymes and cell lysates **[TbL**^**diag-66**^**]**^**–**^.^254^ A positive response assay has also been developed for protease enzymes based on a DO3A chelate **[TbL**^**diag-67**^**]**. Model compounds of peptides specific to protease enzymes were conjugated onto an aniline derivative, forming a nonemissive acylated compound. Upon reaction with the corresponding enzyme, the peptide bond was cleaved, yielding an aniline antenna capable of sensitizing Tb(III) luminescence (Φ = <0.001 (off), 0.051 (on), reference phenol).^255^ Chelates have been developed to monitor the action of glycosidase enzymes that hydrolyze glycosidic linkages have been developed, based on Tb(III) cyclen chelates with a pendant 1,4-substituted phenyl antenna, conjugated to a carbohydrate unit **[TbL**^**diag-68**^**]**^**3+**^.^256^ The conjugated phenyl antenna is not an effective sensitizer of Tb(III) luminescence. Upon cleavage of the glycosidic bond, the phenol antenna is liberated, producing an antenna that can sensitize Tb(III) luminescence, providing a positive signal for glycosidase enzyme activity. A modular design has been developed to develop turn-on probes to monitor enzyme activity. In the first example, cyclen chelates with a selectively reactive pendant arm have been developed.^149,257^ The pendant arm is a nonsensitizing caged antenna precursor customized with a conjugated analogue of the enzyme substrate **[TbL**^**diag-69**^**]**. In this approach, cleavage of the cage group by the corresponding enzyme leads to formation of a coumarin sensitizer group, switching the probe into an “on” state (Φ Eu 1.08%, Tb 1.63%, reference coumarin 2 or Cs_3_[Ln(dpa)_3_]). The combination of different cage derivatives and lanthanides has enabled assays to be conducted concurrently for monitoring more than one enzyme at a time. Furthermore, the β-galactosidase probe has been used successfully in live cell assays and was found to be nontoxic to the cells.

Enzymes can be used to trigger the formation of antenna groups leading to a luminescent complex **[TbL**^**diag-70**^**]** either by direct action of the enzyme on the ligand or by reaction of the enzyme product with the ligand (Figure 12D). Turn-on lanthanide probes have also been developed for the monitoring of nitroreductase enzyme activity in live bacteria.^258^ In this work, a DOTA-based fluorescent probe has been designed by incorporating a nonsensitizing caged antenna precursor containing a nitrobenzene moiety. Before reaction, the antenna precursor does not transfer energy to the Tb(III) ion in the DOTA-based chelate. Enzyme reduction of the nitro group on the inactive complex triggers a self-immolative fragmentation cascade that leads to the formation of a carbostyril chromophore, turning on the energy transfer from the antenna to the lanthanide, leading to a large signal increase upon activation. The probe was demonstrated to sense nitroreductase in both cell lysates and ESKAPE (*Enterococcus faecium*, *Staphylococcus aureus*, *Klebsiella pneumoniae*, *Acinetobacter baumannII*, *Pseudomonas aeruginosa*, and *Enterobacter* species) family live bacteria.

Peroxidase enzyme activity has been measured through the use of solutions of Eu(II), stabilized using phosphate buffered saline.^259^ Horseradish peroxidase or human myeloperoxidase and hydrogen peroxide were added to the solution with a sensitizer 4,4′-bis(1″,1″,1″,2″,2″,3″,3″-hepatafluoro-4″,6″-hexanedione-6″-yl)chlorosulfo-*o*-terphenyl (BHHCT) **L**^**diag-71**^. In the case of this system, the lanthanide ion is oxidized by the product rather than a change to the ligand. Reaction of Eu(II) in the presence of HRP and hydrogen peroxide oxidized the Eu(II) to Eu(III), leading to the formation of a characteristic luminescent species.

The enzymatic product hydrogen sulfide is sensed by a specially designed lanthanide chelate.^260^ In this case, a triethylenetetramine hexaacetic acid (TTHA) complex with a pendant methyl ester of 2-(2-azidophenyl)-1-cyclohexene-1-carboxylic acid is used as a probe **[LnL**^**diag-72**^**]**^**2–**^ for the enzyme product. Upon reaction of hydrogen sulfide, the pendant arm reacts to a quinolinone that can act as an antenna to sensitize lanthanide luminescence. Activity of the probe was demonstrated *in vitro* using sodium sulfide and hydrogen sulfide produced by cystathionine γ-lyase (CSE). In a subsequent publication,^261^ a heterobinuclear Tb(III)–Cu(II) complex was used to sense hydrogen sulfide. The approach used in this case differed in the use of a Tb(III)-DO3A chelate with a Cu(II)-cyclen pendant arm **[TbL**^**diag-73**^**]**^**2+**^. In this system, the Cu(II)-cyclen complex of the heterobinuclear complex quenches the Tb(III) luminescence of the probe. Upon addition of hydrogen sulfide, the Cu(II) reacts with the sulfur, precipitating from the complex and yielding a mononuclear, luminescent Tb(III) product. The probe has been tested *in vitro* with CSE and using a sodium sulfide stimulated HeLa mammalian cell culture.

A pH-responsive cyclen-based complex with a phenanthroline pendant arm **[LnL**^**diag-74**^**]**^**3+**^ has been reported for the monitoring of catheter urinary tract infections in real time. The mode of sensing for this is through a change in pH caused by urease products.^262^ Urease action results in elevation of the pH of urine, leading to corresponding reduction in luminescence. The pH dependence of the Eu(III) chelate has been reported previously^274^ with luminescence showing a bell-shaped curve. It was predicted that at higher pH the Eu(III) is reduced to Eu(II) and the amide of the chelate is deprotonated. At lower pH, the phenanthroline is protonated, reducing the chromophore’s ability to populate the ^5^D_0_ state efficiently, leading to a reduction in luminescence. The complex has been incorporated into a hydrogel that could subsequently be incorporated into catheters; the luminescence switches off in the advent of a urinary infection as the urine passes through the catheter. A cyclen-based chelate with a maleimide pendant arm has been used to monitor the action of glutathione reductase **[TbL**^**diag-75**^**]**^**3+**^.^263^ In this case, the reactive glutathione product of the enzyme reacts with the maleimide pendant arm through a 1,4-Michael addition. Formation of the succinimide derivative effects the sensitization of Tb(III), leading to an increase in luminescence in a pH 7.4 HEPES buffer. The chelate was tested with other biologically relevant thiols and was found to yield the same increase in luminescence.

A near-infrared porpholactone system has been developed where the lanthanide ion was successfully shielded from coordinated water molecules **[YbL**^**diag-76**^**]**^**2+**^, and quenching in water was attributed to outersphere water.^264^ A glucose conjugate of the chelate was prepared and tested in an assay with glucose oxidase **[YbL**^**diag-77**^**]**^**2+**^, yielding an increase in the emission of the Yb(III) complex. Addition of glucose led to a decrease in the emission. The hypothesized mechanism in this case was the binding of the pendant glucose conjugate of the complex to the binding site of the enzyme, excluding outer sphere quenching water.

A method has been developed for the monitoring of lysozyme through formation of ternary complexes between the antibiotic compound metacycline that bears a β-diketonate chelating group, Eu(III), and lysozyme **L**^**diag-78**^.^265^ Upon binding of the Eu(III)–metacycline complex to lysozyme, there is an increase in the europium-based fluorescence that is attributed to improved energy transfer from the metacycline–lysozyme complex and the Eu(III) and a decrease in nonradiative energy loss through O–H vibrations.

Other than design of the complex, there are additional benefits to the design assays incorporating lanthanide complexes. Measurement of enzyme activity *in vitro* and *in vivo* requires concentrations in the assay to be well understood. Measurement of concentration in live cells is particularly challenging due to the uneven uptake and distribution of the tag in cells. One approach is through the use of ratiometric probes where changes in the assay can be compared relative to a known reference and can operate in a concentration-independent manner.^267^ Lanthanides are well suited to applications in ratiometric probes, as they share common useful attributes, e.g., line-like emission and long luminescence lifetime, but they also can be made to respond differently in the presence of the target due to differences in ligand structure or distinct excited states including the effect of quenchers such as water. These attributes of lanthanides can be used to use more than one lanthanide complex in the same assay or pull-out data from the same type of lanthanide complex through deconvolution of signals, e.g., lifetime. Work by Parker et al. demonstrated the measurement of uric acid *in vitro* through selection of Eu(III) and Tb(III) complexes selected from a panel of candidates through interaction of the uric acid with the complexes.^266^ The ultimate assay was based on a common cyclen-based ligand **[TbL**^**diag-79**^**]**^**3–**^ where Tb(III) luminescence was quenched preferentially by electron transfer from the urate anion. The assay was tested *in vitro* with uric acid standard solutions and urine samples from healthy volunteers with an improved precision for the ratiometric probe over the commercial uricase assay kit tested for comparison.

In one example,^267^ the approach was adopted for the monitoring of esterase activity, whereby the pendant chromophore of the lanthanide complex, containing an ester moiety **[LnL**^**diag-80**^**]**^**3+**^, is converted to the corresponding carboxylic acid with Tb(III)/Eu(III) emission ratios of 0.35 and 1.62, respectively. Ratiometric sensing has also been applied to the field of nucleotide monitoring for enzymes.

Pierre et al. have developed DOTAM-type complexes functionalized with a phenanthridine antenna **[TbL**^**diag-81**^**]**^**3+**^ for the monitoring of ATP *in vitro*.^269^ The probe is designed to distinguish between nucleotides in buffered neutral solution, binding preferentially with ATP over ADP and AMP. The binding event is attributed to stacking of ATP with the phenanthridine antenna which induces PET quenching on the antenna and consequently turns off the Tb(III) luminescence. Selectivity between nucleotides is most likely driven by electrostatic interactions between the positively charged terbium complex and negative charges of the three nucleotides, with ATP carrying the greatest charge. Guest binding for an equivalent DOTA complex, yielding neutrally charged complexes with lanthanides, was compared to the positively charged DOTAM and did not distinguish between tr-, di-, and mono-nucleotides.^268^ A ratiometric assay was developed based on the differing affinities of these two types of complexes, using **[Tb-DOTAM-Phen]**^**3+**^ and **[Eu(DOTAPhen)]**. The system was demonstrated in a ratiometric kinase *in vitro* assay, monitoring the catalytic domain PKAc that is a cancer therapy target. It was noted in this case that the lack of selectivity between different types of nucleotides, e.g., ATP and GTP, precludes use in cells, as the results would be hard to interpret. Oxidoreductase enzymes use nicotinamide adenine dinucleotide (phosphate) (NAD(P)) cofactors; however, static quenchers such as ATP can interfere with these assays.^271^ To address this, lanthanide probes have been used with time-domain ratiometry to further use the time domain for discrimination in assays. In this case, the time-domain of a terbium complex, sensitive to dynamic quenching from NAD(P)H,^270^ is used as the ratiometric probe **[TbL**^**diag-82**^**]**^**3+**^, measuring the luminescence intensities of two successive time regions. By comparison, ATP is a static quencher. The principle of the assay was demonstrated through a coupled reaction of hexokinase, using ATP to convert glucose to glucose-6-phosphate, followed by glucose-6-phosphate dehydrogenase with conversion of NAD(P)^+^ to NAD(P)H. It was possible to separate out the two forms of quenching, distinguishing NAD(P)H through time-domain ratiometry.

A ratiometric sensor using Tb(III) composite containing carbon dots has been used to measure glucose oxidase action.^275^ The composite is composed of carboxyphenylboronic acid (CPBA), acting as an antenna for Tb(III) luminescence, adenosine monophosphate, Tb(III), and glucose oxidase. CPBA-sensitized Tb(III) emission is quenched upon conversion of glucose to hydrogen peroxide, the product deboronating the carboxyphenylboronic acid (CPBA) antenna. Carbon dots in the composite are not impacted. In this case, the enzyme confined in the composite displayed enhanced activity.

### 2.4 Development of Receptors for Anion Recognition in Water

Anions are ubiquitous in the natural world and play a key role in defining biology and the environment. Despite this, the discussion of coordination chemistry was framed from the perspective of the metal ion for many years; it is only more recently that anions have been given the attention they deserve.^276,277^ The development of potent anion receptors to recognize and sense biologically and environmentally important anionic species in aqueous conditions has emerged as a significant area of research in the field of chemical sensors.^278^

Water is a highly competitive polar solvent due to its ability to act as both a hydrogen bond donor and acceptor. Anions possess intrinsic properties that make them difficult to bind effectively in aqueous solution. This is because anions are more heavily solvated in water than analogous cations of the same charge and similar size; they also display a much greater range of hydrophilicity/hydrophobicity, and their recognition is further complicated by the multiple protonation equilibria. Therefore, anion recognition in water requires sufficient binding free energy to overcome the high hydration energies of anions in water. Strong electrostatic and metal–ligand interactions are required to overcome the anion hydration energy and allow binding in aqueous conditions.^279^ The nature and abundance of potentially interfering species such as competing anions, cations, and complex ionic biomolecules (e.g., proteins and nucleic acids) define the requirements for selectivity of the receptor.^33,34,278,280^Table 6 summarizes the lanthanide complexes that bind to biologically relevant anions in solution. Examples of lanthanide systems tested in cells are described in section 5.2.2.

**Table 6:**
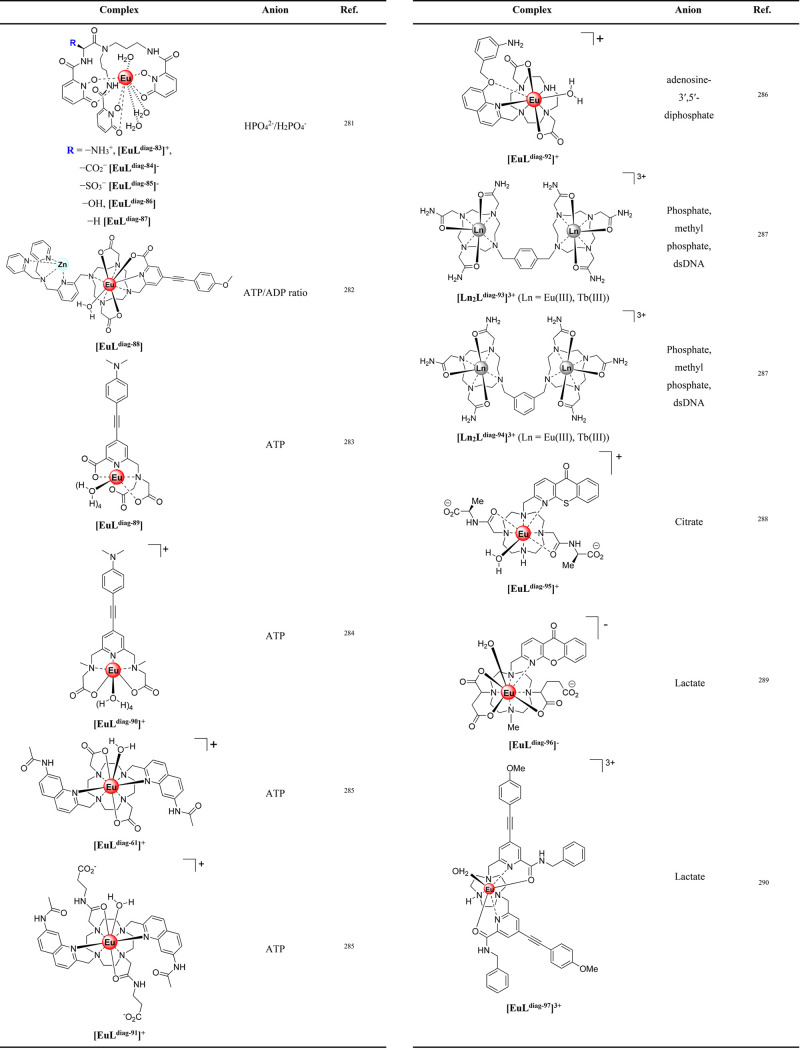

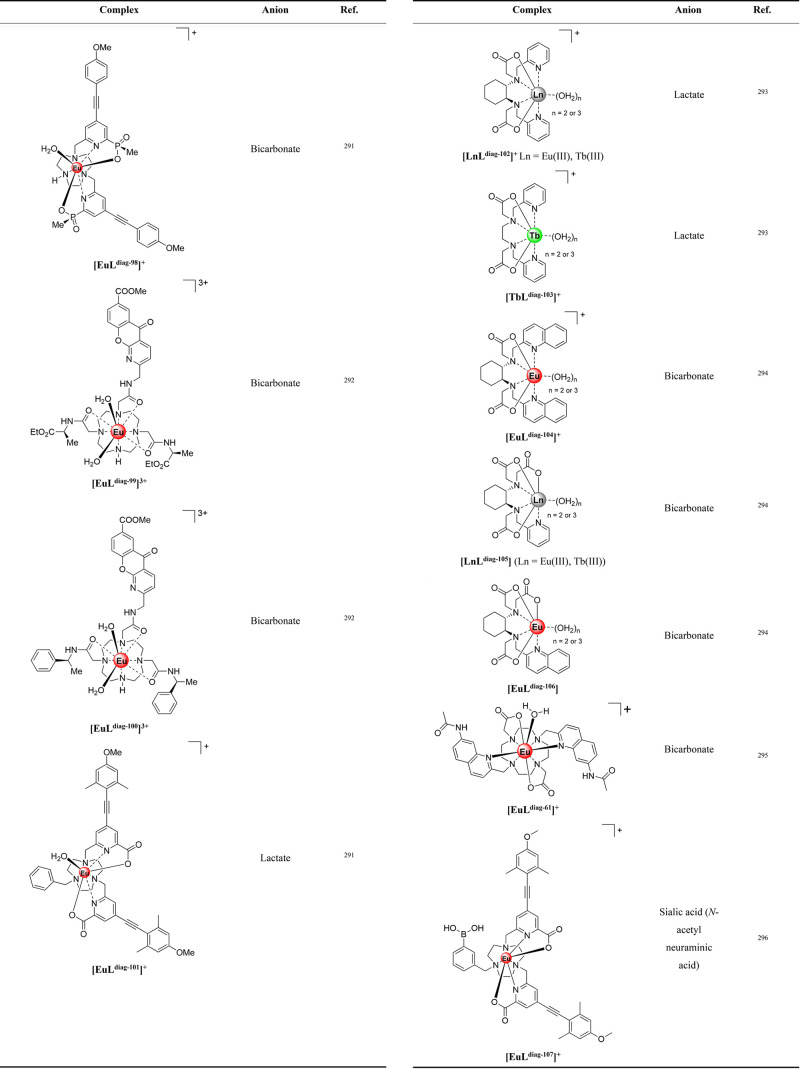

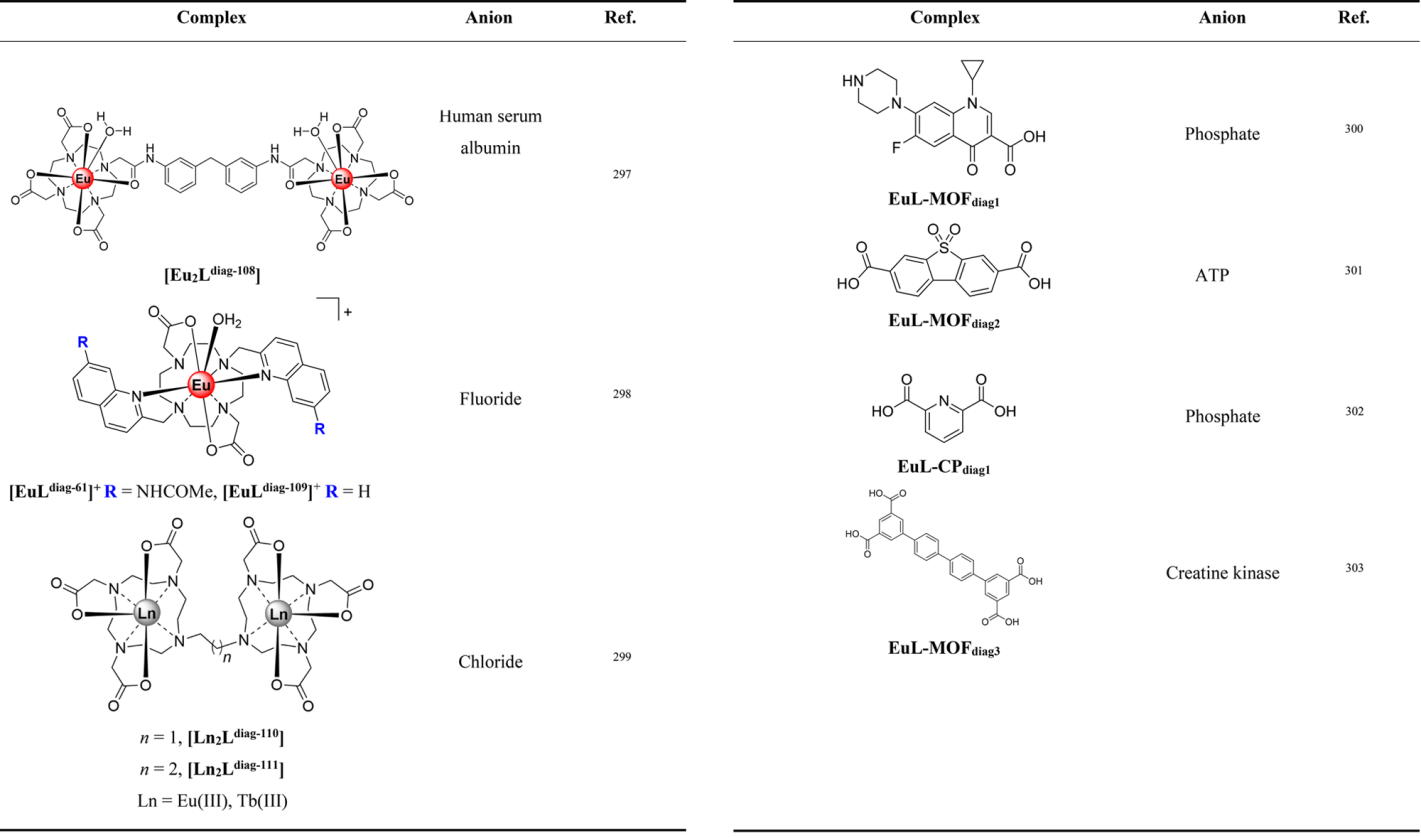
Lanthanide Complexes That Bind to Biologically Relevant Anions in Solution

#### 2.4.1 Luminescent Lanthanide Complexes for Anion Binding and Sensing

Anion recognition by lanthanide complexes can reflect either collisional quenching of excited states by anions^40,304^ or anion binding.^34^ The former interaction is rather weak, which makes it a less attractive approach to probe anions in biology. The strongest interaction that can be used for anion binding in water is direct coordination to a metal cation.^34^ Metal-containing receptors are typically designed around an organic scaffold that binds metal cations with high kinetic and thermodynamic stability such that at least one coordination site on the metal center remains vacant or coordinated to a weakly bound ligand (like water) and thus available for anion binding. The metal can induce a geometrical preorganization of the complex that results in a better host–guest complementarity.^1,34^ The long luminescence lifetimes (up to milliseconds) enable time-gated or time-resolved measurements to distinguish the lanthanide-centered emission from the background short-lived autofluorescence.^25,26,305^ This feature is particularly advantageous if the complex is to be used as a probe in complex aqueous media such as in biological and environmental samples.^235,306,307^

Anion binding is explored directly at the metal center by the displacement of one or more inner-sphere water molecules. This, in turn, alters the Ln(III) coordination environment and the local ligand field, resulting in changes in the emission intensity, spectral form, and lifetime of the complex.^35,308,309^ These properties readily enable determination of the mode by which the lanthanide receptor binds anion as well as the stoichiometry of the receptor–host complex. This is best reported by changes in the emission spectra of Eu(III) complexes.^33,34^ The absence of degeneracy in the emissive ^5^D_0_ excited state of Eu(III) simplifies its emission spectral form. The intensity of the magnetic dipole-allowed Δ*J* = 1 transitions at ∼590 nm is relatively unaffected by any coordination change, whereas the hypersensitive electric dipole-allowed Δ*J* = 2 transitions at ∼615 nm are strongly perturbed.^310^ This causes differences in the relative intensity of this pair of emission bands allowing ratiometric analysis.^288,289,311,312^ This strategy has been utilized extensively toward anion recognition in water and in biological media. By varying the charge density on the lanthanide ion and modifying the ligand architecture to change overall charge or modulate steric demand at the metal center, the binding selectivity for an anion can be controlled. This allows the desired anion affinity to be tuned to the relevant concentration range in the background medium.^308^ The efficacy of lanthanide complexes to recognize anions have been extensively reviewed,^28,33,34,310,313^ and here, we focus on certain biologically relevant anions.

#### 2.4.2 Phosphate Recognition

The development of phosphate receptors that are both effective and selective in water is key to the treatment of medical conditions such as hyperphosphatemia^314^ and ectopic mineralization^315^ and to address environmental problems such as the eutrophication of surface water.^316,317^ The design of many synthetic supramolecular receptors for phosphate relies on the interplay of electrostatic and hydrogen bonding interactions.^318,319^ Ln(III)-based receptors are well suited for sensing phosphate species in water due to the inherent affinity of Ln(III) ions for hard oxyanions.^33,34,308,320^ The recognition of inorganic phosphate is particularly challenging because of its high hydration energy and pH-dependent speciation. Developing Ln(III)-based receptors for phosphate or AMP is difficult because other oxyanions such as ATP, bicarbonate, and lactate can compete for coordination to the Ln(III) center.^33,34,320^

Pierre et al.^281^ developed a series of acyclic tripodal Eu(III) complexes of hydroxypyridinone-based ligands containing a single charged group (−NH_3_^+^, **[EuL**^**diag-83**^**]**^**+**^, −CO_2_^–^**[EuL**^**diag-84**^**]**^**–**^, or −SO_3_^–^**[EuL**^**diag-85**^**]**^**–**^) or neutral hydrogen-bonding moiety (−OH, **[EuL**^**diag-86**^**]**, −H **[EuL**^**diag-87**^**]**) on the periphery with an open coordination site that bind 2 or 3 molecules of phosphate with high affinity and very good selectivity.

Parker et al. reported a series of Eu(III)–Zn(II) complexes capable of monitoring the ATP/ADP ratio by induced circularly polarized luminescence.^33,282^ The complexes were designed based on the highly emissive EuroTracker probes, containing one alkynylpyridine chromophore and a substituted picolylamine moiety to hold the Zn(II) metal. The differences in the ligand structure were found to modulate binding affinities for both Zn(II) and nucleoside phosphate anions. This *d*–*f* hybrid complex **[EuL**^**diag-88**^**]** binds to ATP and ADP in 0.1 M HEPES at pH 7.4 to induce strong CPL signals of the opposite sign, which facilitated the monitoring of changes in the emission dissymmetry factor as a function of the ratio of ATP/ADP. Circularly polarized luminescence data supported by DFT calculations revealed differences in the chirality of the ATP and ADP adducts of **[EuL**^**diag-88**^**]**, which differ in the arrangement of the exocyclic ring substituents (Λ/Δ). DFT studies indicate the terminal phosphate of the nucleotide bridges the Zn(II) and Eu(III) ions and suggest that the Δ diastereomer is preferred for ATP and the Λ isomer for ADP or AMP.^282^

Rather than using a macrocycle to host the lanthanide metal, Schäferling et al. used a modified aryl alkynylpyridine as a ligand to chelate Eu(III) and investigated the prospects of nucleoside phosphate anion binding.^283,284^**[EuL**^**diag-89**^**]** with four inner sphere waters displayed a 60-fold enhancement in Eu(III) emission upon the addition of 1 mM ATP (or 1 mM pyrophosphate), vs a 27-fold increase with ADP. However, in the presence of Mg(II) ions, discrimination between ATP and ADP was lost due to the competitive interaction of ATP with Mg(II). However, at lower concentrations of Mg(II), it was possible to track the ATPase catalyzed conversion of ATP to ADP by measuring a decrease in luminescence as a function of time.^33,283,284^ A cationic version of this complex having two ethyl 2-(methyl amino) acetate arms **[EuL**^**diag-90**^**]**^**+**^ was found to have high luminescence response for ATP over ADP, although no binding constants were reported. **[EuL**^**diag-90**^**]**^**+**^ was used to monitor apyrase activity at pH 6.5 by following a decrease in Eu(III) luminescence as ATP was converted to ADP/AMP.^284^

Butler et al. investigated a series of *C*_2_ symmetric Eu(III) complexes toward the binding to nucleoside phosphates. **[EuL**^**diag-61**^**]**^**+**^ and **[EuL**^**diag-91**^**]**^**+**^ discriminated ATP, ADP, and AMP in the presence of 5 mM Mg(II) ions.^285^**[EuL**^**diag-91**^**]**^**+**^ binds most strongly to ATP (log *K*_a_ 5.8), resulting in 24-fold increase in intensity of the Δ*J* = 2 emission band. Despite similar binding affinity for ADP to **[EuL**^**diag-91**^**]**^**+**^, only a smaller increase in emission was observed. The high affinity of **[EuL**^**diag-91**^**]**^**+**^ for ATP is primarily due to strong metal–ligand interactions, complemented by hydrogen bonding to the quinoline amide arms projecting from the same face of the receptor. NMR and crystallographic studies reveal a bidendate mode of binding of ATP to **[EuL**^**diag-91**^**]**^**+**^ in a 1:1 host–guest stoichiometry. Cellular uptake studies in NIH-3T3 cell line show localization in mitochondria, permitting real-time visualization of elevated mitochondrial ATP levels.^285^

Heparan sulfotransferases mediate the attachment of a sulfate group to an atom in the heparanosan/heparan sulfate polysaccharide.^321^ The sulfated products from this reaction play important roles in cell communication and also feature in various pathologies including cancer, Alzheimer’s, and the mucopolysaccharidoses.^322^ Heparan sulfotransferases belong to the wider group of sulfotransferases found across nature that use the universal sulfate donor compound adenosine-3′-phosphate-5′-phosphosulfate and produce adenosine-3′,5′-diphosphate as byproduct.^323^ Butler et al. developed a Eu(III)-based probe **[EuL**^**diag-92**^**]**^**+**^ that binds reversibly to both adenosine-3′-phosphate-5′-phosphosulfate and adenosine-3′,5′-diphosphate, producing a larger luminescence enhancement with the latter anion.^286^

Morrow et al.^287^ have studied the sensing of biologically relevant anions by exploiting luminescence resonance energy transfer in heterodinuclear Ln(III) complexes of the macrocycles **[Eu**_**2**_**L**^**diag-93**^**]**^**3+**^ and **[Eu**_**2**_**L**^**diag-94**^**]**^**3+**^ (Ln(III) = Eu, Tb). The binding of phosphate, methylphosphate, double-stranded DNA, a DNA hairpin loop, and fluoride to both Eu(III) centers in **[Eu**_**2**_**L**^**diag-93**^**]**^**3+**^ (pH 7.0, 0.1 M NaNO_3_) is studied by monitoring the ^7^F_0_ → ^5^D_0_ excitation peak. The ^5^D_0_ → ^7^F_0_ transition occurs between two electronic states that are nondegenerate and are thus not split by ligand fields; this gives rise to a single excitation peak for each Eu(III) complex species, barring the overlap of peaks. The recovered luminescence lifetime data indicate anion binding to the metal centers by the replacement of coordinated water molecules. There are distinct changes in the relative intensities of Eu(III) emission peaks for complexes of **[Eu**_**2**_**L**^**diag-93**^**]**^**3+**^ and **[Eu**_**2**_**L**^**diag-94**^**]**^**3+**^ with phosphate, fluoride, carbonate, and phosphate ester ligands. The Eu(III) luminescence lifetime data for the binuclear complexes bound to these anions can be fitted to a single exponential decay upon addition of saturating ligand concentrations.^287^ Other works on phosphate binding lanthanide probes have been extensively reviewed.^33,237,320^

Lanthanide metal–organic frameworks Ln-MOFs are also quite popular for cation and anion sensing as they provide attractive structural platform designs for specific ion recognition.^324^ A visual and ultrasensitive ratiometric fluorescent detection of phosphate was achieved with a dual-functional **EuL-MOF**_**diag1**_ based on an Eu-ciprofloxacin.^300^ Ciprofloxacin functions as an energy donor and results in the fluorescence enhancement of Eu(III); the introduction of pyromellitic acid can cause the aggregation, and red characteristic fluorescence of Eu(III) is further enhanced. When phosphate is added, ciprofloxacin is released from the MOF, and the red fluorescence of Eu(III) is quenched while blue fluorescence of ciprofloxacin is recovered. The limit of detection is ultrasensitive and reaches 4.4 nM. The ratiometric fluorescent probe has been successfully used in real human urine samples.^300^ A novel **EuL-MOF**_**diag2**_ based on the coordination of 5,5-dioxo-5H-dibenzo[b,d]thiophene-3,7-dicarboxylic acid with Eu(III) was shown to effectively detect ATP via a luminescent quenching mechanism vs ADP or monophosphate nucleoside.^301^

A nanoparticle based on a coordination polymer **EuL-CP**_**diag1**_ shows an increase in the red luminescence upon phosphate addition.^302^ The luminescence enhancement is attributed to the inhibition from quenching of H_2_O molecules in the coordination sphere and the improvement of energy transfer by shortening the distance between the ligands and Eu ions. The quantitative detection of phosphate in biological fluids is demonstrated.^302^

A series lanthanide MOF complexes are constructed for detection of different biomarkers including nitrofuran-based antibiotics,^325,326^ urinary thiodiglycolic acid,^327^ serotonin,^328^ and sulfamethzine (SMZ), metronidazole (MDZ), and dimetridazole (DMZ) antibiotics,^329−332^ in biological samples with low detection limits. An **EuL-MOF**_**diag3**_ shows a highly porous structure adopts a four-connected lvt topology with one-dimensional (1D) channels of about 1.8 nm. The red Eu emission can be quenched by ATP but less influenced by ADP, which make it a luminescent probe for creatine kinase, a transformative switch between ATP and ADP. The nanoprobes showed superior selectivity and reliability toward the practical detection of creatine kinase activity in human serum. The detection limit was 1.0 U/L, and the linear range of the quantitative assay kit of creatine kinase activity was 1.2–156.2 U/L.^303^

#### 2.4.3 Carboxylic Acids and Bicarbonate Recognition

Lactate and citrate are biological anions which are biomarkers for prostate and breast cancer,^333^ and elevated lactate levels are associated with Parkinson’s disease.^334^ Lactate and citrate are key metabolites in the intermediary metabolism of the cell. Lactate is the important final product of anaerobic and hypoxic glucose metabolism via glycolysis. Citrate is a key metabolite in the Krebs cycle (citric acid cycle) of virtually every aerobic cell. The synthesis and oxidation of citrate provide the major energy supply (∼70%) of cells. Citrate is also a source for fatty acids and cholesterol metabolism.^335,336^

A family of nine luminescent Eu(III) complexes, based on a cyclen macrocyclic core, which incorporates a heterocyclic azaxanthone or azathiaxanthone moiety for facilitating the sensitization of lanthanide emission, was evaluated for their binding affinity for lactate, citrate, and bicarbonate in solution by observing the change in the intensity ratio of the Eu emission bands at 616/686 nm as a function of added anion.^289^ Pronounced selectivity for citrate or lactate was observed with the cationic complexes in which the ring NH group was retained. For example, **[EuL**^**diag-95**^**]**^**+**^ exhibits citrate/lactate affinity constants ratio of 42:1. Lactate binding is preferred by the more sterically demanding oxo-anionic complex **[EuL**^**diag-96**^**]**^**–**^, in which the side chain glutarate is introduced to tune anion binding to the desired range and suppress citrate complexation, which exhibits the lactate/citrate binding ratio of 30:1, allowing lactate to be measured in the range 10 to 100 mM in the presence of up to 100 mM citrate. This methodology has been applied to the measurement of citrate in seminal fluid samples. The concentration of citrate in 0.5 μL seminal fluid samples was assessed using the ratiometric analysis of two well separated Eu(III) emission bands.^288,289^

The high sensitivity of circularly polarized luminescence (CPL) spectroscopy to subtle changes in Ln(III) coordination environment is exploited for sensing chiral moieties using Ln(III) probes. Eu(III) complexes (*q* = 1) of achiral triazacyclononane-based ligands bearing arylalkynyl pyridyl antenna with phosphinate, amide, or carboxylate donors, reported by Parker et al.,^290^ bind chiral carboxylic acids and α-hydroxy acids such as lactate and mandelate, forming 1:1 adducts. Anion binding was monitored by emission spectroscopy and signaled by the switching on of strong circularly polarized emission. The strongest binding to lactate was observed for the tricationic Eu(III) bis(amide) complex **[EuL**^**diag-97**^**]**^**3+**^ with log *K*_a_ = 4.57, whereas the bis(phosphinate) complex **[EuL**^**diag-98**^**]**^**+**^ showed the weakest affinity for lactate (log *K*_a_ = 2.76). Luminescence lifetimes of the lactate adducts in water and D_2_O revealed *q* = 0 for **[EuL**^**diag-97**^**]**^**3+**^ and *q* = 0.6 for **[EuL**^**diag-98**^**]**^**+**^, respectively, indicating chelation of lactate to the former complex via the OH and CO_2_^–^ oxygens, forming a 5-ring chelate, in which there is one O–H oscillator in the Eu(III) coordination environment.^290^

Bicarbonate plays important roles in the regulation of cellular pH, removal of metabolic waste, and kidney function.^337^ Probes capable of monitoring spatiotemporal bicarbonate dynamics in living cells would provide a deeper understanding of the diverse biological processes this anion controls, potentially facilitating the development of new therapeutic agents (e.g., channel replacement therapies). Given that the concentration range of HCO_3_^–^ in human serum is 24–27 mM, around 10–20-times higher than other oxyanions (including phosphate, lactate, and citrate),^34^ it was proposed that selective sensing of HCO_3_^–^ could be attained.

Parker et al.^291^ have reported a series of Eu(III) complexes of a triazacyclononane-based ligand with two strongly absorbing alkynyl pyridyl chromophores, generating “bright” complexes. The binding of oxy anions such as carboxylates, lactate, and citrate at the metal center by the displacement of coordinated water molecule has been studied by emission. Upon the binding of bicarbonate, there is a pronounced change in emission spectral form, characterized by an increase in intensity of the Δ*J* = 2 band and a 30% rise in the ratio of the intensities of the Δ*J* = 2/Δ*J* = 1 manifolds. Selectivity of **[EuL**^**diag-98**^**]**^**+**^ for bicarbonate allows its rapid determination in human serum with *K*_d_ = 37 mM by means of a ratiometric analysis of the bright Eu(III)-based luminescence. Parker et al.^292^ have developed Eu(III) and Tb(III) cyclen complexes of four chiral ligands incorporating an azaxanthone sensitizer and studied their binding affinities with bicarbonate, lactate, citrate, phosphate, and serum albumin. The anion binding event was signaled by modulation of circularly polarized luminescence, and the affinity constants were measured by examining changes in emission intensity ratios. **[EuL**^**diag-99**^**]**^**3+**^ showed selective binding to bicarbonate (log *K*_a_ = 3.85, 0.1 M NaCl, pH 7.4)^292^ in the presence of endogenous anions such as phosphate, lactate, and citrate when present at their common biological concentrations. The concentration of bicarbonate was determined directly in cellular mitochondria and in human serum by measuring the ratio of the intensity of a pair of emission bands within the Eu(III) emission spectrum or a red/green band intensity ratio when using mixtures of Eu(III)/Tb(III) complexes of a common ligand.^292^ Measurements of Eu(III)/Tb(III) emission intensity ratios report changes in the steady-state bicarbonate concentrations both *in vitro* and *in cellulo*. Furthermore, the Eu(III) complex **[EuL**^**diag-100**^**]**^**3+**^ was able to selectively stain the mitochondrial region of HeLa cells and to increase the image intensity upon increasing percentage of CO_2_. The Tb(III) complex **[TbL**^**diag-100**^**]**^**3+**^ showed negligible changes in emission, thereby allowing ratiometric analysis of intracellular HCO_3_^–^ levels by monitoring the red/green (600–720/450–570 nm) emission ratio.^292^

The ability of the Eu(III) complexes of triazacyclononane-based ligand with two strongly absorbing arylkynyl pyridyl chromophores to signal lactate binding by the switching on of CPL was demonstrated.^291^ Addition of *R*- and *S*-lactate to each complex in methanol gave rise to mirror image induced CPL spectra. The *N*-benzyl complex **[EuL**^**diag-101**^**]**^**+**^ produced the strongest CPL signal, ascribed to helical alignment of the benzyl and two pyridyl groups, creating a more rigidified chiral structure. The *R*-enantiomer gave rise to a common induced CPL signature across the series of Eu(III) complexes, identified by the Δ*J* = 4 emission band around 700 nm.^291^

The chiral Tb(III) complex **[TbL**^**diag-102**^**]**^**+**^, developed by Piccinelli et al.,^293^ binds lactate weakly in water. The ligand is based on a *trans*-1,2-diaminocyclohexane scaffold, which upon binding, a Ln(III) ion creates a dissymmetric environment with *q* = 2. Lactate displaces the two inner-sphere water molecules, signaled by an increase in Tb(III) emission intensity, particularly the band at 546 nm, which plateaued after the addition of 100 equiv of lactate. In contrast, the achiral complex **[EuL**^**diag-103**^**]**^**+**^ did not show CPL activity due to the flexible nature of the ethylenic group allowing the interconversion between the two isomers compared with the cyclohexane derivative. The potential biological utility of **[EuL**^**diag-102**^**]**^**+**^ was demonstrated by detecting lactate levels in a commercial aqueous Ringer’s solution, commonly used to treat metabolic acidosis.^293^

The binding of a series of Eu(III) and Tb(III) complexes of chiral hexadentate ligands to bicarbonate was reported by Piccinelli et al.^294^ The complexes differ in their overall charge, steric hindrance at the metal ion, and lipophilicity of the heterocycles which impact on the stability of the host–guest complexes. Each complex possesses either two or three inner-sphere water molecules (*q* = 2.5).^338^ Addition of HCO_3_^–^ to the Eu(III) complexes in water (pH 7.4) gave rise to an increase in intensity of the Δ*J* = 2 band and a small decrease in the Δ*J* = 0 band. The cationic complexes **[LnL**^**diag-102**^**]**^**+**^ and **[LnL**^**diag-104**^**]**^**+**^ were shown to bind two HCO_3_^–^ ions with relatively high affinity, in the range log *K*_a_ = 4.62–5.94. In contrast, the neutral and less sterically hindered complexes **[LnL**^**diag-105**^**]** and **[EuL**^**diag-106**^**]** bind only one equivalent of HCO_3_^–^ with lower affinity in the range log *K*_a_ = 2.06–3.11 probably due to the negative charge of the 1:1 adducts which disfavors binding of a second HCO_3_^–^ ion.^294,338^

Butler et al.^295^ have exploited the luminescent Eu(III) complex **[EuL**^**diag-61**^**]**^**+**^ to study the transmembrane transport of bicarbonate. It binds reversibly to HCO_3_^–^ in aqueous solution and shows an increase in Eu(III) emission intensity upon binding.^295^ Sørensen et al.^339^ have investigated the binding of HCO_3_^–^ to Eu(III) complexes of classical DOTA and DO3A ligands. The study highlighted the influence of buffer type, ionic strength, and pH on anion affinity. **[EuDO3A]** binds to HCO_3_^–^ in aqueous buffer by displacing the two coordinated water molecules but was not selective for HCO_3_^–^ over other oxyanions, including HPO_4_^2–^, lactate, and citrate. The luminescence response of **[EuDO3A]** in the presence of 30 mM HCO_3_^–^ was also shown to be pH-dependent, with the emission signal significantly increasing at higher pH (from 6.8 to 8.0).^339^

Sialic acids are saccharides from a diverse family of nine-carbon naturally-occurring 2-keto-deoxy-nononic acids, with a carboxy group at the anomeric carbon atom that gives the molecule a negative charge at physiological pH (p*K*_a_ = 2.2). The most common of them is *N*-acetylneuraminic acid.^340^ It has been demonstrated that sialic acids are relevant biomarkers of metastatic activity of tumors and that the amount of sialic acid expression on the surface of cancer cells correlates with the prognosis of patients.^341^ Parker et al. reported a complex **[EuL**^**diag-107**^**]**^**+**^ bearing a phenylboronic acid group. This design incorporated a coordinatively unsaturated Eu(III) center from a TACN derivate with two aryl-alkynyl pendant arms, which can chelate the carboxylate of *N*-acetylneuraminic acid, while the arylboronate forms a boronate ester with the vicinal diols present in the saccharide. **[EuL**^**diag-107**^**]**^**+**^ binds reversibly to sialic acid and lactic acid, as signaled by changes in the Eu(III) total emission spectrum and the induction of strong circularly polarized luminescence.^296^ The CPL signature is distinctive for *N*-acetyl neuraminic acid and differs from that observed with methyl sialate and *N*-acetyl glucosamine. The reversible binding of sialic acid by the boronate complex **[EuL**^**diag-107**^**]**^**+**^ was signaled via the induction of a “fingerprint” CPL response accompanying changes in the total emission spectrum.^296^

Wong et al. identified a binuclear DO3A-based ligand bridged by two benzyl groups with a bent geometric shape **[Eu**_**2**_**L**^**diag-108**^**]**, like Bisphenol A.^297^**[Eu**_**2**_**L**^**diag-108**^**]** binds to human serum albumin with the binding constant of log *K* 4.87, determined ratiometrically. Bisphenol A is known for its ability to bind to proteins and DNA. Tb(III) analog showed less luminescence enhancement than the Eu(III) variant due to the bathochromic shift of the ligand as a result of the protein’s interaction with the triplet state of the ligand. Cellular uptake studies in HeLa cell lines suggest localization in the cytoplasm, and no significant cytotoxicity was observed.^297^

#### 2.4.4 Halide Recognition

Fluoride is an essential element for healthy teeth and bones, leading to artificial fluoridation of water in some countries. However, excess fluoride consumption leads to dental and skeletal fluorosis as well as acute gastric problems and kidney failure. Therefore, quantitative determination of fluoride in water and in biology is emerging. Ln(III) complexes of *C*_2_ symmetric DO2A ligands with two inner-sphere water molecules readily form the Ln–F bond reversibly. Butler has developed two water-soluble luminescent probes **[EuL**^**diag-61**^**]**^**+**^ and **[EuL**^**diag-109**^**]**^**+**^ capable of binding and sensing fluoride in water with high selectivity over other anions including Cl^–^, Br^–^, I^–^, HPO_4_^2–^, CH_3_CO_2_^–^, HSO_4_^–^, and NO_3_^–^.^298^ Fluoride binds reversibly to each probe, displacing the coordinated water, resulting in a 9-fold enhancement in Eu(III) emission intensity and dramatic changes in emission spectral form, particularly within the Δ*J* = 1 band around 595 nm, which enabled quantification of fluoride in drinking water samples, within the environmentally relevant concentration range of 20–210 μM.^298^

Chloride is the most abundant transportable anion in all cells of the body, and it performs fundamental biological functions in all tissues. It is the main physiological anion, serving as the principal compensatory ion for the movement of major cations such as Na(I), K(I), and Ca(II) across cell membranes. Anion channels are proteinaceous pores in biological membranes that allow the passive diffusion of anions along their electrochemical gradient. Chloride channels reside both in the plasma membrane and in intracellular organelles. Chloride deficiency leads to cystic fibrosis and epilepsy. Despite its importance, there are less than a handful of synthetic receptors for chloride recognition mainly due to poor solvation of the host in water and the large hydration sphere of the anion itself. Faulkner et al. achieved chloride binding in water by using two coordinatively unsaturated DO3A centers bridged by flexible ethane or propane spacers, forming **[Ln**_**2**_**L**^**diag-110**^**]** and **[Ln**_**2**_**L**^**diag-111**^**]**.^1,299^ The binding affinity of chloride in water and in buffers ranges from log *K* 3–4, as determined by Eu(III) luminescent titrations upon direct excitation at the metal center. Although these binuclear systems recognize fluoride too (weaker than chloride in buffered media), the concentration of chloride in physiology (in mM) outweighs the concentration of fluoride (in μM), which makes these binuclear receptors selective toward chloride. Luminescent titration experiments with **[EuDO3A]** derivative suggest the need for two coordinatively unsaturated metal centers at proximity to facilitate chloride chelation. This work paves the way for a new generation of these complexes with light-harvesting antenna for the monitoring of chloride in biological applications.^299^

## 3 Luminescent Lanthanides as Probes in Cellular Imaging

Lanthanide luminescence is attractive for probing cellular environment due to the large Stokes shift and the characteristic long-lived signal which is easily distinguished from any background signal in the cellular environment. We have reviewed the lanthanide complexes as probes used in cellular imaging based on their ligand framework. The ligand framework is important for the stability of the lanthanide complex, its sensitizing activity, and also in its function for cellular uptake. Table 7 has summarized different ligand systems and the related luminescent lanthanide complexes for cellular imaging. The mechanisms governing the cellular uptake of lanthanide complexes have been reviewed elsewhere.^306,342^

**Table 7:**
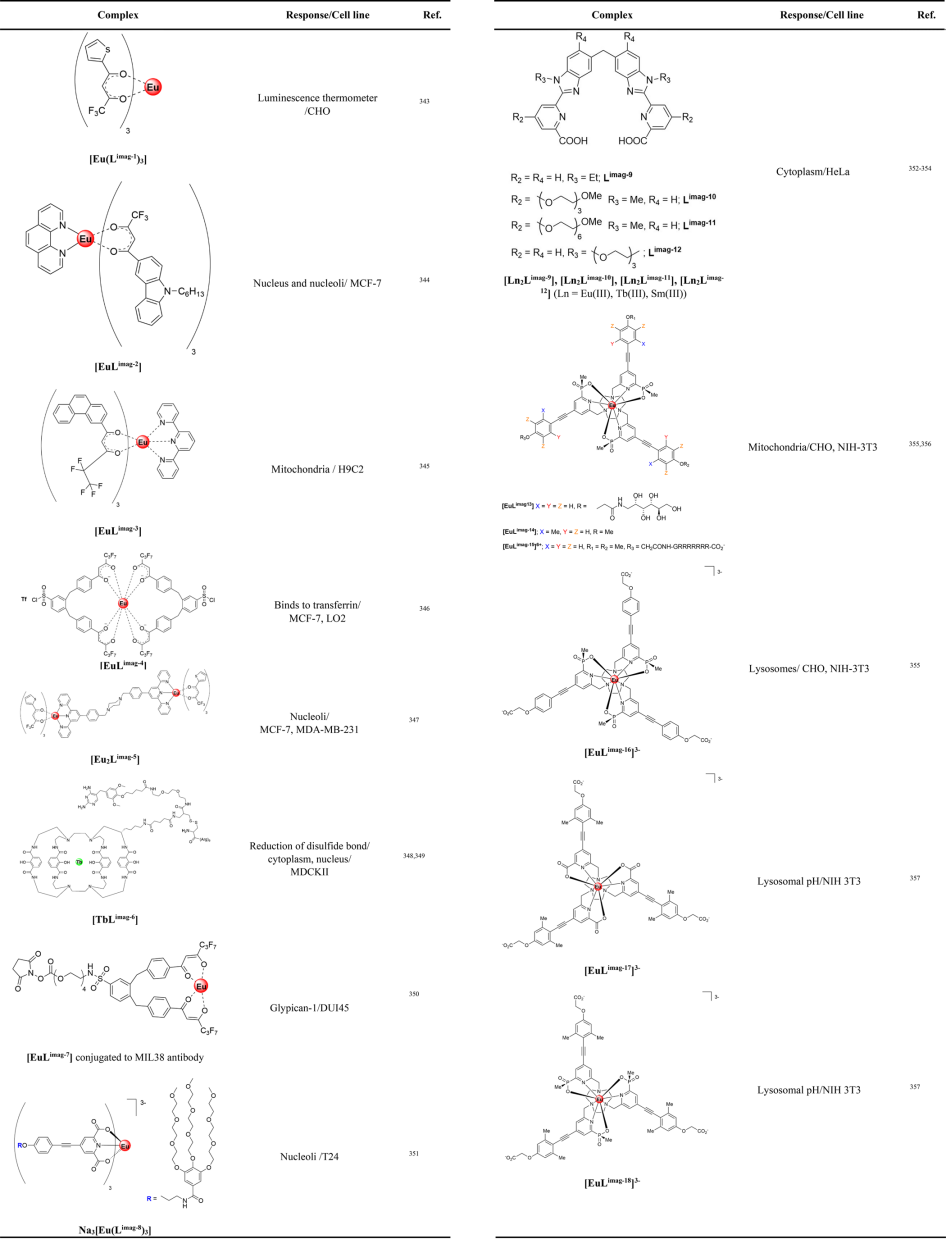

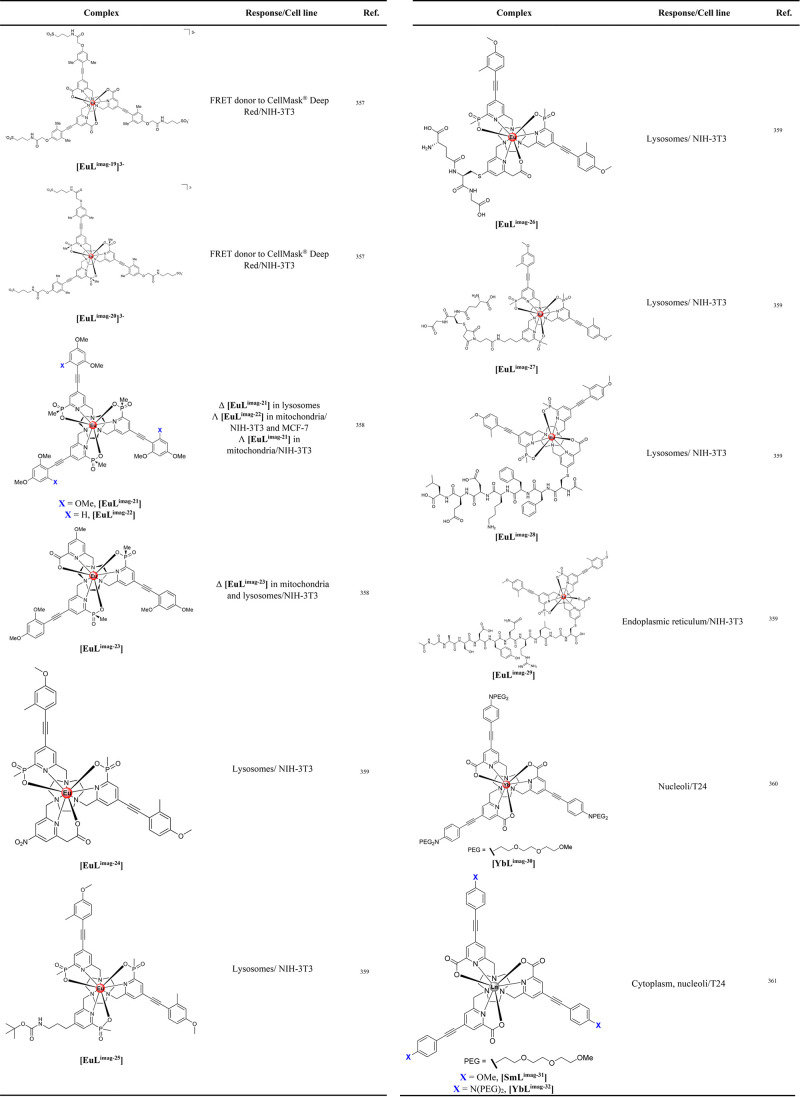

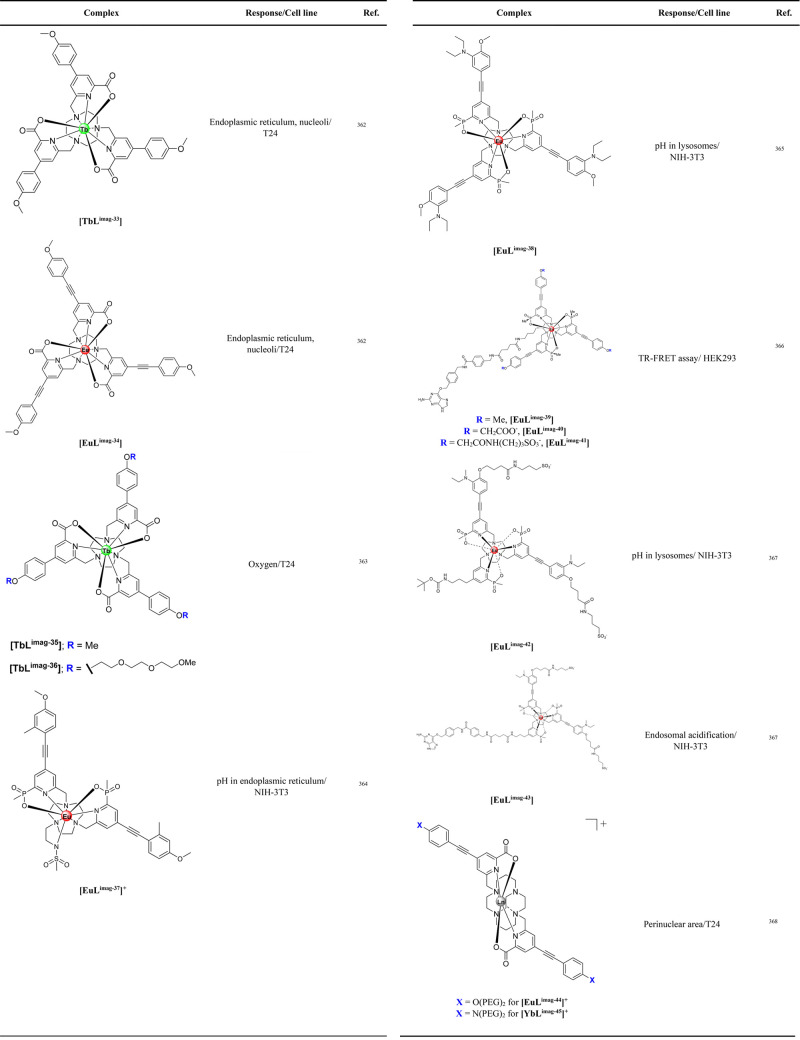

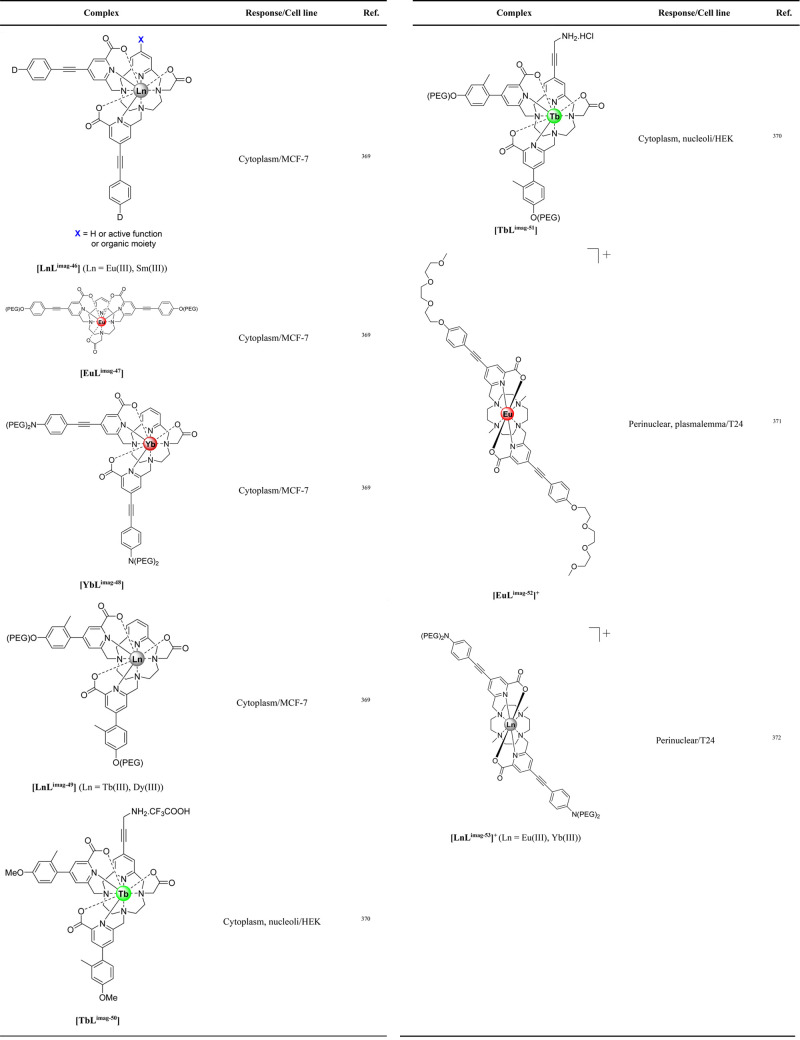

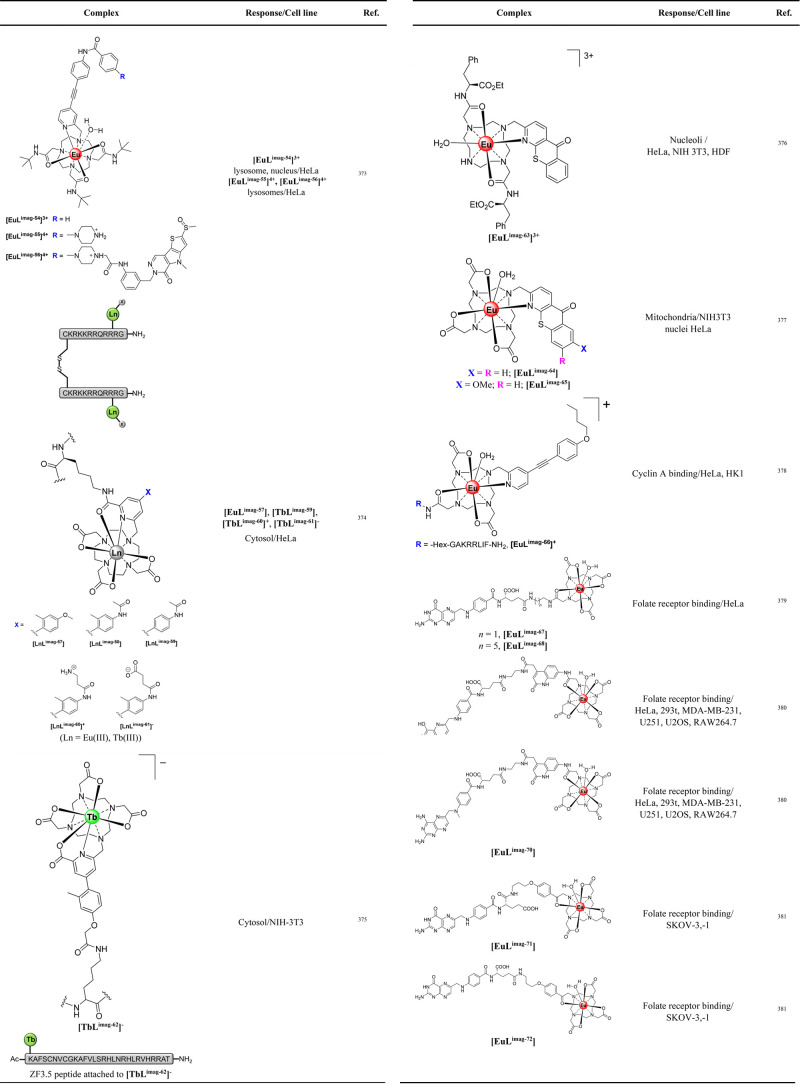

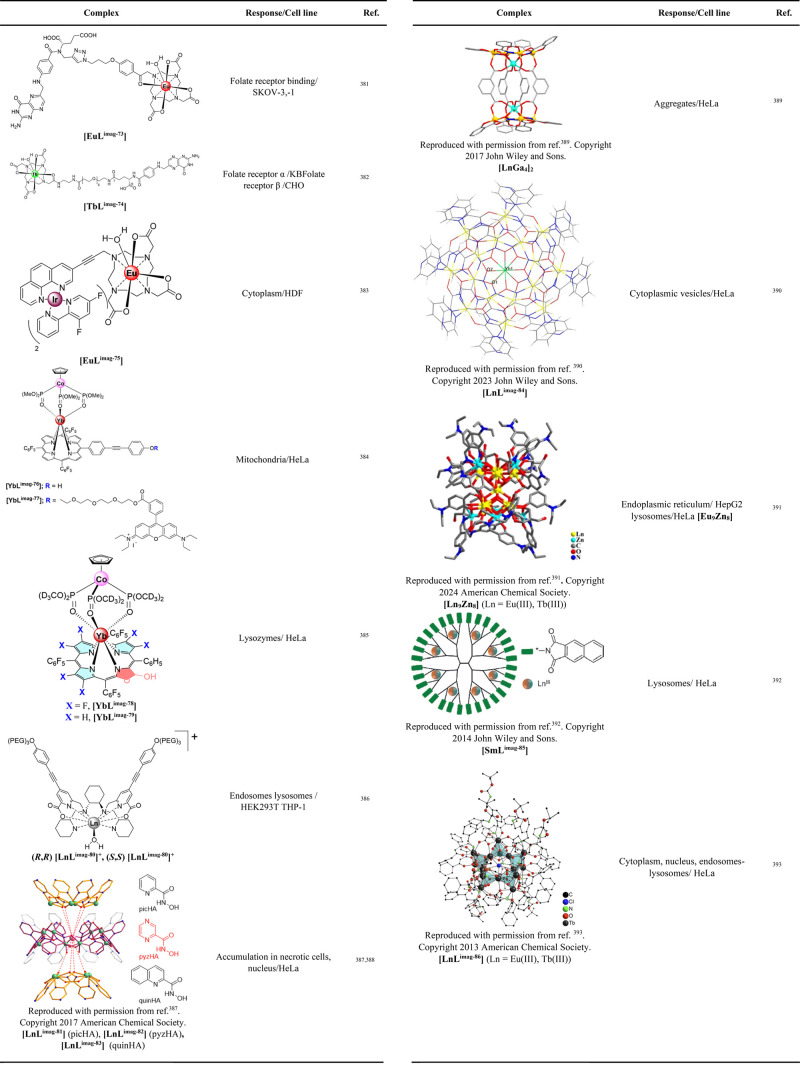

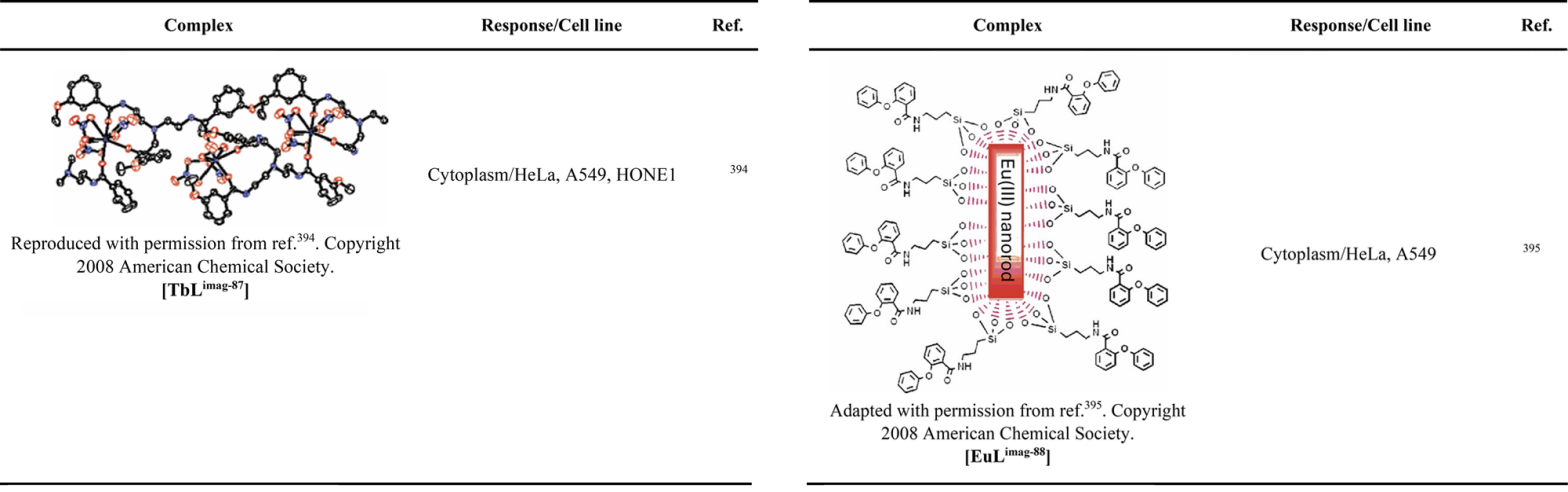
Luminescent Lanthanide Complexes for Cellular Imaging

### 3.1 β-Diketonates and Dipicolinates

Lanthanide diketonates are popular luminescent complexes, and their photophysical properties have been mainly elucidated for their incorporation in materials.^396,397^ β-Diketones provide a bidentate anionic binding site with a formation of a six-membered ring about lanthanide coordination which assists in the complex ability. There have been a few examples of complexes with the required solubility that are successfully incorporated in cellular imaging.

Europium(III) thenoyltrifluoro-acetonate, **[Eu(L**^**imag-1**^**)**_**3**_**]**, is a well-explored system for luminescence imaging, especially in its application as a luminescence thermometer. A thermal imaging method combining diffraction-limited spatial (300 nm) and sampling-rate-limited time resolution was developed.^398^ In the lifecycle of biological cells, enthalpy changes occur in intracellular chemical and biophysical events. While these enthalpy changes have been monitored by microcalorimetry, thermocouples, or pyroelectric films, these methods offer minimal spatial and temporal resolution. Thermal imaging was feasible using **[Eu(L**^**imag-1**^**)**_**3**_**]**, while the temperature dependent luminescence plays the role of a luminescence thermometer. Intracellular heat waves evoked in Chinese hamster ovary cells were monitored after activation of the metabotropic m1-muscarinic receptor by acetylcholine. It was found that Ca(II) was released simultaneously, suggesting that the m1-muscarinic signal transduction pathway was involved in the heat production.^398^ In tissue imaging, **[Eu(L**^**imag-1**^**)**_**3**_**]** enabled the differentiation between *n*-butyl-2-cyanoacrylate and Lipiodol, an oil in embolized vessels within the arteriovenous malformation model in swine.^343^

Wu, Tian et al. reported a carbazol β-diketonate derivative for accessing two-photon excitation in an Eu(III) complex with 1,10-phenanthroline as ancillary ligand, **[EuL**^**imag-2**^**]**. The two-photon cross-section at 720 nm was found to be 80 GM, and two-photon luminescence microscopy was used to its cellular uptake and localization characteristics in live cells.^344^ Confocal microscopy studies reveal that **[EuL**^**imag-2**^**]** acts as luminescent cellular DNA stain for human breast carcinoma MCF-7 cells. At high concentration of **[EuL**^**imag-2**^**]** (400 mM), most of the luminescence emerges from cellular cytoplasm, although punctate bright dots outside the nuclei region are attributed to aggregation of the complex inside the lysosome or mitochondria. Upon lowering the concentration to 200 mM, the punctate luminescence disappears, but the luminescence from the cell nucleus and nucleoli is clearly observed. No aggregation was observed in the cytosol at this concentration and hence the cell nucleus and nucleoli uptake the complex.^344^

Reddy et al. developed **[EuL**^**imag-3**^**]**, which exhibits quantum yield of 41% and a lifetime of 880 μs at pH = 7.4 in DMSO/PBS media.^345^ This complex was examined for live cell imaging using the rat embryonic heart cell line, H9c2.^345,399^ The ternary Eu(III) complex permeates into the H9c2 cells in 30 min and colocalizes with the mitochondria, as demonstrated by counterstaining experiments with commercial Mitotracker, CellLight.^399^ An advantage of the complex compared with the commercial mitochondria targeting dyes is its chemical stability for storage.^399^

Song et al. reported a β-diketonate complex, **[EuL**^**imag-4**^**]**, which covalently binds to proteins, resulting in long-lived and intense luminescence with Eu(III) analogue. It was tagged to transferrin biomacromolecule, forming **[EuL**^**imag-4**^**]** for it to bind with cancer cells overexpressing transferrin. Time-gated luminescence imaging allowed visualization of cultured cancerous cells in MCF-7 and LO2 (human hepatocyte) cell lines as well as the tumor-bearing H22 cells (mice hepatoma cell line) in the subcutaneous tissue of BALB/c nude mice.^346^*Ex vivo* tumor imaging from the mice exhibits clear images of the tumor sections in comparison with the transferring free complex, which suggests the specificity of **[EuL**^**imag-4**^**]** toward malignant tumors.^346^ In a separate work, triazine-based Eu(III) scaffold coordinated to three thenoyltrifluoroacetonate diketonates showed a high two photon absorption cross-section (320 GM).^400^ However, no specific localization profile was deducible when tested on HeLa cell lines.^400^

Patra et al. reported a binuclear Eu(III) complex where piperazine-4-methyl-phenyl terpyridine acts as a bridging ligand containing piperazine as a linker between two N3-donor phenyl-terpyridine ends and thenoyltrifluoroacetone acts as a capping antenna ligand, **[Eu**_**2**_**L**^**imag-5**^**]**.^347^ Cellular uptake studies in MCF-7 and MDA-MB-231 cell lines show localization in the nucleoli. Cellular uptake mechanism might involve endocytosis-based active transport pathways.^347^ A diethylenetriaminepentaacetic acid-based Eu(III) acyclic chelator with two morpholine units showed localization in the lysosome of MCF-7 and A498 cell lines.^401^

Raymond et al. identified a macrotricyclic ligand that features 2-hydroxyisophthalamide (IAM) chelating units, **Lumi4**. This octadentate ligand binds to Tb(III) **[TbLumi4]**, leading to very high quantum yields (Φ_total_ ≥ 50%), large extinction coefficients (ε_max_ ≥ 20,000 M^–1^ cm^–1^), and long luminescence lifetimes (τ_H2O_ ≥ 2.45 ms) at dilute concentrations in standard biological buffers.^402^ Miller et al. used **[TbLumi4]**, and a **[TbLumi4]** with trimethoprin, **[TbL**^**imag-6**^**]**, and coupled to cell penetrating peptides nonaarginine and HIV Tat-derived sequences mediated passive, cytoplasmic delivery in MDCKII cell line.^348^ Time-gated luminescence microscopic detection of Tb(III) luminescence or LRET between Tb(III) and a red fluorescent protein revealed that the cell penetrating peptide conjugates directly translocated from culture medium to the cytoplasm, and diffused freely throughout the cytoplasm and nucleus. In MDCKII cell line, **[TbL**^**imag-6**^**]** translocated directly into cells where its disulfide bond was reduced in the cytoplasm and nucleus.^348^**[TbL**^**imag-6**^**]** was further used to design and evaluate Tb(III) luminescence and Tb(III)-mediated FRET in cultured mammalian cells using a bespoke time-gated luminescence microscopy.^349^

Sayyadi et al. studied a series of tetradentate β-diketonate complexes^403^ and identified **[EuL**^**imag-7**^**]** with PEG chains and an NHS-ester.^350^ This highly emissive probe (Φ = 39%) was conjugated via lysine residues to MIL38, a mouse monoclonal antibody against Glypican 1, and used for the immunodetection of prostate cancer cells in DUI45 cell line. Other approaches to tagging **[EuL**^**imag-7**^**]** by using biotin–strepavidin conjugates were also explored. This indirect approach was less sensitive but had high selectivity. In comparison to conventional fluorescein isothiocyanate labeling of antibodies, **[EuL**^**imag-7**^**]** time-gated luminescence with a gated autosynchronous luminescence detector suppressed cellular autofluorescence background to allow high contrast images of immune-stained cancer cells.^350^

Dipicolinates provide a tridentate ligand framework and form anionic lanthanide complexes with a relatively high luminescent quantum yield. Andraud, Maury et al. reported a tris-dipicolinate Eu(III) complex **Na**_**3**_**[Eu(L**^**imag-8**^**)**_**3**_**]** with relatively high single photon quantum yield of Φ = 15.7%, long lifetime of τ_H2O_ = 1.062 ms, and a two-photon absorption cross-section at 700 nm of 92 GM. The three dipicolinates in the ligand framework fill in the lanthanide coordination sphere resulting in a negatively charged complex,^351^ and they are functionalized by an extended π-system, 3,4,5-tris(triethylene glycol)phenyl moieties to establish hydrophilicity. Two-photon luminescence microscopy experiments were carried out using T24 cancer cells. In comparison to a phase contrast image, it was indicated that **Na**_**3**_**[Eu(L**^**imag-8**^**)**_**3**_**]** is primarily localized in a perinuclear region and its distribution is like that of the endoplasmic reticulum. Additionally, bright spots were observed in the nucleus, indicating that the complex preferentially targeted nucleoli.^351^ However, it is noteworthy that lanthanide dipicolinate-based systems dissociate with increasing dilution. The dissociation is expected to be about 30% in 10 to 50 μM concentration of the complex.^404^

A powerful application showing the potential of the long luminescence lifetime monitoring in cellular imaging^351^ was demonstrated with the participation of **Na**_**3**_**[Eu(L**^**imag-8**^**)**_**3**_**]** in cellular FRET processes by precise detection of the long luminescence lifetimes.^405^ Fine variations of the luminescence lifetime of **Na**_**3**_**[Eu(L**^**imag-8**^**)**_**3**_**]** were revealed and mapped in cells in the presence of a heptamethine based FRET acceptors, allowing quantification of the FRET efficiency independently of donor concentration. These studies show the potential of lanthanide luminescence in monitoring biosensing events by FRET *in cellulo* and potentially *in vivo* settings. A few years later, Nagano et al. reported time-resolved, long-lived luminescence microscopy for the luminescence imaging of lanthanides in cells.^406^

### 3.2 Helicates

A class of ligands based on dipicolinates and bis(benzimidazole)pyridines have been found to self-assemble in solution by coiling around Ln(III) ions to afford nine-coordinate helicate complexes in which the ligand strands are also held together by weak intramolecular π–π interactions.^407^ Such helicates have been widely studied as probes in circularly polarized luminescence studies. Bünzli et al. tested an array of dinuclear Ln(III) helicates, composed of hydrophilic hexadentate ditopic ligands of dipicolinate core bearing two benzimidazole antenna units for cellular imaging. The interactions between the ligands and different lanthanides have been well studied spectroscopically,^408,409^ indicating that at physiological pH, a 2:3 lanthanide:ligand species dominates in aqueous solution, the proportion of which is larger than 97% for **[Ln**_**2**_**L**^**imag-9**^**]** and **[Ln**_**2**_**L**^**imag-10**^**]**, while it is substantially smaller (∼90%) for the benzimidazole-substituted ligand **[Ln**_**2**_**L**^**imag-11**^**]**. These helicates are found to be stable in solution with log β_23_^Ln^ values ranging from 23 to 28. The stability of these helicates is not influenced along the lanthanide series, which allows a range of Ln(III) metals for photophysical studies without significantly affecting its speciation.^352,407,409^

Among the helicates reported, ligand **L**^**imag-10**^ proves to be the best sensitizer for Eu(III) (Φ = 19.8 ± 0.7% **[Ln**_**2**_**L**^**imag-10**^**]**; ε = 85,000 M^–1^ cm^–1^), although it also sensitizes Tb(III), Sm(III), and Yb(III).^409^ Cytotoxic profiles for the helicates reveal half inhibitory concentration of IC_50_ > 500 μM. Lactase dehydrogenase test was performed to assess the potential damages caused to the cells by the probe of interest with leakage found to be <4% in Jurkat (human T leukaema), 5D10 (mouse hybridoma), and MCF7 cells in the case of treatment with **[Ln**_**2**_**L**^**imag-12**^**]**.^352,409^ Upon incubating the helical probes **[Ln**_**2**_**L**^**imag-10**^**]** and **[Ln**_**2**_**L**^**imag-11**^**]** (∼250 μM) in HeLa cells, the time course and temperature-dependence of the loading were determined.^352−354^ The first bright spots in the cytoplasm of the cells can be observed for 15 min. No Eu(III) luminescence was observed when the complex was loaded at 4–8 °C, attributing endocytosis for the helicate uptake in cells. The proposed mechanism of uptake is verified with endocytosis inhibitors.^354^

In order to determine the localization of the helicates within the cytoplasm of the live cells, colocalization experiments with organic stains were used.

### 3.3 Macrocyclic Complexes

#### 3.3.1 TACN-Based Complexes

**Figure 14:**
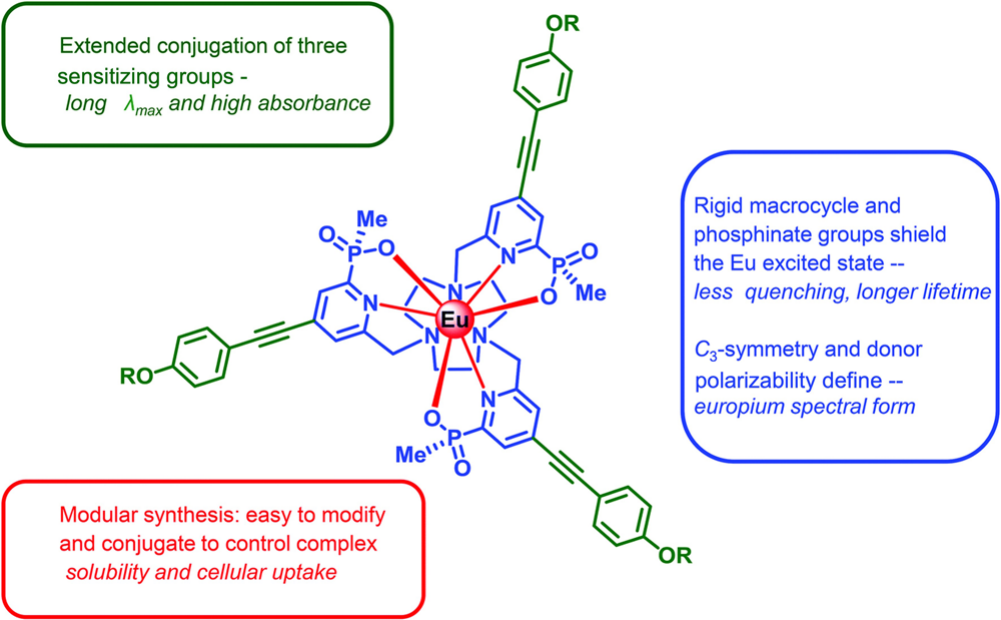
Essential design elements and structural features in emissive **[Eu(TACN)]** probe design from the Parker group. Reproduced with permission from ref (356). Copyright 2015 Royal Society of Chemistry.

TACN (TACN = 1,4,7-triazacyclononane) is a macrocycle of 9-ane-N_3_ framework with secondary amines available for functionalization to pride additional coordination. This framework is attractive compared to other macrocycles due to the lack of N–H bonds, reducing detrimental N–H oscillator quenching of lanthanide luminescence. The lanthanide is positioned above the plane of the macrocycle supported by the coordinating arms of the macrocycle. Pyridylalkyl antenna arms have been extensively employed in 9-ane-N_3_ macrocycles for lanthanide luminescent complexes in cellular imaging. Parker et al. reported EuroTracker dyes which are bright Eu(III) complexes of *C*_3_ symmetry, capable of staining different regions of the cell selectively (Figure 14).^355,356^ Their exceptional brightness is attributed to several factors including: the very high extinction coefficient associated with the intraligand charge transfer transition, the efficient intramolecular energy transfer process and the effective shielding of the bound Eu(III) ion from vibrational deactivation.^355^ Several derivatives of this complex framework were explored by varying charge, lipophilicity, and bulkiness of the complexes.^291^

The neutral complex **[EuL**^**imag-13**^**]** (18 μM) was incubated in Chinese Hamster Ovarian (CHO) cell line and mouse-skin fibroblasts (NIH-3T3).^355^ Rapid uptake of this complex was observed over a period of 48 h with no inhibition to cellular proliferation (IC_50_ > 100 μM, 24 h), leading to localization in mitochondria, verified by MitoTracker green. Selective staining of **[EuL**^**imag-13**^**]** to the mitochondria is primarily associated with the amphipathic nature of the complex, and the excellent tolerance may be attributed, in part, to the presence of the peripheral glucamide substituents. The selective mitochondrial localization could be tentatively attributed to local phosphorylation of the sugar primary hydroxyl groups, leading to an anionic complex that only slowly escapes the mitochondrial organelle. A similar localization profile was observed with **[EuL**^**imag-14**^**]**, however slower than **[EuL**^**imag-13**^**]** with 40% emission intensity enhancement between 4 and 24 h incubation period. An inductively coupled plasma mass spectrometry (ICP-MS) study of NIH-3T3 cell lines revealed that for 4 × 10^6^ NIH-3T3 cells incubated with **[EuL**^**imag-13**^**]** (18 μM), each cell contained 34 μM of Eu at 24 h, substantially higher than that of **[EuL**^**imag-14**^**]** (6 μM) under the same conditions.^355^

Anionic **[EuL**^**imag-16**^**]**^**3–**^ (30 μM) was rapidly internalized in CHO and NIH-3T3 cells. Microscopy images taken after 30 min (up to 12 h) revealed localization preference within the lysosomes and colocalized using LysoTracker Green. In this case, the negative charge of the complex enhanced hydrophilicity and localization in the lysosomes. Cationic polyarginine complex **[EuL**^**imag-15**^**]**^**6+**^ (10 μM) was found to be localized on the mitochondria of NIH-3T3 and CHO cells with subsequent tracking to the endoplasmic reticulum over time (12–24 h). In order to understand the mechanism of cell uptake, the behavior of these complexes was examined in the presence of known inhibitors and promoters of defined cell-uptake pathways. In each case, the extent of intracellular uptake was evaluated by comparing the observed image intensity using laser scanning confocal microscopy and the amount of Eu(III) in cell populations using ICP-MS. The brightness of these Eu(III) complexes is about the same as the “red-fluorescent protein”, mCherry, which emits at 610 nm (λ_ex_ = 587 nm) with an extinction coefficient of 72 mM^–1^ cm^–1^ and a fluorescence quantum yield of 22%.^355^

Parker et al. investigated the anionic Eu(III) complexes for lifetime measurements of lysosomal pH. Incubation of **[EuL**^**imag-17**^**]**^**3–**^ and **[EuL**^**imag-18**^**]**^**3–**^ for 2 h in NIH-3T3 cell lines revealed internalization and gave rise to a punctate staining pattern.^357^ Parallel incubations with LysoTracker Green confirm the selective lysosomal staining pattern. However, the sulfonate version of the complexes, **[EuL**^**imag-19**^**]**^**3–**^ and **[EuL**^**imag-20**^**]**^**3–**^, showed no cellular uptake and internalization, as the complexes are observed in the growth medium surrounding the cells by microscopy. These sulfonated Eu(III) complexes served as a FRET donor to Cell Mask Deep Red, a membrane staining dye.^357^ Time-resolved FRET microscopy was undertaken to observe the probe localization. Energy transfer between **[EuL**^**imag-20**^**]**^**3–**^ as the FRET donor and the membrane-immobilized dye acceptor can only occur in the vicinity of the membrane, so the clarity of the FRET image is quite superior to that obtained examining dye fluorescence only.^357^

**Figure 15:**
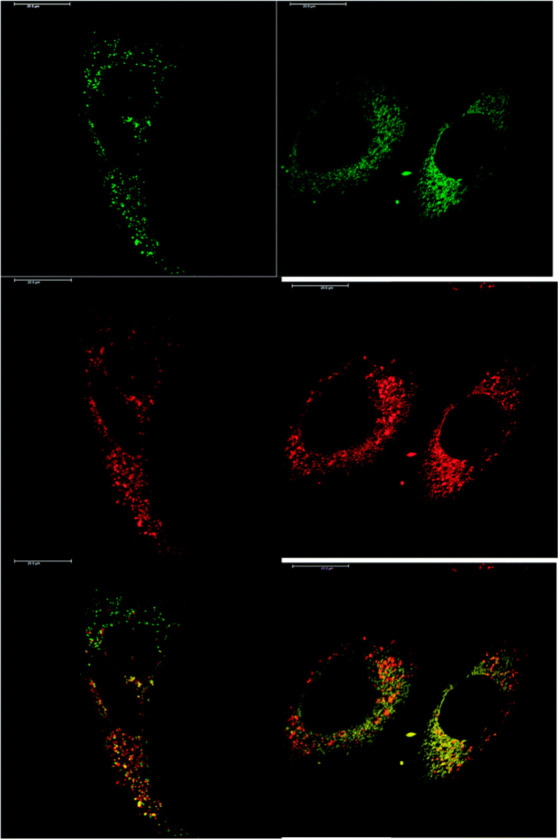
Laser scanning confocal microscopy images of NIH-3T3 cells treated with Λ-**[EuL**^**imag-22**^**]** (right) and Δ-**[EuL**^**imag-22**^**]** (left) showing the predominant mitochondrial (*P* = 0.71) and lysosomal (*P* = 0.77) localization profiles, respectively (red for Eu(III) emission, green for Mitotracker Green and LysoTracker Green). Scale bar = 20 μm. Reproduced with permission from ref (358). Copyright 2018 Royal Society of Chemistry.

Parker et al. studied the impact of chirality on the cellular uptake of Eu(III) complexes.^358^ Although the racemic complex **[EuL**^**imag-22**^**]** was studied for cellular uptake, its mechanism of internalization remained unknown. This was investigated alongside the uptake and subcellular localization of the enantiomers **[EuL**^**imag-21**^**]**, **[EuL**^**imag-22**^**]**, and **[EuL**^**imag-23**^**]**. Racemic **[EuL**^**imag-22**^**]** and resolved enantiomers of **[EuL**^**imag-22**^**]** were studied for cellular uptake and localization (Figure 15), while enantiomers of **[EuL**^**imag-21**^**]** were studied in parallel to explore if chromophores influence the uptake or localization profile.^358^ It was shown that **[EuL**^**imag-22**^**]** was taken up by the cells through macropinocytosis using various inhibitors and promoters of macropinocytosis (amiloride, wortmannin, phorbol ester, Di-Rac), clathrin-mediated endocytosis (chlorpromazine, sucrose), caveolin-dependent endocytosis (filipin), and inhibitors of the maturation of endosomes to lysosomes (chloroquine, monensin).^342,358^ Macropinocytosis is a nonspecific mechanism of internalization which cannot preclude the possibility of chirality-dependent cell uptake and subcellular localization, especially when proteins which are chirally sensitive involve in transport of these complexes within cells. It is possible that different subcellular localizations may be present for different enantiomers.

Cell uptake with enantiopure Δ- and Λ-**[EuL**^**imag-21**^**]**, **[EuL**^**imag-22**^**]**, and **[EuL**^**imag-23**^**]** was performed with NIH-3T3 and MCF-7 cell lines. In enantiopure Δ- and Λ-**[EuL**^**imag-22**^**]**, no significant changes in localization profiles were observed after 24 h of incubation. However, the emission brightness of Λ-**[EuL**^**imag-22**^**]** was higher than that of Δ-**[EuL**^**imag-22**^**]** by a factor of 2 in each cell line. The subcellular distribution of the Λ- and Δ-enantiomers of **[EuL**^**imag-22**^**]**, **[EuL**^**imag-21**^**]**, and **[EuL**^**imag-23**^**]** was studied using confocal microscopy and organelle-specific stains. Λ-Enantiomers accumulated preferentially in mitochondria, while the Δ-enantiomers were found in lysosomes. This difference in subcellular localization is due to a faster relocalization of the Δ-enantiomers to the lysosomes. This time-dependent localization behavior was reported in racemic complexes bearing pyridyl/aryl/alkynyl chromophores and azaxanthones.^356,410^ It is noteworthy that phorbol ester, which promotes macropinocytosis, quenches **[EuL**^**imag-22**^**]** luminescence. This observation has a potentially misleading effect on using the brightness of the given complex to assess the extent of cellular uptake. As a consequence, a need has arisen for robust methods to correlate change in luminescence with respect to the change in cell uptake by the complexes. These observations are best supported by nonluminescence methods to account for the uptake of complexes by the different components of a cell.^342,358^

Another strategy to enable cell permeation and selective staining of organelles is to tag a luminescent complex onto cell penetrating peptides. However, polycationic peptides have been shown to promote cell toxicity, as the peptide inserts into the lipid bilayer and the cell membrane may then be permeabilized rather dramatically.^411^ Parker et al. investigated a series of Eurotracker-type complexes conjugated on to endoplasmic reticulum targeting peptide KDEL^412^ or the trans golgi targeting peptide SDYQRL^413,414^ using different strategies and exploring the potential of these complexes to stain cells.^359^ Cellular localization of these complexes was studied using NIH-3T3 cells. Complexes **[EuL**^**imag-24**^**]**, **[EuL**^**imag-25**^**]**, and glutathione conjugates **[EuL**^**imag-26**^**]** and **[EuL**^**imag-27**^**]** stain lysosome as confirmed by costaining experiments with Lysotracker green. **[EuL**^**imag-28**^**]** with the peptide AcCFFKDEL was found to stain the endoplasmic reticulum as its staining was observed slowly from 3 to 23 h, resulting in increased luminescence intensity. **[EuL**^**imag-29**^**]** with AcGASDYQRLGC was supposed to target trans golgi network but was found in the lysosomes. Macropinocytosis is the likely mode of cellular uptake of these complexes in the cells.^359^

Maury et al. reported for the first time metal-centered two-photon NIR imaging of lanthanide complexes in cells. This was achieved using Yb(III) complexes of dipicolinic acid **Na**_**3**_**[Yb(L**^**imag-8**^**)**_**3**_**]**^**3–**^ (τ_H2O_ = 0.34 μs) as a control and kinetically inert macrocycle substituted with dipicolinate ligand, **[YbL**^**imag-30**^**]** (τ_H2O_ = 3 μs), incubated in T24 human cancer cells.^360^ One-photon microscopy image reveals successful staining of the complex on the cell with a preferential localization in the nucleoli as observed in related Eu(III) complexes. The two-photon cross-section was estimated to be in the same range with that of Eu(III) and Lu(III) analogues, determined to 775 and 500 GM, respectively.^360^

Conventional two-photon microscopes proved challenging for detection in NIR due to poor signal-to-noise ratio, and a lack of optical filtering schemes to allow both λ_ex_ and λ_det_ in the NIR range. Therefore, a bespoke biphotonic microscopy with adequate optical filtering was developed to test with **[YbL**^**imag-30**^**]**. After validation experiments, the mouse brain vascular network was successfully imaged in depth with both λ_ex_ = 760 nm and λ_det_ = 1000 nm in the biological transparency window. This proof-of-concept experiment opened an interesting perspective for the development of thick tissue imaging toward biologically relevant applications (for diagnostic or guided surgery).^360^

In a separate work, Maury et al. investigated a Sm(III) complex bearing picolinate-alkynyl arms **[SmL**^**imag-31**^**]** and compared it against hydrophilic Yb(III) complex of picolinate-alkynyl arms modified with polyethylene glycol (PEG) chains **[YbL**^**imag-32**^**]** (τ_MeOH_ = 3 μs) by two-photon microscopy.^361^ Owing to the hydrophobicity of **[SmL**^**imag-31**^**]**, living T24 cells were stained with a solution of the complex in 10% DMSO. The complex was internalized, but the participation of DMSO to membrane permeabilization cannot be excluded. Under two-photon excitation, high-contrast images were obtained with NIR-to-visible configuration in the microscope.^361^**[SmL**^**imag-31**^**]** stained lipophilic parts of the cells such as organelles in the cytoplasm, and to some extent, membranes and diffused labeling of nuclei, with a more intense accumulation in the nucleoli. The contrast and resolution of these images were comparable with the more emissive Eu(III) or Tb(III) complexes. The signal-to-noise ratio in the images obtained in the case of NIR excitation-NIR detection for **[YbL**^**imag-32**^**]** was similar to the one obtained from **[SmL**^**imag-31**^**]** with an identical distribution. These results proved that Sm(III) can be tailored for use in two-photon NIR and visible imaging by the same complex, despite its poor photophysical properties (Φ = 0.91%).^361^

In the quest to combine the photophysical properties of lanthanide complexes and the intrinsic advantage of a nonlinear two-photon excitation, a biphotonic multiplexing bioimaging experiment was envisioned.^362^ A Tb(III) luminescent bioprobe containing tris-picolinate-biaryl arm containing an electron donating methoxy group on a TACN framework was investigated **[TbL**^**imag-33**^**]** and explored along with the reported Eu(III) TACN complex **[EuL**^**imag-34**^**]**. The quantum yield and lifetime of **[TbL**^**imag-33**^**]** remain rather modest (Φ = 12%, τ = 0.24 ms) compared to related nonsubstituted complex.^415^ The two probes clearly stained the endoplasmic reticulum and the nucleosol with a higher concentration of **[TbL**^**imag-33**^**]** in the nucleoli. The emission spectra were monitored *in cellulo* using the spectral detection mode of the microscope.^362^ The cells were stained with a mixture of **[EuL**^**imag-34**^**]** and **[TbL**^**imag-33**^**]**, and the two photon-images were deconvoluted.^362^ This combined detection associated with specific functions for the targeting of different cell-compartments would open the way for a high-content multiparameter multiplexed labeling.^362^

Cells and biological tissues are surrounded in a complex medium which require understanding on the study of its physical properties. Mobility of species is a factor of paramount importance governing numerous cellular processes.^416^ Changes in diffusion behavior are closely related to the development of diseases,^417^ which places a need to develop fluorescent tools that respond to the viscosity of the environment.^419,420^ Bui, Grichine, Maury et al. explored the luminescent **[TbL**^**imag-36**^**]** as a probe for sensing viscosity.^363^**[TbL**^**imag-36**^**]** (Φ = 15%, τ = 0.23 ms in MeOH) was reported for two-photon multiplex imaging but has poor photophysical properties compared to its parent nonfunctionalized **[TbL**^**imag-35**^**]** (Φ = 60%, τ = 2 ms in tris buffer),^415^ as the aryl–aryl rotations of the antennae constitute a source of nonradiative relaxation.^362^ These features were exploited to investigate the potency of these complexes toward viscosity responses.^363^**[TbL**^**imag-36**^**]** displayed a strong enhancement of luminescence intensity in viscous media, leading to an increase in quantum yield from 15% in MeOH to 67% in glycerol. Studies of the nonluminescent **[GdL**^**imag-36**^**]** suggest that there are ligand energy levels low enough for the thermal repopulation of the triplet state from the terbium-centered excited state to occur.^418^ The Eu(III) analogue, which has much higher energy gap, showed no conspicuous change in its luminescence properties correlated to solvent viscosity, giving evidence that reducing energy back transfer to the ligand prevents viscosity-dependent deactivation pathways. Consequently, by allowing ligand-induced relaxation for the duration of the complex excited-state lifetime, the reversibility of the energy transfer to Tb(III) constitutes a key feature of this photophysical mechanism leading to sensitivity for viscosity.^372^ In air-saturated media, methanol and glycerol **[TbL**^**imag-36**^**]** shows very different lifetime values, which highlights the role of oxygen concentration in **[TbL**^**imag-36**^**]** rather than viscosity.^363^

Parker et al. reported an Eu(III) TACN derived complex with two pyridyl-alkynyl-aryl based sensitizers and a methyl substituted sulfonamide **[EuL**^**imag-37**^**]**^+^.^364^ Cellular localization studies in NIH-3T3 cells reveal permeability in the endoplasmic reticulum, verified by colocalization studies with ER-tracker green. **[EuL**^**imag-37**^**]^+^** was pH-responsive due to the deprotonation of the sulfonamide amine proton, resulting in its coordination to the Eu(III) center (p*K*_a_ of **[EuL**^**imag-37**^**]**^**+**^ is 6.52 ± 0.03, determined ratiometrically). Information gained on pH changes (from 7.7 to 6.0) of the endoplasmic reticulum using **[EuL**^**imag-37**^**]**^**+**^ provided information on overall cytosolic pH in NIH-3T3 cells.^421^ From Eu(III) luminescence, by ratiometrically plotting Δ*J* = 2/(Δ*J* = 0 + Δ*J* = 1) against pH, a near linear response was observed between the pH 6.7 and 7.4, with an overall 40% change in ratio. Changes in the excited state lifetimes were also observed, with values of 672 and 413 ms recorded at pH 6.7 and pH 7.4, respectively. This 60% increase in the lifetime upon lowering the pH within the endoplasmic reticulum suggests that changes in the structural form of the complex when the sulfonamide moiety is unbound may be affecting certain protein–complex interactions, resulting in longer lifetime values due to a reduction in the quenching process.^364^ Cellular changes in pH are important feature of the aging process of endosomes and phagosomes (endosomal pH ranges from 6.5 to 5.5, mature lysosomes around 4.5).^422^ The processes of internalization and endosomal uptake can therefore be followed in time if the species being internalized (receptor or substrate) is for instance labeled with a luminescent pH-sensitive dye whose emission intensity or lifetime varies with pH.^423^ Ratiometric systems offer reliable pH changes over unduly celebrated switch-on sensors which possess modest emission intensity variations.^365^ Cellular uptake and colocalization studies with **[EuL**^**imag-38**^**]** in NIH-3T3 and MCF-7 cell lines ascertain localization in the lysosomes. Upon the addition of nitroglycerin to NIH-3T3 incubated **[EuL**^**imag-38**^**]** (lysosomal pH changes from 4.5 to 6.5), a decrease in the emission intensity of **[EuL**^**imag-38**^**]** was observed.^365^

**Figure 16:**
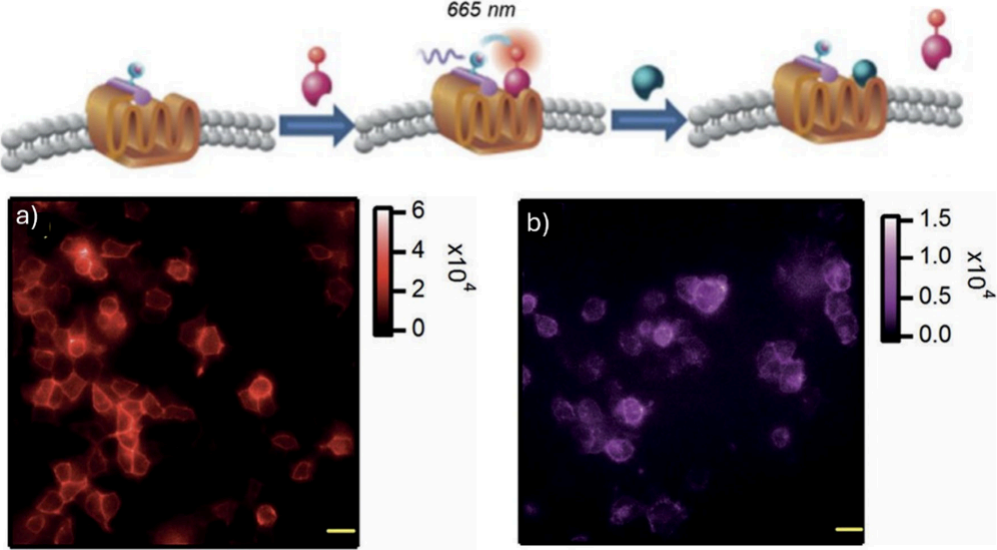
(top) Illustration of the consecutive binding of an IR-dye labeled agonist acceptor to the Eu-SNAP-tagged cholecystokinin-2 cell surface receptor followed by competitive displacement of the agonist by an added antagonist; (bottom) (a) Time-resolved luminescence microscopy image of SNAP cholecystokinin-2 receptor-transfected HEK293 cells labeled with **[EuL**^**imag-41**^**]**. (b) Time-resolved FRET channel image after adding red- cholecystokinin-2 receptor (26–33) dye to transfected HEK293 cells labeled with **[EuL**^**imag-41**^**]** to reveal emission from the dye and quenching of Eu(III) luminescence. Adapted with permission from ref (366). Copyright 2014 John Wiley and Sons.

Complexes that stain intracellular components of a cell are a disadvantage to develop assays for G-protein coupled receptors. This was circumvented by tailoring a Eu(III) TACN complex with para-substituted aryl-alkynyl sensitizer bearing sulfonate or carboxylate groups.^366^ The anionic nature of the resulting complex was expected to decrease interactions with the cell membrane because of repulsive Coulombic interactions. The complexes were tagged on to benzylguanine derivatives to form **[EuL**^**imag-39**^**]**, **[EuL**^**imag-40**^**]**, and **[EuL**^**imag-41**^**]**:benzylguanine labels SNAP-tag, a self-labeling suicide enzyme.^424^ The complexes were evaluated using SNAP-tag technology^425^ on the cholecystokinin-2 receptor (CCK-2), a G-protein-coupled receptor which has elevated expression levels when transiently transfected in HEK293 cells. The labeling of **[EuL**^**imag-39**^**]**, **[EuL**^**imag-40**^**]**, and **[EuL**^**imag-41**^**]** on living HEK293 cells or HEK293 cells expressing the SNAP tagged CCK-2 was measured by monitoring the time-gated luminescence intensity. **[EuL**^**imag-39**^**]** showed significant labeling in HEK293 cells. Time-resolved Förster resonance energy transfer was studied between **[EuL**^**imag-41**^**]**-labeled living SNAP-CCK2 cells and a red-fluorescing agonist of CCK-2, red-CCK(26–33) (λ = 665 nm).^426^ Binding of the natural agonist linked to an acceptor IR dye was signaled by dynamic quenching of Eu(III) luminescence and an increase in the long-lived luminescence (λ = 665 nm) of the acceptor dye, red-CCK(26–33). When the agonist (CCK-2) was displaced by competitive binding of a candidate antagonist (PD135158) to the receptor binding site, the FRET signal was lost and the Eu(III) emission intensity and lifetime increased (Figure 16). Such a system allows the affinity of competitive antagonists to be assessed in high throughput formats or in live cell assays using time-resolved microscopy.^2,366^

To minimize nonspecific binding to proteins, sulfonamide groups were added to the two pyridyl-alkynyl-aryl chromophores. The third arm on the TACN core has a pyridine group functionalized with a primary amino-propyl group (N-Boc protected) to permit subsequent site-selective conjugation to different targeting vectors. These incorporations led to the formation of **[EuL**^**imag-42**^**]**.^367^ Up to 50-fold increase in luminescence was observed from pH 8 to 4. Monitoring changes in emission lifetime or luminescence allowed p*K*_a_ values to be estimated: the p*K*_a_ in NIH-3T3 cell lysate was 6.00 ± 0.04 for **[EuL**^**imag-42**^**]**. This p*K*_a_ value was sensitive to the nature of the medium and was reduced slightly in cell lysate solution, highlighting the need to calibrate the pH-dependence in the medium of interest. The glucagon-like peptide-1 receptor is targeted for antidiabetic drugs due to its involvement in the metabolic pathway for insulin production. Therefore, **[EuL**^**imag-42**^**]** was tagged with a benzylguanine derivative, forming **[EuL**^**imag-43**^**]**:benzylguanine labels SNAP-tag, a self-labeling suicide enzyme.^424^**[EuL**^**imag-43**^**]** was used to label the transfected HEK-293 cell line. Increased Eu(III) luminescence response over HEK-293 cell line proved labeling of **[EuL**^**imag-43**^**]** on the cell surface of the transfected HEK-293 cell line. Glucagon-like peptide-1 receptor agonist Exendin-4 was added to **[EuL**^**imag-43**^**]** labeled receptors at pH 7.4 to observe a 5-fold increase in luminescence intensity due to receptor internalization and subsequent endosomal acidification.^367^ The increases in quantum yield and brightness following acidification are due to a strongly emissive and longer-lived protonated complex, wherein the photoinduced electron transfer process that quenches the europium excited state was suppressed.^367^

#### 3.3.2 Cyclam-Based Complexes

Cyclam, 1,4,8,11-tetraazacyclotetradecane, is a macrocyclic ligand of type [14]aneN_4_, which was first reported in 1937^427^ and investigated for its affinity toward Ni(II).^428^ It can complex various cations including transition metals, often with very high thermodynamic and kinetic stability with respect to metal ion dissociation.^429^ Cross-bridged tetraaza macrocyclic ligands based on cyclam typically contain a linker connecting two *trans* nitrogen atoms of the macrocycle using ethylene or propylene groups or linkers integrating additional donor groups, which results in constrained positions of the four nitrogen lone pairs of the macrocyclic cavity.^430^ The presence of this linker increases the rigidity of the corresponding metal complexes, which as a result are often very inert with respect to complex dissociation.^431^*Trans*-dipicolinate cyclam macrocycle cross-bridged by ethylene group exhibit exceptional and unprecedented inertness toward Ln(III), forming **[Ln(cb-tedpa)]**.^432^ This unprecedented stability relies on a *cis-V* geometry, leading to a very strong interaction between the macrocyclic nitrogen donor atoms of the macrocycle and the metal ion.^432^

Using **[Ln(cb-tedpa)]**, Tripier, Maury et al. investigated its potential for two-photon excitation by extending its dipicolinates with donor π conjugated antenna, forming **[EuL**^**imag-44**^**]**^**+**^ (**L**^**imag-44**^ has alkoxyphenylethynyl electron donating group) and **[YbL**^**imag-45**^**]**^**+**^ (**L**^**imag-45**^ with a dialkylaminephenylethynyl group).^368^ The presence of electron donating groups improves hydrophilicity and lanthanide luminescence in the corresponding metals. Photophysical properties of **[EuL**^**imag-44**^**]**^**+**^ and **[YbL**^**imag-45**^**]**^**+**^ reveal that these complexes have poor quantum yield in water in comparison to other polydentate complexes. **[EuL**^**imag-44**^**]**^**+**^ and **[YbL**^**imag-45**^**]**^**+**^ possess charge transfer antennas responsible for the two-photon absorption properties, similar to their diMe-cyclen counterparts, and can therefore be assumed to present similar two photon cross-sections. These complexes were tested in bioimaging experiments using living T24 cells or fixed HeLa cells. The quality of the images obtained using **[EuL**^**imag-44**^**]**^**+**^ was excellent with a high contrast and high signal-to-noise. In addition, for **[YbL**^**imag-45**^**]**^**+**^, NIR-to-vis and NIR-to-NIR images were also recorded. In the case of living cells, the images clearly indicate the spontaneous internalization of the complexes and localized in the perinuclear area of the cytosol. The circular and filamentous organelles can be observed by accumulation of the dyes in endocytic vesicles and mitochondria.^368^ The presence of such stained vesicles indicates that the passive internalization mechanism occurs via endocytosis or pinocytosis or possible passive transmembrane diffusion of luminescent probes.^368^

#### 3.3.3 Pyclen-Based Complexes

Pyclen (1,4,7,10-tetraaza-2,6-pyridinophane)^433^ is a macrocyclic ligand which contains a pyridyl ring fused onto cyclen.^434^ The stability of this macrocycle bearing carboxylate arms toward transition metals^435^ and Ln(III) ions has been extensively studied to conclude the favorability of this ligand toward Ln(III) both in terms of dissociation kinetics and thermodynamic stability.^436^ Pyclen offers a more rigid structure that may impose unusual coordination environments or provide unusual redox behavior.^437^ However, the *sp*^2^ character of the aromatic nitrogen atom implies the unavailability of a donor atom of the macrocycle for a fourth *N*-functionalization. Despite this, pyclen complexes have been explored as potential radiopharmaceuticals^438^ and theranostic^439^ agents.

**Figure 17:**
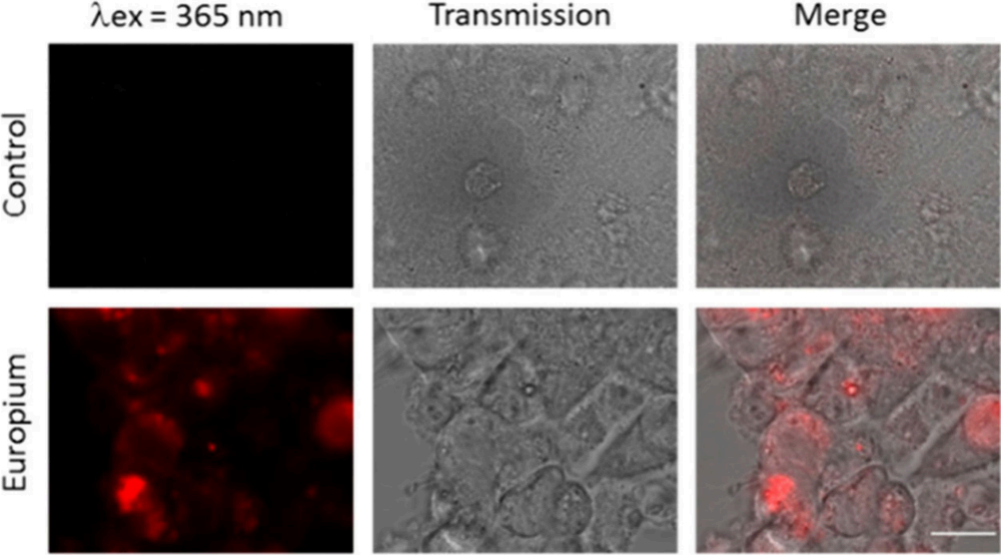
Fluorescence imaging of human breast cancer cells (MCF-7) incubated with **[EuL**^**imag-46**^**]** for 24 h. Scale bar: 10 μm. Reproduced with permission from ref (369). Copyright 2020 American Chemical Society.

Maury, Tripier et al. reported the first pyclen-based ligand bearing two picolinate intraligand charge transfer transition antennae and one acetate arm, arranged in a dissymmetric manner.^440^ Eu(III) and Sm(III) complexes of this ligand **[LnL**^**imag-46**^**]** were explored toward the development of luminescent bioprobes.^440^ Although TACN derivatives bearing pendent arms that possess antennae with intraligand charge transfer have been shown to be good luminescent agents for two-photon luminescent lanthanide bioprobes, bioconjugation of these complexes are synthetically challenging. However, pyclen offers a solution by functionalization of the *para* position in pyridine (i.e., X = Br, I, OH, and NO_2_) for further bioconjugation or targeting purposes.^440^ The complexes **[EuL**^**imag-46**^**]** were involved in bioimaging experiments using T24 cells. High-quality images were obtained with **[EuL**^**imag-46**^**]** with an excellent signal-to-noise ratio showing a diffuse staining of the different parts of the cells that are also visible in the transmitted image. Although **[SmL**^**imag-46**^**]** is weakly emissive, good images were observed due to the sharp Sm(III) emission bands which distinguished themselves from the fluorescence background.^440^ The arrangement of coordinating groups has an influence on the stability and photophysical properties of pyclen chelates.^437,438^ Building upon the successful two photon imaging using **[LnL**^**imag-46**^**]**,^440^ an in-depth study on the whole family of pyclen-based probes derived from **[LnL**^**imag-46**^**]** (Ln = Eu(III), Tb(III), Sm(III), Dy(III), and Yb(III)) were explored.^369^ A symmetrically arranged regioisomer **[LnL**^**imag-47**^**]** was explored to understand the importance of the dissymmetric arrangement.^369^ Comparing the photophysics of the regioisomers **[LnL**^**imag-46**^**]** and **[LnL**^**imag-47**^**]**, nonsymmetric **[EuL**^**imag-46**^**]** exhibits excellent photophysical properties in MeOH and H_2_O compared to **[EuL**^**imag-47**^**]**. This variation in photophysical properties is due to the existence of a mixture of two species in solution with different hydration number (*q*) in symmetrical regioisomer **[LnL**^**imag-47**^**]**, where the partial water coordination quenches lanthanide luminescence.^369^ This unexpected effect is related to the subtle difference in steric protection of the central ion afforded by the two different regioisomers and illustrates the crucial role of the ligand scaffold design.^437,438^ Slight variations in the antenna were incorporated to form **[YbL**^**imag-48**^**]** and **[LnL**^**imag-49**^**]** (Ln = Tb(III), Dy(III)). Upon incubating **[EuL**^**imag-46**^**]** in MCF-7 cell line for 24 h, one- and two-photon excitation microscopy revealed that the complex was successfully internalized and mainly localized in the cytoplasm (Figure 17). However, the PEG derivatives of the complexes required a mild fixation process to permeabilize the cell membrane to enhance cellular internalization, leading to a more diffuse cytoplasmic localization with a stronger accumulation in the nucleus, where the brightest spots indicate the nucleoli.^369^

Bifunctional analogues of the pyclen ligand were accessed by utilizing the *para* position in pyridine of **[LnL**^**imag-46**^**]** to incorporate propargylamine to allow coupling on biological vectors for reaching deep tissue imaging.^370^ The hydrophilic **[TbL**^**imag-51**^**]** was not internalized in HEK cells after 6 h of incubation: the complex was primarily present in solution and accumulates in floating membrane clusters from dead cells that are in easily accessible lipophilic areas. In the case of **[TbL**^**imag-50**^**]**, apparent internalization is observed in the cells with pronounced labeling of the cytoplasmic area and nucleoli. It is worth noting that the amount of DMSO used for the staining is rather high (10% *vol*/*vol*), inducing strong membrane permeabilization or even acting as a fixing agent.^370^

#### 3.3.4 Cyclen-Based Complexes

The macrocyclic ligand DOTA was first reported as a chelate in 1976^441^ and the first lanthanide complex was published by Bryden et al. in 1981.^442^ It exhibits a very strong complexing ability toward Ln(III) ions because it provides a preformed internal cavity of eight donor sites well suited to wrap around the metal ions.^443^ This results in tightly packed complexes with high thermodynamic stability, conformational rigidity, and kinetic inertia.^444^ Owing to its remarkable stability, cyclen based octadentate (DOTA) and heptadentate (DO3A) complexes of lanthanides have been widely studied as MRI imaging agents, some of which are approved for clinical use.^445,446^ This macrocyclic family has also been explored toward cellular imaging which is mentioned below.

**Figure 18:**
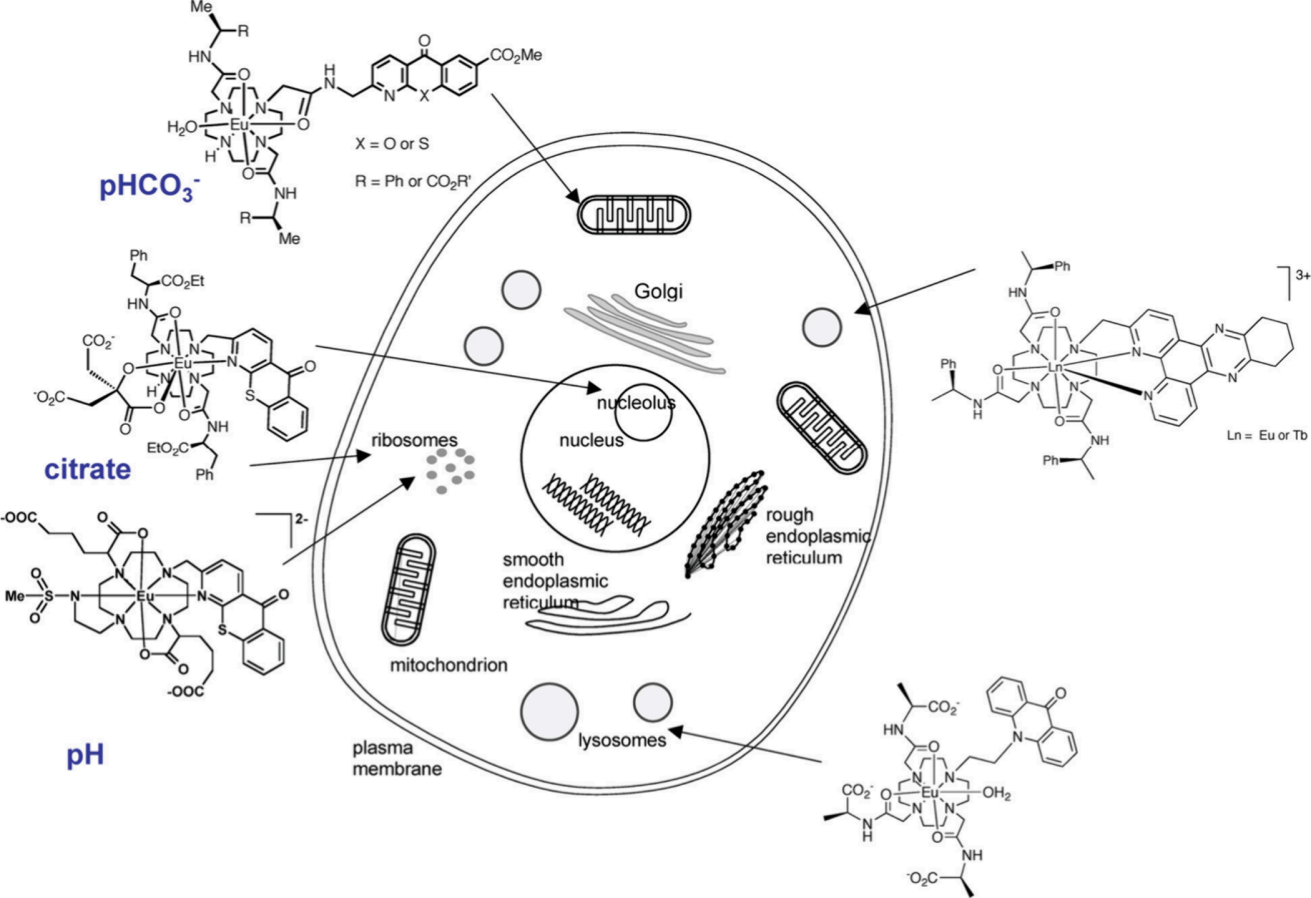
Schematic illustration of the dominant intracellular localization profiles of the emissive Eu(III) and Tb(III) macrocyclic complexes reported by Parker et al. Reproduced with permission from ref (45). Copyright 2009 American Chemical Society.

Luminescent cyclen-based complexes have been extensively studied by Parker et al. for their cellular uptake properties. This has been achieved by systematic variation in charge, lipophilicity, antenna, and bulkiness of the complex.^45,313,410,447−453^ Most of these complexes were reported to enter the cell by macropinocytosis,^410,449^ as demonstrated by the use of inhibitors (wortmannin, amiloride, chlorpromazine, filipin, sucrose, temperature decrease to 4–5 °C), or promoters (phorbol ester, fatty acid glycerol) of different cellular pathways and of inhibitors of endosome maturation (chloroquine, monensin).^342^ The accumulation of the complexes in cells was determined by luminescence microscopy and ICP-MS. Changes in the antenna, the linker, or the lanthanide do not seem to impact the cell uptake pathways of these complexes.^410,449^ However, different subcellular localization profiles have been seen, with species accumulating in lysosomes,^306,313,411,450,451,453^ mitochondria,^410,448,453^ or the nucleolus.^410,451,452^ Some compounds showed an accumulation in mitochondria before relocating into lysosomes.^291,356,357^ The antenna and the nature of the linker play an important part in the subcellular localization of the complexes.^410^ In contrast, neither the overall charge of the complex nor the lanthanide ion influences the subcellular distribution.^410^ Notably, the accumulation of complexes in the nucleoli of cells was shown to be linked with more permeable cell membranes,^306^ which was observed in cells under stress conditions.^342^ Thus, a nucleolus localization may indicate that the complex perturbs the cell and changes the permeability of the cytoplasmic membrane. These studies by Parker et al. have been extensively reviewed (Figure 18);^2,45,342,356,410^ therefore, it is not discussed in detail here.

The complexes of trans-methylated cyclen bearing dipicolinate arms (H_2_Me-DODPA) have been reported to have good stability with no inner-sphere water molecules.^454^ Further functionalization of the dipicolinate antenna has led to complexes suitable for two-photon imaging.^371^

**Figure 19:**
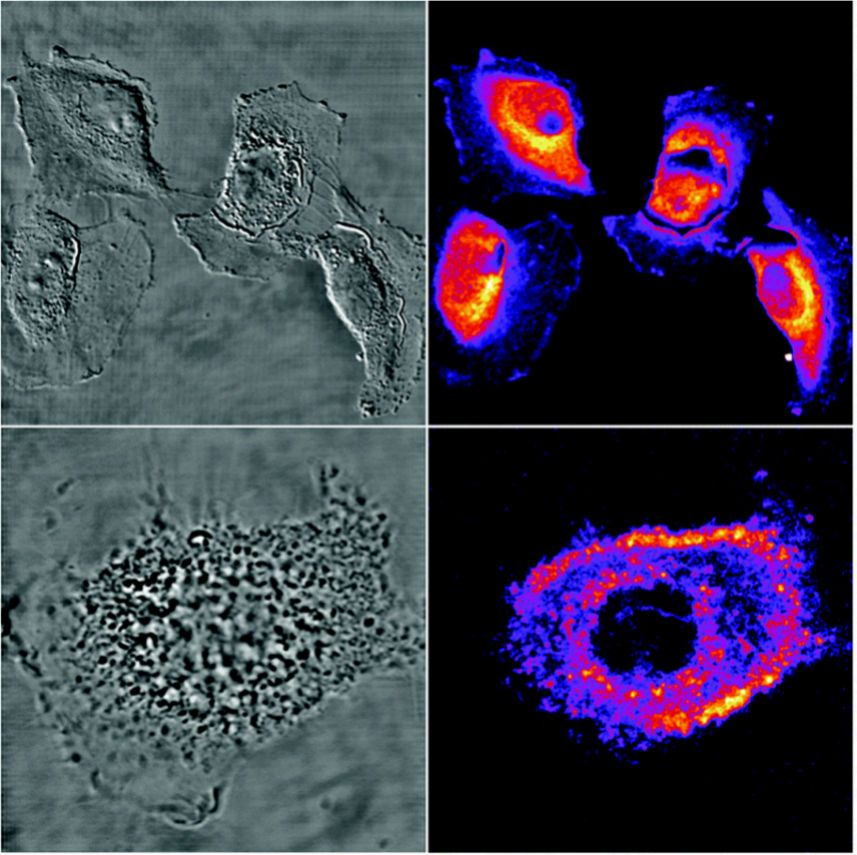
Two-photon imaging of T24 cells costained for 4 h with **[YbL**^**imag-53**^**]**^**+**^ (*c* = 10^–5^ mol L^–1^, two-photon excitation at λ_ex_ = 800 nm, detection in a descanned mode in the residual ILCT emission) (right) and transmitted light DIC image (left) using an LSM710 NLO (Carl Zeiss) microscope. Reproduced with permission from ref (372). Copyright 2017 Royal Society of Chemistry.

Two-photon imaging of live T24 cells was performed using a solution of **[EuL**^**imag-52**^**]**^**+**^ in phosphate buffer. After 4 h of incubation, **[EuL**^**imag-52**^**]**^**+**^ was internalized in cells and accumulated in the perinuclear zone and at the plasmalemma in spot-like cytoplasmic structures (Figure 19).^371^ It was found that the 28 h cell incubation in the presence of the complex does not impair cell viability or proliferation, and the staining remains present in dividing mitotic cells. The unambiguous identification of **[EuL**^**imag-52**^**]**^**+**^ inside living cells was confirmed by the collection of the emission spectrum from the image.^371^ Colocalization experiments with Nile Red (a lipophilic membrane stain) were performed showing overlap of the signals in most of the vesicular structures which suggested the endocytic or pinocytic internalization pathway of **[EuL**^**imag-52**^**]**^**+**^.^371^

Exploiting the benefit of the smaller size of Yb(III) over Eu(III), the Me-DODPA ligand mentioned above was used to chelate Yb(III) to form **[YbL**^**imag-53**^**]**^**+**^, and to test its potency toward two photon NIR-to-Vis and NIR-to-NIR imaging.^372^ The photophysical properties of **[YbL**^**imag-53**^**]**^**+**^ in water are excellent, showing an absence of coordinated water and a high brightness in the NIR spectral range. Live T24 cells were stained with **[YbL**^**imag-53**^**]**^**+**^ in phosphate buffer (Figure 19). After 4 h of incubation, two photon images (NIR-to-Vis configuration) reveal the spontaneous internalization of **[YbL**^**imag-53**^**]**^**+**^ in live cells and accumulated in the perinuclear zone. Spot-like cytoplasmic structures can be identified that can be assigned to lipophilic vesicles by analogy with **[EuL**^**imag-53**^**]**^**+**^.^371^

NIR-to-NIR imaging experiments were performed on **[YbL**^**imag-53**^**]**^**+**^. Identical images were obtained for both channels as both emissions came from the same complex. The visible image showed a high signal-to-noise ratio in all parts of the cells.^372^

Parker, Li et al. reported a series of Eu(III) tricationic complexes bearing an inner sphere water to observe the variations in emission lifetime and spectral signature as a function of probe environment has elucidated the nature of changes to probe speciation.^373^ Using the cationic Eu(III) complex which targets primary celia in living cells as a starting point^455^**[EuL**^**imag-54**^**]**^**3+**^; two other complexes were explored, one with a piperazine whose nitrogen is protonated at ambient pH **[EuL**^**imag-55**^**]**^**4+**^, while the other probe contains a piperazine linked to thieno-[3,2-*b*]-pyrrole-[3,2-*d*]-pyridazinone derivative^456,457^**[EuL**^**imag-56**^**]**^**4+**^, which has been shown to serve as an activator of the glycolytic enzyme pyruvate kinase M2,^458,459^ with a view to exploring its use subsequently as a probe that may selectively bind to a pocket on the PKM2 subunit interface. Nonspecific binding of these complexes to bovine serum albumin was observed (log *K*_a_ = 5.5–6.2). **[EuL**^**imag-55**^**]**^**4+**^ and **[EuL**^**imag-56**^**]**^**4+**^ were found to localize on the lysosomes of HeLa cells, while **[EuL**^**imag-54**^**]**^**3+**^ had little lysosomal staining and some nuclear staining.^373,460^

Transactivator of transcription of human immunodeficiency virus is a cell penetrating peptide dimerized by disulfide bonds which localizes on the cytosol.^461,462^ Maury, Sénèque et al. tagged this peptide onto heptadendate DO3A picolyl complexes for cytosol localization of these complexes **[LnL**^**imag-57**^**]** to **[LnL**^**imag-61**^**]**^**–**^ (Ln = Eu(III) and Tb(III)).^374^ Various electron donating or withdrawing aryl groups were attached on the picolyl pendent arm for better two-photon absorption properties. It is desired to have the two-photon cross-section of the probes higher than NAD(P)H and FAD (which undergo two-photon excitation giving rise to autofluorescence in cells) to be visualized in cells (if they accumulate sufficiently enough to be detected easily). Emission from **[TbL**^**imag-57**^**]** and **[EuL**^**imag-59**^**]** was observed in HeLa cells (incubated for 1 h). Diffuse emission was observed in the entire cell (including nucleus) indicating cytosolic delivery of the complexes, while the punctate emission is attributed to **[TbL**^**imag-57**^**]** trapped in endosomes even after 4 h. Cytotoxicity studies reveal **[EuL**^**imag-59**^**]** to be the least toxic, while **[TbL**^**imag-57**^**]** to **[TbL**^**imag-59**^**]** was toxic. In order to address toxicity with Tb(III) complexes, charged pendants on the amide electron donating group were added: cationic (β-alanine, **[TbL**^**imag-60**^**]**^**+**^) and anionic (succimide **[TbL**^**imag-61**^**]**^**–**^). This significantly lowered the cytotoxicity of the Tb(III) complexes. Finally, multiplex imaging **of [EuL**^**imag-59**^**]** and **[TbL**^**imag-60**^**]**^**+**^ or **[TbL**^**imag-61**^**]**^**–**^ (with similar two photon brightness) demonstrated the cytosolic delivery demonstrated the cytosolic delivery and colocalization throughout the entire cell.^374^

In separate work, Sénèque et al. investigated two cell penetrating peptides: TP2, a spontaneous membrane translocating peptide,^463^ and ZF5.3, a zinc finger peptide,^464^ by attaching them on DO3A picolyl-type complexes.^375^ One-photon time-gated and two-photon confocal imaging was performed with the complexes on NIH-3T3 cells. No luminescence was detected with complexes conjugated with TP2 peptide. This was due to the polarity effect on the fluorescent cargo attached to the TP2 peptide, having strong impact on membrane translocation efficiency, as the complexes were anionic. Polarity did not influence ZF5.3 peptide, which made **[TbL**^**imag-62**^**]**^**–**^ visualize in the cytosol of NIH-3T3 and HeLa cells. **[TbL**^**imag-62**^**]**^**–**^ was hypothesized to enter cells by the endosomal pathway and to escape endosomes as per the behavior of ZF5.3.^375^

Parker et al. reported a DO2A-type cationic complex possessing an azathiaxanthone sensitizer **[EuL**^**imag-63**^**]**^**3+**^. Cellular uptake studies in HeLa, NIH 3T3, and HDF cell lines suggest selective staining of **[EuL**^**imag-63**^**]**^**3+**^ in the nucleoli.^376^ Colocalization experiments in live cells with **[EuL**^**imag-63**^**]**^**3+**^ or nucleolar stain SYTO RNA and experiments in fixed cells ascertain nucleoli staining. However, it is noteworthy that **[EuL**^**imag-63**^**]**^**3+**^ binds to citrate and human serum albumin (0.1 mM). Sørensen et al. used azathiaxanthone in a DO3A scaffold **[EuL**^**imag-64**^**]** and **[EuL**^**imag-65**^**]** to visualize its cellular permeability.^377^ The complexes were investigated on a high-resolution laser scanning confocal microscope.^465^ Cell uptake and colocalization studies for **[EuL**^**imag-64**^**]** and **[EuL**^**imag-65**^**]** in living mouse skin fibroblasts (NIH-3T3) suggest predominant localization in the mitochondria. Time-resolved emission spectra (λ_ex_ = 355 nm) were measured from the cells stained with **[EuL**^**imag-64**^**]**, which confirms cellular permeability. The complexes were then tested on a conventional microscope on formaldehyde fixed HeLa cells. No signals were obtained from **[EuL**^**imag-65**^**]** stained cells, but bright images were obtained on cell nuclei for **[EuL**^**imag-64**^**]**.^377^

Cyclin-dependent kinases (CDKs) and their natural inhibitors are central and crucial to cell cycle regulation.^466^ Their functions are altered in tumor cells in which the activity of cyclin A/CDK2, one of the key cell cycle kinases, is overexpressed.^467^ CDK interacts with the critical cell cycle substrates through the cyclin binding motif of which the consensus sequence has been found in many cell cycle and tumor suppressor proteins.^468^ Wong et al. reported a series of Eu(III) DO3A picolinate-type complexes (one with an amide linker connecting the macrocycle to picolinate moiety and the other has picolinate directly attached to the macrocycle) tagged to Cyclin A-specific peptides.^378^ Cellular uptake studies in HeLa cell line show the complexes with direct picolinate linkage to the macrocycle to have bright Eu(III) emission. *In vitro* imaging experiments in HeLa and HK1 cell lines show **[EuL**^**imag-66**^**]**^**+**^ to have manifested detectable and indicated *in vitro* responsive emission enhancement upon adding cyclin A into HeLa cells. Upon the addition of a Cyclin A inhibitor, the Eu(III) emission diminished in HeLa cells, which suggests the selectivity of **[EuL**^**imag-66**^**]**^**+**^ to Cyclin A.^378^

Folates (also called vitamin B9, folacin, pteroyl-l-glutamic acid, or pteroyl-l-glutamate) are essential for the maintenance of the human genome and cell health, due to their central role in key metabolic functions, such as RNA and DNA biosynthesis.^469^ Folate receptors have minimal expression in normal tissue, but increased expression is observed in malignant cells/tissues, including, colorectal, ovarian, breast, lung, cervical, renal, kidney, brain and nasopharyngeal carcinomas, and this increased expression has been linked with tumor progression.^470^ This makes folate receptors a potential target for cancer diagnosis/therapy,^471^ where folic acid can be used as a biocompatible and nonimmunogenic targeting motif to covalently conjugate with an imaging or a therapeutic agent.^472^ Antifolates, such as methotrexate, have been directly exploited as therapeutic agents.^473^ Fluorescent agents tethered to folic acid have been investigated for intraoperative identification of malignant disease.^474^

Plush et al. reported a DO3A conjugated to folic acid or pteroic acid (substitute for folic acid) where different linker lengths were explored for probe emission and cellular uptake.^379^ Uptake studies in HeLa using the complexes show high intracellular concentration for **[EuL**^**imag-67**^**]** and **[EuL**^**imag-68**^**]**. Folate receptors showed higher affinity for folic acid appended Ln(III) complexes over pteroic acid appended Ln(III) complexes. It is noteworthy that the complexes mentioned here have the shortest linker lengths, which have better cellular uptake and photophysical properties (emission intensity decreased over increasing linker length) than the longer linker length ones. However, these complexes were sensitized through folic acid (at 280 or 360 nm) resulting in weak luminescence.^379^ Building upon this, carboxystyril was used as a sensitizer, which was placed between DO3A and folic acid **[EuL**^**imag-69**^**]**, methotrexate (an antifolate) **[EuL**^**imag-70**^**]**.^380^ Folate receptor-positive (HeLa cervical cancer cells, 293t embryonic kidney cancer cells, MDA-MB-231 breast cancer cells, U251 glioma cells, U2OS osteosarcoma cells, and RAW264.7 auto immune leukemia cells) and folate receptor-negative (A549 lung cancer cells, PC 3 prostate cancer cells, and CAL-27 human tongue squamous cell carcinoma cells) cells were used to test the complexes. No luminescence enhancement was observed in folate receptor negative cells, but high luminescence intensity was observed in folate receptor positive cells after 24 h of incubation. In CAL-27 cells (which has low folate receptors), high luminescence emission was observed in **[EuL**^**imag-70**^**]**, suggesting an alternate uptake mechanism. Increased cytotoxicity was observed in methotrexate-based **[EuL**^**imag-70**^**]** over **[EuL**^**imag-69**^**]**, suggesting a synergistic effect of Ln(III) ion/chelate near the methotrexate moiety, or the release of methotrexate following degradation of the amide bond after a long incubation time.^380^

Folic acid consists of three distinct parts: a pterin moiety, a *p*-aminobenzoate linker, and a glutamate residue. This molecular structure contains at least five possible sites for functionalization, which are potentially useful for the preparation of folate conjugates. Quici et al. studied the relationship between the conjugation site and the recognition capability of the resulting conjugates by folate receptors.^381^ A phenacyl DO3A unit was conjugated to α- and γ-carboxylic groups of the glutamic residue, forming **[EuL**^**imag-71**^**]** and **[EuL**^**imag-72**^**]**, respectively, and the nitrogen atom of the *p*-aminobenzoic residue for **[EuL**^**imag-73**^**]**. The two regioisomers **[EuL**^**imag-71**^**]** and **[EuL**^**imag-72**^**]** were synthesized as a mixture and carefully separated, while **[EuL**^**imag-73**^**]** was obtained through multistep synthesis. Cellular internalization of the complexes was observed in SKOV-3, IGROV-1, and A2780 ovarian cancer cells. The amount of internalization was the same for all the three complexes, suggesting the indifference in the position of the DO3A unit on folic acid. No internalization was observed for A2780 cells that do not express folate receptors, thereby indicating that the internalization of the complexes was folate receptor-mediated. In SKOV-3 cells, binding activity follows the order **[EuL**^**imag-72**^**]** > **[EuL**^**imag-71**^**]** > **[EuL**^**imag-73**^**]**. Competition experiments in the presence of folic acid showed selectivity and specificity of **[LnL**^**imag-72**^**]** for the folate receptor comparable to that of folic acid, thus suggesting that the conjugated moiety did not likely affect folic acid binding properties. **[EuL**^**imag-72**^**]** was chemically and photophysically stable after the internalization process.^381^

Faulkner et al. explored a series of Tb(III) complexes conjugated to folates on the α- and γ-carboxylic groups of the glutamic residue along with different PEG chain lengths.^382^ This was to establish the ideal design of synthetic molecules which bind to folates on cells with strong affinity. Tb(III) reporting moiety attached on the γ-unit displayed the highest binding affinity, with **[TbL**^**imag-74**^**]** being the strongest, as established on a flow cytometry binding competition assay in folate receptor α and β expressed KB and CHO cell lines, respectively. A substantial folate fluorescence in **[TbL**^**imag-74**^**]** was observed in steady-state mode which overlaps with Tb(III) emission. This was eliminated when **[TbL**^**imag-74**^**]** was observed in time-resolved phosphorescence mode. Incorporation of a light-harvesting antenna onto **[TbL**^**imag-74**^**]** will enable phosphorescence imaging lifetime microscopy on cells.^382^

While *d*–*f* hybrids have been widely used as bimodal imaging probes,^475,476^ here we focus on complexes whose transition metal sensitizes lanthanide luminescence. Ward et al. reported a DO3A ligand covalently attached to a Ir(III) phenanthroline complex where the Ir(II) is coordinated to a fluoropyridine ligand **[EuL**^**imag-75**^**]**.^383^ The triplet MLCT state of Ir(III) phenanthroline was 21,000 cm^–1^, which was sufficient to sensitize Eu(III) emissive level (17,500 cm^–1^). In Human dermal fibroblast cells, **[EuL**^**imag-75**^**]** (λ_ex_ = 780 nm) showed punctuate and cytoplasmic staining toward the perinuclear region. However, when the cells were incubated in phosphate buffered saline, a small population of cells stained the nuclei. The partial energy transfer from Ir(III) to the Eu(III) center allowed simultaneous detection of green Ir(III) and red Eu(III) emissions from **[EuL**^**imag-75**^**]**.^383^

#### 3.3.5 Porphyrin-Based Complexes

Porphyrin or porphynoid lanthanide complexes have been attractive for their NIR luminescent properties but also for the potential theranostic applications based on the studies of metalloporphyrins in photodynamic therapy.^3^ Lakowitz et al. reported the proof of concept of two photon sensitization of Yb(III) complexes in 2001.^477^ A decade later, Kwok, Wong et al. reported a Yb(III)-porphyrinato complex tethered to a rhodamine B antenna through a polyethylene glycol chain.^384^ To improve its stability in water, a Yb(III)-porphyrin was capped with a tripodal monoanion [(η^5^-C_5_H_5_) Co(OCH_3_)_2_P=O_3_]^−^, forming **[YbL**^**imag-76**^**]**.

This complex was tested for influence on the luminescence with different analytes such as citrate, carbonate, phosphate Zn(II), Cu(II), and at different pH. No significant changes were observed with these analytes which ascertain the biocompatibility of this complex. The ligation of rhodamine B improves the solubility of the porphyrin complex in water and aids the targeting to mitochondria.^384^ The sensitization of Rhodamine-B to Yb(III) in **[YbL**^**imag-77**^**]** is not fully optimized as there is an intense residual ligand centered emission at around 600 nm (Φ = 2.5% in water, τ_H2O_ = 1.1 μs, two photon absorption cross-section = 375 GM in DMSO). **[YbL**^**imag-77**^**]** successfully stained HeLa cells and two-photon microscopy reveals specific localization within the mitochondria in contrast with the derivative without the Rhodamine-B **[YbL**^**imag-76**^**]**, which was spread out through the cytoplasm. It is noteworthy that the cell images were recorded in the classical NIR-Vis configuration by exploiting the ligand-centered emission (λ_ex_ = 860 nm; detection range = 500–800 nm) rather than the Yb(III) emission.^384^

Perfluorinated porphyrins as antenna ligands show promising sensitization toward Yb(III) luminescence as they also do not carry C–H bonds near the metal center, although their solubility only in organic solvents is a challenge.^478^ Therefore, Jing, Zhang et al. developed a series of biocompatible Yb(III) complexes of β-fluorinated porphyrinates through modification of meso-β-phenyl and β-peripheral positions.^385^ These complexes were capped with a tripodal monoanion [(η^5^-C_5_H_5_) Co(OCD_3_)_2_P=O_3_]^−^ to improve the stability of the complexes in water. Substitution on meso-phenyl groups has little effect in the Yb(III) NIR emission but improves the solubility of these porphyrins in water (0.1% DMSO was necessary).

**Figure 20:**
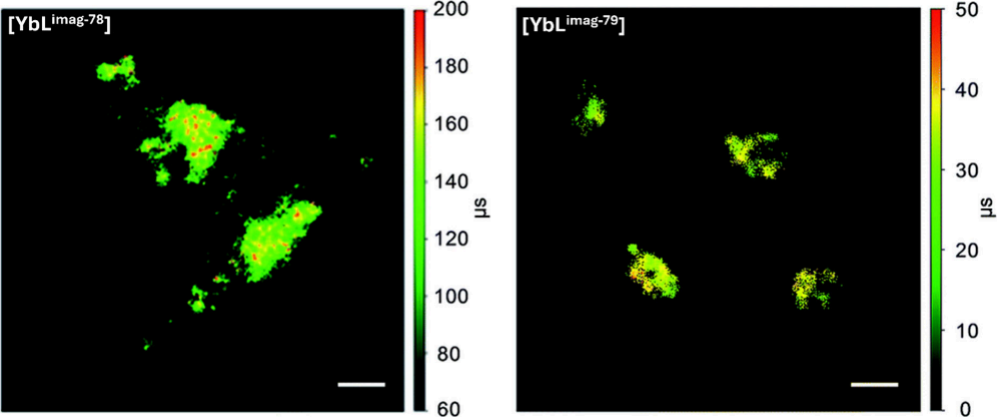
NIR time-resolved images of living HeLa cells incubated with 10 μM **[YbL**^**imag-78**^**]** and **[YbL**^**imag-79**^**]**. Scale bar: 10 μm. Reproduced with permission from ref (385). Copyright 2018 Royal Society of Chemistry.

Based on their photophysical properties and their cellular uptake, determined by ICP-MS, the complexes proved to be very good candidates for cellular imaging with **[YbL**^**imag-78**^**]** (Φ = 10(1)%) and **[YbL**^**imag-79**^**]** (Φ = 2.4(0.1)%). It is noteworthy that **[YbL**^**imag-78**^**]** has very good NIR luminescence and **[YbL**^**imag-79**^**]** has higher cellular uptake. These complexes were incubated with HeLa cells and colocalized with Lyso-Tracker Green and the cellular images taken using confocal FLIM microscopy (Figure 20). From these studies, **[YbL**^**imag-78**^**]** and **[YbL**^**imag-79**^**]** preferentially colocalize in the lysozyme of cells. Cytotoxicity studies reveal IC_50_ < 1 μM with negligible phototoxicity toward HeLa cells (cell viability >80% at 10 μM).^385^

### 3.4 Podate-Based Ligands

The dipicolinate-based donor-π conjugated antenna previously described in macrocyclic structures was also incorporated in a nonmacrocyclic podate ligand, L, *N*,*N*′-bis(2-pyridylmethyl)-1,2-(*R*,*R* or *S*,*S*)-cyclohexanediamine. Maury, Melchior, Piccinelli et al. reported two enantiomeric complexes of *C*_1_ symmetry, (***R***,***R***) **[LnL^imag-80^]^+^** and (***S***,***S***) **[LnL**^**imag-80**^**]**^**+**^ (Ln = Eu(III), Sm(III)).^386^ Phototophysical studies with these complexes reveal that the most emissive complex was (***S***,***S***) **[EuL**^**imag-80**^**]** (Φ = 0.11% in H_2_O, τ_H2O_ = 480 μs, ε^max^ = 44,652 L mol^–1^ cm^–1^ in H_2_O, *B*^(1)^ λ_max_ = 4912 L mol^–1^ cm^–1^ in H_2_O, *q* = 1.4). Cell-viability study with (***S***,***S***) **[EuL**^**imag-80**^**]**^**+**^ showed promising results for 6 and 24 h time window in HEK293T and THP-1 cell lines. Two-photon imaging experiments showed internalization of the complex^386^ but also indicated different emitting species contribution inside the cells. It has been reported that cationic complexes can be internalized via an active endocytosis mechanism resulting in a final localization of the probes in the endosomes and lysosomes.^368,479^ but also via passive diffusion mechanisms, leading to electrostatic accumulation near the charged inner mitochondria membrane.^480^ In the present case, the perinuclear diffuse localization in the cytosol suggests either a different internalization process, intracellular release of the probe from the endosomes to the cytosol, or their fusion with the endoplasmic reticulum.^386^

### 3.5 Polymetallic Structures: Metallacrowns, Dendrimer, and Metal–Organic Frameworks (MOFs)

Strategies to increase luminescence output in imaging probes have involved incorporation of lanthanide probes in metallacrowns, dendritic/polymeric structures, and metal–organic frameworks. We have reviewed these lanthanide-containing assemblies in their cell imaging applications.

Metallacrowns are macrocycles which resemble crown ethers and built up of repeating [M–N-O]_*n*_ units, where the carbon atoms are replaced most commonly by *d*- and *p*-block metals, whereas the cavity of the macrocycle binds Ln(III).^481^ Strong luminescence from the Ln(III) is envisaged in this motif as the quenching C–H oscillators are absent, and this has led to the investigation of Ln(III)-based supramolecular metallacrowns for cellular imaging applications. A series of NIR luminescent lanthanide metallacrowns, **[LnL**^**imag-81**^**]**, **[LnL**^**imag-82**^**]** (Ln(III) = Nd, Er, Yb) were reported by Petoud and Pecoraro.^482^ The quinHA ligand enables excitation with low energy light, while the rigid structure excludes solvent molecules from the lanthanide, affording high quantum yields. In order to overcome the hydrophobicity of this system, a new generation of metallacrowns was envisaged.^387^ Upon introducing pyrazinehydroxamic acid (H_2_pyz_HA_), highly water-soluble NIR emitting compounds, **[LnL**^**imag-83**^**]** (Ln = Yb(III), Nd(III)) were obtained.^388^ Cell-fixation studies carried out by systematic monitoring over a month upon preincubating HeLa cells with **[LnL**^**imag-83**^**]** (>150 μM) were shown to preserve the cell morphology and its features. HeLa cells incubated with **[YbL**^**imag-83**^**]** produce sufficiently intense signal in the NIR region which can be captured by detectors from commercial confocal microscopes, unlike Nd(III) analogues. Cellular studies reveal successful permeabilization and labeling of the nucleus, stained with visible emitting propidium iodide. Exposure of the cells to UV-A light for 8 and 10 min did not affect the cell morphologies but exposure for 12 h led to cell death.^388^ The ability of **[YbL**^**imag-83**^**]** to label preferentially necrotic cells was explored as an agent to induce cell fixation upon UV-A exposure and NIR imaging stain.^388^ The studies showed that **[YbL**^**imag-83**^**]** is highly photostable and demonstrated selectivity toward cell necrosis^388^ and nucleus targeting.^387^ Using bifunctional linkers salicylhydroxamic acid and isophthalic acid, a set of stable metallacrowns based on Ga(III) framework was developed for visible and NIR emitting Ln(III) **[LnGa**_**4**_**]**_**2**_.^389^ Photophysical studies have demonstrated that the metallacrown scaffold in **[LnGa**_**4**_**]**_**2**_ sensitizes efficiently the characteristic emission of Nd(III), Pr(III), Sm(III), Tb(III), Dy(III), Ho(III), Er(III), and Yb(III) in the visible and/or NIR ranges in the solid state.^389^ Due to the promising photophysical properties of **[YbGa**_**4**_**]**_**2**_, it was used for cellular imaging. Epifluorescence signal from **[YbGa**_**4**_**]**_**2**_ detected in the NIR area results from the nonspecific interactions of **[YbGa**_**4**_**]**_**2**_ aggregates on the surface of living HeLa cells.^389^

Combining two series of Ln(III)/zinc(II) metallacrowns assembled using pyrazine and quinoxaline hydroximate building blocks, a water-soluble mixed-ligand MCs **[LnL**^**imag-84**^**]** with extended absorption to the visible range was reported.^390^ Hydroximate ligand derivatives with unique “encapsulated sandwich” structures that efficiently protect Ln(III) from nonradiative deactivations and sensitize characteristic NIR emissions of Yb(III), Nd(III), and Er(III) ions. NIR epifluorescence microscopy experiments in living HeLa cells incubated with 500 μg/mL of **[LnL**^**imag-84**^**]** show a granular pattern suggesting that **[LnL**^**imag-84**^**]** is localized in cytoplasmic vesicles.

Lanthanide nanoclusters Ln_9_Zn_8_ of 3 nm were prepared with enhanced antenna effect for cellular imaging.^391^ These ultrasmall nanoparticles are expected to have higher cellular uptake and deep tissue penetration for bioimaging applications. Applications of lanthanide nanoclusters have been limited due to their low stability, quenching by water and low dispersion in aqueous solution. In a new approach, a two-ligand system was used, with one of them delivered as the Zn-complex in a solvothermal preparation to yield the clusters protected by the ligands. **[Eu**_**9**_**Zn**_**8**_**]** displays strong luminescence compared with **[Tb**_**9**_**Zn**_**8**_**]**, attributed to the matching of the energy levels of the triplet states. The enhanced effect of **[Eu**_**9**_**Zn**_**8**_**]** is attributed to the dual antenna effect of the Zn-complex and the extra ligand. The **[Eu**_**9**_**Zn**_**8**_**]** shows uptake and excellent cell imaging in many cell lines.^391^

Dendrimers are highly branched polymeric chains used in catalysis and drug delivery. They are very efficient light-harvesting molecules^483,484^ and excellent energy donors which can easily be functionalized and can encapsulate several lanthanide ions, which may compensate for low quantum yields, particularly with NIR emitting Ln(III) ions.^485^ Dendrimers are built from successive replication (generation) of an initial dendron so that their radius grows linearly with the generation number, while the number of coordinating groups grows geometrically.^484^ Many have used dendrimers to improve the solubility of the molecule of interest and to improve its organelle localization and cellular uptake. Cationic dendrimers are reported to be cytotoxic, although this is not the case for anionic dendrimers with carboxylate arms.^342^ Dendrimers such as polyamidoamine are biocompatible and have demonstrated great potential in cancer therapy.^342^ Dendritic structures based on lanthanide macrocycles have been developed for their luminescent and radiopharmacutical applications.^439,486^

Petoud, Eliseeva et al. reported a luminescent Sm(III) based dendritic structure **[SmL**^**imag-85**^**]** (Φ = 2.2(2) × 10^–2%^ and 8.5(5) × 10^–4^% (NIR, ^4^G_5/2_ → ^6^F_*J*_) in DMSO; τ_DMSO_ = 15.1(6) μs), where eight Sm(III) ions were sensitized by 32 naphthalimide antenna moieties.^392^ Interestingly, the dendrimer complex exhibits both visible and NIR excitation. Cell viability of 80% was observed for **[SmL**^**imag-85**^**]** in HeLa cells and 88% in NIH 3T3 cells (2.5 μM), Confocal microscopy images of HeLa cells after incubation with 1 μM concentration of **[SmL**^**imag-85**^**]** indicate cellular uptake and distribution between cytoplasmic structures (probably lysosomes). Epifluorescence microscopy on HeLa cells confirmed the ability to detect Sm(III) emission in visible and NIR regions (λ_ex_ < 377 nm). **[SmL**^**imag-85**^**]** was stable in the cell after 24 h of incubation, allowing detection of the characteristic Sm(III) emission bands through the energy-transfer processes as dictated by the 2,3-naphthalimide moieties.^392^ This is one of the few examples of NIR luminescence of Sm(III) in cells.

In order to improve cell permeability and selective localization of imaging probes, cell-penetrating peptides are used where the imaging agent is tagged. However, these peptides are prone to enzymatic degradation. Therefore, peptidomimetics such as cell-penetrating peptoids are used.^487^ This is based on oligo-*N*-alkylglycine varying in different side chains connected to the N atom of the backbone. Peptoids are stable against proteases *in vivo* and *in vitro*, and they exhibit antibiotic properties. It is noteworthy that these peptoids bind to certain receptors and proteins. Schepers, Roesky et al. reported nanoscale clusters of pentadecanuclear europium and terbium hydroxyclusters which are ligated by cell penetrating peptoid monomers.^393^ This is achieved by reacting the cell penetrating peptoid monomer 2-[{3-(((tert-butoxycarbonyl) amino)methyl)benzyl}amino]acetic acid hydrochloride with [LnCl_3_·(H_2_O)_6_] (Ln = Eu, Tb) and dibenzoylmethane (DBMH) in the presence of potassium *tert* butoxide in methanol, resulting in the formation of the pentadecanuclear lanthanide hydroxy cluster [Ln_15_(μ_3_–OH)_20_(PepCO_2_)_10_(DBM)_10_Cl]Cl_4_ (represented as **[LnL**^**imag-86**^**]** Ln = Eu(III), Tb(III)). Owing to the distinct biological compatibility of the peptoids, the obtained clusters feature a pronounced propensity to cell penetration. **[EuL**^**imag-86**^**]** is more luminescent (Φ = 19(1)%) than **[TbL**^**imag-86**^**]** (Φ < 3%). Both the clusters were found to be moderately toxic in HeLa cell line. Cellular uptake in HeLa cell line (visualized using conventional fluorescent confocal microscopy and time-resolved long-lived luminescent microscopy) reveals internalization in cytoplasm, nucleus, and endosomal-lysosomal system. This suggests an endocytotic uptake and an endosomal escape, accompanied by a subsequent distribution to the cytoplasmic and nuclear compartments. The uptake and escape mechanism was hypothesized as an energy-dependent endocytic processes and validated by cellular uptake studies for 4 h at 4 °C. Energy depletion at 4 °C severely reduced the uptake, giving rise to the assumption that endocytosis was involved in the cellular uptake of the clusters. Although whether these clusters are chemically modified upon the cellular uptake is known, the concept of using structurally well-defined clusters as optical markers was proven. This is the first example where peptoids were used as supporting ligands to promote cellular uptake.^393^

**Figure 21:**
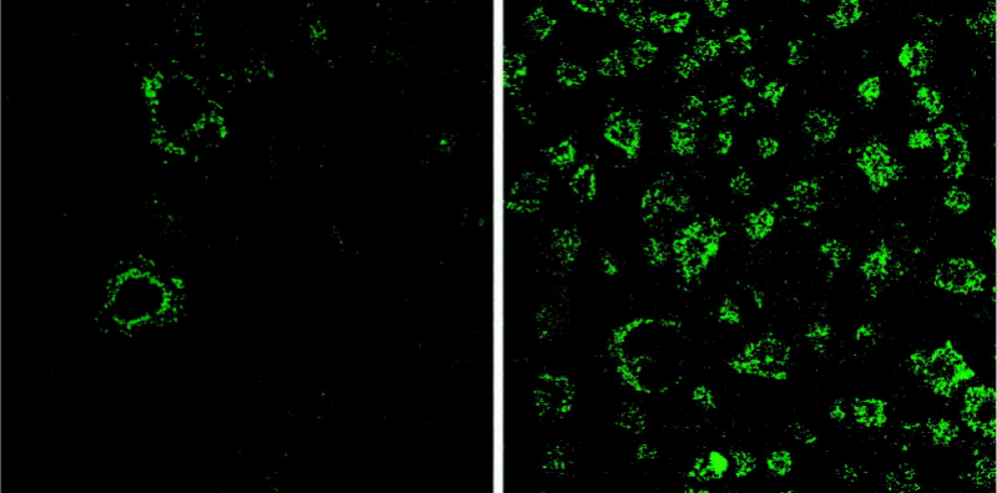
Three-photon confocal fluorescent microscopy images of **[TbL**^**imag-87**^**]** incubated in human lung carcinoma A549 (left) and human cervical carcinoma HeLa cells (right) (λ_ex_ = 800 nm). Reproduced with permission from ref (394). Copyright 2008 American Chemical Society.

A tripodal ligand, N-[2-(bis{2-[(3-methoxybenzoyl)amino]ethyl}amino)ethyl]-3-methoxybenzamide, was chelated to Tb(III) **[TbL**^**imag-87**^**]**.^394^ Single crystal X-ray revealed its existence as a polymeric assembly in linear form. Cellular uptake of **[TbL**^**imag-87**^**]** in HeLa, A549, and HONE1 cell lines was performed and observed on a three-photon confocal fluorescent microscope (Figure 21). After 1–2 min of exposure, pale green signals were observable in the cytoplasm as a punctuated pattern. After 1 h, >95% of the cells exhibited green luminescence, as was observed in the cytoplasmic foci around the cell nucleus. All three cell types were viable over the 24-h examination period, as exemplified by the presence of intact cell membranes with bright field images. As a control, no Tb(III) emission was observed that were directly excited under the same experimental conditions outside of the cell, proving that the three-photon processes occurred inside the cell. In addition, confocal microscopy of the cells with Hoechst 33342 labeled nuclei showed that the Tb complexes were internalized in the cytoplasm but not in the nuclei. **[TbL**^**imag-87**^**]** could be a potential candidate for future infrared excitation imaging dyes.^394^

Lam et al. prepared an Eu(III) nanorod by mixing Europium hydroxide (heated on a Teflon digestion bomb to form a europium nanorod) and organically modified silicate **[EuL**^**imag-88**^**]**, characterized using high-resolution TEM.^395^ Internalization of **[EuL**^**imag-88**^**]** in human lung carcinoma A549 and HeLa cells reveals red luminescence in the cytoplasm (around the nucleus). No emission was observed inside cells that were exposed to comparable concentrations of commercial EuCl_3_ or Eu_2_O_3_ as controls. Others have adopted a similar nanorod approach for immunoassays^488^ and live-cell imaging.^489^

Metal–organic frameworks (MOF) have shown great promise as new imaging probes. Their high surface area and controllable pore size makes them suited for drug delivery. The choice of the sensitizer for the lanthanide is built in the organic framework. Table 8 shows some representative ligands to construct lanthanide MOFs for imaging. One of the early examples of cellular detection of a near-infrared emitting MOF, **Yb-MOF**_**imag1**_, was based on octacoordinated Yb(III) with six carboxylates from three ligands and two oxygen atoms from two dimethylformamide molecules.^490^ In both water and buffer (pH 7.3), **Yb-MOF**_**imag1**_ showed characteristic Yb(III) emission with relatively small quantum yields and short microsecond biexponential lifetime decays, although these properties did not prevent imaging perhaps based on the localized signal initiated from the polymetallic design. The **Yb-MOF**_**imag1**_ is stable in some biological media, does not photobleach, and has an IC50 of 100 μg/mL, which is sufficient to allow live-cell imaging in HeLa and NIH 3T3 cells. Later the same group reported **Yb-MOF**_**imag2**_ and **Yb-MOF**_**imag3**_, consisting of 2-aminoterephthalate organic linkers.^491^ Both MOF contain [Yb_4_(OH)_4_]^8+^ clusters with four Yb(III) forming a tetrahedron with faces bridged by four μ_3_-hydroxides. Both exhibits longer wavelength absorption and photosensitization of Yb(III) NIR emission at 980 nm in DMF solution with slightly higher quantum yields. These well dispersed and miniaturized MOFs were incubated with living RAW 264.7 macrophage cells for 18 h. The collected NIR epifluorescence images (λ_ex_ = 482 nm) showed the Yb(III) emission signal, which reveal that these miniaturized MOFs can sustain the biological conditions of cell through the incubation process in the presence of living cells by continuing to generate bright emission.

**Table 8:**
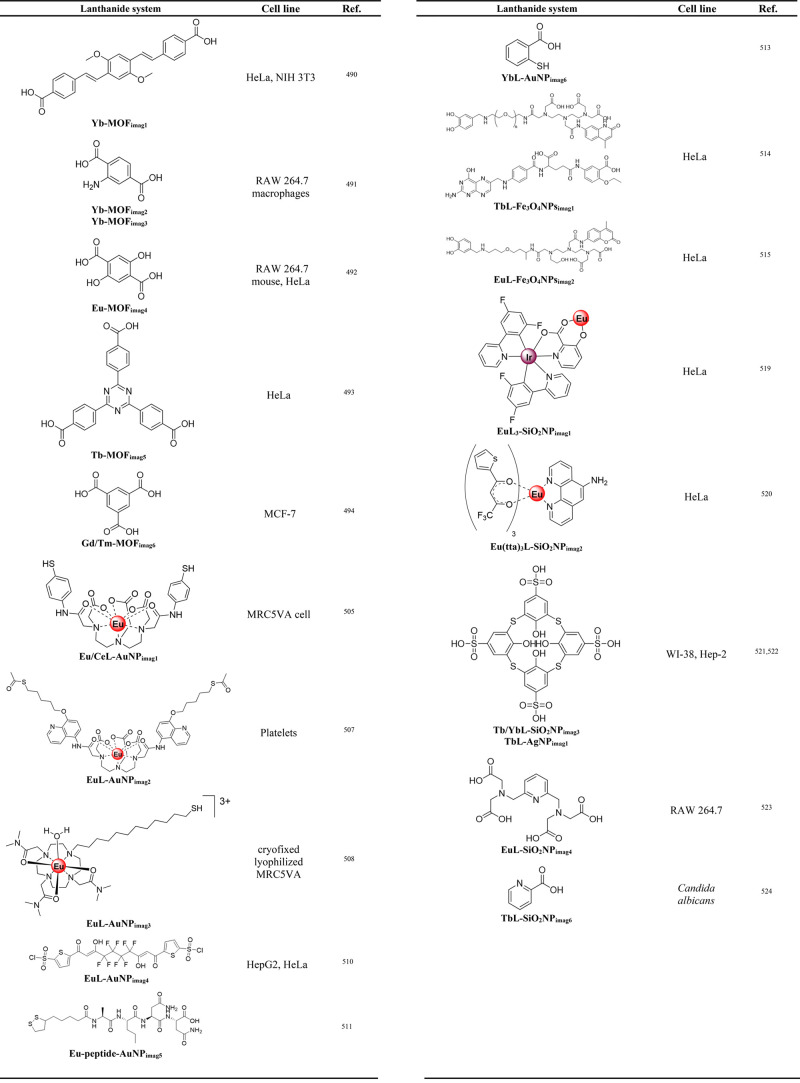

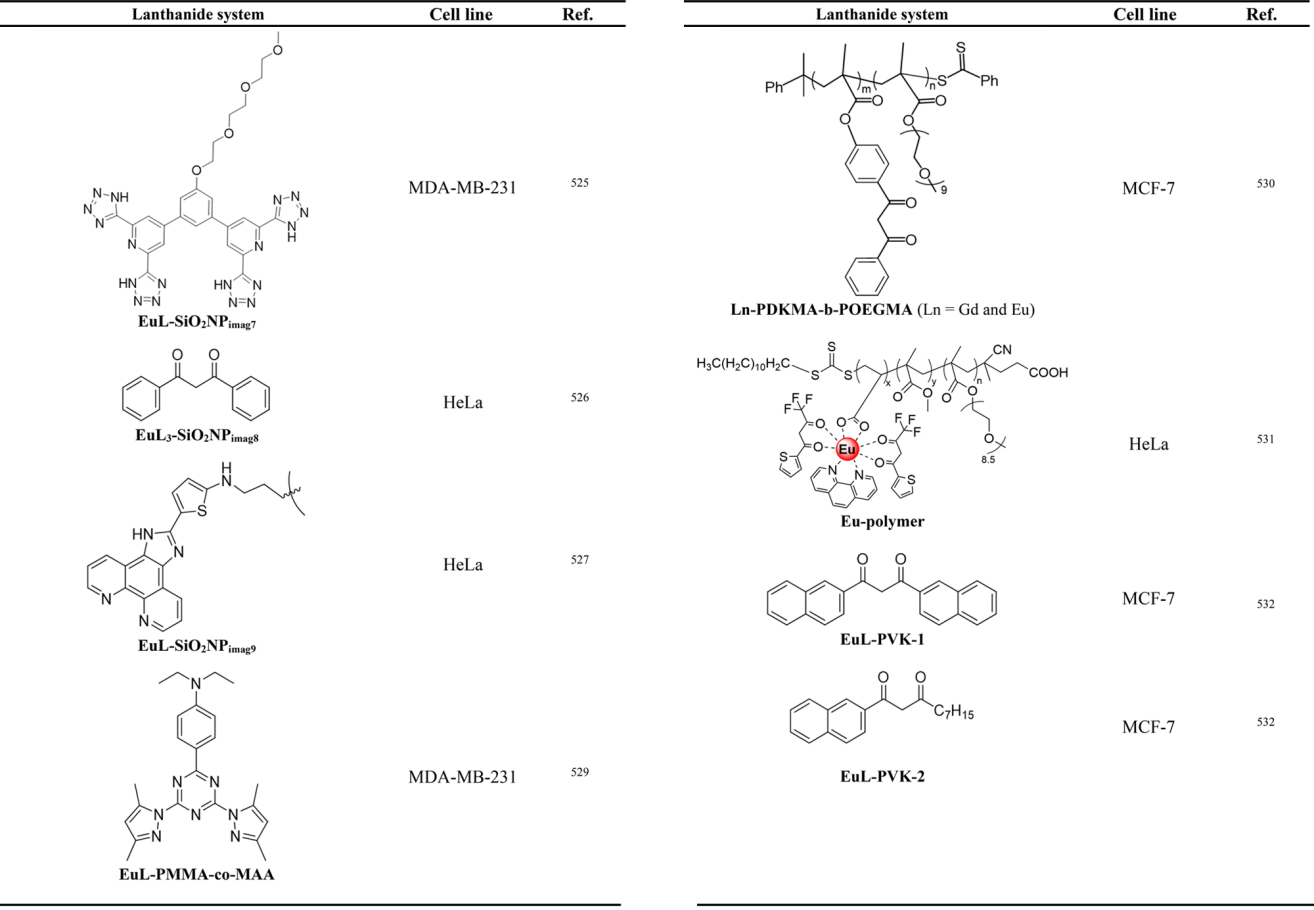
Lanthanide Systems Based on MOF and Nanoparticles for Imaging^a^

a Ligands are only shown for MOF and main components only for the nanoparticles.

A visible-emitted **Eu-MOF**_**imag4**_, with 2,5-dihydroxyterephthalic acid shows a three-periodic framework with octahedral cages ∼10.4 Å in diameter. *In vitro* studies using RAW 264.7 mouse macrophage and HeLa human cervical cancer cells demonstrate the viability of using these materials as bioimaging agents. The red emission is preserved even after 48 h incubation. However, the aggregation in biological media suggests that additional steps might be needed to further stabilize the particles.^492^ Tb-based metal–organic framework nanoparticles with good colloidal stability and stable fluorescence properties in an aqueous solution were prepared by a simple mechanical grinding of **Tb-MOF**_**imag5**_ with a biocompatible polymer surfactant. Their efficient cellular uptake via an energy-dependent endocytosis was observed by confocal laser scanning microscopy. By taking advantage of the porous nature of the **Tb-MOF**_**imag5**_ nanoparticles, an anticancer drug (doxorubicin) was successfully loaded and delivered to kill cancer cells to demonstrate their usage as a drug delivery vehicle.^493^

**Gd/Tm-MOF**_**imag6**_ showed unexpected upconversion luminescence as well as drug carrier properties for doxorubicin hydrochloride. The strongest upconversion luminescence with a lifetime of 379 ± 2 μs and a quantum yield of 0.76% could be achieved at the concentration of doped Tm(III) ions of 6%. Through modifying **Gd/Tm-MOF**_**imag6**_ with uniform mesoporous silica shells and folic acid, the drug loading was improved up to 41.5 mg g^–1^, and pH-responsive drug release increased to 64% from 12% by regulating the pH from 5.8 to 7.4. The cell imaging shows obvious blue and red luminescence under 980 nm laser excitation, and the up-conversion luminescence is unique because there is no autofluorescence from cells under 980 nm excitation.^494^

### 3.6 Nanoparticles

#### 3.6.1 Metallic Nanoparticles

Nanoparticles with a metallic core provide a wealth of applications in bioimaging and cellular detection and more recently nanotheranostics. Many of the materials are based on metal fluoride matrices, doping of oxides or iron oxide nanoparticles, recently reviewed. We have summarized the approaches which involve lanthanide complex functionalization to demonstrate the development from coordination chemistry to nanoscience (Table 8).^495−500^

Gold nanoparticles, AuNP, provide an attractive scaffold for attachment of luminescent probes due to their size tunability, availability of selective surface chemistry and, and the multimodality detection with electron and optical microscopy techniques. Even though most of organic fluorescent probes attached onto gold nanoparticles were reported to have their luminescence quenched,^501^ it has been shown that this is not the case for the lanthanide luminescence possibly due to its origin from higher multiplicity state which has highlighted the potential of the lanthanide-coated nanoparticles in biomedical applications and imaging.^502,503^ The first report was based on bipyridine functionalized ligands coated on 4 nm in diameter AuNP, followed by the addition of Eu(III) or Tb(III) in 1:3 ratio of Ln(III):bpy to lead to well resolved luminescence and long lifetime of 0.36 and 0.7 ms for Eu(III) and Tb(III) AuNP, respectively.^504^

**Figure 22:**
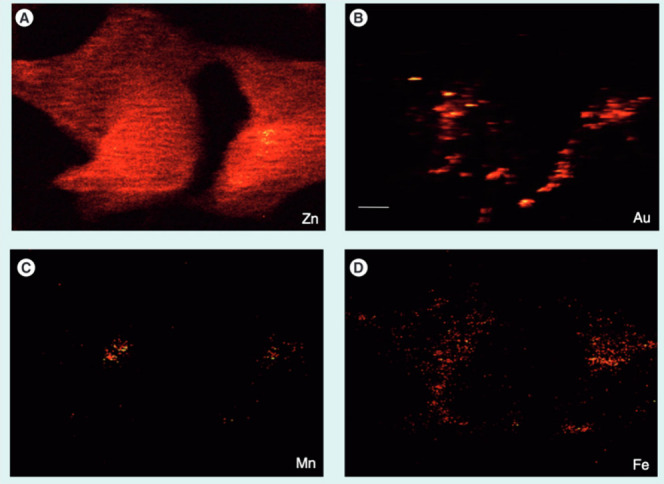
X-ray fluorescence mapping of intracellular distribution of **CeL-AuNP**_**imag1**_ in two neighboring cryofixed lyophilized MRC5VA cells. (A–D) Representative comparative elemental subcellular distributions of Zn, Au, Mn, and Fe. Scanning step-size: 200 nm; dwell time: 100 ms; the white size bar in the Au panel represents the distance of 5 μm. Reproduced with permission from ref (506). Copyright 2010 Taylor and Francis.

Pikramenou et al. introduced AuNP functionalization with isolated lanthanide complexes by titration of the lanthanide complex in citrate-based AuNP (13 nm in diameter) in water **EuL-AuNP**_**imag1**_.^505^ The complex is based on bisamide derivative of diethylenetriamine pentaacetate functionalized with thiophenols for selective anchoring onto gold. The surface plasmon resonance of the AuNP shows a 7 nm bathochromic shift upon titration of the complex, which is characteristic of surface functionalization. Purification of the AuNP was important to eliminate any luminescence from the unbound by size exclusion chromatography. The functionalized AuNP displays red Eu(III) luminescence, which is partially quenched by gold as indicated by the luminescence lifetime, which is reduced by an order of magnitude compared to the free complex (Figure 22).^505^ These particles were detected in cells by synchrotron-based X-ray fluorescence microscopy, which enabled the detection of the particles that enable ultrasensitive detection of intracellular distribution in MRC5VA cells for functionalized AuNP and PtNP. It was found that the charge of the coating complex can cause elevated levels of DNA damage detected by histone H2AX phosphorylation.^506^ The lanthanide complex design was further developed with longer “legs” to distance the lanthanide from the gold to eliminate quenching mechanisms.^507^ An Eu(III) complex with quinoline as harvesting ligand and a hexyl thioacetate group led to luminescent complexes with estimated number of 1335 Eu-complexes per AuNP and minimal luminescence lifetime quenching upon attachment onto gold **EuL-AuNP**_**imag2**_.^507^ This complex was used in responsive cell uptake as described in section 5. Surface-active cyclen lanthanide complexes have also been incorporated on AuNP (4 nm) **EuL-AuNP**_**imag3**_ and sensitized by a naphthalene β-diketone as an ancillary ligand.^508^

A peptide modified with an Eu(III) complex and a coumarin derivative for dual emission upon single excitation was attached to AuNP and delivered in HepG2 cells.^509^ The peptide is built with metalloproteinase-2 substrates for selective enzyme cleavage and displayed dual luminescence from the two probes. The Eu(III) complex is based on a sensitizing ligand previously developed for assays, BCTOT = 1,10-bis(5′-chlorosulfo-thiophene-2′-yl)-4,4,5,5,6,6,7,7-octafluorodecane-1,3,8,10-tetraone), which was attached to the N terminus of a peptide while the C terminus of the peptide was modified with the coumarin acceptor. Attachment of the peptide to AuNP resulting in stronger quenching of the Eu(III) luminescence rather than the coumarin signal, attributed to FRET to the AuNP. In the presence of one or both targeting enzymes, the substrate was cleaved and dramatic enhancement of the emission of the luminescent probes was observed.^509^ The approach has been further developed for **EuL-AuNP**_**imag4**_ monitoring protease activity in cells which allowed quantification of a protease, Caspase-3.^510^

It is worth noting that the conformations of peptides on AuNP **EuL-AuNP**_**imag5**_ affect the luminescence and binding of lanthanides.^511^ An analysis of the quenching mechanism of lanthanide probes by AuNP was performed based on Tb-streptavidin attachment to biotinylated AuNP (5–80 nm). The luminescence quenching was attributed to nonradiative dipole–dipole energy transfer based on a distance from the AuNP of 4.5 nm for the biotinylated bridge.^512^ A Dexter-type energy transfer was suggested for the mechanism of small AuNP (1–3 nm) energy transfer Yb(III) by examining different distances of Yb(III) positioning from AuNP **YbL-AuNP**_**imag6**_.^513^

Magnetic nanoparticles have been attractive for decoration with lanthanide complexes to produce nanoprobes for multimodal detection combining luminescence with magnetic resonance imaging. Novel Fe_3_O_4_ NP-conjugates present two main advantages for cell fluorescence labeling: water solubility and targeting ability. A Tb(III) DTPA-bis amide complex coated on Fe_3_O_4_ NP via catechol attachment **TbL-Fe**_**3**_**O**_**4**_**NPs**_**imag1**_ showed a strong luminescence with long lifetime.^514^ Interaction with folic acid coordinated to the NP enables targeted fluorescent imaging of HeLa cell lines with overexpressed folic receptor.^514^ Europium complexes of DTPA-bisamide functionalized with coumarin have also been attached to Fe_3_O_4_ NP **EuL-Fe**_**3**_**O**_**4**_**NPs**_**imag2**_ and shown excellent cell permeating activity in HeLa cells and strong red luminescence.^515^

Most of the upconverting nanoparticle imaging applications are associated with tissue imaging due to the attractiveness of excitation in the NIR. Recent examples of UCNP in cellular imaging are summarized. Polydopamine-coated UCNP based on NaYF_4_:Yb/Tm@NaYF_4_ conjugated with dual-targeting peptides RGD10-NGR9 were designed to target both integrin αvβ3/αvβ5 and aminopeptidase receptors in tumor cells.^516^ Under 980 nm excitation, the UCNP exhibited a strong NIR band at 802.5 nm (^3^H_4_ → ^3^H_6_), blue bands at 450 nm (^1^D_2_ → ^3^F_4_) and 475.5 nm, (^1^G_4_ → ^3^H_6_), and purple bands at 354 nm (^1^I_6_ → ^3^F_4_) and 361.5 nm (^1^D_2_ → ^3^H_6_) due to the upconversion luminescence from Tm(III). This biocompatible probe can specifically target A549 cancer cells. This was also shown *in vivo* with BALB/c nude mice bearing tumor xenografts.^516^ Upconversion submicron particles based on NaBiF_4_:Yb(III),Tm(III) exhibit a photostable, wide upconversion emission range (NIR-to-NIR and NIR-to-Vis) under 980 nm excitation. *In vitro* imaging of the UCNP in human cancer cell lines, Huh-7 and A549 as well as in bacterial strains, *E. coli*, *S. aureus*, using confocal microscopy show high cellular uptake and a high signal to background ratio.^517^ PEG-modified Sr_2_YbF_7_:0.2%Er(III), 0.8%Tm(III) UCNP showed intense red-light emission, high photostability, excellent cell membrane permeability, and low toxicity.^518^ The particles were also tested in revealing upconversion luminescence under 980 nm laser excitation at the liver of mice.

#### 3.6.2 Silica and Polymer Nanoparticles

Silica and polymer nanoparticles provide the advantage of encapsulation of luminescent agents in their network structures. Mesoporous silica nanoparticles provide a periodic-structured porous framework with the possibility of inclusion of agents in their porous structure. A highly luminescent nanosystem was based on heterometallic Ir(III)–Eu(III) complex inside mesoporous silica nanoparticles (MSN) **EuL-SiO**_**2**_**NP**_**imag1**_. These nanoparticles showed bright red luminescence with high quantum yields of 55.2% and 16.1% in powder and in water dispersion, respectively. The excitation window was extended up to 470 nm and showed uptake in living cells with confocal luminescence microscopy luminescence under excitation at 458 nm.^519^ The Ir–Eu system was also used as a donor–acceptor pair in a different approach in porous silica nanoparticles **Eu(tta)**_**3**_**L-SiO**_**2**_**NP**_**imag2**_. The nanoparticles were prepared with the two luminescent complexes incorporated in the silica structure. Efficient energy transfer from Ir(III) cyclometalated moiety to Eu(III) complex occurred in nanoparticles, which leads to the stable and intense red emission from Eu(III) complex. After incubation of HeLa cells for 30 min, intense intracellular red luminescence with high signal-to-noise ratio was observed.^520^

Encapsulation of both Tb(III) and Yb(III) complexes of *p*-sulfonatothiacalix[4]arene into silica nanoparticles (51–60 nm) led to nanoparticles with dual green and NIR luminescence **Tb/YbL-SiO**_**2**_**NP**_**imag3**_. The heterometallic nanoparticles exhibit low cytotoxicity toward human embryo and lung carcinoma cell lines. The green luminescence of the heterometallic nanoparticles reveals their internalization into human embryo cells, which opens the opportunity to use the dual visible–NIR luminescence of the nanoparticles for cellular sensing and imaging.^521^ Ultrasmall nanosilver particles (4 ± 2 nm) were also incorporated in deposited onto amino-modified silica nanoparticles (35 ± 6 nm) doped by green luminescent Tb-calixarene complex **TbL-AgNP**_**imag1**_.^522^ The confocal luminescence studies indicate that the composite nanoparticles are both adsorbed at the cell membrane and distributed within the cell cytoplasm but also produce enhanced cytotoxicity of cancer vs normal cells that makes them promising theranostic in cancer diagnostics and therapy.^522^

**Figure 23:**
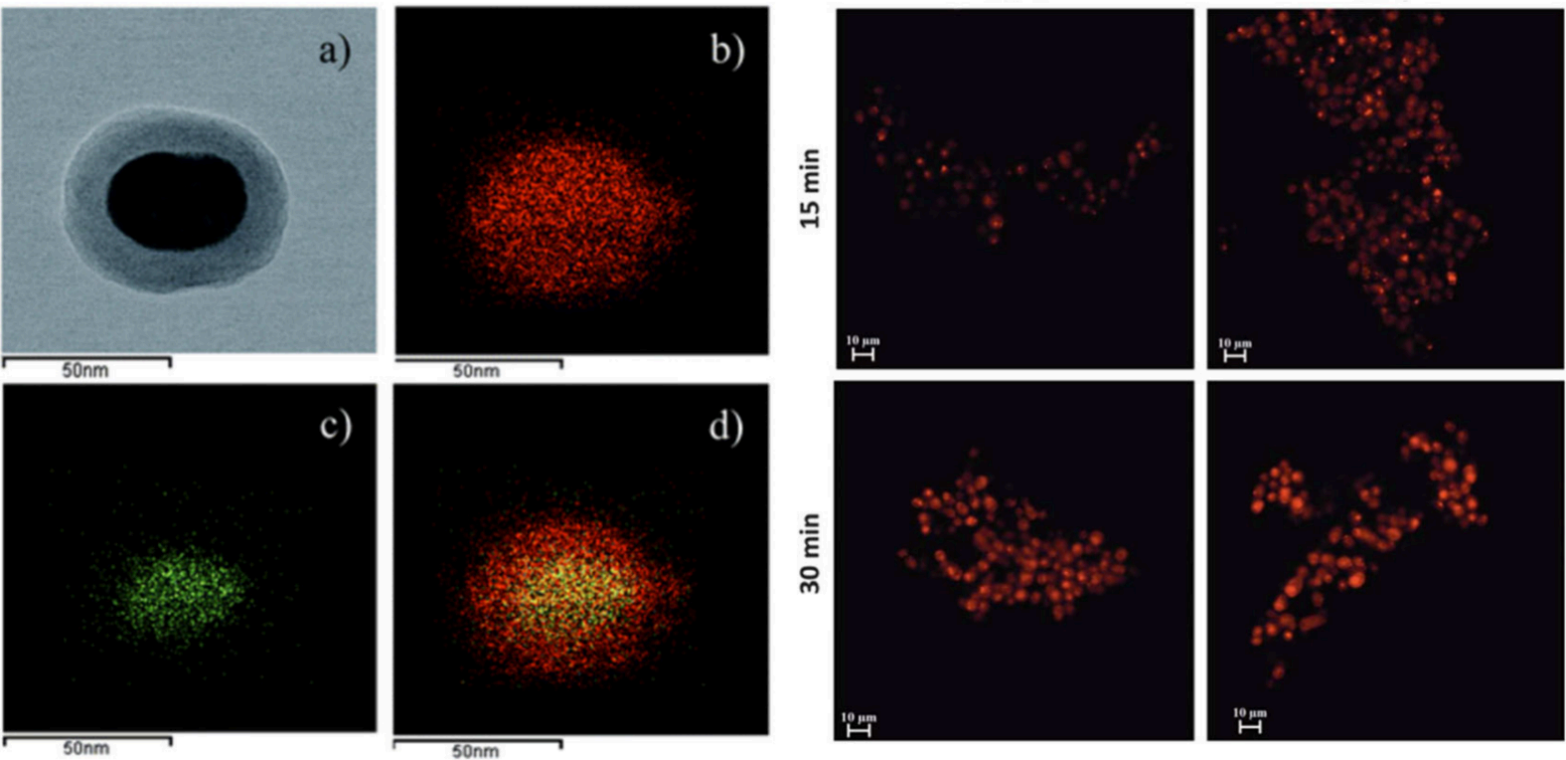
(left) (a) TEM image and (b–d) corresponding EDX mapping: (b) Si, (c) Tb, and (d) overlapping Tb and Si signals of **TbL-SiO**_**2**_**NP**_**imag6**_. (right) Confocal fluorescence microscopy images of *C. albicans* cells, stained with **TbL-SiO**_**2**_**NP**_**imag6**_ after 15 and 30 min of incubation with 10 μg mL^–1^ of each sample, at 37 °C. Reproduced with permission from ref (524). Copyright 2013 Royal Society of Chemistry.

Eu(III) emissive silica nanoparticles were obtained by grafting a pyridine-based aromatic backbone on to the silica surface **EuL-SiO**_**2**_**NP**_**imag4**_.^523^ The nanoparticles were rapidly and efficiently taken up by RAW 264.7 cells.^523^ Encapsulation of the picolinate Tb(III) complex K_2_[Tb_2_(pic)_8_]·7H_2_O in silica particles through a reverse microemulsion process led to development of core–shell nanoparticles with a silane amino functionalized shell **TbL-SiO**_**2**_**NP**_**imag6**_. It was found that quaternization of the amino groups with methyl iodide was needed amino in order to increase their water-stability and promote uptake by cells (Figure 23). Confocal fluorescence microscopy showed the selective uptake in *Candida albicans* cells.^524^

In a recent approach, europium(III) polyhedral complexes Eu_8_L_12_ were embedded in mesoporous nanoparticles **EuL-SiO**_**2**_**NP**_**imag7**_.^525^ The hybrid materials displayed increased quantum yields of luminescence compared to the polyhedral complexes, and the biotin-functionalized nanoparticle exhibited much enhanced fluorescence-imaging in MDA-MB-231 human breast cancer cells, with significantly reduced dosage of the complex.^525^ Lanthanide diketonates have also been linked onto silica nanoparticles via ancillary ligand coordination **EuL**_**3**_**-SiO**_**2**_**NP**_**imag8**_.^526^ In one approach, a derivative of 1,10 phenanthroline was attached onto silica nanoparticles, and an [Eu(tta)_3_(H_2_O)_2_] complex was coordinated onto the particles **EuL-SiO**_**2**_**NP**_**imag9**_. The particles show low cytotoxicity and good biocompatibility and showed uptake in HeLa cells by confocal fluorescence imaging.^527^

**Figure 24:**
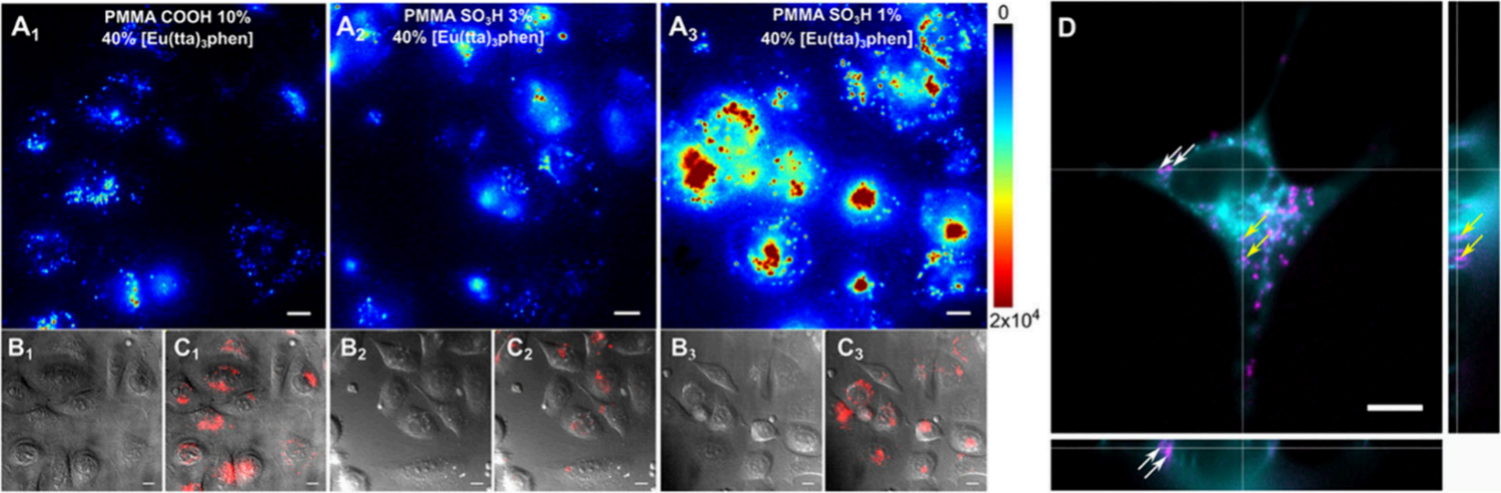
Live-cell images of HeLa cells incubated with **Eu-PMMA**. (A) Time-gated photoluminescent images with PMMA-COOH 10% (A1), PMMA-SO3H 3% (A2), and PMMA-SO3H 1% (A3) NPs at 40% [Eu(tta)_3_phen] loading. For a better comparison between the different images, the intensity scale was fixed at 0 to 2 × 104 counts. (B) Differential interference contrast (DIC) images. (C) Overlay of images from A and B (ECP-NP PL is shown in red, PL intensities were normalized to the highest values in A1, A2, and A3). (D) Projections of a z-stack of images of a HeLa cell incubated with **Eu-PMMA** (PMMA-COOH 10% at 40% [Eu(tta)_3_phen]). ECP-NPs are shown in magenta and the cell membranes in turquoise (costained with DiD). Large image: x/y-optical section at 6 μm from the surface; small images are optical sections of cell along the x- and y-axis of the z-stack with a x/z side view (bottom) and y/z side view (right). Lines in the images indicate positions of the sections; white and yellow arrows point to ECP-NPs in endosomes/lysosomes. Scale bars in all images: 10 μm. Reproduced with permission from ref (528). Copyright 2019 American Chemical Society.

[Eu(tta)_3_(phen)] complex has been incorporated into poly(methyl methacrylate)-based particles **Eu-PMMA** of 10, 20, and 30 nm size. The resulting particles contain up to 5000 complexes and a quantum yield of ≥ 0.2. They were uptaken in HeLa cells, and the confocal luminescence images were obtained using low illumination intensities and acquisition times (Figure 24).^528^

Encapsulation of a Eu-diketonate complex, [Eu(tta)_3_(dpbt)] (tta = thenoyltrifluoroacetonato, dpbt = 2-(N,N-diethylanilin-4-yl)-4,6-bis(3,5-dimethylpyrazol-1-yl)-1,3,5-triazine) in the hydrophobic cores of water-dispersible and biocompatible nanoparticles of poly(methyl methacrylate-*co*-methacrylic acid) **EuL-PMMA-***co***-MAA** led to highly luminescent particles which could be functionalized with an anti-EGFR monoclonal antibody. The antibody-functionalized nanoparticles were studied by two-photon-excitation luminescence imaging for target specific localization in live MDA-MB-231 cancer cells.^529^

Micelles based on amphiphilic block copolymer (**PDKMA-***b***-POEGMA**) polymers with β-diketone chelating units were developed for binding to Gd(III) and Eu(III). The sizes of lanthanide hybrid micelles are in the range of 87 and 222 nm and showed good uptake in MCF-7 cells. Confocal laser scanning microscopy was employed with an excitation wavelength of 405 nm and detection of Eu(III) emission at 620 nm. Low cytotoxicity and excellent cellular uptake demonstrate the potential of these particles in biomedical imaging, especially suitable for assessing thin biopsies in confocal imaging.^530^

Water-dispersible Eu(III)-based nanoprobes were prepared by reversible addition–fragmentation chain transfer polymerization-induced self-assembly of hydrophobic monomers [(Eu(III)-containing monomer bearing phenanthroline, TTA, and acrylic acid ligands with methyl methacrylate (MMA)] using hydrophilic macro-chain transfer agent poly(PEGMA)-CTA. The resulted **Eu-polymer** nanoprobes showed spherical in shape in good monodispersity with average diameters of around 210 nm high aqueous stability and good luminescence properties with quantum yields of 37.21% and fluorescence lifetime of 312.4 μs. Moreover, the poly(PMEu) nanoprobes exhibited good cellular biocompatibility with cell viabilities of 88.2% and high fluorescence intensity for *in vitro* cellular imaging.^531^ Polymer dots (Pdots) based on semiconducting polymer poly(9-vinylcarbazole) were used as the host matrix for Eu complexes **EuL-PVK-1** and **EuL-PVK-2**.^532^ Not only did PVK function as a host matrix to disperse the Eu complexes and reduce the self-quenching of the Eu complexes, but it also acted as an efficient fluorescence energy donor with a large absorption cross-section to transfer energy to the Eu complexes. The Eu complex-Pdots showed much higher emission brightness than pure Eu complex nanoparticles and their localization in MCF-7 cells was studied by **EuL-PVK-1** confocal and time gated luminescence imaging.^532^

## 4 Lanthanide Probes in Tissue Imaging

Optical probes for tissue imaging are increasingly important in technological developments for disease diagnosis, local therapeutic treatment, and optical-guided surgery approaches. Common challenges for luminescent probes include the tissue penetration and transmittance of incident and detection light, and biocompatibility for administration.^4,533,534^ The second NIR window (NIR-II, 1000–1700 nm) is clearly advantageous for tissue imaging, and NIR-IIb (1500–1700 nm) is increasingly important with the development of intraoperative imaging in surgical procedures and for enabling multiplex detection with different channels for *in vivo* imaging. Additionally, high spatial-temporal resolution is particularly important during surgical procedures, for example, in visualization of small-size tumors; the size of nanoparticles as well the strength of signal output are particularly important factors. Lanthanide-based luminescence provides obvious advantages for imaging, primarily NIR emission range lanthanides, with scope for visible emitting lanthanides more limited. Recently, Cherenkov radiation mediated sensitization applications were introduced by Boros et al. as alternative *in situ* excitation mechanisms.^535^ UCNP and down-converting nanoparticles (DCNP) dominate examples and are ideal with lanthanide combinations that allow NIR excitation and detection to NIR, although the availability of lanthanide designs for detection over 1500 nm to avoid scattering by the biological tissue following NIR excitation is limited.^536^ There have been many approaches to designs of nanoparticles to improve efficiency for detection in NIR(II) window.^537^ The response of lanthanide-doped nanomaterials to local temperature changes has attracted a lot of interest and the different mechanisms to explain their function have been recently reviewed and not included in this review.^538^ In this section, we have reviewed the systems from molecular lanthanide complexes to polymetallic systems and nanoparticles (Table 9) to provide an overview of the designs, requirements, and challenges for tissue imaging. Several systems which report a responsive or therapeutic effect with imaging are reported in Section 5.

**Table 9:**
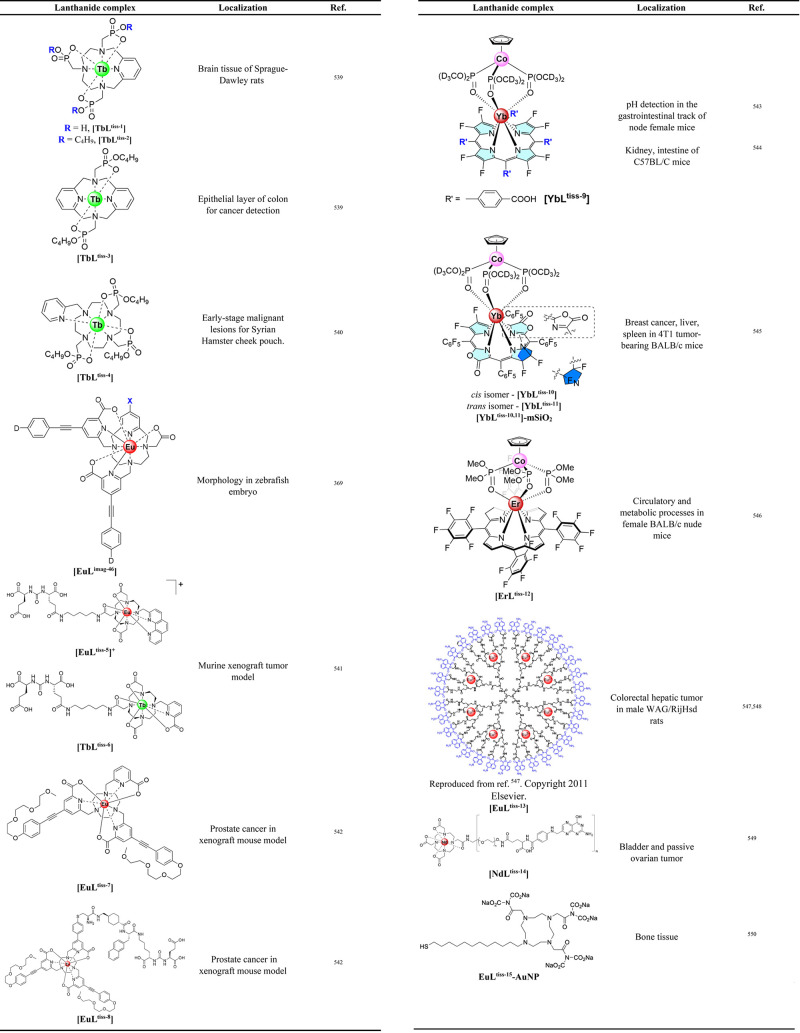
Lanthanide Complexes and Polymetallic Constructs in Tissue Imaging

### 4.1 Macrocyclic Probes

Luminescent macrocyclic probes for cell imaging were suggested in 1995^551^ for lanthanide cryptates, although the first images were obtained for Tb(III) macrocyclic complexes with azacrown ether derivatives featuring fused or substituted pyridines as sensitizing groups.^539^ The chelates [**TbL**^**tiss-1**^**]**, **[TbL**^**tiss-2**^**]**, and **[TbL**^**tiss-3**^**]**, have sizable quantum yields in water, 21, 48, and 40%, respectively, upon excitation in the range 260–270 nm, and **[TbL**^**tiss-1**^**]** has a lifetime of 2.7 ms. Biodistribution experiments conducted with **[**^**153**^**SmL**^**tiss-1**^**]**, and **[**^**153**^**SmL**^**tiss-3**^**]**, evidence a preferential localization in the bone tissue of Sprague–Dawley rats after venous tail injection, which allows luminescence imaging of the rat femur.^539^ This prompted the construction of an endoscopic spectrometer for this type of imaging. On the other hand, the more lipophilic **[TbL**^**tiss-2**^**]** preferentially localizes in the epithelial layer of the colon. Moreover, carcinogenic cells have greater affinity for **[TbL**^**tiss-2**^**]** compared to normal cells. **[TbL**^**tiss-2**^**]** was therefore introduced into the intestine of rats having colon cancer through a microendoscope, which allowed to clearly evidence a suspect mass in the colon.^552^ Later, the authors switched to the cyclen framework,^553^ grafting it with a pyridine antenna to give the luminescent contrast agent **[TbL**^**tiss-4**^**]** with a long terbium lifetime of 3.5 ms. It was used for the detection of early stage malignant lesions in Syrian hamster cheek pouch.^540^

**Figure 25:**
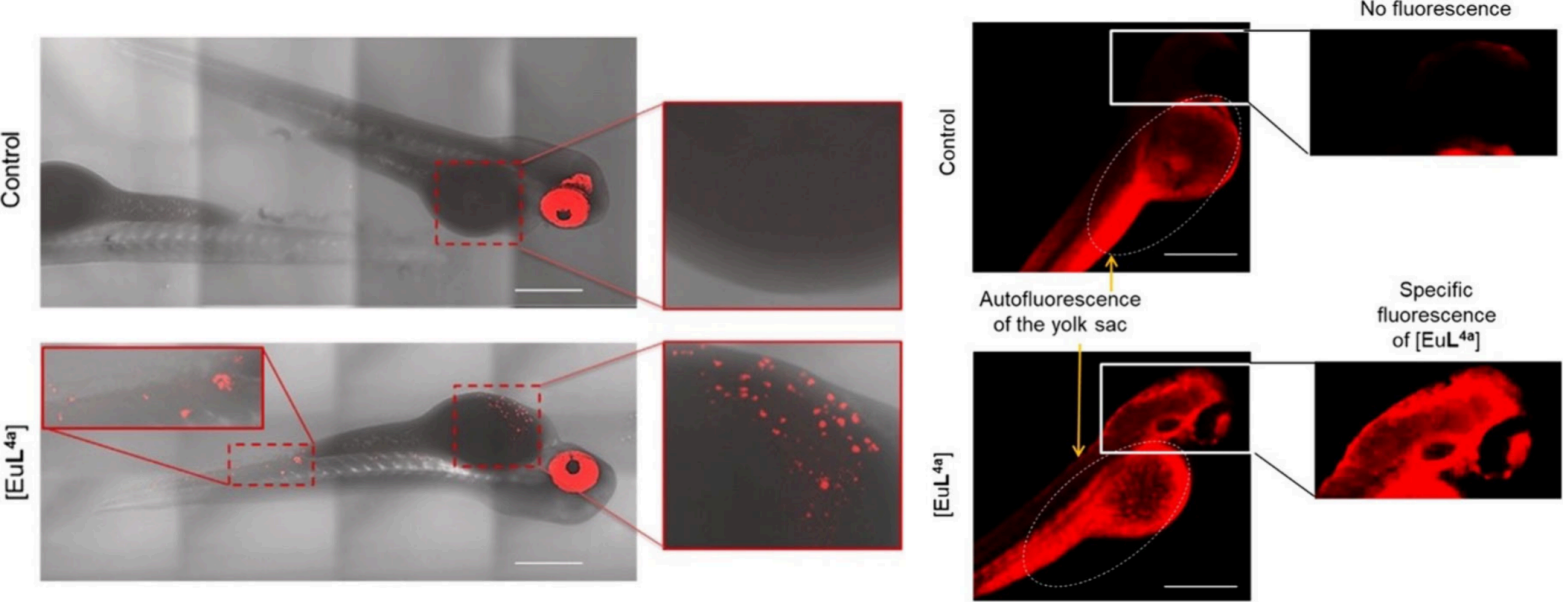
Confocal imaging studies of zebrafish embryos injected with **[EuL**^**imag-46**^**]** (10^–4^ M) and their control images with no optical probes under single (top) and two photon excitation (bottom), Scale bar: 500 μm. Reproduced with permission from ref (369). Copyright 2020 American Chemical Society.

**[EuL**^**imag-46**^**]**, which was successful in cellular imaging, was also studied as an imaging probe in zebrafish. Zebrafish embryos are frequently used for *in vivo* studies due to their genetic similarities with humans and their transparent tissues.^554,555^ Preliminary toxicology study with **[EuL**^**imag-46**^**]** on two lines of zebrafish, the wild-type AB and the Casper line reveal no significant modification in the morphology and the vitality of the embryos was detected after early stage **[EuL**^**imag-46**^**]** injection. The head of the zebrafish, the dorsal vascular network, and additional punctuation in the yolk sac were clearly observable in comparison to the control zebrafish embryos which only show yolk sac due to autofluorescence (Figure 25).^369^ In a two-photon excitation experiment, 3D reconstructed images obtained upon incubating **[EuL**^**imag-46**^**]** to zebrafish embryos reveal punctuation that corresponds to a vascular capillary section. Only a few slices were imaged due to the very large size of the animal model and the highly localized two-photon excitation. Images based on autofluorescence reveal only the eye in control embryos (Figure 25). These studies are particularly promising because of the high quality of images obtained and the low toxic profile.

**Figure 26:**
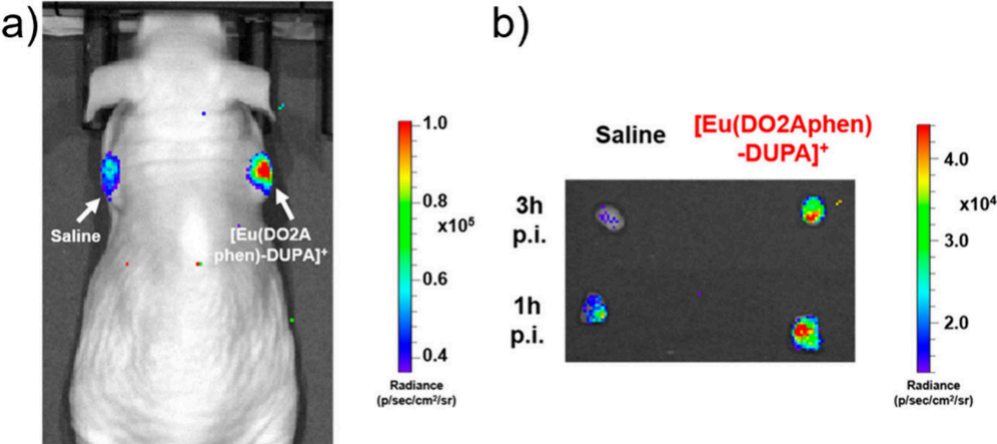
(a) *In vivo* optical imaging upon administration of [^18^F]-fluorodeoxyglucose. [^18^F]-FDG and **[EuL**^**tiss-5**^**]**^**+**^. (b) Excised tumors imaged at 1 and 3 h postinjection (p.i.) demonstrate the persistent, detectable emission signal of **[EuL**^**tiss-5**^**]**^**+**^. Adapted with permission from ref (541). Copyright 2021 American Chemical Society.

Boros et al. demonstrated that excitation of discrete Tb(III) and Eu(III) complexes in deep tissue can take place in situ via Cherenkov radiation emitted by clinically employed radioisotopes, overcoming the problem of UV light penetration.^535,556^ Cherenkov radiation is emitted by radioactive isotopes that decay by the emission of a charged particle with an energy greater than the threshold energy of 264 keV in water.^557^ Radionucleotides such as ^18^F and ^89^Zr, which are used for positron emission tomography imaging, have shown energy transfer to lanthanide and the Cherenkov radiation energy transfer is elucidated.^535,556^ The ligand designs are based on a macrocyclic core for lanthanide binding site, picolyl or phenathroline antenna sensitizers and a pendant coordination site for ^89^Zr.^535^*In vivo* imaging of optimized probes **[EuL**^**tiss-5**^**]**^**+**^ (Φ = 10%), **[TbL**^**tiss-6**^**]** (Φ = 38%) bearing a peptide chain was successful upon administration of [^18^F]-fluorodeoxyglucose.^541^ The dipeptide, 2-[3-(1,3-dicarboxypropyl)ureido]pentanedioic acid was designed to target the prostate-specific membrane antigen which is highly overexpressed in metastasizing prostate cancer cells, while the [^18^F]- fluorodeoxyglucose targeting GLUT-1, overexpressed in most cancer subtypes, was used to serve as an *in situ* Cherenkov radiation source. The complexes **[TbL**^**tiss-6**^**]** and **[EuL**^**tiss-5**^**]**^**+**^ were first studied *in vitro* to explore tissue penetration and then were successfully tested in mice (Figure 26). **[EuL**^**tiss-5**^**]**^**+**^ displayed strong signal where the Tb-signal was attenuated by the tissue.^541^

More recently, lanthanide complexes based on the TACN macrocycle were introduced with optimized luminescence properties, due to the incorporation of extended π-conjugated systems with picolyl-functional groups, **[EuL**^**tiss-7**^**]** (Φ = 76%), **[EuL**^**tiss-8**^**]** (Φ = 24%).^542^ The complex **[EuL**^**tiss-8**^**]** is functionalized with a targeting vector composed of the peptide cysteine-cyclohexyl-naphtyl-KuE, a peptide sequence previously validated for the targeting of the prostate-specific membrane antigen. In live PC-3 PiP cells, **[EuL**^**tiss-8**^**]** is observed localized in the cell membrane, in line with the localization of the transmembrane protein, while **[EuL**^**tiss-7**^**]** does not display any accumulation in cells. *In situ* excitation of Eu(III) emission produced by a ^68^Ga complex coordinated to prostate-specific membrane antigen resulted in 5-fold optical signal amplification and enables the resection of tumor tissue in live mice.^542^ The approach shows the powerfulness of Cherenkov radiation sensitization of lanthanides in imaging but also for potential therapeutic effects. Challenges for further optimization are based on the sensitization of the lanthanide emission and the pharmacokinetics of the complexes.

**Figure 27:**
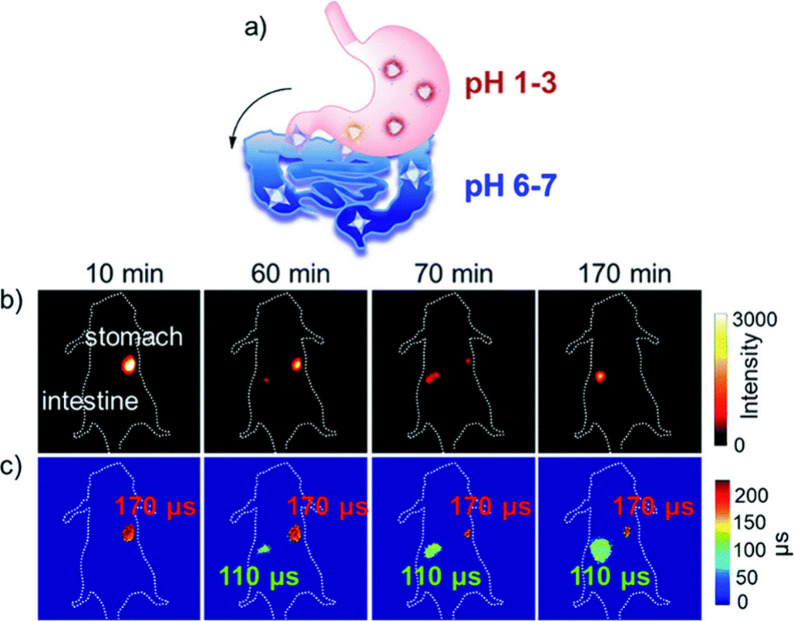
(a) Schematic illustration of the metabolic process of **[YbL**^**tiss-10**^**]** from the stomach to the intestine. (b) NIR fluorescence intensity imaging (exposure time, 25 ms) of the Yb(III) complex. (c) FLIM images (exposure time, 250 ms). λ_ex_, 532 nm; λ_em_, 1000 nm long-pass. Reproduced with permission from ref (544). Copyright 2019 Royal Society of Chemistry.

Exploiting the previous success in using β-fluorinated Yb(III) porphyrins for cellular imaging,^385^ Li, Zheng et al. developed a series of pH responsive Yb(III) porphyrins. These were used for gastrointestinal pH detection^543^ based on time-resolved fluorescence lifetime imaging (FLIM) in the near-infrared region, 900–1700 nm. This FLIM approach does not only allow a deep tissue penetration depth, but also offers the unique benefit of the quantitative visualization of molecular events *in vivo* and is independent of local luminescence intensity and fluorophore concentration. Fluorinated β-porphyrins with aryl carboxylate **[YbL**^**tiss-9**^**]** were ideal candidates based on their high quantum yield, deep penetration in tissues, and pH sensitivity.^544^**[YbL**^**tiss-10**^**]** has a p*K*_a_ of 6.48, which shows pH response between pH 5 to 9 with decreasing luminescence intensity (Figure 27). Intracellular pH sensitivity was explored on HeLa cells using chloroquine as an apoptosis inducer. However, it was difficult to distinguish intracellular pH variations using **[YbL**^**tiss-9**^**]**. This complex accumulates on the lysosome of the cell. Testing **[YbL**^**tiss-9**^**]***in vivo* using FLIM mode reveals distinct lifetime changes in stomach (pH 1–3, τ = 170 μs) and intestine (pH 6–7, τ = 110 μs) which was difficult to observe in fluorescence intensity imaging.^543^ The Antiacid metabolism in the stomach as well as changes in the pH of the stomach before and after fasting was visualized using FLIM technique. Therefore, these results indicate that this NIR fluorescent lifetime probe can realize the real-time and reversible monitoring of gastrointestinal pH fluctuations *in vivo*.^558^

Although **[YbL**^**tiss-9**^**]** shows lower brightness than commercial NIR fluorophore Cy5.5, this β-porphyrin shows strong resistance to photobleaching. *Ex vivo* biodistribution studies reveal that [**YbL**^**tiss-9**^**]** is mainly accumulated in the kidney and intestine, matching the *in vivo* luminescence images.^544^ These indicate that the clearance routes of Yb(III) complexes were through both hepatobiliary and renal systems, like most organic fluorophores. Zhang, Sessler et al. investigated the possibility of using regioisomerization to switch between diagnosis and therapeutic approaches selectively.^545^ This was achieved with perfluorinated porphodilactone regioisomers **[YbL**^**tiss-10**^**]**, *cis* isomer and **[YbL**^**tiss-11**^**]**, *trans* isomer whose triplet energy level was modulated with different isomers because of reducing π-conjugation. This forms the chemical basis for switching from imaging to photodynamic therapy functions via the selection of the appropriate Yb(III) isomer.^545^ In NIR emission spectra, **[YbL**^**tiss-10**^**]** has high emission intensity while **[YbL**^**tiss-11**^**]** was relatively nonemissive. This suggests that 1,3-dipolar cycloadditions serve to increase the extent of β-pyrrolic saturation, thus giving rise to chlorin-type photophysical properties. For biological applications, both Yb(III) complexes were modified to bear positive charges via methylation of the pyrrolidine ring with iodomethane.^545^*In vivo* studies in 4T1 tumor-bearing BALB/c mice were performed both with the complexes as separate compounds and as mixture (*cis*/*trans* = 2:1, n/n, **[YbL**^**tiss-10,11**^**]-mSiO**_**2**_ encapsulated in mesoporous silica nanoparticles (mSiO_2_) (100–200 nm). MSNs exclude interference from systemic effects which could vary as a function of regioisomerism. MSNs can cocomplex both the isomers and act as carriers for both species. This avoids the dispersion of the isomers *in vivo* while ensuring that the proportion of isomers reaching a biological target reflects those that were initially administered. **[YbL**^**tiss-10,11**^**]**-**mSiO**_**2**_ displayed tumor suppression upon light irradiation suggesting that this regioisomeric mixture could possibly have a role to play as an integrated theranostic cocktail.^545^

Wang, Zhang, Bünzli et al. reported a Er(III)-bacteriochlorin NIR probe **[ErL**^**tiss-12**^**]** to operate in the NIR window for excitation and emission, enabling multiplexed imaging in visualization of circulatory and metabolic processes in living mice and tracking cancer cell metastases in mouse brain.^546^ Using a phospholipid micelle formulation, this complex primarily accumulates in the liver and spleen at 24 h postinjection, with little retention observed in other organs, including lung, heart, kidney, muscle, bone marrow, and brain. **[ErL**^**tiss-12**^**]** micelles with the protection of surface polyethylene glycol showed an extremely long blood circulation time of *t*_1/2_ ≈ 12 h. This allowed fluorescence imaging of cerebral vascular structures and the closely spaced femoral artery and vein in an unhurried time window.^546^ The unique spectral characteristics of **[ErL**^**tiss-12**^**]** enables robust excitation or emission wavelength-based multiplexing. The **[ErL**^**tiss-12**^**]** complex was conjugated to the cell-penetrating peptide HIV-TAT to study cancer cell metathesis using NIR epifluorescence microscopy. The two channels of detection were based on the ligand fluorescence observed in the short-wavelength region (800–1000 nm) and Er(III) luminescence in the long-wavelength region (1400–1600 nm). Cancer cells were labeled with CT1530 and injected intracardially into a mouse model to simulate brain metastases. The dual emission allowed multiplexing by monitoring two channels and identification of the cells in the brain channel.^546^

### 4.2 Polymetallic Probes

Lee, Petoud, Brown et al. explored the use of lanthanide-dendrimers for *in vivo* luminescence imaging of colorectal cancer.^547,548^ A generation-3 dendrimer, composed of 32 amino end branches functionalized with 4-amino-1,8-naphthalimide sensitizers using glycine linkers, chelate eight Eu(III) centers, formed **[EuL**^**tiss-13**^**]**.^547,548^ The absorption spectrum of **[EuL**^**tiss-13**^**]** reveals a maximum at 440 nm but absorption at 630 nm, an attractive wavelength for bioimaging, with a molar absorption coefficient of 5000 M^–1^ cm^–1^.^547,548^ The dendrimer was infused on six-week-old male WAG/RijHsd rats. *Ex vivo* imaging confirms the high intensity luminescence signal at 610 and 740 nm on tumors only seconds following administration. *In vivo* intrahepatic infusion of **[EuL**^**tiss-13**^**]** in anesthetized rats provides consistent results from *ex vivo* settings. Two-photon absorption scanning microscope reveals that the luminescence response originated within the vasculature of the liver. Higher magnifications reveal the luminescence location outside the vessels in the perivascular space. The relatively small size of this dendrimer^559^ facilitates its exit through the fenestrae and allows it to be trapped in the extravascular spaces of the tumor. Confocal microscopy observations are consistent with the hypothesis that **[EuL**^**tiss-13**^**]** has increased extravasation from the leaky tumor vasculature and, therefore, is more likely to be trapped in the perivascular spaces of the tumor.^547,548^ This work, therefore, provides a platform for *in vivo* imaging of tissues using dendrimers functionalized with Ln metals.

Another approach to polymetallic assemblies was introduced with the conjugation of [NdDOTA]^−^ with polyethylene glycol polymer functionalized with folic acid, **[NdL**^**tiss-14**^**]** for NIR detection in tissues.^549^ Upon excitation of the 0.54 kDa polymer at 808 nm, the characteristic Nd emission appears at 1060 and 1330 nm. *In vivo* pharmacokinetics suggest strong uptake in the bladder after 20 min post injection which lasted beyond 100 min. **[NdL**^**tiss-14**^**]** showed higher NIR-II imaging quality than that of clinically approved NIR probes (indocyanine green) with deep tissue penetration of 7 mm. **[NdL**^**tiss-14**^**]** showed rapid passive ovarian tumor uptake, capable of identifying tumors within 30 min postinjection, resulting in the discrimination between malignant and normal tissue types under surgical resection.^549^

Near-infrared luminescence and photoacoustic imaging attract attention for real-time monitoring of biological samples owing to high sensitivity, resolution, and pronounced signal detection depth resolution.^560,561^ Eliseeva, Petoud et al. reported a proof-of-concept study with a ternary complex formed with the anionic Yb(III) tetrakis(2-thenoyltrifluoroacetonate) and the cationic NIR-absorbing chromophore, 1,1′-diethyl-2,2′-dicarbocyanine, encapsulated inside 100 nmNH_2_-functionalized polystyrene (PS/NH_2_) nanoparticles.^562^ NIR and photoacoustic imaging experiments were carried out using specially designed phantoms mimicking blood vessels and biological tissues. Scattered Yb(III) luminescence was detected through a 3 mm layer of tissue mimicking media. A sharp photoacoustic signal arising from the tube filled with an aqueous suspension of the Yb(III) complex @PS/NH_2_NPs was clearly distinguished by superposition of ultrasound and photoacoustic images obtained upon excitation at 720 nm.^562^

Detection in the visible of microdamaged regions of bone tissue was achieved with two-photon excitation of a gold nanoparticle system functionalized with **EuL**^**tiss-15**^**-AuNP**, where the **L**^**tiss-16**^ bears three disodium iminodiacetate pendant arms for Ca(II) binding and an alkyl thiol group that enables attachment to gold.^550^ The luminescence of the nanoparticles is “switched on” within the physiological pH window via the addition of the β-diketone antenna sensitizer, 4,4,4-trifluoro-1-naphthalen-2-yl)butane-1,3-dione (nta), by formation of ternary complex species. The 3.5 nm **EuL**^**tiss-15**^**-AuNP** showed that nanoagents can be used *in vitro* and *in vivo* for the visualization of damage in bone tissue and analysis of microcrack formation.

### 4.3 Nanoparticles

This section is organized based on the detection process, from initial light excitation to the final signal which is monitored in imaging, which are summarized in Table 10. DCNP are particularly noted for NIR excitation to NIR luminescence.

**Table 10:** Nanoparticles in Tissue Imaging

Nanoparticles	Localization	Ref
Eu_2_O_3_ NPs, 7 nm	Lymph node and tumor	(563)
NaY(Gd)F_4_:3%Er@NaY(Gd)F_4_	Organs in deep tissue	(564)
NaYF_4_:Yb30, Er6@NaYbF_4_@NaYF_4_:Nd40	Adipose tissues	(565)
**DCNP:** NaGdF_4_@NaGdF_4_:Yb/Ln@NaYF_4_:Yb@NaNdF_4_:Yb (Ln = Er, Ho)	MCF-7 and BT-474 tumors	(566)
**DCNP**: NaYbF_4_:2%Er,2%Ce@NaYF_4_	Mouse brain	(567)
**DSNP**: α-NaYF_4_@NaErF_4_:Ce@NaYbF_4_@NaErF_4_:Ce@NaYF_4_	Mouse tumor	(568)
**DSNP**: β-NaErF_4_:2%Ce@NaYbF_4_@NaYF_4_	Mice	(569)
**DCNP**: NaYF_4_@NaYbF_4_:Er,Ce@NaYF_4_:Ca	Mice cerebral vasculature	(570)
NaYbF_4_:Gd,Er,Ce@NaYF_4_	Brain tumor	(571)
**DSNP**: NaLuF_4_:40Gd/20Yb/2Er modified with poly(acrylicacid)(PAA)-modified and doped with 5% Ce(III)	Small tumor and metastatic tiny tumor	(572)
**DSNP**: NaYF_4_:Yb_0.8_/Tm_0.08_@NaYbF_4_@NaYF_4_	Mouse subcutaneous tissue and ischemic stroke model	(573)
**DCNP**: NaYbF_4_:Ln(III)@NaYbF_4_@NaYF_4_	Lymph, blood vessels and intestines	(574)
**UCNP**: NaErF_4_:Yb@NaYF_4_:Yb	Hypothermia mouse brain tissue	(575)
**UCNP**: (α-NaYbF_4_:Tm(III))/CaF_2_	Imaging in rat femoral bone and thick pork tissue	(576)
**UCNP**: NaYF_4_:20% Yb, 4% Tm	Mouse liver tissue	(577)
**UCNP**: NaYb_0.8_Er_0.2_F_4_	Mice mammary fat pads	(578)
**UCNP**: NaYF_4_@NaYbF_4_@NaYF_4_:Yb/Tm@NaYF_4_	Liver and two abdomen subcutis in Kunming mice	(579)
**UCNP**: NaYF_4_:Yb,Er@NaYF_4_:Nd	Acidic tumor	(580)
**DSNP**: Na_3_CrF_6_:X@Na_3_CrF_6_ (X = Er(III), Nd(III))	Stomach and gastric tumor	(581)

#### 4.3.1 Downshifting Light Conversion Systems

Cerenkov luminescence was introduced to excite a Eu_2_O_3_ down conversion nanoparticle with 300–450 nm light.^563^ Ultrasmall Eu_2_O_3_ nanoparticles (7 ± 2 nm) coated with polyethylene glycol were developed and injected in mice intravenously to visualize the lymph node and tumor.^563^ An approach to replace optical illumination with X-ray activation of lanthanide luminescence was also introduced by Fan and Zhang et al. for core–shell nanoparticles.^564^ NaYF_4_ or NaGdF_4_ was chosen as a low phonon energy material with Nd(III), Ho(III), Tm(III), or Er(III) as the activators. The particles display characteristic NIR-II luminescence at 1064 nm for Nd(III), 1180 nm for Ho(III), 1475 nm for Tm(III), and 1525 nm for Er(III).^564^ The feature of these particles is the persistent luminescence signal after illumination. The Er-nanoparticles were injected in live mice via the abdominal vein; after 10 s, clear images of the abdominal vascular network (imaging depth ∼1–2 mm) were obtained via NIR-II luminescence imaging with visualization of many tiny capillary vessels. Additionally, multiplexed *in vivo* imaging using Nd- and Er-nanoparticles was successful to probe and differentiate multiple organs in deep tissue.^564^

QD Liposome-coated nanoparticles NaYF_4_:Yb30,Er6@NaYbF_4_@NaYF_4_:Nd40 were prepared to evaluate a sensitive detection method for thermogenic adipose tissues, which are important in the diagnosis of metabolism disorders and to examine efficiency of detection in vascular imaging, and lymph node localization biopsy using NIR-II imaging detection. Liposomes were selected in order to increase the ability of excretion from the body. These nanoparticles were selected with multiple emission wavelengths to examine their multifunctional imaging under single excitation. The particles display emission with three bands at 1000–1100 nm (NIR-II), 1300–1350 nm (NIR-IIa), 1500–1700 nm (NIR-IIb) simultaneously under an 808 nm excitation.^565^ They were studied *in vivo* showing accumulation in brown adipose tissues. The nanoparticles cleared from the body within 7 days after intravenous administration and imaging showed higher resolution imaging of the longer wavelength detection.^565^

A novel approach allowing multiplex detection with down-converting nanoparticles DCNP has been introduced by using luminescence lifetime signals.^566^ Nanoparticles consisting of the outer layer doped with Nd(III) sensitizers and Yb(III), an intermediate layer doped with Yb(III) only, and inner layer with codoped with Yb(III) and Er(III) emit at 1550 nm upon 808 nm excitation, following a series of energy transfer steps from Nd(III) to Yb(III) and finally Er(III). The studies show that selected lifetimes from the NIR-II nanoparticle probes are resolved at depths of up to 8 mm in biological tissues, with signal-to-noise ratio compared to signal intensity measurements. It is shown that luminescence lifetime can be used for detection independent of tissue penetration depth and *in vivo* multiplexing based on lifetime coding is introduced to identify tumor subtypes in living mice.^566^

**Figure 28:**
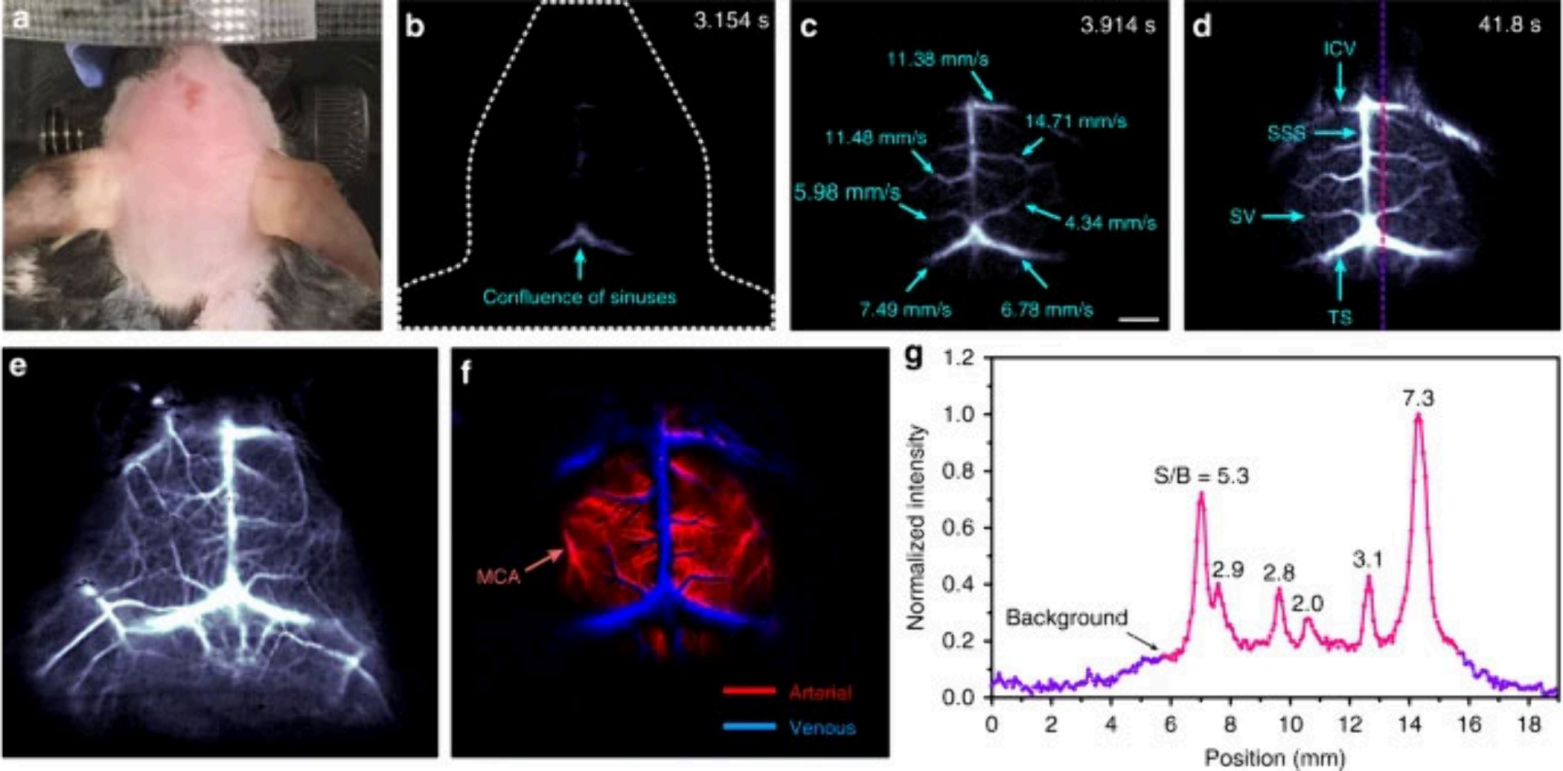
Fast *in vivo* brain imaging with NaYbF_4_:2%Er,2%Ce@NaYF_4_ coated with PMAh, PEG in the NIR-IIb region. (a) Color photograph of a C57Bl/6 mouse (with hair shaved off) preceding NIR-IIb fluorescence imaging. (b–d) Time-course NIR-IIb brain fluorescence images (exposure time: 20 ms) showing the perfusion of RENPs into various cerebral vessels. The blood-flow velocities of cerebral vessels are given in (c) (scale bar corresponds to (b–d): 2 mm). (e, f) Cerebral vascular image (exposure time: 20 ms) in NIR-IIb region with corresponding PCA overlaid image (f) showing arterial (red) and venous (blue) vessels. (g) SBR analysis of NIR-IIb cerebrovascular image (d) by plotting the cross-sectional intensity profiles. Reproduced with permission from ref (567). Copyright 2017 Springer Nature.

The challenge with DCNP based on Er-doping is the competing upconverting emission pathways. It was found that Ce-doping suppresses the upconversion pathway in DCNP with an Yb core, NaYbF_4_:2%Er,2%Ce@NaYF_4_ and poly(maleic anhydride-*alt*-1-octadecene) PMAH/polyethylene glycol (PEG) coating achieving enhanced down-conversion by ∼9-fold for 1550 nm luminescence under 980 nm excitation.^567^ These bright DCNP were successful in *in vivo* mouse brain vessel imaging and could facilitate (Figure 28) fast imaging of arterial blood flow in the mouse brain using short exposure times (20 ms).

**Figure 29:**
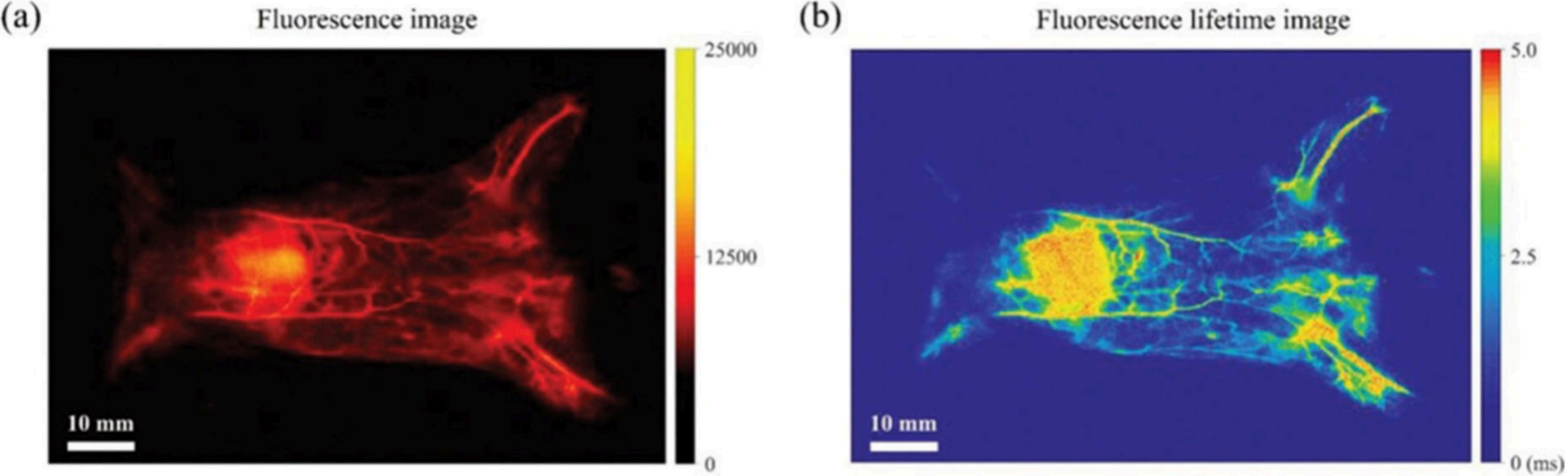
Comparison of fluorescence intensity image with fluorescence lifetime image of whole-body vascular in mice detected by Er-fluorescence signal. Reproduced with permission from ref (569). Copyright 2023 John Wiley and Sons.

Lifetime multiplexed imaging was also achieved by a series of Er(III) doped double interface fluorescent nanoprobes: α-NaYF_4_@NaErF_4_:Ce@NaYbF_4_@NaErF_4_:Ce@NaYF_4_ using Yb(III) as sensitizer and Er(III) as emitter.^568^ The design enabled double interface energy transfer and minimized the unfavorable cross-relaxation effect between Yb(III) and Er(III), by the multilayer nanostructure. The strong intensity of the Er(III) signal enabled *in vivo* studies and multiplexed detection after coating of the particles with phospholipids. The experimental results confirmed the advantage of these probes with comparable fluorescence intensity for high-fidelity multiplexed lifetime bioimaging.^568^ To further optimize the fluorescence intensity, nanoparticles were designed with different thicknesses of the Yb(III) layer and various amounts of Ce(III), controlling cross relaxation between Ce(III) and Er(III).^568^ The nanoparticles α-NaYF_4_@NaErF_4_:Ce@NaYbF_4_@NaErF_4_:Ce@NaYF_4_ emit ca. 2.6-fold stronger NIR-II and *in vivo* multiplexed lifetime imaging was achieved. Furthermore, a modification of the particles with an inert NaYF_4_ shell and a core–shell–shell structure allowed change of the luminescence quantum yield and lifetime to achieve fast fluorescence lifetime imaging. The nanoparticles β-NaErF_4_:2%Ce@NaYbF_4_@NaYF_4_ coated with polyethylene glycol entered blood circulation with half-lifetime of 0.3 h. Whole-body vascular fluorescence lifetime imaging in mice was successful to reveal a murine abdominal capillary network with a lower tissue background as compared with fluorescence imaging (Figure 29).^569^

A core–shell–shell structure was used with Ce(III) doping in the middle layer and liposome encapsulation for high contrast through-scalp and through-skull luminescence imaging of mice cerebral vasculature without craniotomy.^570^ The particles’ design NaYF_4_@NaYbF_4_:Er,Ce@NaYF_4_:Ca increased downconversion by 24-fold. Nanoparticles with sizes of 50 nm based on NaYbF_4_:Gd,Er,Ce@NaYF_4_ NaYbF_4_ were designed by C. Wang, Z. Liu et al. for brain tumor imaging and surgical navigation.^571^ Yb(III) ions were used as sensitizers to harvest 980 nm photons, and then transfer the energy to Er(III) ions efficiently which emit from the ^4^I_13/2_ level, leading to 1550 nm luminescence. Nanoparticles doped with 5% Er(III) displayed the strongest signal. The particles were coated with cell membrane vesicles obtained from human glioblastoma in order to overcome the blood brain barrier; with final size of 160 nm. Strong glioma-associated nanoparticle emission was observed in the tumor position in the brain of mice, while there was almost no signal in the nearby normal brain tissue. This indicated that the tumor in the brain can be clearly distinguished under the guidance of NIR-II imaging.^571^

**Figure 30:**
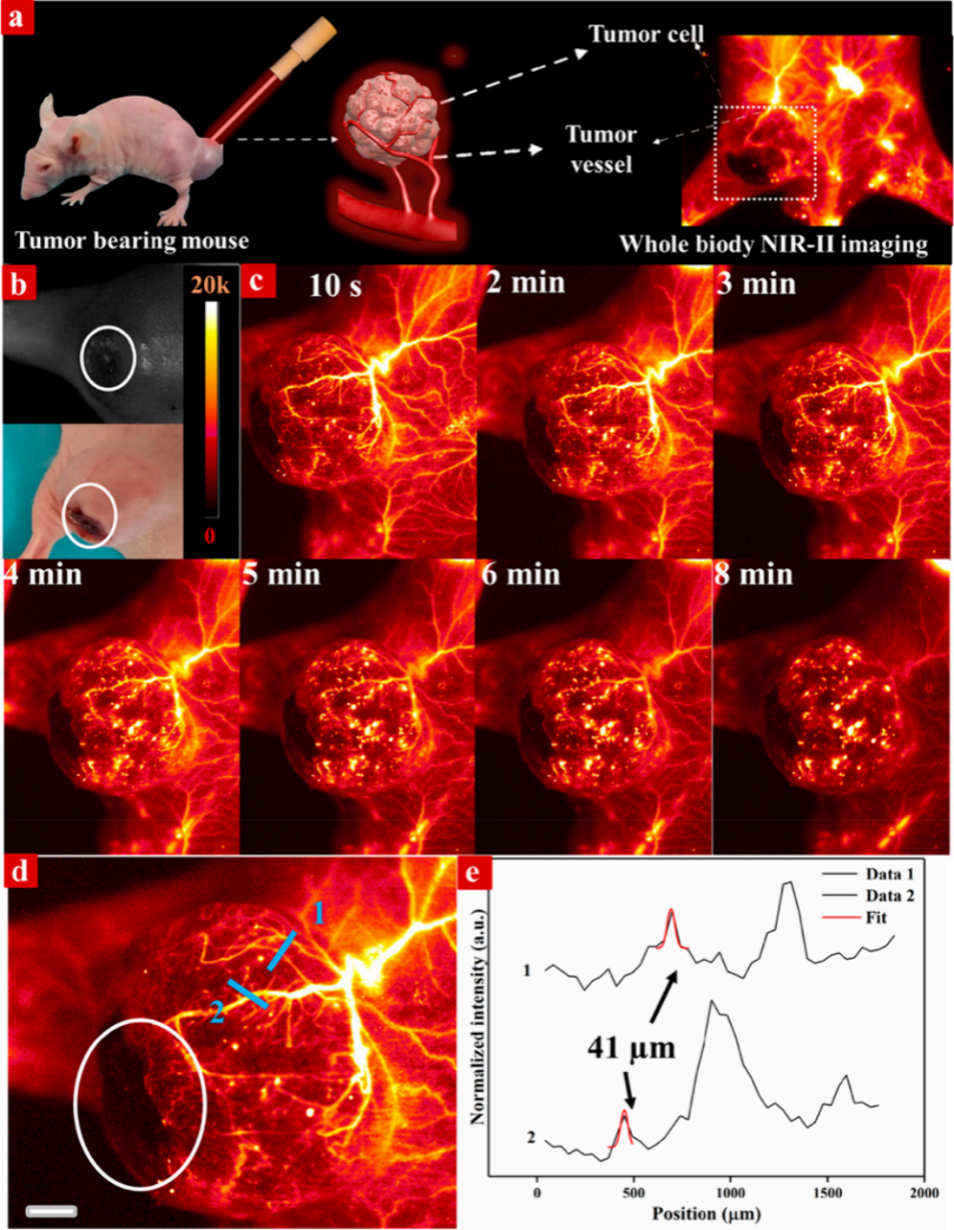
Illustration and images of Lewis Lung Carcinoma (LLC) bearing mice. (a) Schematic illustration of NIR-IIb optical imaging-guided LLC tumor vessel detection. (b) Bright field image (up) and digital photograph (down) of the LLC tumor. (c) The time coursed NIR-IIb imaging of a mouse tumor under the excitation of 980 nm lase with an excitation power density of 100 mW/cm^2^. (d) Magnified tumor vascular image. (e) Cross-sectional fluorescence intensity profiles along blue lines 1 and 2 of the tumor site. The scale bar is 2 mm in (d), and the white circles in (b) and (d) indicate the necrosis region in the epidermis of the primary tumor. Reproduced with permission from ref (572). Copyright 2019 American Chemical Society.

Poly(acrylic acid) (PAA)-modified NaLuF_4_:40Gd/20Yb/2Er nanorods with 5% Ce(III) doping presents enhanced downshifting NIR-IIb emission, high quantum yield of 3.6% (QY) and relatively narrow bandwidth (∼160 nm), and high biocompatibility via Ce(III) doping for high performance NIR-IIb bioimaging. The probes were used for high sensitivity small tumor (∼4 mm)/metastatic tiny tumor detection (∼3 mm), tumor vessel visualization with high spatial resolution (41 μm), and brain vessel imaging (Figure 30).^572^

A core–shell strategy for downshifting nanoparticles was developed for Tm(III), NaYF_4_:Yb_0.8_/Tm_0.08_@NaYbF_4_@NaYF_4_ (α-TmNPs) and then was expanded to Er(III)(α-ErNPs) and Ho(III)(α-HoNPs). The Tm(III) 1632 nm luminescence was amplified by Yb(III) while upconversion was decreased. The longer 1632 nm emission of α-TmNPs is located in the valley of the water absorption curve decreasing the filtering effect of water usually observed in the case of α-ErNPs. A simultaneous dual-channel imaging system with high spatiotemporal synchronization and accuracy was developed for Er and Tm. Real-time dynamic multiplexed imaging of hemodynamic was studied in mouse subcutaneous tissue and ischemic stroke model.^573^

The development of nanocrystals based on Tm(III) and Ho(III) allowed detection to an extended NIR-II window in the region of 1850–2840 nm optimizing photon scattering and penetration with minimum absorption of water for increased signal output in *in vivo* studies.^574^ A series of lanthanide nanocrystals based on Tm(III) and Ho(III) doping were developed for luminescence generation at 1852 nm (Tm(III), ^3^F_4_–^3^H_6_) and 2030 nm (Ho(III), ^5^I_7_–^5^I_8_) upon excitation at 980 nm from the Yb(III) sensitizer. The absorption spectra of water overlapped with emissions of Tm(III) at 1474 nm and Ho(III) at 2030 nm, resulting in strong luminescence quenching, where the peaks at 1852 nm (Tm(III)) and 1532 nm (Er) showed good signals. The luminescence properties of these particles were tailored with different compositions of Tm(III), Ho(III), and Er(III) by controlling the interface energy transfer based on the core–shell nanostructures. The percentage of Tm(III) as activator and Yb(III) as sensitizer was first investigated in β-NaYF_4_:Yb,Tm@NaYF_4_ nanocrystals and showed that β-NaYbF_4_:5%Tm(III)@NaYF_4_ and β-NaYbF_4_:1%Tm(III)@NaYF_4_ were optimal components for 1852 nm (^3^F_4_–^3^H_6_) and 2324 nm (^3^H_4_–^3^H_5_) luminescence, respectively. The quantum yields of β-NaYbF_4_:5%Tm@NaYbF_4_@NaYF_4_ and β-NaYbF_4_:1%Ho@NaYbF_4_@NaYF_4_ were found to be 3.92% and 2.86%, respectively. Multiplexed, *in vivo* imaging was achieved by different nanocrystals which were tail-vein injected in mice for the detection of vascular and intestine systems as well as tumors lymph tubes and nodes.^574^ A NIR-III emissive NaErF_4_:Yb@NaYF_4_:Yb exhibits improved thermal responsive sensitivity and enhanced 1550 nm emission intensity by modulating the interaction between H_2_O and lanthanide ions. Through optimization of a thin active shell layer doped with Yb(III), the probe showed temperature response of the nanocomposite in aqueous, which can be attributed to energy transfer between lanthanide ions (Er(III) and Yb(III)) and H_2_O molecules via environment quenching assisted downshifting process. By utilizing the temperature response behavior and the long-wavelength emission of the nanocomposite, high spatial resolution temperature monitoring of micron-scale cerebral vessels in a hypothermia mouse brain tissue was achieved via NIR-III imaging.^575^

#### 4.3.2 Upconversion Luminescent Systems

Upconverting nanoparticles have shown immense potential in biomedical imaging due to the efficient excitation by NIR light, with allows penetration in deep tissues but also eliminates autofluorescence due to the lack of excitation of other biological pigments.^13,49,582−584^ Yb(III) and Er(III) codoped NaYF_4_ nanoparticles coated by amphiphilic PEG–phospholipids were tracked in living HeLa cells, demonstrating their stability for real-time imaging and their potential in tracking active transport by motor proteins such as dyneins and kinesins.^585^ Prasad e*t al*. reported a fluoride host, NaYF_4_ for Tm(III) and Yb(III), visualizing the Tm(III) emission at 800 nm with 975 nm excitation in animal imaging studies.^582^ A core–shell design of UCNP, α-NaYbF_4_:Tm(III)/CaF_2_ showed highly efficient NIR–NIR upconversion, and although these UCNP showed high-contrast photoluminescence imaging of deep tissues, the limitations of deep tissue penetration and light scattering were identified due to the low quantum yield of 0.6 ± 0.1% under low power density excitation (∼0.3 W/cm^2^).^576^

UCNP based on NaYF_4_:20% Yb, 8% Tm have shown nanometer-scale optical resolution as single nanoparticles using super-resolution stimulated emission depletion (STED) microscopy under low-power.^586^ The resolution achieved was 28 nm, 1/36th of the excitation wavelength. They further performed a study with NaYF_4_:Yb, Tm nanoparticles (4% Tm(III) 40% Yb(III)) attached to a 93 μm thick slice of mouse liver tissue to demonstrate the potential of UCNP for deep tissue super resolution imaging.^577^ Using a doughnut beam excitation from a 980 nm diode laser and detecting at 800 nm, a resolution of sub 50 nm, 1/20th of the excitation wavelength was achieved.^577^ For deep tissue imaging this NIR-to-NIR configuration is attractive by detecting emission above 700 nm and enabling upconversion nanoscopy imaging in deep tissues. Tian et al. have designed UCNP without doping Ln(III) ions into an inert NaYF_4_ host matrix. The alloyed core/shell UCNP NaYb_0.8_Er_0.2_F_4_ are brighter than comparably sized doped UCNP at all laser intensities tested, over a range of 4 orders of magnitude. In live mice, aqueous 12 nm core/shell UCNP can be imaged with strong contrast through several millimeters of tissue with a laser intensity of just 0.1 W cm^–2^.^578^

**Figure 31:**
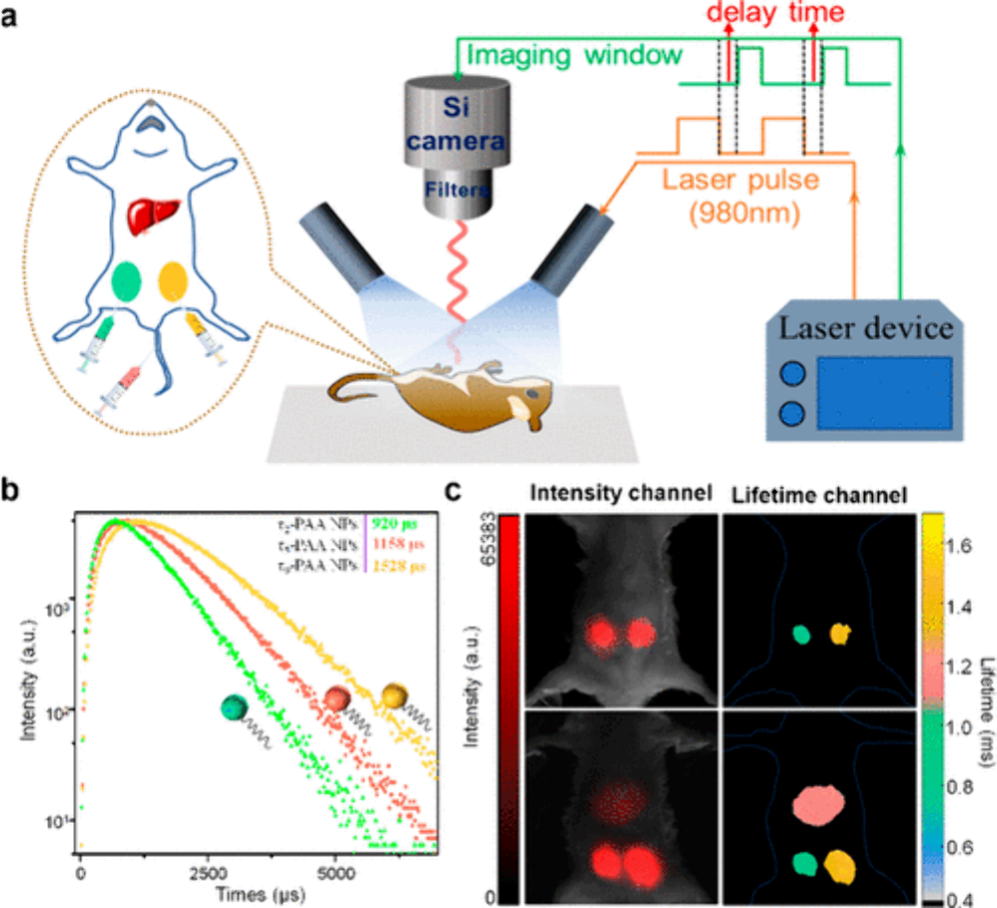
Temporal multiplexed *in vivo* upconversion imaging. (a) Schematic illustration of nanoparticles administration followed by imaging in a home-built time-resolved upconversion luminescence imaging system. (b) Measured upconversion luminescence decay profiles of PAA-coated core/multishell nanoparticles with lifetimes of τ_2_, τ_5_, and τ_9_ (aqueous dispersion). (c) Upconversion luminescence intensity (top left) and lifetime (top right) imaging with lifetimes of τ_2_ and τ_9_ in a Kunming mouse and in a second Kunming mouse (bottom) (subcutaneous injection of these nanoparticles into abdomen). Intravenous administration of nanoparticles with a lifetime of τ_5_ was implemented for the second mouse, enabling to light up the internal organs for both luminescence intensity (bottom left) and lifetime (bottom right) upconversion imaging. τ values correspond to those of nanoparticles. Reproduced with permission from ref (579). Copyright 2020 American Chemical Society.

A tetradomain nanostructure design of UCNP NaYF_4_@NaYbF_4_@NaYF_4_:Yb(III)/Tm(III)@NaYF_4_ was designed based on multilayers built on the hexagonal NaYF_4_ host lattice to allow energy migration processes from Yb(III) to Tm(III) for sensitization of the 808 nm upconverting luminescence (^3^H_4_ → ^3^H_6_ transition) upon excitation at 980 nm. The inert core and outer shell provided regions to minimize energy migration losses and UCL quantum yields of 3.5–6.1%, 0.11 W/cm^2^ were reported. The characteristic advantage was a multitude of distinct lifetimes that span 2 orders of magnitude (from 78 to 2157 μs) which enabled lifetime-colored imaging alongside luminescent intensity imaging of internal organs such as liver and two abdomen subcutis with high contrast in a Kunming mouse (Figure 31).^579^

High contrast mapping of the acidic tumor microenvironment *in vivo* was achieved with UCNP NaYF_4_:Yb,Er@NaYF_4_:Nd integrated with a pH-modulated reversible responsive dye asymmetric cyanine. The dye acts as a quencher for upconversion luminescence at 540 nm under normal conditions and as a sensitizer under acidic conditions.^580^

Wong, Bünzli, Zhang et al. reported a uniformly structured Cr(III)-sensitized lanthanide-doped nanoparticles with monoclinic-phase Na_3_CrF_6_ as host and sensitizer.^581^ The brightness of Na_3_CrF_6_:X (X = Er(III), Tm(III), Yb(III) or Nd(III)) was up to 280-times higher than that of the most intense conventional lanthanide-sensitized DSNP with similar sizes. By the epitaxial growth of Na_3_CrF_6_ shell onto the Na_3_CrF_6_:X core, the brightness of Na_3_CrF_6_:X@ Na_3_CrF_6_ was enhanced up to 370-times higher than that of DSNP with an inert shell coating. High-contrast bioimaging was explored by using persistent luminescence as *in vivo* internal excitation source for gastric tumor and stomach using cRGD-conjugated Na_3_CrF_6_:Er for *in situ* tumor labeling of the stomach and lipid-conjugated Na_3_CrF_6_:Nd. Based on the deliverable persistence luminescence internal excitation source, the stomach and tumor site can be highlighted by the NIR signals enabling high-contrast imaging with a low excitation threshold and high brightness.^581^

## 5 Therapy and Diagnosis in Cellular Environments

The detection of lanthanide luminescence signal is particularly attractive when it is combined with a therapeutic or a cellular monitoring event. The bioresponsive properties and therapeutic activities of lanthanides have been highlighted in designs of metal complexes,^28,159^ biorthogonal probes^587^ and nanoparticles.^49,588−591^ Exceptionally, the lanthanide-based NIR signal in noninvasive detection brings new dimensions in diagnostics.^592^ The second NIR window (1000 to 1700 nm) is popular for deep tissue, *in vivo* multiplex detection of biomarkers with high sensitivity and localized detection in nanoparticle designs.^593^ We describe an overview of bioactivity of the probes focusing on the lanthanide luminescence signal combined with a cellular activity for therapy or diagnosis.

### 5.1 Therapy

There has been considerable interest in anticancer activities of lanthanide complexes and oncotherapy using nanoparticles based on different pathways of activity.^159^ Predominantly, the activity is based on lanthanide-DNA interactions but there are many others leading to apoptosis influenced by the design: ligands generating singlet oxygen, nature of the lanthanide (NIR or visible emission), attached chemotherapy drug or drug included in a nanostructure. These approaches have been covered extensively in reviews on anticancer drug activity.^159,594^ The surface modification of nanoparticles for photothermal or photodynamic effect have also been reviewed elsewhere and are summarized herein with examples of designs and functions.^589,590^ A fascinating synergy is the optical detection accompanied by drug delivery or therapeutic activity which demonstrates the robustness of lanthanide signal detection in optical imaging and the area is covered in the section herein. We present a summary of the designs based on their function as photobased therapy (Table 11).

**Table 11:**
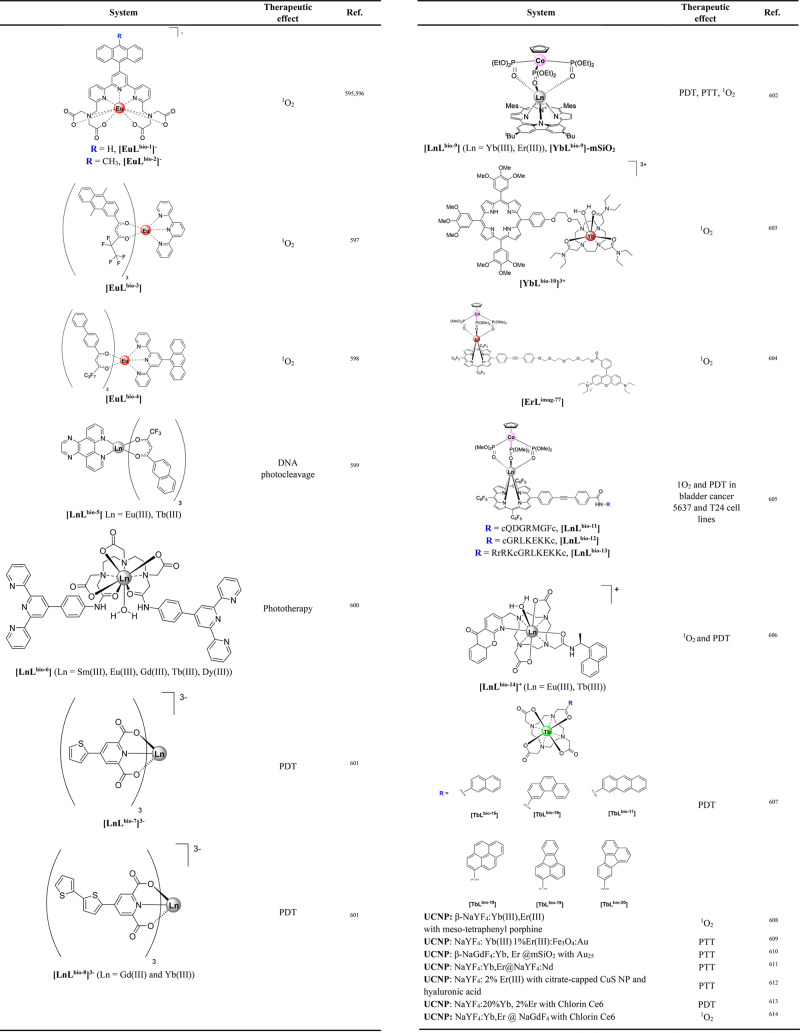

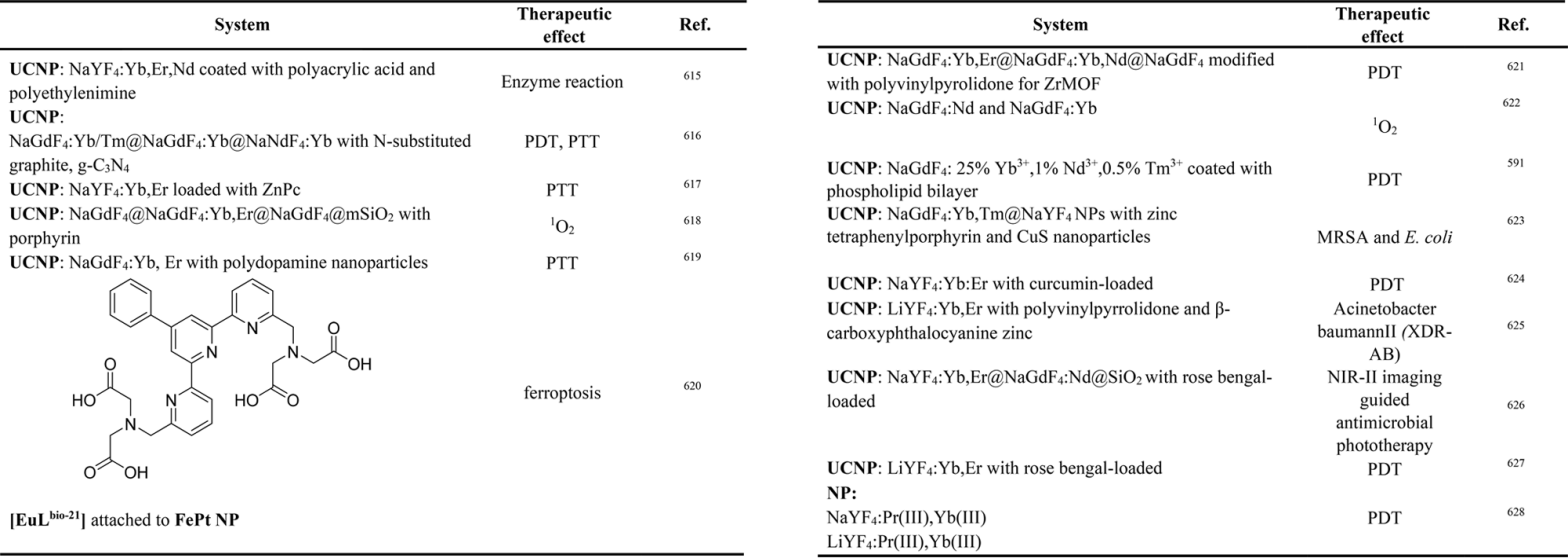
Chelated Lanthanides and Nanoparticles for Phototherapy Applications

#### 5.1.1 Photo-Based Therapy

Light-induced therapy is attractive for achieving high spatial-temporal control and noninvasiveness but limitations in clinical application arrive from weak efficacy of the available probes in the production of singlet oxygen (^1^O_2_), or shallow tissue penetration and phototoxicity.^629,630^ Formation of reactive oxygen species (ROS) by different sensitizers incorporated in lanthanide molecular complexes or nanoparticles is a widely spread approach supported by imaging and photothermal effects from the nanoparticle constructs. Photothermal therapy’s effect on cancer cells is led by the effective high local temperature to induce denaturation of proteins and cancer cell death. UCNP dominate recent approaches based on the in-depth tissue penetration of the NIR light.^590,631^ Our review expands from molecular to different nanoconstructs designs to provide an appreciation of strategies used incorporating lanthanide luminescence.

Lanthanide complexes have been studied for their DNA photocleavage properties for potential therapies. Yuan et al. introduced the 9-anthryl group onto Eu-terpyridine complexes to [**EuL**^**bio-1**^**]**^**–**^ sensitize singlet oxygen.^595^ In the absence of ^1^O_2_, there is no Eu(III) signal, presumably due to quenching by photoinduced electron transfer. In the presence of ^1^O_2_, the corresponding endoperoxide is formed, which leads to enhancement of the red Eu(III) emission. Modification of the 9-anthryl unit **[EuL**^**bio-2**^**]**^**–**^ allowed faster reaction of the probe with singlet oxygen, and the europium complex has good uptake in HeLa cells.^596^ The ^1^O_2_ was generated from a MoO_4_^2–^/H_2_O_2_ system, a photosensitization system of a porphyrin and a horseradish peroxidase catalyzed aerobic oxidation system of indole-3-acetic acid. When the anthracene derivative is attached onto the diketonate ligand, **[EuL**^**bio-3**^**]** was successfully used in time gated luminescence imaging of HepG2 cells.^597^ The complex acts as a luminescent probe for the monitoring of the time-dependent generation of ^1^O_2_ in the photodynamic activity of a drug, 5-aminolevulinic acid, loaded HepG2 cells. There is evidence by confocal luminescence imaging of the complex’s localization in mitochondria of HepG2 cells. An anthryl substituted terpyridine derivate and a β-diketonate coligand was used to develop **[EuL**^**bio-4**^**]** for the time-gated detection of singlet oxygen.^598^**[EuL**^**bio-4**^**]** was dissolved in DMSO, and detection of ^1^O_2_ in HepG2 cells was performed in the presence of 5-aminolevulinic acid (a PDT drug which acts as a photosensitizer) under photoirradiation. Increasing concentration of up to 40 μM of 5-aminolevulinic acid saw the increase in luminescence intensity of **[EuL**^**bio-4**^**]** in HepG2 cells.^598^ The quinoxaline ligand dpq (dipyrido[3,2-d:2′,3′-f]quinoxaline) is a known intercalator for nucleic acids and was successfully used as ancillary ligand in the formation of Eu(III) and Tb(III) complexes with naphthyl-substituted diketonates **[LnL**^**bio-5**^**]**.^599^ The luminescent complexes **[EuL**^**bio-6**^**]** show DNA phototoxicity cleaving supercoiled DNA at 365 nm excitation. The DNA photocleavage activity is attributed to the formation of reactive oxygen species by the dpq photoexcited triplet states evidenced by confocal luminescence imaging. A terpyridine functionalized lanthanide complex has also been introduced successfully in phototoxic studies and luminescence imaging in cancer cells.^600^

Bettencourt-Diaz et al. reported thiophene and bithiophene based dipicolinate complexes **[LnL**^**bio-7**^**]**^**3–**^ and **[LnL**^**bio-8**^**]**^**3–**^, respectively.^601^ These complexes generate ^1^O_2_, which was confirmed by a phosphorescence peak at 1270 nm in **[GdL**^**bio-7**^**]**^**3–**^ emission spectra. Cytotoxicity studies in the presence and absence of light in HeLa cells suggest high photocytotoxicity for **[LnL**^**bio-8**^**]**^**3–**^ (Ln = Gd(III), and Yb(III)). After irradiating with light, cells incubated with **[GdL**^**bio-8**^**]**^**3–**^ are 44% necrotic and 28% apoptotic, yet less than 25% of combined cells show as necrotic or apoptotic when in the dark. Likewise, cells incubated with **[YbL**^**bio-8**^**]**^**3–**^ show 32% of cells as necrotic and 19% as late apoptotic in the light. Control experiments in the dark and under light irradiation indicate that all cells remain viable in the absence of **[LnL**^**bio-8**^**]**^**3–**^. These results indicate that **[LnL**^**bio-8**^**]**^**3–**^-mediated photolysis induces cell death by necrosis.^601^

Porphyrinoid ligands have been employed as antenna ligands based on their large absorption coefficients, tunable triplet energy states, and versatile chemical modification.^558^ However, the low stability of the lanthanide complexes has led to the development of capping designs such as Co(II) complexes to cap the vacant coordination sites of the lanthanide. Sessler, Zhang et al. have fine-tuned excited-state energy dissipation and phototheranostic function as exemplified by a set of lanthanides (Ln = Gd(III), Yb(III), Er(III)) carbazole-containing porphyrinoid complexes which gives intense absorption in the within the phototherapeutic window **[LnL**^**bio-9**^**]**.^602^ The porphynoid ligand **L**^**bio-9**^ has a triplet state at 10,300 cm^–1^ which facilitates direct energy dissipation pathways for Ln(III) metals (Ln = Yb(III) and Er(III)). In addition, long-lived excitons are formed which are kinetically and thermodynamically competent to undergo fast nonradiative relaxation. This converts excited state energy to heat. In biology, this promotes local temperature elevation, permitting photothermal therapy and acoustic wave generation, enabling photoacoustic imaging (evaluated in a murine tumor model). When the energy gap between lowest triplet state and emissive Ln(III) excited state is larger than 2000–3000 cm^–1^, Ln(III) centered luminescence with a characteristic *f*–*f* transition dominates, while photodynamic therapy effect is minimal.^305^ Narrowing the energy gap to less than 2000 cm^–1^ leads to ^1^O_2_ sensitization and a turning on of photodynamic therapy effects, with a corresponding decrease in the Ln luminescence. In this regime, fluorescence imaging and photodynamic therapy effect can be achieved concurrently. When the ligand has a T_1_ state energy that is lower or comparable in energy to the Ln(III) cation excited state, a strong photodynamic therapy effect is typically observed.^602^

Mesoporous silica nanoparticles (mSiO_2_) have been used as hosts of photosensitizers based on their porous structure. A lead construct was prepared by encapsulating **[YbL**^**bio-9**^**]** into mesoporous silica nanoparticles. **[YbL**^**bio-9**^**]-mSiO**_**2**_ was tested as a theranostic agent and found effective for photoacoustic imaging-guided photothermal therapy and synergistic photodynamic therapy in murine cancer models. Photophysical studies reveal that **[YbL**^**bio-9**^**]** shows oxygen sensitive luminescence which is not observed in **[ErL**^**bio-9**^**]**. Both complexes show negligible absorption and emission differences upon continuous photoirradiation up to an hour, therefore are suitable for photosensitizing. **[YbL**^**bio-9**^**]-mSiO**_**2**_ promotes the formation of ^1^O_2_ and has good photothermal stability.^602^ Photothermal studies showed promising results with a decrease in tumor growth observed over days. These results show promise to develop as a phototherapeutic agent that is capable of inhibiting tumor growth following a single course of NIR laser photoirradiation.^602^

Zhang, Wong et al. reported a hydrophilic cationic porphyrin conjugated cyclen-based ytterbium complex **[YbL**^**bio-10**^**]**^**3+**^ which selectively targets the Golgi apparatus with emission within biological window (650 to 750 nm) in HeLa and A549 cells using linear to near-infrared excitation via two-photon laser confocal microscopy.^603^**[YbL**^**bio-10**^**]**^**3+**^ has ^1^O_2_ quantum yield of 0.45. It shows very high photocytotoxicity and less toxicity in dark in HeLa cells. To further substantiate its potentiality and therapeutic efficiency, photoinduced ^1^O_2_ generation had been sequentially performed *in vitro* for **[YbL**^**bio-10**^**]**^**3+**^. Upon excitation at 457 nm for a short period of time (to avoid ^1^O_2_ generation), **[YbL**^**bio-10**^**]**^**3+**^ was taken up by the cells intact, specifically localizing in the Golgi with no significant cell death. However, after 15 min of laser flash excitation at 430 nm, 90% HeLa cells loaded with **[YbL**^**bio-10**^**]**^**3+**^ deformed or had even lost their integrity.^603^

Wong et al. reported a hydrophilic Er(III) porphynoid complex, **[ErL**^**imag-77**^**]**, which was linked to rhodamine-B **[ErL**^**imag-77**^**]** which targets mitochondria.^604^ Numerous studies suggest that effective photodynamic therapy agents should be mitochondria-specific. The low amount of ^1^O_2_ generated by agents in mitochondria is sufficient to damage the mitochondria and initialize the death of cancer cells.^632^ In addition, a mild efficiency of ^1^O_2_ quantum yields selectively accumulated in the cancer cell is beneficial to the development of the bifunctional agents in monitoring the imaging process. **[ErL**^**imag-77**^**]** showed accumulation in HeLa cells. However, the cellular uptake of **[ErL**^**imag-77**^**]** was slower than rhodamine-B due to steric hindrance of the complex. **[ErL**^**imag-77**^**]** was noncytotoxic in HeLa cells in the dark; however, after 30 min of flash excitation with a 430 nm laser, the cells were either deformed or had lost their integrity.^604^

A modification of **L**^**imag-77**^ was performed, where Rhodamine-B was replaced with bladder specific or integrin α_v_β_3_ isoform-specific peptides, forming **[LnL**^**bio-11**^**]**, **[LnL**^**bio-12**^**], [LnL**^**bio-13**^**]** (Ln = Er(III), Yb(III)).^605^ A hydrophilic peptide chain RrRK was conjugated to the relatively more hydrophobic bladder cancer-specific peptide sequence (−cGRLKEKKc−), affording the amphiphilic peptide with an improved cell membrane permeability for **[LnL**^**bio-13**^**]**. Er(III) exhibits higher ^1^O_2_ quantum efficiency than Yb(III) due to efficient energy transfer from porphyrin to Yb(III), resulting in more excitation energy being channeled to singlet oxygen production for Er(III). Cellular uptake studies in bladder cancer 5637 and T24 cell lines locate **[LnL**^**bio-11**^**]** on the cell membrane and no localization was observed in HeLa, and MRC5 cell lines. This is consistent with the reported specificity of the conjugated R1 peptide in **[LnL**^**bio-11**^**]** toward integrin α_v_β_3_ overexpressed on the bladder cancer cell membrane. **[LnL**^**bio-12**^**]** and **[LnL**^**bio-13**^**]** localize on the lysosome in bladder cancer 5637 and T24 cell lines. From flow cytometric cell uptake experiments, uptake rates follow the trend **[LnL**^**bio-13**^**]** > **[LnL**^**bio-13**^**]** > **[LnL**^**bio-11**^**]**. Photodynamic therapeutic index (ratio of dark IC_50_ over light IC_50_) was the highest for **[ErL**^**bio-13**^**]** (ca. 34) in bladder cancer 5637 and T24 cell lines. The dark cytotoxicity of all these complexes were very low (dark IC_50_ of over 1000 μM).^605^

Lanthanide luminescence from complexes possessing a sensitizing moiety is unperturbed by variations in dissolved oxygen concentration in aqueous media. Eu(III) complexes (^5^D_0_ of Eu(III) at 17,200 cm^–1^) with sensitizers having a triplet energy of more than 22,000 cm^–1^ are generally insensitive to oxygen in solution. However, when thermally activated back energy transfer can occur from the long-lived metal excited state (e.g., Tb(III) ^5^D_4_ at 20,400 cm^–1^) to the triplet state of the proximate sensitizer, the lanthanide emission intensity and lifetime is modulated by variation of temperature and pO_2_.^633^ Parker et al. reported an azaxanthone-based sensitizer, placed close to a single naphthyl group on a cyclen core **[LnL**^**bio-14**^**]**^**+**^.^606^ The naphthyl triplet energy is around 20,850 cm^–1^, i.e., within 2 kT (298 K) of the Tb(III) ^5^D_4_ emissive level. Energy transfer can occur rapidly at ambient temperatures, leading to the establishment of a photoequilibrium that elongates the lifetime of the aromatic triplet state.^634^ In turn, this renders the triplet state susceptible to dynamic quenching by atmospheric oxygen, leading to formation of singlet oxygen. The insensitivity to oxygen of emission from **[EuL**^**bio-14**^**]**^**+**^ contrasts with the sensitivity of **[TbL**^**bio-14**^**]**^**+**^, which allows ratiometric analysis of pO_2_ in aqueous media, using mixtures of the two complexes. This was realized in determining pO_2_ in human serum. NIH-3T3 cells were incubated for 4 h in the presence of **[EuL**^**bio-14**^**]**^**+**^ or **[TbL**^**bio-14**^**]**^**+**^ and uptake visualized using a confocal microscope. Thirty minutes after irradiation of the cells in the field of view, over 70% of the observed cells were deformed or had lost integrity in **[TbL**^**bio-14**^**]**^**+**^. In contrast, for cells that were not irradiated, no observable change was evident in Eu(III) and Tb(III) analogues. These experiments were consistent with local damage associated with the formation of singlet oxygen, only for cells loaded with **[TbL**^**bio-14**^**]**^**+**^ and excited via the sensitizer at 355 nm. Such behavior affords the possibility of gaining spatial control using dual laser methods to damage selected compartments of cells, loaded with appropriate mixtures of complexes of Eu(III) (for observation) and Tb(III) (for damage) that possess a suitable subcellular localization profile.^606^

Porphyrins and their structurally related analogues (chlorins, porphycenes, and phthalocyanines) have made enormous contributions in photodynamic therapy. However, their tedious synthesis, poor bioavailability, and prolonged patient photosensitivity have limited their application. Therefore, alternate ligand families are in pursuit toward phototherapy.^607^ Ung, Gasser et al. reported a series of heptadentate **[TbDO3A]** complexes bearing different polycyclic aromatic hydrocarbons; naphthalene **[TbL**^**bio-15**^**]**, phenanthrene **[TbL**^**bio-16**^**]**, anthracene **[TbL**^**bio-17**^**]**, pyrene **[TbL**^**bio-18**^**]**, and fluoranthene **[TbL**^**bio-19**^**]** and **[TbL**^**bio-20**^**]** in order to identify the best photosensitizer for use in photodynamic therapy.^607^ The red-shifted absorption of **[TbL**^**bio-18**^**]** with its high its molar absorptivity (ε = 7600 M^–1^ cm^–1^, 406 nm) suggests **[TbL**^**bio-18**^**]** as the most promising candidate. EPR studies confirm that all these complexes can produce ^1^O_2_, but not OOH^•–^ and OH^•–^ radicals. No oxidative damage in HeLa cells was observed in the dark, but a significant amount of cell blebbing, cell debris, and shrinkage for samples treated with **[TbL**^**bio-18**^**]** upon photoactivation.^607^

UCNP have also been used for NIR excitation of ^1^O_2_ photosensitizers such as meso-tetraphenyl porphine encapsulated into a composite of β-NaYF_4_:Yb(III), Er(III) UCNP and biocompatible poly(ethylene glycol-*block*-(dl)-lactic acid) block copolymers (PEG-*b*-PLA). The particles show low dark toxicity and efficient cancer cell-killing activity (75% HeLa cancer cells) upon 980 nm excitation when exposed with 134 W cm^–1^ of 980 nm light for 45 min.^608^ NaYF_4_-based UCNP, with 30% Yb(III), 1% Er(III) were modified with adsorbed Fe_3_O_4_ nanoparticles followed by a seeded growth of an Au-shell for enhancing phototherapeutic activity while the supermagnetic iron oxide acts as layer to reduce quenching of the lanthanide luminescence by Au as well as to act as MRI probe for tracking. The particles were coated with polyethylene glycol polymer for stability and biocompatibility. The photothermal ablation of cancer cells was shown *in vitro* and the particles have been tracked *in vivo* with upconverting luminescence and MRI imaging in resultant particles.^609^

In a different approach, the photothermal effect of gold nanoclusters, Au_25_ was combined with Nd(III)-sensitized UCNP to produce probes for imaging guided cancer therapy.^610^ The Au_25_ nanoclusters were loaded into β-NaGdF_4_:Yb,Er UCNP modified with mesoporous silica shell, β-NaGdF_4_:Yb,Er@mSiO_2_. The mSiO_2_ shell was functionalized with poly(allylamine) to increase the absorption of the negatively charged Au_25_. *In vivo* experiments demonstrated the imaging function based on upconverting luminescence and the photothermal effect of the new constructs.^610^

Combined Au nanoclusters with UCNP were assembled as new materials with DNA hydrogels for photothermal therapy.^611^ The UCNP NaYF_4_:Yb,Er@NaYF_4_:Nd were coated with poly(ether imide) for hydrophilicity and the Au nanoclusters were formed in situ growth onto the modified NaYF_4_:Yb,Er@NaYF_4_:Nd. The DNA-UCNP-Au hydrogels were formed by electrostatic complexation of salmon sperm DNA with the cationic NaYF_4_:Yb,Er@NaYF_4_:Nd–Au to form low viscosity hydrogels NaYF_4_:Yb,Er@NaYF_4_:Nd–Au-DNA. The hydrogels showed high photothermal efficiency (42.7%) in *in vivo* experiments with mice bearing tumors in the leg region.^611^

Although photothermal therapy with nanoparticles seem to have high efficiency for localized killing of tumors, the size of nanoparticles affects blood circulation and clearance with the larger (100 nm) particles to be more effective in tumor penetration, but the smaller <10 nm ones to be better in tumor infiltration.^612^ To address this, size-switching NP were introduced so that they retain their size in blood circulation but release smaller NP when they reach the tumor. Capped UCNP were made, NaYF_4_:2% Er(III) (30 nm) and citrate-capped CuS NP were directly absorbed onto the positively charged UCNP and then coated with hyaluronic acid (HA) to yield the composite NaYF_4_:Er@CuS-HA. CuS was chosen as a potential photothermal agent. Upon reaching the tumor, high penetration of the particles was secured by the enzymatic degradation of the coating from the overexpressed HAase to release the small CuS particles which are expected to have high penetration infiltration. The particles showed high penetration as evidenced by electron microscopy studies of tumor slices and *in vivo* tumor ablation studies.^612^ These “nanofirework” particles have led to a new approach in tumor therapy.

**Figure 32:**
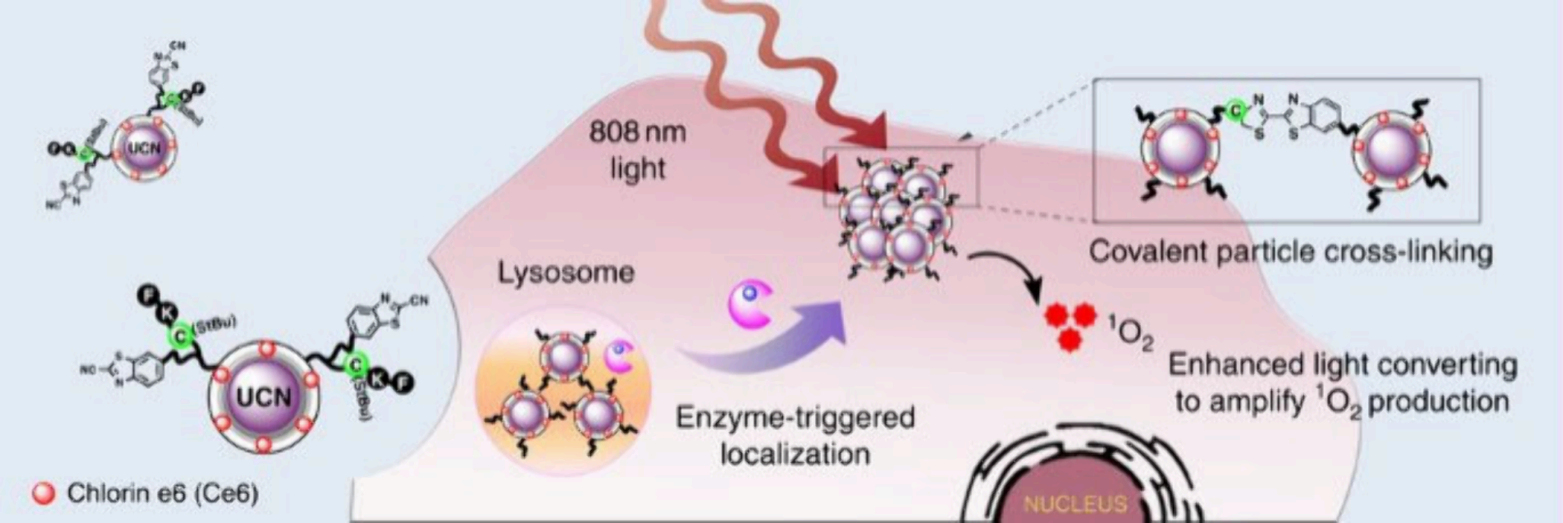
Schematic illustration of enzyme-triggered covalent cross-linking of peptide-premodified UCNP in tumor areas.^615^ The enhanced upconversion amplifies the singlet oxygen generation from the Ce6 onto the UCNP for enhanced photodynamic therapy treatment *in vitro* and *in vivo*. Reproduced with permission from ref (615). Copyright 2016 Springer Nature.

An FDA-approved photodynamic therapy drug (Chlorin Ce6) was incorporated into PEGylated amphiphilic polymer-coated NaYF_4_:Yb,Er nanoparticles. Photodynamic therapy efficacy is achieved in tumor-bearing mice upon direct injection of UCNP-Ce6 and after 980 nm laser excitation followed by NIR light exposure. UCNP after treatment are gradually cleared out from mouse organs, without rendering appreciable toxicity to the treated animals.^613^ Core–shell designs of upconverting nanoparticles allow deep tissue luminescence imaging and induce phototoxicity. Rod-shape NaYF_4_:Yb,Er nanoparticles coated with a shell of NaGdF_4_ were modified with chlorin Ce6 photosensitizer which can be covalently attached or absorbed on the coating layers of the rod. Upon excitation with 980 nm near-infrared light, the nanoparticles emit characteristic green and red light based on Er transitions. The red emission matches with the absorption peak of Ce6, leading to excitation of the photosensitizer which generates cytotoxic singlet oxygen. The nanoparticles were readily accumulated in tumor sites which could be clearly observed in the upconversion luminescence image.^614^ UCNP were developed with selective conjugation of peptides in order to be selectively cross-linked to tumor sites for effective delivery of photosensitizers (Figure 32).^615^ UCNP based on NaYF_4_:Yb,Er,Nd was coated with poly(acrylic acid) and polyethylenimine to improve biocompatibility and allow conjugation. The UCNP were modified with Ce6 as photosensitizer and an enzyme responsive peptide which contained 2-cyanobenzothiazole for reacting with cathepsin B, an important lysosomal cysteine protease that is overexpressed in various malignant tumors. The modified UCNP showed that the enzyme reaction was effective not only in cells but also in tumor bearing mice by localizing the light at the tumor microenvironment.^615^

A nanocomposite system based on a core–shell nanoparticle system NaGdF_4_:Yb/Tm@NaGdF_4_:Yb@NaNdF_4_:Yb with N-substituted graphite, g-C_3_N_4_, and polyethylene glycol was constructed using the distinctive properties of g-C_3_N_4_ in the production of ROS. Excitation by 808 nm NIR light, the emitted ultraviolet, and visible light can activate g-C_3_N_4_ to generate significant amount of ROS and the doped Nd(III) ions give rise to an obvious thermal effect, which leads to excellent antitumor efficiency due to the combined photodynamic and photothermal effect.^616^

NaYF_4_:Yb, Er UCNP were developed to achieve orthogonal emissions so that they have strong red or UV/blue emissions upon selective excitation at 980 or 808 nm that led to selective photoactivation.^617^ The UCNP were loaded with Zn-phthalocyanine and a SOD1 siRNA for gene knockdown for superoxide dismutase1 (SOD1), which is responsible for destroying free radicals. The enhanced phototherapy properties were shown *in vitro* in cervical cancer and oral cell carcinoma models and *in vivo* in human oral cell carcinoma models.^617^

A nanoarchitecture of mesoporous silica-coated UCNP with porphyrin photosensitizers covalently embedded inside the silica walls was designed with 808 nm-responsive diarylethene photochromic switches in the nanopores.^618^ Upon irradiation with 980 nm NIR light, ^1^O_2_ is generated based on the energy transfer to the porphyrins, while the 808 nm NIR light transforms the photochromic diarylethene to the open form, allowing recovery of the 980 nm light for further ^1^O_2_ generation. The photodynamic therapy potential of the system was demonstrated *in vivo* in tumor bearing mice.^618^ UCNP with a synergistic photodynamic and photothermal therapy effect were designed to control antitumor immunity.^619^ Polydopamine nanoparticles were coated with a NaGdF_4_:Yb, Er shell followed by modification with chlorin e6. In this design the NP has a core for photothermal effect and the shell for reactive oxygen species production. It was shown that the NPs are effective in ablating the tumor, while the released antigens can trigger the maturation of dendritic cells, to activate cytotoxic T lymphocytes and T memory cells, for inhibition of tumor metastasis.^619^

The anticancer effect of a novel ferroptosis agent was demonstrated by a novel design of FePt nanoparticles modified with folic acid for targeting; an Eu(III) terpyridine agent sensitizer, (N,N,N1,N1-[4′-phenyl-2,2′:6′,2′-terpyridine-6,6′diyl]bis(methylenenitrilo)tetrakis (acetate) **[EuL**^**bio-21**^**]** was attached to **FePt-NP**. Mechanistic studies showed that the FePt induced cancer cell death was affirmed as the ferroptosis mechanism. *In vitro* tests demonstrated that the nanoparticles exhibit a satisfactory anticancer effect toward folic acid positive tumor cells including 4T1, MCF-7, and HeLa cells and *in vivo* studies using tumor-bearing balb/c mice revealed that they significantly inhibit tumor progression.^620^

An upconversion MOF was designed for mitochondria-targeted photodynamic therapy.^621^ NaGdF_4_:Yb,Er@NaGdF_4_:Yb,Nd@NaGdF_4_ UCNP were modified with polyvinylpyrolidone for growth of Zr-based porphyrinic MOF on their surface. Following the growth of MOF, the surface was modified with triphenylphosphine molecules, which are known to target mitochondria. The UCNP transferred the light to the MOF for the photodynamic activity to take place and it was evidenced in tumor growths in mice.^621^ Sensitization of the triplet state of organic photosensitizers by NIR emitting nanocrystals was shown based on NaGdF_4_:Nd and NaGdF_4_:Yb systems.^622^ The nanocrystals were loaded with photosensitizers such as Ce6 and various porphyrins and phthalocyanines and studied in deep-tumor models. They demonstrated 100-fold improved performance for ^1^O_2_ generation compared with conventional upconversion-based NIR photosensitization.^622^

Two recently developed heavy-metal free photosensitizers dibenzothioxanthene imide and thiochromenocarbazole imide with high ^1^O_2_ production quantum yields (between 74% and 86%) were combined with UCNP, NaGdF_4_: 25% Yb^3+^,1% Nd^3+^,0.5% Tm^3+^ coated with phospholipid bilayer.^591^ The organic photosensitizer could be directly attached to the inorganic UCNP via the maleimide group or incorporated in the phospholipid bilayer via the aliphatic groups. UCNP to photosensitizer FRET efficiencies between 11% and 42% and ^1^O_2_ generation quantum yields between 74% and 86% were observed. HeLa cells incubated with the probe can be efficiently destroyed via 808 nm laser irradiance at 140 mW cm^–2^ for 3 min (<30% cell viability) or 3.2 W cm^–2^ for 6 min (<10% cell viability), demonstrating efficient NIR-sensitized PDT.^591^

A hybrid system including Tm-based UCNP, Zn-tetraphenylporphyrin (ZnTPP), and photothermal copper sulfide was introduced for phototherapy of brain glioma tumor using a dual step Fοster energy transfer.^623^ Tm(III) emission at 475 nm sensitizes the ZnTPP but also the 800 nm emission sensitizes CuS. The UCNP were engineered with a peptide (apolipoprotein E, ApoE) which is known for good penetration of the Blood Brain Barrier and good targeting of glioma cells. Upon excitation at 980 nm the nanosystem leads to formation of ROS and heat for efficient ablation of glioma cells in mice.^623^

Recently antimicrobial photodynamic therapy has been identified as an efficient method for bacteria eradication *in vitro* and *in vivo* and UCNP have been popular candidates.^624^ Curcumin-loaded UCNP have been proposed as dual activity agents based on the activity of curcumin itself and upon light for treating methicillin-resistant *Staphylococcus aureus* (MRSA) infection in joints.^624^ Another photosensitizer, β-carboxyphthalocyanine zinc, was also included in UCNP, LiYF_4_:Yb,Er coated with polyvinylpyrrolidone to treat MRSA and *E. coli*.^625^ Rose bengal-loaded UCNP^626^ and LiYF_4_:Yb,Er, were designed to treat an extensively drug resistant Gram-negative pathogen *Acinetobacter baumannII* (XDR-AB).^627^ Polyvinylpyrrolidone was used to coat LiYF_4_:Yb,Er and rose bengal was loaded based on electrostatic interactions. The UCNP achieved nearly 100% antibacterial efficacy upon 980 nm laser irradiation *in vivo* of XRD-AB infected tissues. This nanosystem achieved about 5 mm tissue penetration depth without side effects in the murine model.^627^

A design to achieve germicide action via induced damage of DNA and RNA of targeted microorganisms, was introduced with a dual activity NP exhibiting upconversion and downconversion luminescence. Their activity was demonstrated in bovine teeth and chicken flesh.^628^ Phototherapy and imaging was achieved with NP using Pr(III) and Yb(III)-doped fluoride nanocrystals into a nanosized fluoride matrix (NaYF_4_ and LiYF_4_) which exhibit upconversion and downconversion luminescence. These emissive modes combined in a single nanoparticle include emission upon NIR excitation (λ_ex_ at 980 nm and λ_em_ at 1320 nm), which was used for bioimaging in the NIR-II window and upconversion emission (λ_ex_ at 447 nm and λ_em_ at 275 nm) which was employed for germicide action. This was demonstrated by the denaturation of double-stranded into single-stranded DNA under 447 nm irradiation.^628^

#### 5.1.2 Drug Delivery with Luminescent Nanolabels

There is considerable interest to enhance the delivery of chemotherapeutic drugs using targeting vectors and monitor the efficacy of penetration by tracking the delivery with luminescence. Nanoparticles are an ideal platform for functionalization with targeting ligands and UCNP have shown increased interest in applications such as drug delivery systems.^593^ We have summarized the luminescence lanthanide systems for drug delivery (Table 12).

**Table 12:**
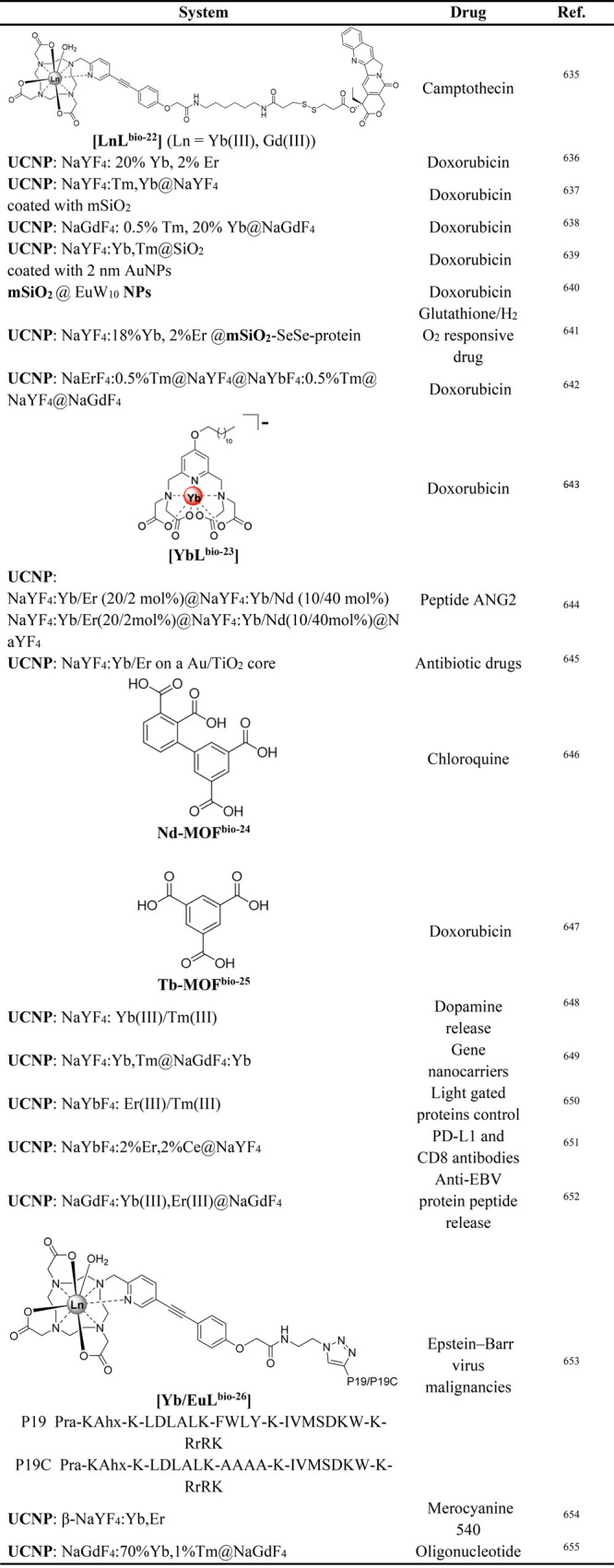
Chelated Lanthanide and Nanoparticles in Drug Delivery Applications

A nanoparticle system based on self-assembled Yb(III) and Gd(III) cyclen complexes, **[LnL**^**bio-22**^**]** was introduced to encapsulate camptothecin and monitor drug delivery in cancer cells by optical imaging *in vitro* and MRI imaging *in vivo*.^635^ The nanosized assembled structures protected camptothecin from degradation.

An anticancer drug, doxorubicin, was adsorbed onto PEGylated-UCNP NaYF_4_: 20% Yb, 2% Er held together by hydrophobic interactions. The upconverting lanthanide nanoparticle luminescence signal was used to track the drug delivery. When a targeting vector for folate receptor was conjugated to the nanoparticles, targeted drug delivery and tracking was achieved in cancer cells.^636^ Another approach for delivering doxorubicin was introduced with UCNP NaYF_4_:Tm,Yb@NaYF_4_ coated with mesoporous silica.^637^ The porous of silica were filled with an azobenzene dye which allowed release of the drug upon cis–trans photoisomerization of the azobenzene molecules. Upon irradiation with 980 nm light of NaYF_4_:Tm,Yb UCNP the doxorubicin release was controlled based on the azobenzene release and was successfully imaged in cell lines.

An amphiphilic azobenzene derivative was also included in phospholipids containing UCNP to give the hybrid system UCNP@Azo-Lipo for release of doxorubicin.^638^ Core–shell structured UCNP were synthesized NaGdF_4_: 0.5% Tm, 20% Yb@NaGdF_4._ The drug-loaded UCNP were studied in multidrug resistant cancer chemotherapy models *in vivo* and luminescence-guided imaging allowed control of the drug release triggered by NIR excitation.^638^ A nanoconjugate system with oligonucleotides was designed to stabilize UCNP and target the delivery of doxorubicin.^639^ UCNP which were modified with a silica shell bearing pendant amine group, NaYF_4_:Yb,Tm@SiO_2_, were coated with small gold nanoparticles (2 nm). Thiol-active oligonucleotides, hpDN, were conjugated onto the gold and loaded with doxorubicin. Drug release was observed by the photothermal effect upon irradiation. Deep tissue imaging *in vivo* confirmed the particle localization and the drug release was examined in tumor-bearing mice.^639^

Mesoporous silica particles were used to carry the therapeutic load, doxorubicin, in the porous network of silica, while a shell with a polymer poly(*N*-isopropylacrylamide-*co*-methacrylic acid), PNIMAM-MAA, carried lanthanide-polyoxometalates as luminescent labels. The loaded particles showed rapid release of doxorubicin in an acidic environment. HeLa cells incubated with mSiO_2_/PNIPAM-MAA/EuW_10_ for 1 h exhibited red luminescence and showed significant toxicity, demonstrating that lanthanide polyoxometalates composites can be used as bioimaging materials for cell monitoring and drug delivery.^640^ Stimuli-responsive mesoporous silica particles with embedded UCNP NaYF_4_:18%Yb, 2%Er bearing Se–Se linkers to proteins such as serum albumin and myoglobin were reported. NaYF_4_:18%Yb, 2%Er@mSiO_2_–SeSe-protein can undergo reductive/oxidative cleavages for glutathione/H_2_O_2_ responsive drug release.^641^ The Se–Se linker was used as it has a low bond energy of 172 kJ·mol^–1^. Upon release of the doxorubicin, UCNP luminescence is recovered due to elimination of the Förster energy transfer pathway between the UCNP core and the doxorubicin in the silica shell allowing the drug release to be tracked. These nanovehicles showed inhibitory effect of tumor growth, in tumor bearing mice.^641^

Photoswitchable multishell UCNP NaErF_4_:0.5%Tm@NaYF_4_@NaYbF_4_:0.5%Tm@ NaYF_4_@NaGdF_4_ were developed for two different light excitations: 980 nm to lead to UV-blue and 1525 nm emission and 800 nm only for 1525 nm emission. Th particles were modified with Ce6 as a photosensitizer and a calcium phosphate layer as a pH-sensitive layer for releasing the encapsulated doxorubicin. Irradiation at 800 nm did not produce tumor inhibition while 980 nm excitation showed effective reduction of tumor cells and inhibition of tumor growth in *in vivo* studies.^642^

Petoud et al. found that Doxil in liposomes (an anthraquinone derivative), an approved anticancer agent can sensitize Yb(III) emission.^643^ PEGylated DMPC/DSPE-PEG(2000) (1,2 dimyristoyl-*sn*-glycero-3-phosphocholine/1,2-distearoyl-sn glycero-3-phosphoethanolamine-N-[amino(polyethylene glycol) 2000]) liposomes incorporated a Yb(III) complex in their bilayer, followed by loading doxorubicin by a pH gradient method, **[YbL**^**bio-23**^**]**. Two distinct luminescent lifetimes were observed at 1.4 and 0.77 μs. This is due to the presence of the Yb(III) complex in and on the hydrophobic liposome where there is altering water exposure, leading to quenching effects on the Yb(III) center. When the liposome nanoparticle remains intact, lanthanide luminescence can be observed. Upon drug release, the lanthanide luminescence signal decreases. This was visualized *in vivo* by a 4T1 tumor bearing female mice on the fourth mammary gland. However, **[YbL**^**bio-23**^**]** had to be intratumorally injected for better visualization rather than intravenous injection, thereby questioning the biodistribution. Furthermore, this strategy lacked the ability to distinguish low liposome concentration from dissolved liposome/released drug.^643^

Doxorubicin and chlorin Ce6 were loaded to UCNP for synergistic chemo-photodynamic therapy for the treatment of glioblastoma.^644^ Mutli-shell UCNP NaYF_4_:Yb/Er (20/2 mol %)@NaYF_4_:Yb/Nd (10/40 mol %), noted as Er@Nd-NP and NaYF_4_:Yb/Er(20/2 mol %)@NaYF_4_:Yb/Nd(10/40 mol %)@NaYF_4_ noted as Er@Nd@Y-NP, were tested with different biocompatible coatings including poly(acrylic acid), human serum albumin, and a peptide ANG2, known to cross the blood–brain barrier. Luminescence bioimaging *in vivo* studies and high-resolution electron microscopy analysis confirmed that the peptide ANG2 functionalized NP traversed the blood–brain barrier into the interstitial tumor space. Enhanced efficiency in treatment of intracranial human glioblastoma model in mice was reported upon irradiation.^644^ It was shown that the UCNP provides a metronomic chemotherapy approach for optimal dosing, inducing an enhanced antitumor effect.

The delivery of ampicillin sodium, a widely used antibiotic drug to resistant bacterial strains, was achieved by an UCNP system, NaYF_4_:Yb/Er, built on a Au/TiO_2_ core.^645^ The TiO_2_ was designed for its photocatalytic properties demonstrating that the payload can be released by NIR-light triggered photocatalytic activity. It was shown that NIR excitation light triggers photocatalytic activity and release of the antibiotic but also leads to long-term release of reactive oxygen species.^645^

MOFs have also been reported to host drug molecules. Near infrared emitting lanthanides have been incorporated in metal–organic frameworks with the aim to increase luminescence and enhance imaging capabilities. A **Nd-MOF**^**bio-24**^ synthesized into block crystals and nanospheres with 2,3,3′,5′-biphenyl tetracarboxylic acid as ligand, with each Nd(III) ion adopting a spherical capped square antiprism configuration, coordinated to nine oxygen atoms.^646^ The **Nd-MOF**^**bio-24**^ displayed high quantum yield (8.9%) and high porosity (42.6%). An anticancer drug chloroquine was added during the MOF assembly and a CD44 receptor targeting agent, hyaluronic acid, was used for capping the MOF. After intraperitoneal injection into A2780 tumor-bearing mice, the NIR-II fluorescence intensity (at 1067 nm) reached maximum at 24 h, suggesting the excellent targeted NIR-II bioimaging property of the nanohybrid system. Both *in vitro* and *in vivo* experiments give evidence that nanohybrids present a great inhibitory effect on cancerous cells and tumors.^646^

Gold nanorods were employed with a luminescent Tb(III)-MOF, **Tb-MOF**^**bio-25**^**@AuNP** taking advantage of both the photothermal effect of nanorods and the delivery capacity of the MOF for doxorubicin. A two-photon absorption ligand 4-(2,4,6-trimethoxyphenyl)-pyridine-2,6-dicarboxylic acid was employed. Real-time fluorescence imaging-guided photostimulation combined with thermal-chemotherapy was achieved with highly effective photothermal conversion capacity and drug release efficiency under near-infrared light irradiation.^647^

#### 5.1.3 Other Therapeutic Activity

**Figure 33:**
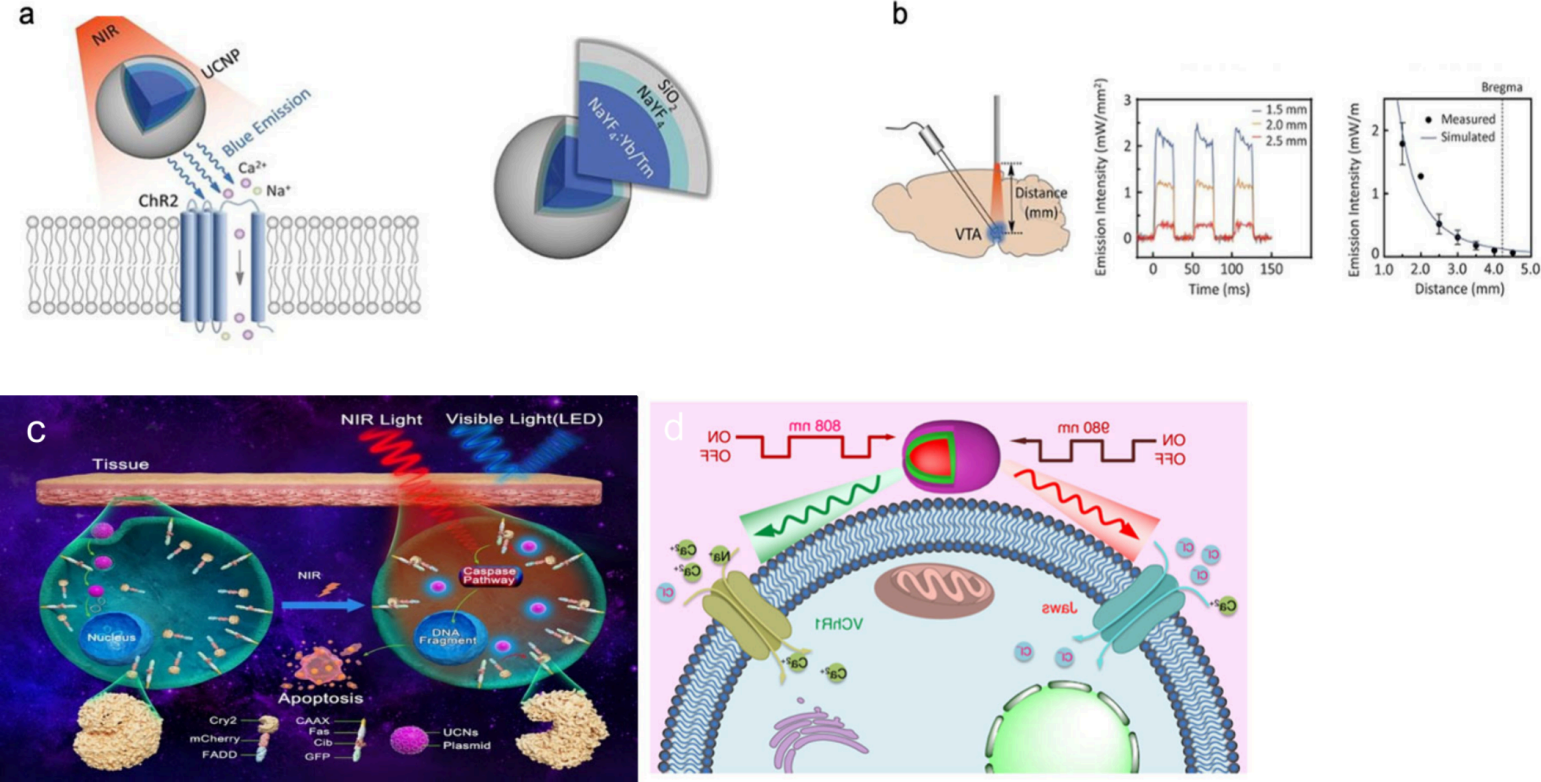
UCNP-mediated NIR upconversion optogenetics. (a, b) Deep brain stimulation. Reproduced with permission from ref (648). Copyright 2018 American Association for the Advancement of Science. (a) Schematic principle of UCNP-mediated NIR upconversion optogenetics and design of a blue-emitting NaYF_4_:Yb/Tm@SiO_2_ particle. (b) Scheme of *in vivo* fiber photometry for measuring UCNP-mediated NIR upconversion in deep brain tissue; upconversion emission at the VTA upon 980 nm NIR irradiation from varying distances; measured (*n* = 4 mice) and simulated intensity of upconversion emission at the VTA as a function of the distance from the NIR irradiation source. (c) Activation of photoreceptors. Reproduced with permission from ref (649). Copyright 2017 American Chemical Society. UCNP deliver plasmid DNA into the cell and then work as a nanotransducer to convert external deep-tissue-penetrating NIR light to local blue light to noninvasively activate photoreceptors leading to apoptotic signaling pathways of cancer cells *in vivo*. (d) Programmable photoactivation in cardiac pacing. Reproduced with permission from ref (650). Copyright 2022 Springer Nature. Schematic illustration of programmable activation of ion channel proteins Jaws and VChR1 through controlling the power and duration times of 980 and 808 nm lasers. The green emission produced upon 808 nm excitation of the UCNP activates VChR1 resulting in calcium cation influx, while the red emission produced by 980 nm excitation activates Jaws for chloride anion influx.

A revolutionary approach for therapy of neurological disorders was reported for mammalian system using UCNP in optogenetics based on their unique property to absorb near-infrared radiation penetrating efficiently brain tissue and leading to dopamine release.^648^ The adaptation of UCNP in optogenetics has been proposed since 2011^656^ and introduced for neural stimulation in culture and tissues.^631,657−661^ To stimulate activation of channelrhodopsin-2 (ChR2) NaYF_4_:Yb(III),Tm(III) blue emitting nanocrystals were developed. An optic fiber equipped with a 980 nm laser (2.0 W) over the mouse skull resulted in 0.34 mW/mm^2^ blue-light emission sufficient to activate the ChR2 cation influx. *In vivo* fiber photometry was performed to examine NIR upconversion by UCNP in the ventral tegmental area (VTA) of the mouse brain, a region located ∼4.2 mm below the skull (Figure 33). Additionally green emitting NaYF_4_:Yb(III),Er(III) were developed to activate halorhodopsin (NpHR) or archaerhodopsin (Arch) for neuronal inhibition.

UCNP NaYF_4_:Yb,Tm@NaGdF_4_:Yb were conjugated with poly(ethylene imine) in order to act as gene nanocarriers.^649^ The optogenetic activation of photoreceptors *Arabidopsis* flavoprotein cryptochrome 2 (Cry2), the photoreceptor of blue light which interacts with its partner Cib1 activating apoptotic signaling pathways in cancer cells. It was found that penetration of NIR light inhibited the growth of tumors. A design of UCNP to minimize overlapping emissions was proposed with orthogonal dumbshell shaped UCNP NaYbF_4_:Er(III)/Tm(III) and a Tm(III) core and a single activator Er(III).^650^ Under excitation of 808 or 980 nm green or red luminescence was selectively obtained. These UCNP were tested for bidirectional control of light gated proteins in order to treat cardiac pacing for arrhythmias. Tests in cells were followed by studies in cardiomyocyte clusters demonstrating the therapeutic action in cardiac pacing.^650^

A core–shell Er-based UCNP design NaYbF_4_:2%Er,2%Ce@NaYF_4_ was studied for downconversion of NIR luminescence. In the cubic α-phase core, an Er(III) ion activator was surrounded by eight F^–^ ions in the fluorite structure where Yb(III) served as the sensitizer to harvest 980 nm photons. The downconversion NIR-IIb luminescence of α-phase Er-nanoparticles was about 7.6 times brighter than that of β-phase ones upon 980 nm excitation. Their long luminescence lifetime (∼4.6 ms) enabled simultaneous imaging of nanoparticles conjugated to different antibodies. *In vivo* NIR-IIb molecular imaging of PD-L1 and CD8 antibodies revealed cytotoxic T lymphocytes in the tumor microenvironment in response to immunotherapy and altered CD8 signals in tumor and spleen due to immune activation. The cross-linked functionalization layer facilitated 90% nanoparticle excretion within 2 weeks without detectable toxicity in mice.^651^

**Figure 34:**
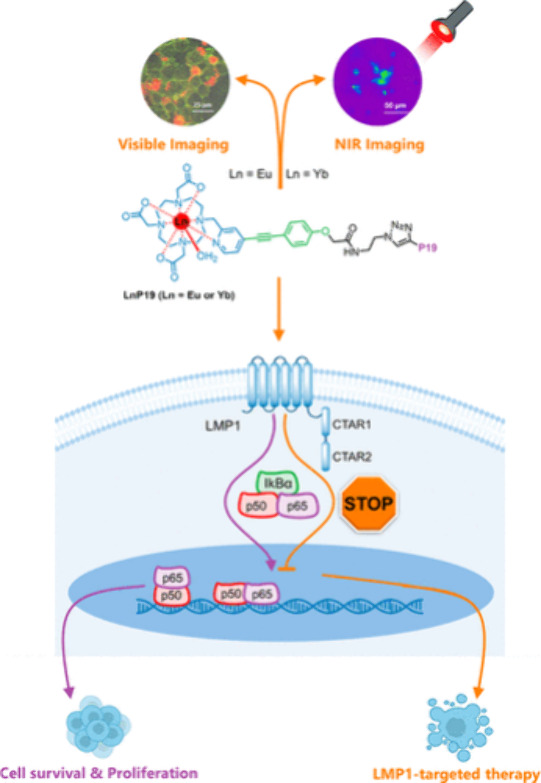
Schematic diagram of the visible and NIR imaging and the NF-κB inhibition capability of [**Yb/EuL**^**bio-26**^**]** on LMP1-positive cells (orange line, with the addition of probe; violet line, without **Ln-probe**). Reproduced with permission from ref (653). Copyright 2021 American Chemical Society.

A treatment strategy against Epstein–Barr virus (EBV) and inhibiting selective proteins has been proposed based on UCNP.^662^ Lung, Wong et al. developed modified UCNP NaGdF_4_:Yb(III),Er(III)@NaGdF_4_ with peptides which display emission signal enhancement upon binding to a viral latent protein, EBNA1.^652^ The motif of the EBNA1 peptide was then modified with a peptide engineered for a recognition of a cell membrane protein, latent membrane protein 1(LP1), and the final dual targeting peptide was attached to UCNP with pH sensitive linkers (Figure 34). The design allowed release of the anti-EBV protein peptide in the acidic tumor microenvironment in order to differentiate recognition minimize undesirable and unintended damage to normal cells and the nanoconstructs not only allowed *in vivo* inhibition of EBV-positive tumors but also enhanced specific uptake in comparison with non-EBV(+) cells.^662^ The peptide for targeting LP1was attached to Eu(III), Yb(III) complexes, [**Yb/EuL**^**bio-26**^**]**^653^ and the luminescent constructs allowed immunofluorescence imaging of inhibitors in cells.

Immunoadjuvant nanoparticles were prepared based on large-pore mesoporous-silica-coated β-NaYF_4_:Yb,Er UCNP.^654^ The particles were designed to combine photodynamic therapy and immunological synergistic therapy. The large mesoporous network was designed to include high loadings of merocyanine 540 for ^1^O_2_ production, mixing with MC540 and mice colon cancer tumor cell fragment the obtained nanovaccines provide dual-mediated immunopotentiation action.^654^ Core–shell UCNP NaGdF_4_:70%Yb,1%Tm@NaGdF light have been used as a local transducer to a photoactivatable immunodevice.^655^ UV light emission from UCNP triggers photocleavage of a hybridized oligonucleotide, liberating the ssDNA to achieve control of its immunoactivity. The surface of the UCNP was engineered with cationic polymers to load the oligonucleotides. The loading capacity of the oligonucleotide was calculated to be 42 molecules per nanoparticle.^655^ The UCNP was found to inhibit tumor growth in mice and selectively induce intratumoral immune activation.

### 5.2 Cellular Assays

In this section, we focus on assays performed in cellular environment to highlight the strong shift in detection and potential for further technological developments (Table 13), which complement the lanthanide systems reported in section 2.4*in vitro*. Most of the examples in vitro are dominated by anion binding in section 2.4 with only few of them performed in cellular assays, which were described in section 2.4 for relevant comparisons, namely **[EuL**^**diag-99**^**]**^**3+**^**[LnL**^**diag-100**^**]**^**3+**^, **[Eu**_**2**_**L**^**diag-108**^**]**.^292,295^ The detection of reactive species in cellular environments has been extensively reviewed elsewhere.^220,663,664^ Ln-nanoparticle systems have also been of wide interest for luminescent assays of biomarkers.^14^

**Table 13:**
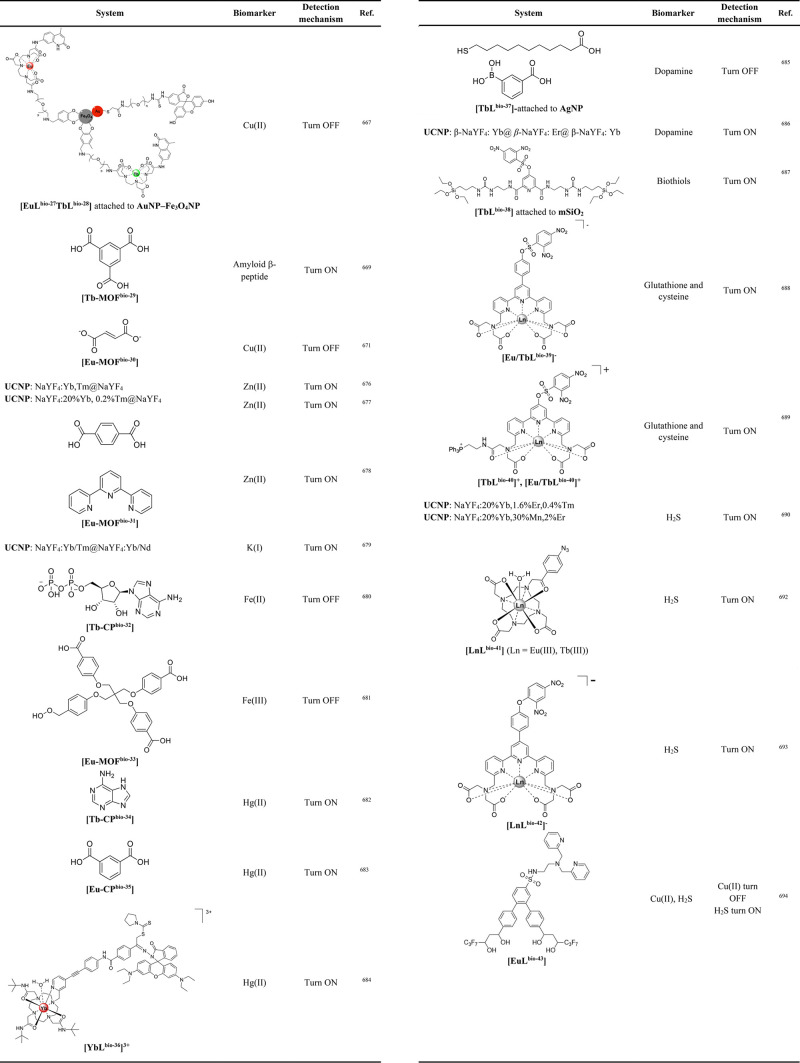

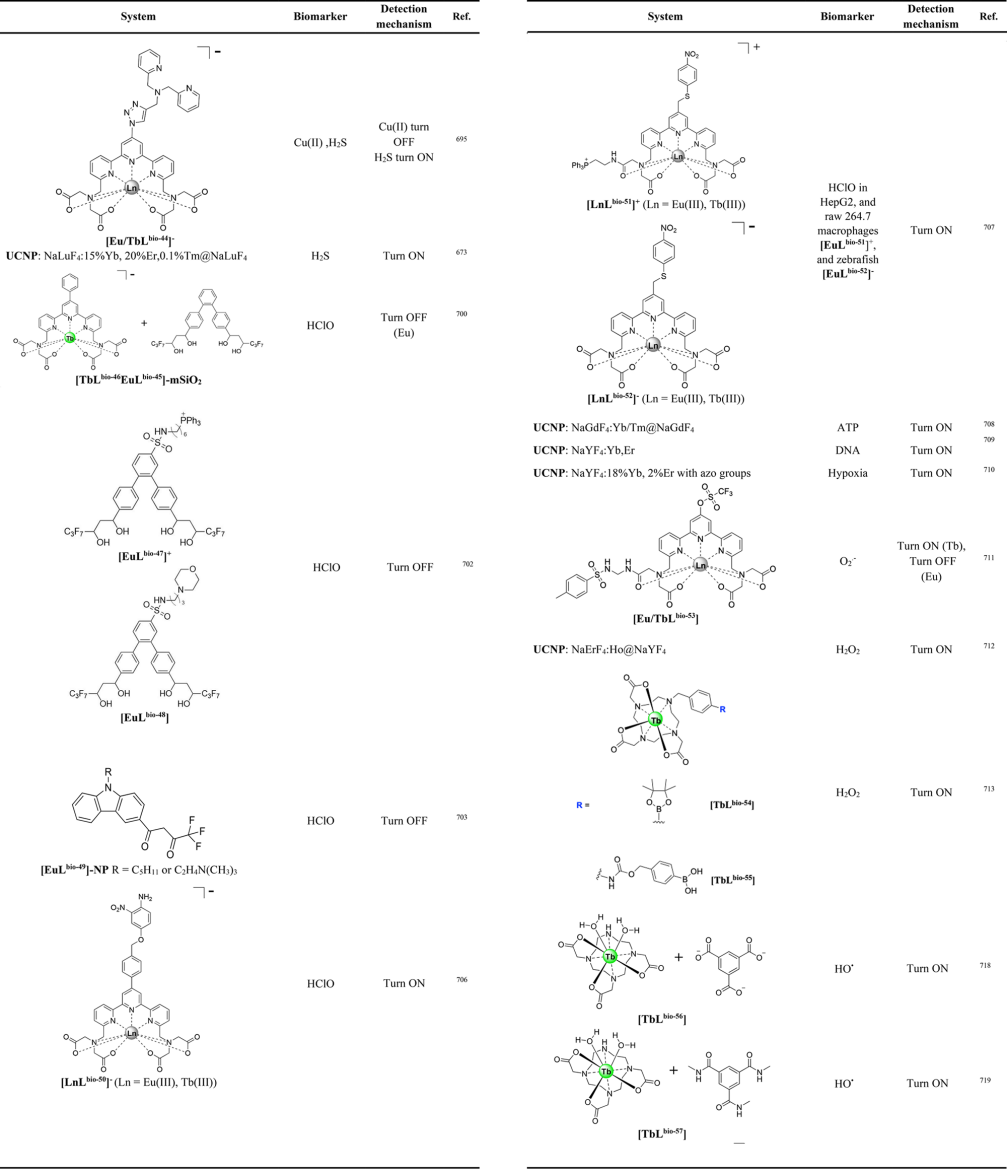

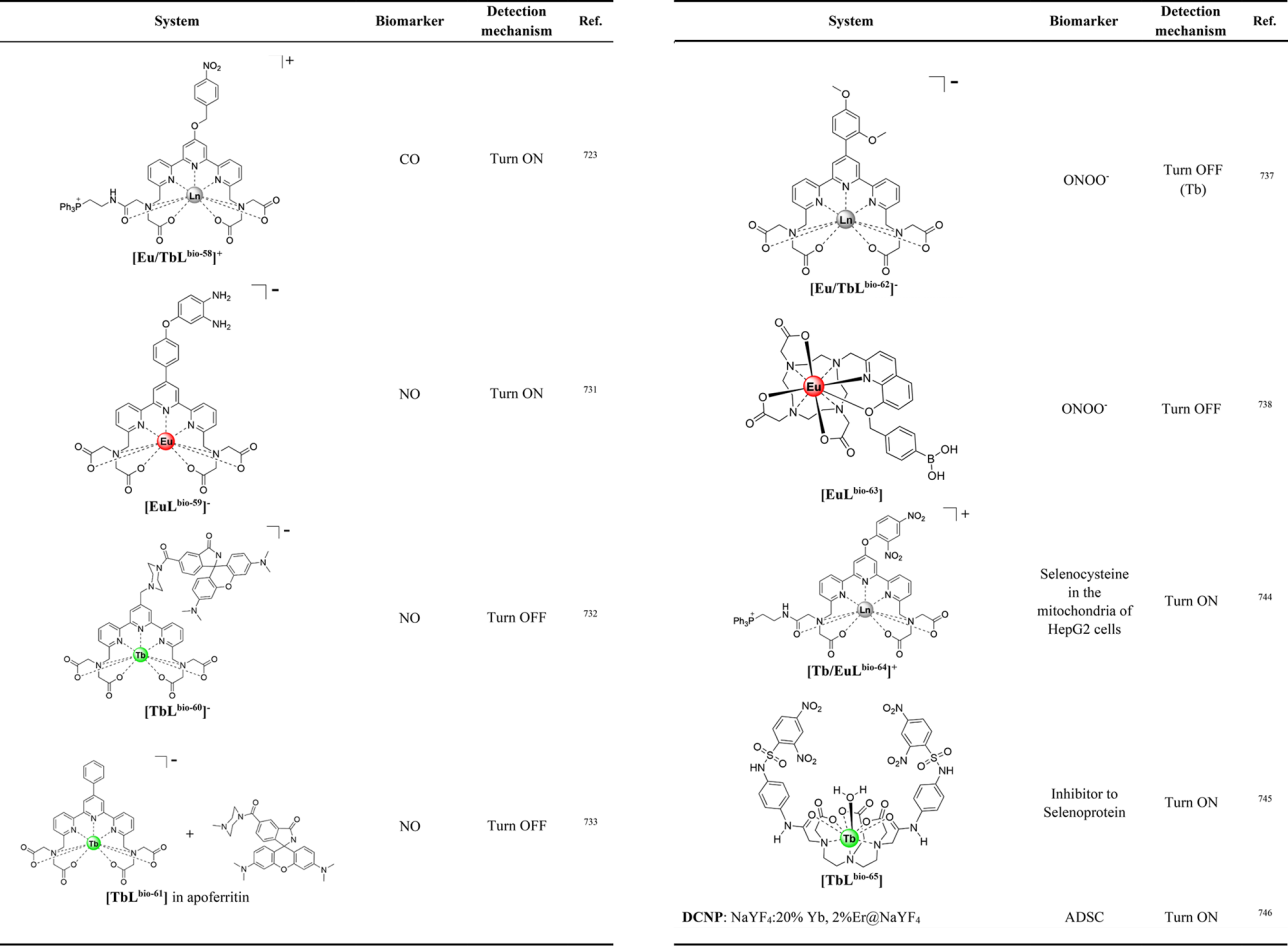
Chelated Lanthanides and Nanoparticles for Biomarker Detection in Cellular Assays

#### 5.2.1 Metal Ion Detection

Detection of metal cations has been of interest for disease diagnosis and lanthanide-based systems have been developed for biological fluid analysis based on time-resolved fluorescence detection.^665,666^

Excessive presence of Cu(II) ion in the body is associated with multiple disorders or chronic diseases. A triple-fluorescence dumbbell nanoprobe with large Stokes shift based on incorporating fluorescein isothiocyanate (FITC) and lanthanide complexes onto Au–Fe_3_O_4_ nanoparticles was developed.^667^**[EuL**^**bio-27**^**]** and **[TbL**^**bio-28**^**]** based on an asymmetric DTPA-bisamide derivatives were attached to Fe_3_O_4_ side and FITC as a reference agent was anchored on the Au side. The hybrid system displays well-resolved triple fluorescence emission, with FITC at 515 nm, Tb(III) complex at 545 nm, and Eu(III) complex at 616 nm under a single-excitation wavelength. In the presence of Cu(II), the Eu(III) and Tb(III) dissociate from the ligands and only FITC fluorescence is observed. The hybrid NP system has been successfully applied for three-channel ratiometric fluorescence and targeted multicolor imaging folate receptor-overexpressing HeLa cell lines *in vitro*.^667^ Tb(III) and Eu(III) diketonate complexes were also used to induce polysaccharide aggregates and detect Cu(II) in A-549 cells by addition of EDTA **[EuL**^**bio-27**^**TbL**^**bio-28**^**]**.^667^

Plasma amyloid-β peptide (Aβ) levels have been generally recognized as useful biomarkers for earlier diagnosis of Alzheimer’s disease.^668^ The chief component of Aβ plaques deposited in the brain are bound to Cu(II) ions.^668^ Using this, Qu, Zu et al. developed a Tb(III) metal–organic coordination polymer **[Tb-MOF**^**bio-29**^**]** built upon 1,3,5-benzenetricarboxylate ligands, bound to Cu(II). Upon the addition of Aβ, the Cu(II) is bound to the peptide which turns on Tb(III) luminescence due to the removal of quenching effect produced by Cu(II) in the MOF. Time-resolved fluorescence assays display high fluorescent intensity upon Cu(II) binding from MOF to Aβ_1–40_.^669^ A Tb(III) complex with DTPA functionalized with two *N*-benzylaniline as antenna can recognize soluble Aβ (biomarkers for early diagnosis of Alzheimer disease) in plasma through human serum albumin (HSA)-mediated coassembly by amplifying Tb luminescent signal in biological fluids.^670^

A microporous **[Eu-MOF**^**bio-30**^**]** was adopted for the detection of Cu(II) in aqueous solutions with two organic linkers butenedioic acid and ethanedioic acid with good selectivity and sensitivity for coordination with Cu(II) ions. The sensing function of MOF under simulated physiological conditions 20 mM HEPES aqueous solution demonstrated a significant quenching of Eu luminescence in the presence of Cu(II).^671^

The detection of Cu(II) ions in biological fluids can also be achieved by using a Tb(III) luminescent sensor based on the specific and high-affinity *Xxx-Zzz-His* (ATCUN) peptide motif, which can detect Cu(II) by significant luminescent quenching.^672^ A Tb complex attached to a bovine serum albumin protein-based sensor has been designed for high-throughput recognition and time-resolved fluorescence (TRF) detection of metal ions in biofluids.^673^ Cu(I) can be selectively detected through coordination with a Tb-bacterial metallo-chaperone complex, which shows enhanced luminescence via cation−π interaction established between the metal ion and the tryptophan indole.^674^ Additionally, an Eu(III) complex with a pendant dansyl antenna can be used for Cu(I) detection via the Huisgen 1,3-dipolar cycloaddition reaction using a stable biological Cu(I) source, showing an enhancement of Eu(III) luminescence.^675^

Detection of Zn(II) is important due to its physiological functions in gene transcription, protein structural stability, development, photosynthesis, immune response, and enzymatic activity. A novel approach for *in vivo* dynamic sensing of Zn(II) ions in cells and in zebra fish has been developed by combining UCNP with DNAzymes.^676^ The UCNP YF_4_:49%Yb,1%Tm@NaYF_4_ was modified by conjugating a Zn(II) specific-DNAzyme onto the silica shell of the UCNP. It was estimated that 31 DNAzyme sensor molecules were conjugated onto a single UCNP. The particle converts NIR light into 365 nm emission, which initiates the photodissociation of the caged 2′-nitrobenzyl group. This allows highly Zn(II)-specific cleavage of the single substrate strand into two shorter product strands. The luminescence signal was monitored in cell cultures before and after NIR irradiation. It was shown that after NIR irradiation, fluorescence enhancement was observed. The UCNP sensors have been tested in zebra fish and response to endogenous Zn(II) has been demonstrated, revealing patterns of distribution which are important in related diseases, especially cancer.^676^

Multilayered UCNP based on poly(acrylic acid)-coated NaYF_4_:20%Yb, 0.2%Tm@NaYF_4_ and assembled with acceptors were constructed for Zn(II) detection based on a FRET process.^677^ The acceptor has ligand sites to coordinate to Zn(II). Upon presence of Zn(II) the FRET between the UCNP and the acceptor is inhibited and the UCNP luminescence is restored. The detection of Zn(II) is rapid, within 5 s and detection is with high sensitivity down to 0.78 μM. The probe was applicable for *in vitro* and *in vivo* Zn(II) detection in the amyloid plaque in AD brain and zebrafish.^677^

An **[Eu-MOF**^**bio-31**^**]** based on terephthalic acid (H_2_BDC) and terpyridine can be used to detect Zn(II) using the ratio of Eu(III)-based emission via ratiometric fluorescence at concentrations ranging from 1 nM to 2 μM, with a low limit of detection (0.08 nM) and detected the presence of Zn(II) within 5 s. The enhanced analytical performance of the [**Eu-MOF**^**bio-31**^**]** probe allowed it to be successfully used for the sensitive monitoring of microdialysates in Alzheimer disease mouse brains and human urine.^678^

The potassium ion is the most abundant intracellular cation related to various biological processes, including neural transmission, heartbeat, muscle contraction and kidney function. Developing effective strategies, especially direct optical imaging methodologies, for monitoring the spatiotemporal dynamics of K(I) fluctuations is greatly valuable. A nanosensor constructed by encapsulating UCNP NaYF_4_:Yb/Tm@NaYF_4_:Yb/Nd and a commercial K(I) indicator potassium-binding benzofuran isophthalate in the hollow cavity of mesoporous silica nanoparticles, followed by coating a K(I)-selective filter membrane is designed. The membrane absorbs K(I) ions from the medium and filters out interfering cations. The UCNP UV light emission excites the K(I) indicator, thus allowing the detection of the fluctuations of K(I) concentration in cultured cells and intact mouse brains.^679^

Iron plays a vital role in many biological processes including oxygen transport, DNA synthesis, and cell proliferation. Deficient or excessive ingestion may cause the loss of some physiological functions and diseases including iron-deficiency anemia and ovulatory infertility and skin ailments, various anemia, and insomnia. Luminescent lanthanide coordination polymers (CP) **[Tb-CP**^**bio-32**^**]** based on self-assembly of nucleotide molecules, Tb(III) ions, and phenanthroline have been developed for detection of Fe(II) ions in human serum. The phenanthroline ligand, with sensitizing and recognition functions, not only enhances the luminescence but also provides high selectivity due to its specific binding ability to Fe(II) ions that can cause Tb luminescence quenching. The detection limit is as low as 30 nM.^680^

**[Eu-MOF**^**bio-33**^**]** with tetrakis[4-(carboxyphenyl)oxamethyl]methane acid as a ligand has shown excellent Fe(III) selectivity and detection. In the MOF, each lanthanide is coordinated by seven oxygens from six different ligands. The layers are further linked by strong hydrogen bonds to form three-dimensional networks with [H_2_NMe_2_]^+^ cations located in the interlayers and can be replaced by metal ions. Upon immersion of the **[Eu-MOF**^**bio-33**^**]** in the simulated physiological conditions, 20 mM HEPES aqueous buffer solution with different concentrations of Fe(III) ions, a remarkable quenching of Eu(III) luminescence is observed due to the cation-exchange reaction forming Fe(III)-Eu compound.^681^

Mercury(II) is one of the most toxic ions and can accumulate in the human body through the food chain, leading to brain damage and other chronic diseases. So, sensitive detection of mercury(II) ions is a very important task to ensure human health. Lanthanide coordination nanoparticles **[Tb-CP**^**bio-34**^**]** have been used to detect Hg(II). The coordination polymers are composed of adenine, Tb(III), and dipicolinic acid. Intramolecular energy transfer from adenine to dipicolinic acid suppresses the energy transfer of dipicolinic acid to Tb(III). In the presence of Hg(II), its coordination with adenine restores the energy transfer to Tb(III) and subsequently enhance its luminescence signal. The detection limit is 0.2 nM.^682^ A turn on fluorescence sensor for Hg(II) was based on a coordination polymer nanoparticle **[Eu-CP**^**bio-35**^**]** consisting of Eu(III) and isophthalic acid. The presence of imidazole carboxylic acid quenches the luminescence signal due to the overlap with the absorption with the polymer. Upon addition of Hg(II), the formation of the Hg(II) complex with imidazole carboxylic acid leads to turn on luminescent signal in biological fluid samples, up to a detection limit of 2 nM for Hg(II).^683^

Tanner, Wong et al. reported a Yb(III) cyclen-type complex bearing a Rhodamine-B sensing unit as a cell permeable sensor for Hg(II) **[YbL**^**bio-36**^**]**^**3+**^.^684^ A carbodithiolate unit in the complex was used to chelate Hg(II). Enhancement in luminescence intensity was observed upon the addition of Hg(II) in both the visible (∼596 nm) and NIR regions (∼980 nm) upon excitation of the pyridine unit. The visible emission enhancement at 596 nm occurs due to the increase in absorption of the Rhodamine-B moiety induced by its ring-opening mechanism (upon the addition of Hg(II)), thereby enabling more efficient FRET from the pyridine antenna to the Rhodamine-B fragment. The reversibility of Hg(II) binding to **[YbL**^**bio-36**^**]**^**3+**^ was observed upon the addition of S^2–^ to the **[YbL**^**bio-36**^**Hg]**^**5+**^ adduct, followed by the decrease in luminescence intensity, which was then restored upon the addition of Hg(II). MRC-5 cells incubated with **[YbL**^**bio-36**^**]**^**3+**^ exhibited very weak but detectable fluorescence in the living cells. However, a gradual luminescence increase was observed after the cells were incubated with varying Hg(II) concentrations at 37 °C. Reversible luminescence response of **[YbL**^**bio-36**^**]**^**3+**^ in living cells was observed when the Hg(II) treated cells were then exposed to S^2–^ at 37 °C.^684^

#### 5.2.2 Detection of Other Biomarkers

In section 2.4, detection of neurotransmitters by a quantitative method is a key role in diagnosis of diseases and disorders, including Alzheimer’s, Parkinson’s, and Huntington’s diseases. A novel terbium luminescent nanoprobe was designed for detection of dopamine based on AgNP modified with a 3-carboxyphenylboronic acid assembled with Tb(III) **[TbL**^**bio-37**^**]**. Tb(III) green emission is turned-off in the presence of dopamine due to the inefficient intramolecular energy transfer since the absorbed ultraviolet energy donated by the organic fluorophore was dissipated by dopamine. The detection process is within 2–3 s. The developed nanosensor gave rise to a linear correlation depending on dopamine concentration, and the detection limit was determined to be 0.41 μM. Two adherent cell lines (HeLa cells and THP-1 cells) exposed to the nanoprobe demonstrated striking green luminescence and showed on–off changes in the regulation of dopamine.^685^ A core–shell–shell “sandwich” structured UCNP [β-NaYF_4_:Yb(III)@*β*-NaYF_4_:Er(III)@β-NaYF_4_:Yb(III)] was made for efficient NIR to visible conversion and coated with silica shell. It was modified with an aptamer for detection of dopamine in neural stem cell derived dopaminergic-neurons, The particles were then coated with graphene oxide. The UCNP fluorescence is quenched due to the concentration-dependent interaction of the aptamer with graphene oxide. However, the presence dopamine induces changes in aptamers’ conformation causing the release of graphene oxide and recovery of luminescence. The system is sensitive to picomolar concentration and at the single-cell level.^686^

Cellular thiol-containing small molecules such as cysteine, glutathione, and homocysteine, play crucial roles in various biological processes. These biothiols regulate the redox states of the cells, and act as the ultimate source of reducing equivalents of enzymes to remove reactive oxygen species such as catalase, superoxide, and glutathione peroxidase. Altering levels of biothiols in biological fluids are associated with cancer, clinical stroke, lung damage, Parkinson’s, and dementia which necessitates the need to develop chemical probes for the detection of biothiols. A Tb(III)-based luminescent probe **[TbL**^**bio-38**^**]** attached on mesoporous silica nanospheres m-SiO_2_ was used for detection of biothiols.^687^ The hybrid probe exhibited the capabilities of quantitative determination and detection limits for biothiols were presented, namely 36.8 nM for cysteine 32.5 nM for glutathione. Evaluation of luminescence changes in cell culture demonstrated that this smart probe is in human embryonic kidney cells and human lung adenocarcinoma cells. The luminescence enhancement is attributed to the efficient energy transfer in the presence of biothiols where the sulfonate ester bonds are cleaved, and the electronic structure of the pyridine ring has been rearranged. Cell imaging under a fluorescence microscope revealed that both HeLa and A549 cells were stained intracellularly resulting in an intense green emission, indicating that the hybrid material penetrated cell membranes and was activated by thio-reactive components within cells.^687^ Terpyridine was functionalized with thiol sensing moiety, 2,4-dinitrobenzenesulfonyl, **[LnL**^**bio-39**^**]**^**–**^, which is a photoinduced-electron-transfer (PET) quencher.^688^ The addition of biothiol at physiological pH cleaves 2,4-dinitrobenzenesulfonyl which enhances Tb(III) luminescence, but not Eu(III). Therefore, a ratiometric approach of combining Eu(III) and Tb(III) analogs **[Eu/TbL**^**bio-39**^**]**^**–**^ (*I*_540_Tb(III)/*I*_610_Eu(III)) was used to quantify glutathione and cysteine in HeLa cell extraction solution.^688^ In a separate work, **[LnL**^**bio-39**^**]**^**–**^ was functionalized with a cationic thiphenylphosphonium **[TbL**^**bio-40**^**]**^**+**^**, [Eu/TbL**^**bio-40**^**]**^**+**^ to facilitate endogenous glutathione and cysteine detection in the mitochondria of Raw 264.7 macrophages. Furthermore, **[Eu/TbL**^**bio-40**^**]**^**+**^ was used to visualize high concentration of biothiol in the gut region of *Daphnia magna*.^689^

Detection of hydrogen sulfide, H_2_S, in cells has attracted interest based on its participation in cell signaling pathways.^690^ Two types of UCNP NaYF_4_:20%Yb,1.6%Er,0.4%Tm and NaYF_4_:20%Yb,30%Mn,2%Er, with green and red luminescence, respectively, assembled with chromophore dyes on their surface. The chromophore dyes act as energy acceptor for the upconverting luminescence. The presence of H_2_S leads to bleaching of the chromophores and restoring luminescence, evident by studies in HeLa cells and detection in blood samples.

Tropiano and Faulkner reported a DO3A-based Eu(III) complex with an azidophenacyl group **[EuL**^**bio-41**^**]** to detect H_2_S.^691^ In 10 mM PBS buffer at pH 7.4, a 6-fold luminescence enhancement was observed upon the addition of NaHS. This is due to the reduction of azide to amine which influences the energy transfer efficiency into Eu(III) center. It is noteworthy that 2-fold enhancement of Eu(III) luminescence enhancement was observed upon the addition of glutathione, but almost negligible enhancement for chloride, bicarbonate and other reducing agents, which shows its potential for cellular studies.^691,692^

**Figure 35:**
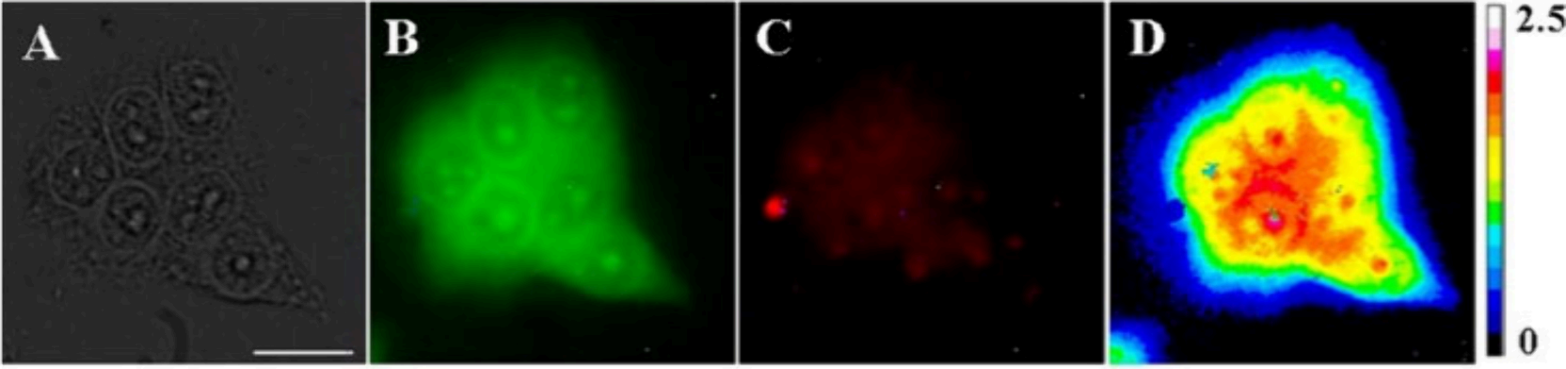
Time-gated luminescence images of **[Eu/TbL**^**bio-42**^**]**^**–**^-loaded HepG2 cells. (A) Bright-field image; (B) Tb(III) luminescence image; (C) Eu(III) luminescence image; and (D) ratiometric image (ratio = *I*_green_/*I*_red_). Scale bar: 10 μm Reproduced with permission from ref (693). Copyright 2014 American Chemical Society.

Yuan et al. prepared a terpyridine ligand conjugated to 2,4-dinitrophenyl **[LnL**^**bio-42**^**]**^**–**^ (Ln = Eu(III) and Tb(III)).^693^ The addition of H_2_S to **[LnL**^**bio-42**^**]**^**–**^ cleaves the PET quenching dinitrophenyl, resulting in luminescence enhancement for Tb(III), but a slight decrease for Eu(III). Therefore, a ratiometric time-gated luminescence approach of mixing **[EuL**^**bio-42**^**]**^**–**^ and **[TbL**^**bio-42**^**]**^**–**^ was established **[Eu/TbL**^**bio-42**^**]**^**–**^ to observe a linear trend of emission intensity (*I*_540_Tb(III)/*I*_610_Eu(III)) against H_2_S. 3:1 Eu(III) to Tb(III) **[Eu/TbL**^**bio-42**^**]**^**–**^ and **[TbL**^**bio-42**^**]**^**–**^ was used to image exogenous H_2_S in HepG2 cell line. Qualitative imaging of H_2_S by **[TbL**^**bio-42**^**]**^**–**^ was observed, and quantitative imaging using **[Eu/TbL**^**bio-42**^**]**^**–**^ was performed after an incubation period of 30 min with NaHS. The ratio image reveals the reacted product of **[Eu/TbL**^**bio-42**^**]**^**–**^ with NaHS localized significantly in nucleoli and to a lesser extent in cytoplasm (Figure 35). According to the calibration curve of ratiometric luminescence detection of NaHS, the concentration of NaHS in a single HepG2 cell was calculated to be in 10 to 20 μM, depending on the intracellular locations (up to 20.1 ± 2.3 μM in the nucleolus region).^693^

A β-diketonate Eu(III) complex possessing dipicolylamine **[EuL**^**bio-43**^**]**^**–**^ was found to be a good chelator for Cu(II) ions. Luminescence was quenched upon the addition of Cu(II) to **[EuL**^**bio-43**^**]**.^694^ However, upon the addition of S^2–^, luminescence was restored as the bound Cu(II) precipitated as CuS. This ‘“on–off–on”’ type signaling behavior of **[EuL**^**bio-43**^**]** toward Cu(II) and sulfide ions enabled the sensing of the two species with the detection limits of 3.7 nM and 0.19 mM, respectively. The presence of Cu(II) and sulfide ions in HepG2 cells were visualized better in time-gated luminescence microscopy.^694^ A similar strategy was reported where dipicolylamine was attached to terpyridine ligand, forming **[LnL**^**bio-44**^**]**.^695^ When Cu(II) was added, luminescence was quenched for both Eu(III) and Tb(III) analogues, but upon the addition of H_2_S, Eu(III) luminescence was restored, while Tb(III) had a slight increase. Therefore, a ratiometric approach **[Eu/TbL**^**bio-44**^**]** (*I*_610_Eu(III)/*I*_540_Tb(III)) was used to visualize H_2_S. Using **[Eu/TbL**^**bio-44**^**]** bound to Cu(II), H_2_S was quantified in human sera to be 42.1 ± 1.8 μM. *In vitro* detection of H_2_S was performed in HeLa cell line and *in vivo* studies using zebrafish with **[Eu/TbL**^**bio-44**^**]** was visualized. Other hydrogen sulfide reactive probes have been extensively reviewed elsewhere.^696^

UCNP NaLuF_4_:15%Yb, 20%Er,0.1%Tm@NaLuF_4_ coated with Prussian blue shells led to detection of H_2_S *in vivo* in a mouse model.^673^ The nanoprobes not only possessed outstanding H_2_S detection capacity *in vitro* (linear range: 0–150 μM, LOD: 50 nM) but were also feasible for H_2_S imaging. Moreover, the UC-PB_5_ can rapidly react with H_2_S to eliminate serum H_2_S with high efficiency. In cooperation with the long-term inhibition of H_2_S production by DL-PAG, effectively inhibited H_2_S-associated myeloperoxidase activation, this approach could further reduce the oxidative stress in the lungs, alleviating AP-associated lung injury.^673^

Hypochlorous acid (HClO) has received growing attention in identification and detection due to its vital role in bioassay of physiological processes, such as cell differentiation, migration, conduction, and immunity.^697,698^ Detection of neutrophil-derived HClO in living systems is related to hepatic ischemia-reperfusion injury, rheumatoid arthritis, lung injury, atherosclerosis, and renal disease.^699^ A β-diketonate scaffold fused to benzene to form a terpyridine like architecture **[EuL**^**bio-45**^**]** was found to rapidly respond to HOCl, resulting in the ejection of Eu(III) from the ligand, causing a decrease in emission.^700^ A terpyridine-based Tb(III) complex which was insentitive to HOCl **[TbL**^**bio-46**^**]** was encapsulated in silica nanoparticles (mSiO_2_). **[EuL**^**bio-45**^**]** was functionalized on the surface of mSiO_2_, forming **[TbL**^**bio-46**^**EuL**^**bio-45**^**]-mSiO**_**2**_, in order to facilitate the ratiometric detection of HClO.^700^ Intracellular exogenous HClO was visualized using **[TbL**^**bio-46**^**EuL**^**bio-45**^**]-mSiO**_**2**_ in RAW 264.7 cells after stimulating the cells with LPS/IFN-γ/PMA. Eu(III) emission decreased while Tb(III) luminescence remained consistent which allowed ratiometric detection of HClO. Macrophage cells respond to bacterial infection, producing abundant HClO in the cytoplasm to kill bacteria.^697^ Therefore, **[TbL**^**bio-46**^**EuL**^**bio-45**^**]-mSiO**_**2**_ was employed to detect endogenous HClO in RAW 264.7 cells which were infected by *Escherichia coli* (*E. coli*). After RAW 264.7 cells and *E. coli* were coincubated, the infected cells were sequentially incubated with **[TbL**^**bio-46**^**EuL**^**bio-45**^**-mSiO**_**2**_ at 37 °C. The infected RAW 264.7 cells exhibited weak red luminescence and bright green luminescence with a 4.33-fold increase of the ratiometric (*I*_green_/*I*_red_) value. It is noteworthy that ratiometric value was larger than that obtained from the LPS/IFN-γ/PMA stimulated RAW 264.7 cells, suggesting that RAW 264.7 macrophage cells are more sensitive to *E. coli* for producing endogenous HClO. *In vivo* studies were performed on *Daphnia magna*, a widely used laboratory animal as an indicator of the health of aquatic ecosystems and as a model organism in ecotoxicology.^701^ Thoracic appendages and gut of *Daphnia magna* exhibited strong luminescence signals. Ratiometric value increased over 6.4-fold after incubation with HClO in the foregut and hindgut were only 0.23-fold and 1.8-fold increased, respectively. This might be ascribed to the convenient contact of the **[TbL**^**bio-46**^**EuL**^**bio-45**^**]-mSiO**_**2**_ with HClO in thoracic appendages and the relatively high concentration of naturally existing bioantioxidants in the esophagus and midgut.^700^

In a separate work, **[EuL**^**bio-45**^**]** was attached to triphenylphosphonium **[EuL**^**bio-47**^**]**^**+**^ or morpholine **[EuL**^**bio-48**^**]** to detect HOCl in mitochondria or lysosomes.^702^ Cellular imaging in HOCl spiked HepG2 and Raw 264.7 cells was established whose localization was verified by costaining experiments. Incubation of 0.25 μM **[EuL**^**bio-47**^**]**^**+**^ in *Daphnia magna* led to intense Eu(III) emission in the gut and thoracic appendages. Further incubation with 50 μM HClO to *Daphnia magna* displayed weakened Eu(III) luminescence in the thoracic appendage but remained bright in the gut.^702^

Aggregation of Eu(III)-diketonate complexes **[EuL**^**bio-49**^**]** has led to self-assembled nanoparticles, **[EuL**^**bio-49**^**]-NP** for ratiometric two-photon probes to achieve sensitive and selective detection of HClO in aqueous solution and living cells.^703^**[EuL**^**bio-49**^**]-NP** coassembled with amphiphilic near-infrared dye IR-780 injected into cells for 1 h at 37 °C emitted a correspondingly weak luminescence emission in red channels under excitation at 780 nm. Nevertheless, when the same cells were treated with 50 μΜ of HClO for 0.5 h, a strong red luminescence signal was observed inside living cells.

Another approach for HClO detection involves a nanocomposite with Rhodamine Rh1000 fluorophores loaded onto the surface of NaYbF_4_:2%Er@NaYF_4_:10%Yb@NaYF_4_:40%Nd,10%Yb to quench the sensitizer Yb(III). The lifetime of the emission at 540 nm from Er(III) was affected largely by the number of attached Rh1000 molecules, proving the greater influence on the apparent luminescent lifetime of Er(III) at 540 nm caused by quenching the Yb(III) excited state. The structure of Rh1000 was degraded in response to HClO, leading to the 980 nm lifetime recovery of the nanocomposite, demonstrating the ability to detect HClO *in vitro*. *In vivo* detection is achieved by monitoring different lifetimes at 540 and 980 nm between the left feet and right feet injection of the nanoprobe into mice feet with l-carrageenan in the left and right feet as controls.^704^ Lanthanide nanoprobes NaGdF_4_:Nd modified with indocyanine green for *in vivo* NIR-II inflammation imaging of HClO in a mouse model.^705^ Indocyanine green allowed NIR activation and imaging with a 5-fold NIR-II fluorescence enhancement at 1049 nm.

Ye, Yuan et al. reported a terpyridine based ligand covalently linked to an *o*-nitroaniline group **[LnL**^**bio-50**^**]** (Ln = Eu(III), Tb(III)).^706^ Owing to the photoinduced electron transfer from the *o*-nitroaniline to the Ln(III) terpyridine moiety, **[LnL**^**bio-50**^**]** is weakly luminescent. In the presence of hypochlorous acid, cleavage of the 4-amino-3-nitrophenyl ether in **[LnL**^**bio-50**^**]** eliminated the photoinduced electron transfer process, thereby enhancing the luminescence intensity. Exogenous HClO in HeLa cell line was visualized by **[LnL**^**bio-50**^**]** after 30 min of incubating the cells containing the complex with HClO. Raw 264.7 macrophages incubated with **[LnL**^**bio-50**^**]** were stimulated with LPS/IFN-γ/PMA to produce endogenous HOCl from H_2_O_2_. Up to 5.8-fold luminescence enhancement of **[EuL**^**bio-50**^**]** was observed upon the addition of the stimulator which indicates the interaction of the probe to HClO in Raw 264.7 macrophages.^706^ In a subsequent work, terpyridine was functionalized with nitrophenylthio group **[LnL**^**bio-51**^**]**^**+**^ to allow photoinduced electron transfer from the Eu(III)-terpyridine moiety to the nitrophenyl group.^707^ Upon the addition of HOCl, the nitrophenyl group of **[EuL**^**bio-51**^**]**^**+**^ was cleaved, enhancing the luminescence from the Eu(III) center. A cationic triphenylphosphonium was functionalized in **[EuL**^**bio-51**^**]**^**+**^ to promote targeting the mitochondria of living cells which was visualized in HepG2 cellline. Endogenous HOCl from Raw 264.7 macrophages was visualized by **[LnL**^**bio-51**^**]**^**+**^. Additionally, *in vivo* studies were performed on a five-day old zebrafish with **[LnL**^**bio-52**^**]**^**–**^ (no triphenylphosphine in this complex) treated with HOCl for 30 min. Strong luminescence were observed from the stomach, yolk and liver of zebrafish, revealing the main localization of the HOCl molecules up-taken by zebrafish during the 30 min HOCl-exposition.^707^

**Figure 36:**
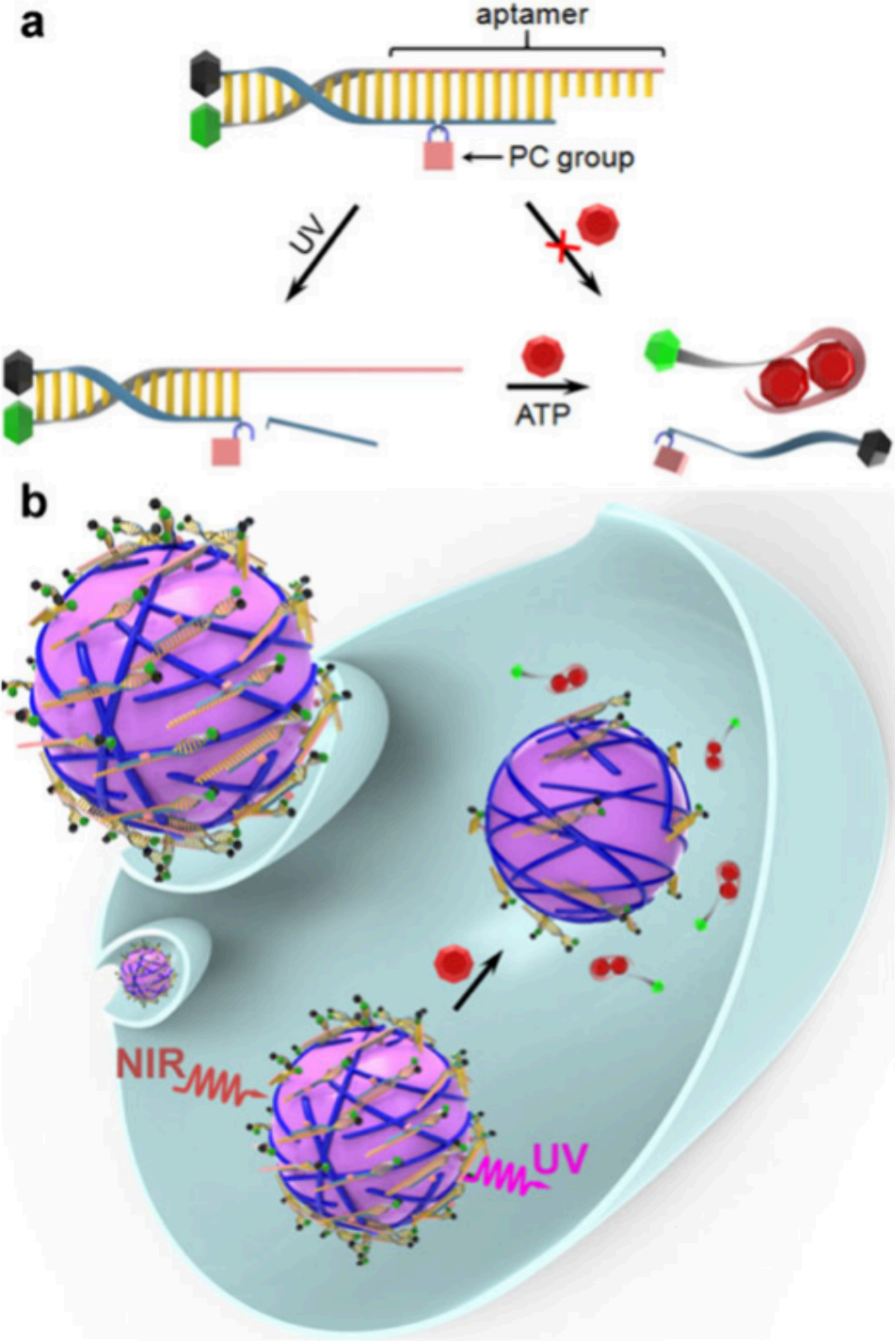
(a) Schematic showing the UV light-activatable ATP sensing mechanism of the aptamer-based probe. (b) Design of DNA nanodevices based on the integration of the aptamer probe with upconversion nanotransducer for NIR-activated intracellular ATP sensing. Reproduced with permission from ref (708). Copyright 2017 American Chemical Society.

UCNP NaGdF_4_:70%Yb,1%Tm@NaGdF_4_ were introduced for ATP sensing in HeLa cells and tumor-bearing mice.^708^ The UCNP is used to initiate photocleavage of an aptamer strand, which is locked by a complementary DNA with a photocleavable inhibitor (Figure 36). Upon light irradiation, short DNA fragments are formed. Only in the presence of ATP does the aptamer restore its structure. The process is monitored with the FRET of a Cy3 dye attached to aptamer.^708^

Detection of nucleic acids in cellular environments is attracting considerable attention for assessment of intracellular disposition of a delivered gene. Optical tracking of DNA release in cells was demonstrated with SiO_2_-coated NaYF_4_:Yb,Er UCNP which releases a DNA-dye. The UCNP acts as a luminescence resonance energy donor to the assembled DNA-binding dye, which acts as the acceptor. DNA attachment to the modified UCNP is confirmed by luminescence-based resonance energy transfer (LRET) between the donor nanoparticle and acceptor POPO-3 dye intercalating the DNA. Exposure of the nanoparticle/DNA complexes with intercalated POPO-3 to 980 nm laser produced a new emission peak at 570 nm (characteristic of the POPO-3 dye), thereby indicating an efficient LRET occurrence between the UCNP and the dye acceptor. Imaging of these nanoparticles in cells was achieved based on tracking of the green UCNP signal in the cytoplasmic and perinuclear regions. Cellular monitoring of the LRET signals demonstrated release of the DNA in HeLa cells and further on monitoring transfection of DNA *in vivo* animal studies.^709^

Hypoxia is a deficiency in the amount of oxygen in tissue and an important parameter in tumors. Thus, the detection of hypoxia is essential for the diagnosis, prognosis, treatment planning, and monitoring response to therapy. A self-assembled nanoprobe UCNP-AuNP, consisting of azo-functionalized upconversion nanoparticles NaYF_4_:18%Yb, 2%Er and gold nanoparticles functionalized with β-cyclodextrin, CD-AuNP, is prepared for ratiometric sensing of hypoxia in living cells. The UCNP-AuNP exhibits only red fluorescence in hyperoxic or normoxic environments. When exposed to hypoxic conditions, various reductases can progressively reduce azo derivatives to aniline derivatives, causing the CD-AuNP to detach and resulting in fluorescence recovery of green emission. Under hypoxic conditions, reductases reduce azo derivatives on the UCNP, leading to detachment of the CD-AuNP and subsequent fluorescence recovery of the green emission. The probe exhibited strong resistance with reductases in biosystem. The use of NIR excitation effectively minimizes interference from strong luminescence backgrounds in biosystems. The UC-AuNP nanoprobe can effectively sense and monitor hypoxia conditions in living cells and has the potential to distinguish hypoxia-related diseases from healthy tissue, making it a valuable tool for early clinical diagnosis.^710^

Lanthanide complexes have been widely studied for detection of ROS, most commonly HO^•^ and O_2_^•–^. A recent review outlines the different systems.^630^ Their detection is usually based on chemical transformation of the organic antenna chromophore which influences the triplet state of the lanthanide or the lanthanide coordination and subsequently the lanthanide signal output. There is considerable interest in monitoring ROS for diagnosis of Alzheimer’s disease.

**Figure 37:**
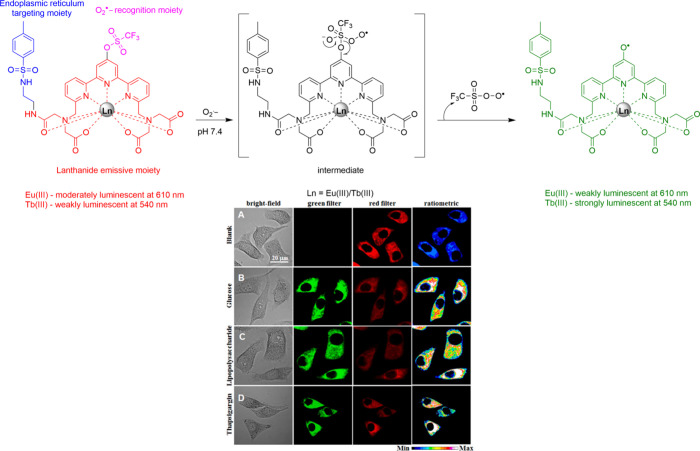
Top: Structure of **[Eu/TbL**^**bio-53**^**]** and the proposed reaction mechanism of the probe with O_2_^•–^. Bottom: Ratiometric time-gated luminescence imaging of endogenously produced O_2_^•–^ in HK-2 cells via cisplatin stimulation using **[Eu/TbL**^**bio-53**^**]** as a probe ratiometric: *I*_green_/*I*_red_. Adapted with permission from ref (711). Copyright 2019 American Chemical Society.

Song, Yuan et al. reported a terpyridine based complex for the detection of superoxide O_2_^•–^ in cells combining ratiometric and time-gated luminescence. An aryl sulfonamide is present in the probe which is used to target the endoplasmic reticulum in cells while a trifluoromethanesulfonyloxy group is present which cleaves upon the detection of O_2_^·–^ (Figure 37). Eu(III) luminescence **[EuL**^**bio-53**^**]** was found to be weak (2.5-fold decrease), while the Tb(III) luminescence **[TbL**^**bio-53**^**]** was enhanced upon the cleavage of trifluoromethanesulfonyloxy group (9-fold increase). However, when both the Eu(III) and Tb(III) complex was mixed **[Eu/TbL**^**bio-53**^**]** and evaluated in cells, a 22-fold ratiometric increase was observed. Cell studies were performed with the complexes on HepG2 cell line along with colocalization studies with ER tracker red. When live cells undergo Endoplasmic Reticulum (ER) stress, ROS are produced in the cells, which can cause further ER stress, forming a vicious cycle. Thus, the endogenous O_2_^•–^ in live HepG2 cells during ER stress initiated by different agents, including a high concentration of glucose, lipopolysaccharide, and thapsigargin, was imaged using **[Eu/TbL**^**bio-53**^**]** as a probe under ratiometric and time-gated luminescence modes (Figure 37). The kidney injury models were prepared in BALB/c mice administrated with cisplatin/gentamicin. Ratiometric analysis reveals changes in **[Eu/TbL**^**bio-53**^**]** with increase in time. Lipopolysaccharide-induced acute inflammation was performed on BALB/c mice to account for the detection of endogenous superoxide formation which was achieved by **[Eu/TbL**^**bio-53**^**]**.^711^

Er(III)-based UCNP with both excitation and emission located in NIR-II window were used for the detection of H_2_O_2_. In the core/shell structured NaErF_4_:Ho@NaYF_4_ nanoparticle, the Er(III) are acting as both sensitizer and emitter to harvest pump photons at 1530 nm and subsequently promote the 980 nm emission, while the Ho(III) dopants can also serve as emitter to generate an efficient upconversion emission at 1180 nm. The microneedle patch sensor for *in vivo* inflammation dynamic detection is developed based on the ratiometric fluorescence (I980/I1180) by combining the effective NIR-II upconversion emission and H_2_O_2_ sensing organic chromophore probe IR1061 under the Fenton catalysis of Fe(II). The luminescence images of the microneedle array were still very clear under the skin tissue.^712^

Chang et al. reported Tb(III)DO3A based peroxy complexes which respond to H_2_O_2_. The time-gated luminescence detection relies on the chemoselective H_2_O_2_-mediated oxidation of boronate to uncage a pendant phenol or aniline. The aryl boronate is an electron withdrawing group and a poor sensitizer to Tb(III) luminescence. Reaction with H_2_O_2_ releases the pinacol cage from **[TbL**^**bio-54**^**]** or boronate-capped aryl carbamate from **[TbL**^**bio-55**^**]** to afford the phenol or aniline which sensitizes Tb(III) luminescence. **[TbL**^**bio-55**^**]** produces a 6-fold luminescence enhancement over **[TbL**^**bio-54**^**]**. The complexes were tested in RAW264.7 macrophages to measure the endogenous production of H_2_O_2_. Both reporters show highly specific turn-on luminescent responses for H_2_O_2_ over these reactive oxygen and/or nitrogen metabolites.^713^

Hydroxyl radical (HO^•^) is the most reactive member of the family of ROS.^714^ Production of HO^•^*in vivo* is a consequence of O_2_ oxidation to superoxide during mitochondrial respiration and enzymes involved in the immune response, primarily NADPH oxidase and xanthine oxidase.^715^ Superoxide dismutase catalyzes the conversion of superoxide to hydrogen peroxide, which interacts with reduced metals, mostly Fe(II) (Fenton reaction) or Cu(I), to produce HO^•^.^716^ The redox properties of HO^•^, and its interactions with reduced metals, link it to disorders involving the accumulation or miscompartmentalization of redox-active metals, including Alzheimer’s, Parkinson’s, and Wilson’s diseases, and the treatment of β-thalassemia and sickle-cell anemia by red blood cell transfusion. Additionally, higher levels of HO^•^ lead to oxidative stress which has been implicated in a variety of pathological conditions.^717^ The exact role of HO^•^ in many of these physiological processes is not well understood, highlighting the need for a chemical probe.

Pierre et al. identified an approach, where **[TbDO3A]** was added to 10-fold excess trimesate, **[TbL**^**bio-56**^**]**, which is a poor sensitizer to Tb(III) (a preantenna).^718^ Upon the addition of HO^•^, trimesate was converted to 2-hydroxytrimesic acid which chelated to **[TbDO3A]** in a bidendate fashion, replacing the two inner-sphere water and increasing luminescence. A linear enhancement in Tb(III) luminescence was observed upon the steady addition of HO^•^ in the femtomolar range. This experiment was also performed in the presence of glutamate to look at the competition between 2-hydroxytrimesic acid and glutamate to the Tb(III) center to observe negligible effects. The interaction of **[TbL**^**diag-56**^**]** with HO^•^ is exclusive over H_2_O_2_, O_2_, ClO^–^, ^1^O_2_, *t*-BuO^•^, and NO as demonstrated by no change in the luminescence intensity.^718^ In a subsequent work, this interaction was studied in detail with six different preantenna to identify trimesamide-**[TbDO3A]**, **[TbL**^**bio-57**^**]** to have the most sensitive detection of HO^•^ with a 1000-fold increase in time-delayed luminescence upon hydroxylation of the preantenna to 2-hydroxytrimesamide. This increase in metal-centered luminescence is devoid of the decrease in the hydration number of **[TbDO3A]**. This suggests that the antenna is interacting with Tb(III) center via a second sphere coordination environment or that coordination by the antenna occurs by displacement of one or more of the carboxylate arms of DO3A.^719^ The cleavage of *p*-aminophenol on a terpyridine based complex was also used to detect hydroxide radicals in HeLa cells.^720^

Carbon monoxide (CO) is one of the important gasotransmitter molecules in mammalian cells, although it is a known pollutant or toxic molecule.^721^ In living systems, CO plays a significant role in the regulation of vasodilation, neurotransmission, antiapoptotic, anti-inflammatory, and antiproliferative activities. Emerging studies revealed that mitochondria could be a major organelle for the action of CO to regulate cellular respiration.^722^ Song et al. reported a Tb(III) terpyridine complex conjugated to 4-nitrobenzyl group **[LnL**^**bio-58**^**]**^**+**^. On treating with CO, the nitrobenzyl group was cleaved which turned on Tb(III) luminescence, but Eu(III) was weakly luminescent.^723^ Therefore, a ratiometric approach **[Eu/TbL**^**bio-58**^**]**^**+**^ was used to monitor CO. Triphenylphosphine in the ligand allowed localization in the mitochondria of HeLa cells. Upon increasing CO concentration in HeLa cells (done using a commercially available tricarbonylchloro (glycinato)Ru(II) which releases CO), green emission from **[Eu/TbL**^**bio-58**^**]**^**+**^ was observed with ratiometric enhancement (*I*_540_Tb(III)/*I*_610_Eu(III)) from 0.12 to 5.05 in the mitochondria of the cell. Endogenous CO production in mitochondria of Raw264.7 cells was also observed. *Ex vivo* studies in mouse liver tissue showed a 20-fold ratiometric enhancement upon treatment with CO. *In vivo* studies on *Daphne magna* found **[Eu/TbL**^**bio-58**^**]**^**+**^ in the gut containing CO. Further, *in vivo* studies on BALB/c nude mouse was performed with **[Eu/TbL**^**bio-58**^**]**^**+**^ injected in the left rear leg followed by sequential injection of CO which showed a 5.8-fold high ratiometric intensity than the right leg which proved the ability of **[Eu/TbL**^**bio-58**^**]**^**+**^ to image CO *in vitro* and *in vivo*.^723^

Nitric oxide is a highly reactive, uncharged free radical which plays crucial roles in human physiology as an intra and extracellular messenger molecule.^724,725^ Lysosomal functions are subtly regulated by NO including the degradation of a cell’s own components in the catabolic autophagy process to provide energy and nutrients for cell growth through the lysosomal machinery.^726^ Alterations in the level of lysosomal NO were associated with a variety of diseases, such as Gaucher’s disease,^727^ Danon disease,^728^ and lysosomal storage disorders.^729^ Therefore, there is a need to develop chemosensors for the detection and understanding of NO. Although a range of fluorescent probes have been developed for the detection of NO, they do not localize in the lysosome.^730^ Yuan et al. was the first to report an Eu(III)-based probe for NO detection. This was achieved by using a terpyridine ligand bearing *o*-diaminophenol **[EuL**^**bio-59**^**]**^**–**^ which is a PET quencher of Eu(III) luminescence.^731^ Upon the addition of NO in aerobic conditions, *o*-diaminophenol transforms into benzotriazole derivative which lifts the PET effect, thereby enhancing Eu(III) luminescence. In Onion’s inner-layer epidermal peels, **[EuL**^**bio-59**^**]**^**–**^ was used for the time-gated luminescence detection of NO. However, **[EuL**^**bio-59**^**]**^**–**^ is irreversible after the addition of NO.^731^

Later, Yuan et al. reported a Tb(III) terpyridine complex which was conjugated to 5-carboxytetramethylrhodamine **[TbL**^**bio-60**^**]**^**–**^. The spirolactam derivative in Rhodamine is nonfluorescent, leaving the Tb(III) luminescence from terpyridine undisturbed.^732^ Upon the addition of NO, the ring of the spirolactam is opened, leading to intramolecular luminescence resonance energy transfer from Tb(III) terpyridine to rhodamine, which enhances rhodamine fluorescence, and concomitantly decreases Tb(III) luminescence. The average luminescence lifetime of **[TbL**^**bio-60**^**]**^**–**^ was altered in different NO concentrations which was used as a signal responding to changes in NO concentration. Ratiometric analysis of **[TbL**^**bio-60**^**]**^**–**^ (*I*_565_ Rh/*I*_540_ Tb(III)) in HepG2 cells reveal localization in an isolated juxtanuclear area in the cytoplasm of the cells. However, upon the incubation of NO to HepG2 cells already incubated with **[TbL**^**bio-60**^**]**^**–**^, the NO reacted **[TbL**^**bio-60**^**]**^**–**^ was localized in the lysosome of the cell. *In vivo* studies in *Daphnia magna* identify **[TbL**^**bio-60**^**]**^**–**^ in the intestine at higher concentration than the abdomen.^732^

A luminescence resonance energy transfer type approach was used in apoferritin for the detection of NO. This involves **[TbL**^**bio-61**^**]**^**–**^ as the energy donor and rhodamine derivative as an energy acceptor encapsulated in apoferritin and covalently bound on the surface of apoferritin, forming **[TbL**^**bio-61**^**]**.^733^ Emission in **[TbL**^**bio-61**^**]** while rhodamine displays weaker emission in **[TbL**^**bio-61**^**]**. Upon the addition of nitric oxide, the emission of rhodamine is switched on due to the ring opening of the spirolactam while Tb(III) luminescence in **[TbL**^**bio-61**^**]** decreases. These effects were used ratiometrically *I*_Rh_/*I*_Tb_ for the detection of NO. Cellular studies in HepG2 cells and *in vivo* studies in *Daphne magna* show initial Tb(III) luminescence, which was reduced and rhodamine fluorescence turned on upon the addition of NO.^733^

Peroxynitrite (ONOO^–^) is a highly reactive species generated spontaneously between superoxide (O_2_^–^) and nitric oxide (NO). In cells, ONOO^–^ is generated predominantly in the mitochondria and is involved in cellular signaling processes, via nitration of tyrosine residues on proteins and nitrosylation of protein thiols.^734,735^ However, elevated levels of ONOO can lead to significant oxidative and nitrosative damage to lipids, proteins, and DNA and has been implicated in several diseases including Alzheimer’s, Huntington’s, and Parkinson’s diseases.^736^

Yuan et al. reported a peroxynitrile-responsive probe which is a terpyridine based complex bearing a dimethyl resorcinol derivative, **[LnL**^**bio-62**^**]**^**–**^.^737^ The Eu(III) and Tb(III) are highly luminescent, but upon ONOO^–^ addition, Tb(III) luminescence was lowered, but Eu(III) luminescence remained consistent. Therefore, a ratiometric approach of combining Eu(III) and Tb(III) complexes **[Eu/TbL**^**bio-62**^**]**^**–**^ were used to image ONOO^–^ in HeLa cells.^737^

Butler et al. reported a quinoline based phenyl boronic acid tethered to a heptadentate Eu(III) complex **[EuL**^**bio-63**^**]**.^738^ Upon the addition of ONOO^–^ to **[EuL**^**bio-63**^**]**, rapid oxidative cleavage of the phenylboronic acid (*k* ≈ 10^6^ M^–1^ s^–1^) is observed, which quenches energy transfer to the Eu(III) center, leading to diminished luminescence (a turn-off approach). Time-resolved luminescence with **[EuL**^**bio-63**^**]** in human serum albumin (using a plate reader assay) revealed a selective turn off response to ONOO^–^ over H_2_O_2_. Cellular uptake studies in HeLa cells suggest localization of **[EuL**^**bio-63**^**]** in mitochondria and no cytotoxicity was observed during the time frame of the imaging experiment. HeLa cells stained with **[EuL**^**bio-63**^**]** were incubated with an ONOO^–^ donor for 30 min to observe a 90% decrease in emission intensity. In THP cell line, the emission intensity of **[EuL**^**bio-63**^**]** decreased by 45% in 5 s treatments of ONOO^–^, while 25% decrease was observed in Jurkat cell line under the same conditions, indicating a lower initial level of ONOO^–^ in Jurkat cells. Thus, **[EuL**^**bio-63**^**]** can be used to visualize elevated levels of ONOO^–^ in different cell lines following extracellular treatment with a plasma therapy device.^738^

**Figure 38:**
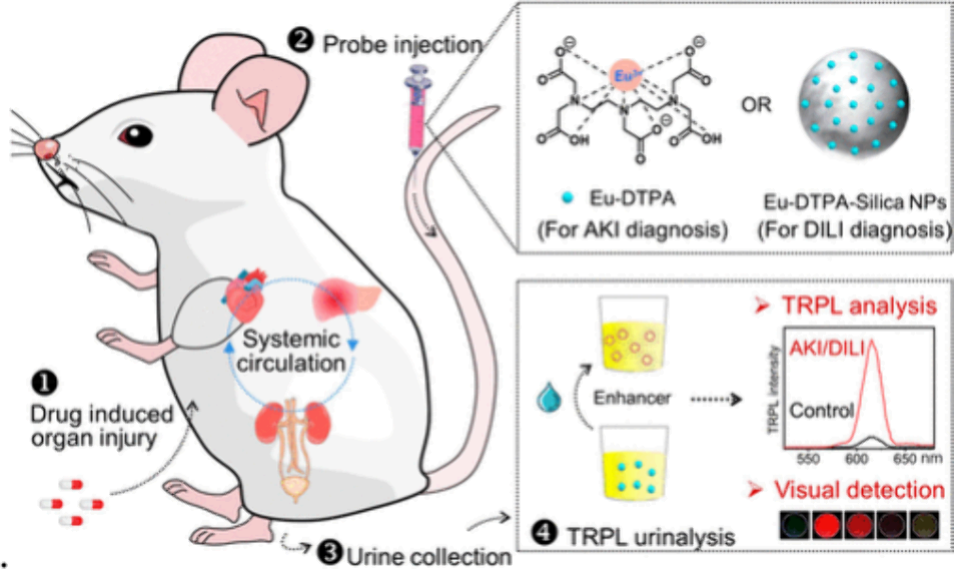
Schematic illustration of the engineering of lanthanide bioprobes for urinary TRPL diagnosis of mice organ injuries. The synthesized Eu–DTPA complex and Eu–DTPA-integrated silica nanoprobes were designed for the diagnosis of AKI and DILI, respectively. TRPL = time-resolved photoluminescence. Reproduced with permission from ref (739). Copyright 2023 American Chemical Society.

A nanoparticle system based on diethylene triamino pentaacetic acid (DTPA) and SiO_2_–NP has been introduced for urinary diagnosis of diseases such as drug-induced liver injury (DILI) and acute kidney injury (AKI)^739^ (Figure 38). Following injection of Eu–DTPA it was found that in AKI mice the Glomerular filtration rate was decreased. The analysis was based on a time-resolved luminescence assay following dissociaition of Eu(III) from the complex upon addition of 2-naphthoyltrifluoroacetone, trioctylphosphine oxide (TOPO), Triton X-100, and acetic acid which results of Eu entering the formed micelles. Eu-DTPA was also encapsulated in SiO_2_–NP which were modified with disulfide cross-linkers to enable ONOO^–^-specific biodegradation and Eu–DTPA release. ONOO^–^ is a sensitive biomarker overexpressed at the early status of liver injury. The nanoprobe was injected in DILI mice and was found that it was accumulated in liver tissues undergoing ONOO^–^-responsive degradation. The released Eu-DTPA is excreted into urine for detection.

Selenocysteine is a vital intermediate in selenium metabolism,^740^ and can attenuate oxidative damage through scavenging oxidants or chelating redox metals.^741,742^ Besides free Selenocysteine, the vast majority endow proteins with a wide range of functions in redox homeostasis, thyroid hormone metabolism, immune responses, cell maintenance, and skeletal muscle regeneration.^743^ The overexpression or dysfunction of selenoproteins is associated with cancer and diabetes, as well as neurodegenerative and cardiovascular diseases. Yuan et al. reported a terpyridine-based Tb complex linked to a 2,4-dinitrophenol, which was cleaved upon reaction with selenocysteine **[TbL**^**bio-64**^**]**^**+**^, resulting in enhancing Tb(III) luminescence, but Eu(III) luminescence remained weak.^744^ Therefore, a ratiometric approach of combining Eu(III) and Tb(III) complex **[Tb/EuL**^**bio-64**^**]**^**+**^ was used to visualize Selenocysteine in the mitochondria of HepG2 cells. The ratiometric value (*I*_Tb_/*I*_Eu_) in mitochondria gradually increased from 0.39 to 5.43 after the cells were incubated with Selenocysteine. *In vivo* studies on a diabetes induced liver injured balb/c mouse was performed ratiometrically which showed an increase in Tb(III) luminescence which was found otherwise on a normal balb/c mouse.^744^ Wang et al. utilized a different approach, where they used 2,4-dinitrobenzenesulfonates as an inhibitor to selenoproteins **[TbL**^**bio-65**^**]**. Tb(III) luminescence was turned on upon the cleavage of 2,4-dinitrobenzenesulfonate by selenol. This was visualized in A2870 cells upon incubation with different concentrations of **[TbL**^**bio-65**^**]**.^745^ More works on luminescent lanthanide probes for detecting reactive species in cellular environments have been extensively reviewed elsewhere.^220,663,664^

Intracellular oxidation characterized by excessive ROS generation also cause cell death either induced by inflammation or oxidative stress. Therefore, the ROS-sensitive probe offers great abilities for tracking stem cell viability *in vivo*. An activated NIR-II fluorescent nanoparticle consisting of lanthanide-based down-conversion nanoparticles, (DCNP NaYF_4_:20% Yb, 2%Er@NaYF_4_ and IR786s (a ROS-sensitive probe (DCNP@IR786s) for cell labeling and real-time tracking of adipose-derived mesenchymal stem cell (ADSC) viability *in vivo* has been developed. In dying cells due to excessive ROS generation, absorption competition-induced emission of IR786s was destroyed, which could turn on the NIR-II fluorescent intensity of DCNP at 1550 nm by 808 nm laser excitation. In contrast, the NIR-II fluorescence intensity of DCNP was stable at 1550 nm by 980 nm laser excitation. This ratiometric fluorescent signal was precise and sensitive for tracking ADSC viability *in vivo*. Significantly, the nanoparticle could be applied to quantitively evaluate stem cell viability in real-time *in vivo*. Two small molecules including glutathione and dexamethasone that could improve stem cell engraftment efficiency and enhance ADSC therapy in a liver fibrotic mouse model.^746^

## 6 Conclusion/Outlook

In the course of this review, we have illustrated the development of a set of powerful tools for understanding physiology, initiating therapeutic activity and stretching the limits of bioassay. The rich power of the lanthanide luminescence signal is evidenced in *in vivo* applications of multiplexing time-resolved assays, intervention on biochemical pathways in optogenetics, localized forms of therapy, and early results in guided surgical interventions. However, exploiting the potential of lanthanide containing systems to the full requires developing a better understanding of how to control cellular uptake and cytotoxicity before full scale biomedical application can be achieved. For broad application, it is likely to be essential to develop complexes that can be addressed using conventional microscope optics, meaning that excitation at wavelengths longer than 320 nm is required. This also means that multiphoton excitation and upconversion processes give scope for further exploitation. Downshifting and upconverting nanoparticle designs are accompanied by coating manipulation to increase biocompatibility and uptake and are dominating the tissue and bioresponsive studies.

While almost all the imaging studies have been done using fluorescence/luminescence microscopy, there is great scope to explore circularly polarized luminescence. The inherent chirality of designed lanthanide complexes can also be exploited in understanding the building blocks of life. We have already mentioned the use of circular polarized luminescence from lanthanide complexes, and it is clear that such approaches have been under-utilized when dealing with the differentiation of biomarkers and in multiplexed analysis. The emerging technique of CPL microscopy is potentially a powerful addition to the range of available methods of addressing lanthanide complexes. At its simplest, chiral guests can be useful as chiral auxiliaries to induce chirality (and hence CPL) into racemic lanthanide complexes. Furthermore, chiral complexes can be used to tune selectivity for particular guests, whose luminescence pattern will alter upon chiral induction from optically active species in biology. This would enable selective imaging of chiral species in question, particularly in cases where chiral biomolecules induce chirality at a lanthanide center. Chiral lanthanide probes may lead to selective membrane protein interactions and selective receptor mediated uptake but also have the potential of monitoring intracellular transport mechanisms and monitoring processes with biomolecule precision that will inform on mechanisms and activity *in cellulo*.

When designing luminescent probes, complexes that possess both a highly hydrophobic moiety and are cationic tend to show significant nonspecific protein binding. Therefore, they are less attractive when creating targeted probes, or when seeking to bind selectively to a cell-surface receptor or target a protein binding pocket. Similarly, nanoparticles’ targeting is altered by surfaces coated with polymers which may have nonspecific binding interactions.

It is important to note that virtually all systems display selectivity for analytes, as opposed to specificity: there are always going to be competitor interferent species. The properties of lanthanide ions suggest that this is not necessarily a disadvantage. Given the ease of multiplexing and parallel addressing of systems with varying luminescence lifetimes, a combination of probes with varying affinities and selectivities for a panel of different lanthanides could easily be used to facilitate personalized diagnosis in combination with artificial intelligence and machine learning to deconvolute complex information and guide effective therapy. In nanoparticle designs, super resolution optical methods are also opening the way for optimized detection of UCNP and NIR to NIR downshifting nanoparticle systems which can lead the way of localized therapy and imaging approaches.

As we enter the next phase of the development of lanthanide probes, there is clear scope to move from specialist facilities into routine clinical diagnosis, and there remain plenty of reasons to be excited about the excited states of lanthanide ions.
